# User-Generated Online Health Content: A Survey of Internet Users in the United Kingdom

**DOI:** 10.2196/jmir.3187

**Published:** 2014-04-30

**Authors:** Braden O'Neill, Sue Ziebland, Jose Valderas, Francisco Lupiáñez-Villanueva

**Affiliations:** ^1^Nuffield Department of Primary Care Health SciencesUniversity of OxfordOxfordUnited Kingdom; ^2^Medical SchoolUniversity of ExeterExeterUnited Kingdom; ^3^Department of Information and Communication ScienceUniversitat Oberta de CatalunyaBarcelonaSpain

**Keywords:** eHealth, consumer health information, patient education, health education, health promotion, social media, superusers

## Abstract

**Background:**

The production of health information has begun to shift from commercial organizations to health care users themselves. People increasingly go online to share their own health and illness experiences and to access information others have posted, but this behavior has not been investigated at a population level in the United Kingdom.

**Objective:**

This study aims to explore access and production of user-generated health content among UK Internet users and to investigate relationships between frequency of use and other variables.

**Methods:**

We undertook an online survey of 1000 UK Internet users. Descriptive and multivariate statistical analyses were used to interpret the data.

**Results:**

Nearly one-quarter of respondents (23.7%, 237/1000) reported accessing and sharing user-generated health content online, whereas more than 20% (22.2%, 222/1000) were unaware that it was possible to do this. Respondents could be divided into 3 groups based on frequency of use: rare users (78.7%, 612/778) who accessed and shared content less than weekly, users (13.9%, 108/778) who did so weekly, and superusers (7.5%, 58/778) who did so on a daily basis. Superusers were more likely to be male (*P*<.001) and to be employed (*P*<.001), but there were no differences between the groups with respect to educational level (*P*=.99) or health status (*P*=.63). They were more likely to use the Internet for varied purposes such as banking and shopping (*P*<.001).

**Conclusions:**

Although this study found reasonably widespread access of user-generated online health content, only a minority of respondents reported doing so frequently. As this type of content proliferates, superusers are likely to shape the health information that others access. Further research should assess the effect of user-generated online content on health outcomes and use of health services by Internet users.

## Introduction

### Background

There can be little doubt that the Internet has changed the way that people experience health and illness. People routinely use the Web to learn about the meaning of symptoms, tests, diagnoses and treatments, and to find out how others have rated doctors and hospitals [1]. In the United Kingdom, more than two-thirds of residents use the Internet to obtain health information [2], and the UK Department of Health considers availability of high-quality information a key policy objective [3]. The Internet once provided information that could be accessed and viewed, but not easily modified. Patients increasingly go online to share information and to access information others have posted on the Internet, including their own experiences. Although this is a widespread practice, especially for long-term conditions [4], relatively little is known about the characteristics of people who share and access user-generated content (UGC) online. This is in contrast to general online health information-seeking behavior which has been extensively studied [1,3,4]. In this paper, we draw on a major European survey to explore who shares and accesses user-generated health content online in the United Kingdom.

### User-Generated Health Content

User-generated health content is presented in many different ways across different types of websites [5]. Traditional, commercially produced online health content (developed by businesses or health systems or governments) has predominantly consisted of facts and figures, such as numerical data related to the diagnosis or prognosis of a condition. User-generated health information, by virtue of its distributed, often informal development and dissemination, is a broad concept and incorporates anything that a patient or carer posts online for the purpose of others seeing it. Entwistle et al [6] noted that online health information can be broadly divided into 2 categories: general facts and personal experiences. Although these categories are not mutually exclusive, the differences have important implications. General facts consist of information about particular conditions, treatments, or services, and are intended to be broadly applicable to many people interested in the same topic. Personal experiences may also provide information about conditions, treatments, or services, but are often in narrative form and portray an individual’s experience of undergoing a particular treatment or of living with a long-term condition. People tend to draw on both general facts and personal experiences, depending on the issue involved [6,7]. It has been noted that adequate information is an important aspect of health care delivery that matters to patients [8].

General health information sites often provide UGC in the form of curated experiences selected by the site’s developers and the ability for site users to post their own comments. There are sites exclusively devoted to interview-based research on health experiences; for example, the UK’s Healthtalkonline has been available for more than a decade and now has many counterparts in other countries [9]. Blogs enable people to post their stories over time and provide the facility to embed video and links to other content, including peer-reviewed journal articles where applicable. Social networking sites enable patients to post content (both facts and experiences) and have pages devoted to specific conditions and treatments. Online forums host pages for specific conditions and facilitate patients coming together who have similar diseases or have undergone similar treatments. Some sites include crowdsourcing: “an approach to accomplishing a task by opening up its completion to broad sections of the public” [10]. For example, the website PatientsLikeMe aggregates data provided by site users. Patients can compare their data with aggregated input from other site users, and data obtained through crowdsourcing can be used for research [11]. Finally, there are those sites that enable a health service user to write and read reviews of specific hospitals, doctors, or health systems [12]. Increasingly, websites include several different ways of incorporating content from patients and the public. What all these disparate sources share is that their content depends on people posting, sharing, and comparing health information, whether about themselves or those for whom they care.

### Use and Nonuse

One of the predominant places where UGC is accessed and shared online is in the context of virtual support groups for particular conditions as well as individual treatments. The heterogeneity of online support group membership has been reported in several publications; these groups consist of some people who post occasionally and some who are frequent prolific posters. Those who post have been described as key players [13], active users [14], and caretakers [15]. Most people who visit support groups do not actively post. In a study of people accessing an online smoking cessation group, 84.7% of those who registered and accessed the resource at least once never posted [16]. To explain this distribution, the “1% rule” has been adapted from marketing literature to the use of health social networks, suggesting that 90% of actors observe but do not participate, 9% rarely contribute, and 1% create most of the content [17]. This is contextual, and people’s behavior may vary between sites if they visit several different sites. Adams [18] has suggested that people may be more motivated to add their own commentary if they feel that their experiences are not covered by others accounts (eg, adverse drug effects). Because the content on these websites is generated by a small proportion of the people who use it, the concept of the “superuser” [17,19] of user-generated online health information—the minority of people who post information online—has received recent attention. This has not been characterized within the United Kingdom.

### The Aim of Our Research

The purpose of this paper is twofold. First, we identify characteristics of UK Internet users who access and share UGC online. In this study, we use UGC to denote 4 specific behaviors about which questions were asked in the survey: (1) participating in an online support group for people who are concerned about the same health or medical issue, (2) participating in social networking sites talking about health and wellness, (3) describing a medical condition or problem online to get advice from other online users, and (4) disclosing medical information on social networking sites.

Second, we investigate the frequency with which respondents engage in the aforementioned study behaviors, paying particular attention to those who do so most frequently because these users predominantly generate the content that others view. By grouping respondents according to how frequently they go online to access and share UGC, we aim to characterize the differences between these groups. We aim to determine areas for further research to support the effective and beneficial use of online health information.

## Methods

### Survey Instrument and Ethics

We analyzed United Kingdom data from the Citizens and Information Communication Technology for Health survey, a project undertaken in 2011 by the Institute for Prospective Technology Studies of the European Commission’s Joint Research Centre. This online survey was developed from a theoretical framework of the social determinants of information and communication technology (ICT) for health, translated into native languages in 14 European Union (EU) member countries. The survey was developed to understand and characterize European citizens’ use of ICT for health. It included questions on a range of triggers, motivations, and behaviors. This analysis examines a subset of questions related to user-generated online health content. The full questionnaire is available in Multimedia Appendix 1 [20].

Technical, methodological, and legal considerations were carefully addressed in the context of designing and implementing the survey. These considerations ensured anonymity and confidentiality of individual responses [21-23]. The survey was conducted in accordance with European Society for Opinion and Marketing Research ethical guidelines [24]. At the time the survey was carried out, one of the authors (FLV) was employed by the European Commission.

### Sample and Data Collection

The sample consisted of 1000 respondents from the United Kingdom who completed an online survey in 2011 covering a variety of domains regarding the use of ICT for health. We randomly sampled people aged 16 to 74 years who had used the Internet in the 3 months before the survey to obtain the sample.

An online invitation to participate in the survey was sent to 7291 individuals. The data were collected between July 20 and August 1, 2011. Simple random sampling was carried out by the software provider who administered the survey (Cint), then quota sampling was used to accurately reflect the demographic composition of UK residents [25]. We obtained 2410 responses (4881 nonresponses) of which 1320 were out of quota and 90 were excluded because of missing data. The average length of time to complete the questionnaire was 20.5 minutes.

More than half (57.00%, 570/1000) of respondents were employed and nearly all (90.00%, 900/1000) had completed upper secondary or tertiary education (Table 1). This is broadly comparable to the characteristics of the UK population, although it should be noted that this sample is more highly educated than the general UK population [25].

**Table 1:** Demographic composition of sample.

Age group (years)	Female, n (%)	Male, n (%)	Total, n (%)
16-24	103 (10.3)	106 (10.6)	209 (20.9)
25-54	297 (29.7)	295 (29.5)	592 (59.2)
55-74	98 (9.8)	101 (10.1)	199 (19.9)
Total	498 (49.8)	502 (50.2)	1000 (100.0)

### Statistical Analysis

Initially, we calculated descriptive statistics about the characteristics of the study participants. After this, to determine the extent to which the 4 behaviors studied represent an underlying composite variable, we employed principal components analysis (PCA). In carrying out PCA, we a priori defined components explaining adequate amounts of variability in the data as those that have an eigenvalue greater than 1.00 [26]. Because the results of the PCA suggested that all 4 behaviors could be explained by one underlying component, for further analysis we treated this as a single composite variable, which we called “accessing and sharing user-generated health content online.”

We then used nonhierarchical *k*-means cluster analysis to characterize respondents based on their reported frequency of accessing and sharing UGC online. We determined differences between the frequency groups by carrying out ANOVA and chi-square tests for categorical and continuous variables, respectively. Data were examined to determine if they met assumptions for the univariate and multivariate statistical tests carried out, and all assumptions were met. Statistical analyses were performed using SPSS version 20.0 (IBM Corp, Armonk, NY, USA).

## Results

### Frequency of Engaging in Individual Study Behaviors

We found that approximately one-quarter of all respondents participated in online support groups (27.80%, 278/1000) and health-related social networking sites (23.80%, 238/1000) (Table 2). A similar proportion reported describing a medical condition or problem online to get help from other online users (23.10%, 231/1000) and disclosing medical information on social networking sites (16.70%, 167/1000).

Less than 10% of respondents (4.80%, 48/1000 to 7.80%, 78/1000) reported engaging in any of the study behaviors at least or more than once a week. Approximately 20% of respondents (22.20%, 222/1000) reported being unaware that it was possible to engage in these behaviors online.

**Table 2:** User-generated content behaviors.

Regarding health, wellness, and the Internet, how often have you...	Frequency, n (%)					
	Never	Less than once a month	At least once a month (but not every week)	At least once a week (but not every day)	Every day or almost every day	Not aware of it
Participated in an online support group for people who are concerned about the same health or medical issue	588 (58.8)	145 (14.5)	66 (6.6)	46 (4.6)	21 (2.1)	134 (13.4)
Participated in social networking sites talking about health and wellness	629 (62.9)	106 (10.6)	54 (5.4)	53 (5.3)	25 (2.5)	133 (13.3)
Described a medical condition or problem online in order to get advice from other online users	628 (62.8)	119 (11.9)	64 (6.4)	25 (2.5)	23 (2.3)	141 (14.1)
Disclosed medical information on social networking sites	660 (66.0)	72 (7.2)	40 (4.0)	33 (3.3)	22 (2.2)	173 (17.3)

### Characterizing Predictors of Accessing and Sharing User-Generated Content

Applying cluster analysis to the “accessing and sharing user-generated health content online” composite variable resulted in a 3-cluster solution. After excluding those who did not know that these resources were available (excluded because they have different reasons for nonuse than those who are aware of the possibility and choose to not engage) [26], we were left with 778 responses for further analysis.

Most respondents (78.7%, 612/778) were rare users who reported that they never or very rarely accessed and shared user-generated health content. Users who went online and engaged in these behaviors infrequently (ie, weekly or monthly but not daily) accounted for more than 10% (13.9%, 108/778) of respondents. A further minority (7.5%, 58/778) were clustered as superusers who accessed and/or generated UGC daily.

### Sociodemographic Characteristics

Comparing the 3 groups, superusers were more likely to be male than users or rare users (Table 3). Those in the 16-24 years age group were more likely to be superusers than users or rare users, whereas those in the 55-74 years age group were generally rare users, as opposed to superusers or users. Superusers were more likely to be employed (57.8%, 33/58) than unemployed (9.3%, 5/58), students (19.0%, 11/58), or retired/inactive in the labor force (24.0%, 13/58). The groups did not differ on level of education.

Regarding household composition, respondents with children (1-parent, 2-parent, or extended family households) were less likely to be superusers than they were to be users or rare users. There were no differences between the groups based on the number of children younger than 16 years in the household. More rare users and superusers reported that they lived in intermediate areas, as opposed to densely or thinly populated areas, whereas users were more likely to report living in densely populated areas.

**Table 3:** Sociodemographic characteristics of respondents (N=778).

Sociodemographic characteristics		Rare users, n (%) n=612	Users, n (%) n=108	Superusers, n (%) n=58	*P*
**Gender**					.02
	Male	301 (49.3)	61 (56.5)	38 (67.2)	
	Female	311 (50.7)	47 (43.5)	20 (32.8)	
**Age (years)**					<.001
	16-24	89 (14.7)	39 (36.1)	25 (43.1)	
	25-54	370 (60.6)	63 (58.3)	32 (55.2)	
	55-74	151 (24.7)	6 (5.6)	1 (1.7)	
**Level of education completed**					.99
	Primary or lower secondary education (ISCED 0-2)^a^	70 (11.4)	14 (13)	7 (12.1)	
	Upper secondary education (ISCED 3 or 4)^a^	261 (42.6)	45 (41.7)	26 (44.8)	
	Tertiary education (ISCED 5 or 6)^a^	281 (45.9)	49 (45.4)	25 (43.1)	
**Occupation**					<.001
	Employed or self-employed (including family workers)	354 (57.8)	72 (66.7)	31 (53.4)	
	Unemployed	57 (9.3)	6 (5.6)	11 (19.0)	
	Student (not in the labor force)	54 (8.8)	21 (19.4)	11 (19.0)	
	Other not in the labor force (eg, retired, inactive)	147 (24.0)	9 (8.3)	5 (8.6)	
**Type of locality**					<.001
	Densely populated area (cities and large towns)	186 (30.4)	52 (48.1)	18 (31.0)	
	Intermediate area (towns)	308 (50.3)	43 (39.8)	36 (62.1)	
	Thinly populated area (village and rural)	118 (19.3)	13 (12)	4 (6.9)	
**Number of members in the household**					.13
	1	81(13.2)	11 (10.2)	7 (12.1)	
	2	224 (36.6)	36 (33.3)	12 (20.7)	
	3	123 (20.1)	22 (20.4)	19 (32.8)	
	≥4	184 (30.1)	39 (36.1)	20 (34.5)	
**Number of children under 16 years**					.08
	None	436 (71.2)	67 (62)	32 (55.2)	
	1	92 (14.9)	23 (21.3)	12 (22.4)	
	2	74 (12.1)	14 (13.0)	10 (17.2)	
	>2	11 (1.8)	4 (3.7)	3 (5.2)	
**Household composition**					.02
	Single person ≥65 years	14 (2.3)	1 (0.9)	0 (0)	
	Single person <65 years	67 (10.9)	10 (9.3)	8 (13.8)	
	2 persons, ≥1 aged ≥65 years	69 (11.3)	6 (5.6)	2 (3.4)	
	2 persons, both <65 years	148 (24.2)	26 (24.1)	8 (13.8)	
	Single person with child(ren) <16 years	7 (1.1)	5 (4.6)	1 (1.7)	
	2 persons with child(ren) <16 years	161 (26.3)	34 (31.5)	20 (34.5)	
	2 persons ≥65 years	27 (4.4)	2 (1.9)	3 (5.2)	
	Extended family (<16 + 16-64 + <65 years)	6 (1.0)	3 (2.8)	3 (5.2)	
	≥3 adults <65	113 (18.5)	21 (19.4)	13 (22.4)	

^a^ISCED: International Standard Classification of Education [27].

### Health Characteristics

We did not identify any differences between groups for health status, undergoing long-term medical treatment, or number of visits to the doctor in the past 12 months (Table 4). Among superusers who reported having someone close to them currently experiencing long-term illness or disability, 64.3% (37/58) reported taking care of them.

**Table 4:** Health characteristics of respondents (N=778).

Health characteristics		Rare users, n (%) n=612	Users, n (%) n=108	Superusers, n (%) n=58	*P*
**Health status reported**					.63
	Very bad	3 (0.5)	1 (0.9)	1 (1.7)	
	Bad	45 (7.4)	7 (6.5)	6 (10.3)	
	Neither good or bad	115 (18.8)	24 (22.2)	6 (10.3)	
	Good	297 (48.5)	53 (49.1)	28 (48.3)	
	Very good	152 (24.8)	23 (21.3)	17 (29.3)	
**Long-standing illness or health problem reported**					.57
	Yes	263 (42.9)	41 (37.5)	25 (43.9)	
	No	349 (57.1)	68 (62.5)	33 (56.1)	
**Undergoing a long-term medical treatment**					.74
	Yes	215 (35.1)	35 (32.1)	18 (31.6)	
	No	397 (64.9)	73 (67.9)	40 (68.4)	
**Visit a doctor during the past 12 months**					.29
	Yes	512 (83.7)	95 (88.0)	52 (89.7)	
	No	100 (16.3)	13 (12.0)	6 (10.3)	
**How many times did you visit a doctor during the last 12 months?**					.64
	None	100 (16.3)	13 (12)	6 (10.3)	
	1-2 visits	216 (35.3)	31 (28.7)	21 (36.2)	
	3-4 visits	118 (19.3)	27 (25.0)	11 (19.0))	
	5-6 visits	70 (11.4)	15 (13.9)	8 (13.8)	
	>6 visits	108 (17.6)	22 (20.4)	12 (20.7)	
**Is someone close to you currently experiencing long-term illness or disability?**					.01
	Yes	227 (37.1)	56 (51.5)	29 (50.0)	
	No	385 (62.9)	52 (48.5)	29 (50.0)	
**Are you taking care of such a person?**					.02
	Yes	234 (38.2)	39 (35.8)	37 (64.3)	
	No	378 (61.8)	69 (64.2)	21 (35.7)	

### Online Health Information and Health Behavior

Superusers were more likely than users or rare users to report that the information they accessed affected the way they care for themselves and the way they eat or exercise (Table 5). They were also more likely to report that after accessing health information on the Internet, they subsequently talked to a doctor or nurse about what they found. Finally, superusers were more likely to report accessing information for their children than users or rare users.

**Table 5:** Online health information and health behavior of respondents (N=778).

Online health information and health behavior		Rare users, n (%) n=612	Users, n (%) n=108	Superusers, n (%) n=58	*P*
**Looking for health and/or wellness information for...**					
	Yourself	531 (86.7)	102 (94.4)	56 (96.6)	.01
	Your child	152 (24.9)	32 (29.6)	26 (44.8)	.004
	Parent	158 (25.8)	48 (44.4)	27 (46.6)	<.001
	Another relative	194 (31.7)	41 (38.0)	24 (41.4)	.19
	Some else	155 (25.3)	39 (36.1)	24 (41.4)	.005
**Did you later talk to a doctor or nurse about the information you got online?**					<.001
	Yes	234 (38.3)	65 (59.8)	43 (74.1)	
	No	378 (61.7)	43 (40.2)	15 (25.9)	
**Overall, how useful was the health information you got online?**					.02
	Not at all useful	15 (2.5)	2 (1.9)	0 (0)	
	Not too useful	55 (9.0)	5 (4.6)	1 (1.7)	
	Somewhat useful	400 (65.3)	71 (65.7)	33 (56.9)	
	Very useful	142 (23.2)	30 (27.8)	24 (41.4)	
**Did the information you got online affect any of your decisions about health treatments or the way you take care of yourself?**					<.001
	Yes	201 (32.8)	56 (51.9)	38 (65.5)	
	No	411 (67.2)	52 (48.1)	20 (34.5)	
**Did the information you got online affect the way you eat or exercise?**					<.001
	Yes	171 (28.0)	56 (52.0)	40 (69.8)	
	No	441 (72.0)	52 (48.0)	18 (30.2)	

### Internet Activity

Almost all superusers (94.8%, 55/58) reported engaging in 11 or more Internet activities (eg, online banking, keeping a blog, and looking for travel information), whereas approximately one-quarter of rare users (26.6%, 163/612) reported the same engagement with other Internet activities (Table 6). Similarly, nearly three-quarters of superusers (74.1%, 43/58) reported accessing the Internet through 3 or more devices (eg, a home computer, work computer, and smartphone), which contrasts with less than one-third (29.4%, 180/612) of rare users doing the same.

**Table 6:** Internet activities and devices of respondents (N=778).

Internet activities and devices		Rare users, n (%) n=612	Users, n (%) n=108	Superusers, n (%) n=58	*P*
**Breadth of Internet use (number of Internet activities)**					<.001
	1-5	109 (17.8)	2 (1.9)	1 (1.7)	
	6-10	340 (55.6)	25 (23.1)	2 (3.4)	
	≥11	163 (26.6)	81 (75)	55 (94.8)	
**Breadth of Internet access (number of Internet devices)**					<.001
	1	203 (33.2)	15 (13.9)	7 (12.1)	
	2	229 (37.4)	27 (25.0)	8 (13.8)	
	≥3	180 (29.4)	66 (61.1)	43 (74.1)	

## Discussion

### Principal Findings

Within the past few years, a rapid expansion of technologies that people can use to generate their own Internet content has provided novel opportunities for members of the general population to share and access health information. Social media have facilitated this ever-growing shift from the production of health information online being in the hands of commercial enterprise and health systems, to being led by users themselves. This study, the first to investigate user-generated health content on the Internet in the United Kingdom, sought to understand this growing trend through people’s responses to survey questions. One of the more striking findings in this study is that one-quarter of this sample of UK Internet users reported accessing and/or posting user-generated health information online. Because this is the first study of its kind in the United Kingdom, there are no equivalent data from which to study trends.

As has been repeatedly shown in studies of online health support groups, most participants do not actively post their own content, although they read what others have written [13-17]. Although it is not possible to directly measure the activity of these “lurkers,” they represent such a large proportion of those engaged with UGC that we should see this as “normal” online behavior. Further exploration of the motivations and usage of UGC by these passively engaged individuals is required. By grouping responses to this survey according to the frequency with which people reported accessing and sharing UGC, we sought to understand the characteristics of people who are highly engaged in these behaviors—the superusers. Although our data do not provide information on the effects of UGC, our findings characterizing the 7.5% of respondents classified as superusers is valuable in describing the group who may be generating the content that others are viewing.

These superusers are predominantly male. Respondents in the youngest age category (16-24 years) are more likely to be superusers, whereas those in the oldest age category (55-74 years) are generally rare users. Superusers carry out more varied activities on the Internet, such as online banking and booking travel, than the users and rare users. Despite these differences, our results suggest that there are no differences in health status or health service use between the superusers and the other 2 groups. This suggests that people who are well may be as responsible for producing our online health content as people who are ill. To our knowledge, this is the first explication that UGC does not appear to be associated with self-reported use of health services. This study did not distinguish between the use of information that provides guidance for a diagnosis or treatment of a health condition, and information shared or accessed by people who are healthy and either seeking or offering lifestyle advice. Several possible mechanisms have been identified by which UGC and, in particular, others’ experiences may affect health, such as finding information, feeling supported, and experiencing health services [5]. Therefore, our results are congruent with the contingent model of health information use, which suggests that health information is one important component of a health care experience, rather than as something that displaces or reduces use of health services [28].

It was surprising that about 20% of participants were unaware of the availability of UGC on the Internet, particularly as this was a sample of Internet users. The outcomes of UGC are unclear; therefore, we would not necessarily recommend interventions to increase access. Further research should focus on reasons why these individuals, who have access to the Internet and are motivated to complete a health survey, are unaware of the availability of this increasingly ubiquitous content.

Previous studies of health-seeking behavior on the Internet have found that females more commonly access health information on the Internet than males [1,4]. However, in the present study, superusers were more likely to be male. It has been suggested that men rate their self-assessed online skills higher than women do, which is one possible explanation for higher reported use of these complex online resources by men [29]. Although our data did not provide an opportunity to examine this issue further, it is an important area for future research as it guides content development and implementation.

### Limitations

The survey used in this study was administered online. Administering a survey in this way enables rapid data collection, reduces administrative burden, and is cost-effective. It may cause selection bias because those who do not have Internet access as well as adequate digital skills are excluded [30]. In this study, rare users may have been excluded because of a lack of digital skills. Those with chronic illness may also have been less inclined to participate in an online survey or excluded because of fatigue or disability. Another limitation is the use of quota sampling (done to facilitate international comparisons in the overall survey), which reduces the generalizability of the results of this analysis to the overall population when compared with a true random sample. Although the sample was selected to be representative of UK Internet users, subsequent studies with different designs might sample purposively for superusers to explore their behavior and motivations more fully. Further research focusing on smaller age categories and going deeper into some of the life-cycle variables that may affect use of online health information (eg, parenting or retirement) would be useful as well. Furthermore, this study reports a secondary analysis of a larger survey on people’s use of the Internet for health. Some potential areas of inquiry relevant to UGC were not included in the original survey, such as its use to obtain social support and to select health services based on others’ experiences [5]. Finally, this study reports cross-sectional data, which precludes determining causal relationships. Because this is the first study of its kind in the United Kingdom, it is exploratory in nature and does not evaluate effects of UGC.

### Conclusions

This study reports results of the first representative sample of UK Internet users that investigates accessing and sharing user-generated health content online. Within the context of available surveys from other countries, our results suggest that UGC may be increasingly popular among those who are healthy and who have chronic conditions alike, and that a minority of people who frequently access and share may be primarily responsible for generating the majority of content that others view.

The potential benefits of ICT for health are vast, but it is likely that some online resources are effective and desirable for some people and they are not for others. Understanding the contexts in which they are helpful is important to be able to support individual patients, public health initiatives, and to develop information policy and strategy in clinics and health systems. Through understanding characteristics of those who already participate in accessing and sharing user-generated health content online, as well as the differences between groups of users based on the frequency with which they do so, the results of this study bring clarity to this important issue regarding use of ICT for health information. These findings inform an agenda for further research to identify why people access and share UGC, what the impact is on health behaviors and outcomes, and if expanding engagement with user-generated health content online is likely to be beneficial.

## Multimedia Appendix 1

"Citizens and Information Communication Technology for Health" survey.

## Abbreviations

EU: European Union
ICT: information and communication technology
PCA: principal components analysis
UGC: user-generated content
