# Supplementary material for: User-Generated Online Health Content: A Survey of Internet Users in the United Kingdom
Source: J Med Internet Res. 2014 Apr 30;16(4):e118. doi: 10.2196/jmir.3187 (PMC4019776; doi:10.2196/jmir.3187)
Supplement: Supplementary file 1 [file jmir_v16i4e118_app1.pdf]

European Commission

JRC SCIENTIFIC AND POLICY REPORTS

Strategic Intelligence Monitor on Personal Health Systems, Phase 2

Citizens and ICT for Health in 14 European Countries: Results from an Online Panel

Authors: Francisco Lupiañez, Ioannis Maghiros, Fabienne Abadie

Editors: Fabienne Abadie, Maria Lluch, Francisco Lupiañez,
Ioannis Maghiros, Elena Villalba, Bernarda Zamora

2013

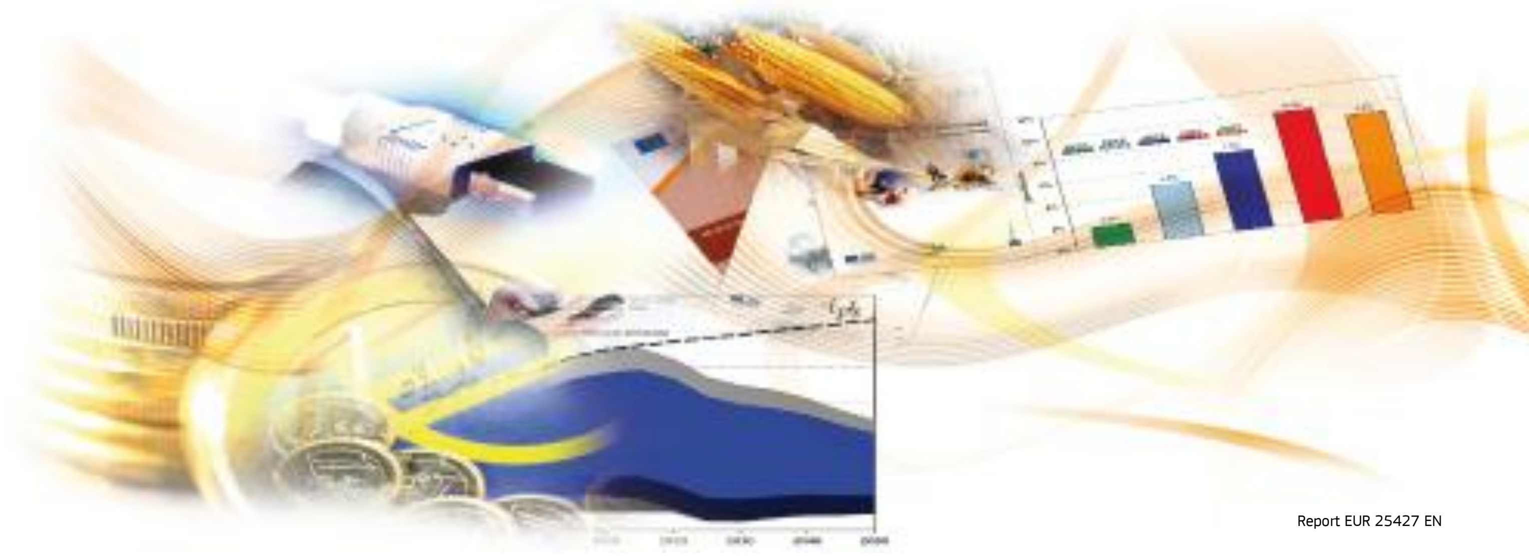

Report EUR 25427 EN

Joint Research Centre

European Commission
Joint Research Centre
Institute for Prospective Technological Studies

Contact information
FAddress: Edificio Expo. c/Inca Garcilaso, 3. E-41092 Seville, Spain
E-mail: jrc-ipts-secretariat@edc.europa.eu
Tel.: +34 954488318
Fax: +34 954488300

http://ipts.jrc.ec.europa.eu/
http://www.jrc.ec.europa.eu/

This publication is a Scientific and Policy Report by the Joint Research Centre of the European Commission.

Legal Notice
Neither the European Commission nor any person acting on behalf of the Commission
is responsible for the use which might be made of this publication.

Europe Direct is a service to help you find answers to your questions about the European Union
Freephone number (*): 00 800 6 7 8 9 10 11
(*) Certain mobile telephone operators do not allow access to 00 800 numbers or these calls may be billed.

A great deal of additional information on the European Union is available on the Internet.
It can be accessed through the Europa server http://europa.eu/.

JRC71142

EUR 25427 EN

ISBN 978-92-79-25709-4 (pdf)

ISSN 1831-9424 (online)

doi:10.2791/84062

Luxembourg: Publications Office of the European Union, 2013

© European Union, 2013

Reproduction is authorised provided the source is acknowledged.

Printed in Spain

Table of Contents

SUMMARY **7**
Background 7
Conceptual framework: Towards social determinants of ICT for Health 7
Online panel survey technical information 8
ICT access 9
ICT for Health Motivation 10
ICT for Health usage 11
ICT for Health Impact 11
Social determinants of ICT for Health: key dimensions 13
Lessons learned 15

1 INTRODUCTION **17**
1.1 Background and rationale of SIMPHS2 17
1.2 Policy context 17
1.2.1 From eEurope to Digital Agenda for Europe 17
1.2.2 Healthcare and ageing in the new policy context toward 2020 18
1.2.3 European Innovation Partnership on Active and Healthy Ageing 21
1.3 Conceptual framework: towards a social determinants of ICT for Health 21
1.4 Outline of the report 25

2. METHODOLOGY **27**
2.1 Questionnaire design 27
2.2 Survey design and sampling 27
2.3 Field work process 32
2.4 Data analysis 35

3. SOCIO-DEMOGRAPHIC PROFILE OF THE PARTICIPANTS **37**
3.1 Gender 37
3.2 Age 38
3.3 Country of citizenship 39
3.4 Country of birth 39
3.5 Level of education 40
3.6 Labour position 41
3.7 Type of location 42
3.8 Members in the household 44
3.9 Socio-demographic comparison: Internet users (sample) and population 45

4. HEALTH STATUS AND HEALTH AND SOCIAL CARE UTILISATION **49**
4.1 Health status 49
4.2 Informal carers 61
4.3 Health and social care demand 64
4.4 Quality of care 66

5. ICT ACCESS **75**
5.1 Internet access and frequency of use 75
5.2 Internet-related activities 79

6. HEALTH INFORMATION SOURCES AND TRUST **85**

7. ICT FOR HEALTH: MOTIVATIONS AND BARRIERS **91**
7.1 Triggers 91
7.2 Empowerment 94
7.3 Barriers 103

8. ICT FOR HEALTH ACCESS **107**
8.1 ICT for Health utilisation 107
8.2 ICT for Health willingness and awareness 118

9. INTERNET HEALTH INFORMATION **121**
9.1 Internet health information utilisation 121
9.2 Factors for the evaluation of an Internet health site 139

10. ICT FOR PARTICIPATORY HEALTH **143**

11. FROM QUESTIONNAIRE ITEMS TO CONCEPTUAL DIMENSIONS: MULTIVARIATE ANALYSIS: **153**
11.1 ICT access dimensions 153
11.2 ICT for Health Motivation dimensions 154
11.2.1 Triggers dimensions 154
11.2.2 Empowerment dimensions 154
112.3 Barriers dimensions 156
11.3 Health information sources and trust dimensions 156
11.3.1 Health information sources dimensions 156
11.3.2 Trust dimensions 157
11.4 ICT for Health access dimensions 157
11.4.1 ICT for Health readiness dimensions 157
11.4.2 ICT for Health willingness dimensions 159
11.4.3 ICT for Health assessment dimensions 160
11.4.4 ICT for Health impact dimensions 161

12. CONCLUSIONS **163**

REFERENCES **167**

ANNEXES **169**
Annex 1. Questionnaire and coding manual 169
Annex 2. Online panel providers 189
Annex 3. Pilot study 191
Annex 4. Internet activities comparison 193
Annex 5: Correlation matrix 196

List of Figures

Figure 1 *The Virtuous Cycle of the digital economy* 20
Figure 2 *Final form of the Commission on Social Determinants of Health conceptual framework* 22
Figure 3 *A Causal and Sequential Model of Digital Technology Access by Individuals in Contemporary Societies* 23
Figure 4 *Social Determinants of Health and ICT for Health conceptual framework* 24
Figure 5 *Gross response rates* 34
Figure 6 *Net response rate* 35
Figure 7 *Interview length (minutes)* 35
Figure 8. *Gender (E1)* 37
Figure 9. *Gender (E1) by country* 37
Figure 10. *Age (E2)* 38
Figure 11. *Age (E2) by country* 38
Figure 12. *Country of citizenship (E3)* 39
Figure 13. *Country of birth (E4)* 39
Figure 14. *Level of education completed (E5)* 40
Figure 15. *Level of education completed (E5) by country* 41
Figure 16. *Labour position (E6)* 41
Figure 17. *Labour position (E6) by country* 42
Figure 18. *Type of locality (E9)* 43
Figure 19. *Type of locality (E9) by country* 43
Figure 20. *Members in the household (E19)* 44
Figure 21. *Members in the household (E19) by country* 44
Figure 22. *Health status (A4)* 49
Figure 23. *Health status (A4) by country* 51
Figure 24. *Long-standing illness or health problem (A5)* 51
Figure 25. *Long-standing illness or health problem (A5) by country* 53
Figure 26. *Long-term medical treatment (A6)* 53
Figure 27. *Long-term medical treatment (A6) by country* 55
Figure 28. *Limited in activities people normally do due to a health problem (A7)* 55
Figure 29. *Limited in activities people normally do due to a health problem (A7) by country* 57
Figure 30. *Health problems reported (A8)* 58
Figure 31. *Someone close to you currently experience long-term illness of disability (A9)* 61
Figure 32. *Someone close to you currently experience long-term illness of disability (A9) by country* 62
Figure 33. *Taking care of a person experience long-term illness or disability (A10)* 62
Figure 34. *Taking care of a person experience long-term illness or disability (A10) by country* 64
Figure 35. *Health and social care demand (A1, A2, A3)* 64
Figure 36. *In general, how often does your usual source of care (doctor or nurse)... (A11)* 66
Figure 37. *In general, how often does your usual source of care (doctor or nurse) explain to you the results of medical exams (A11) by country* 68
Figure 38. *In general, how often does your usual source of care (doctor or nurse) explain to you different treatment options (A11) by country* 69
Figure 39. *In general, how often does your usual source of care (doctor or nurse) listen to your opinion and takeyour preferences into account to choose treatments (A11) by country* 69
Figure 40. *In general, how often do you ask your usual source of care (doctor or nurse).(A12)* 70
Figure 41. *In general, how often do you ask your usual source of care (doctor or nurse) to explain to you the results of the medical exams? (A12) by country* 72

Figure 42. In general, how often do you ask your usual source of care (doctor or nurse) to explain to you the different treatment options? (A12), by country 72
Figure 43. In general, how often do you ask your usual source of care (doctor or nurse) to consider your opinion and your preferences when choosing treatments? (A12), by country 73
Figure 44. Internet access and use (C1) 75
Figure 45. Internet activities (C2) 79
Figure 46. Health information sources (B4) 85
Figure 47. Trust (B5) 88
Figure 48. ICT for Health motivations and triggers (D11) 91
Figure 49. Empowerment and ICT for Health (B1) 94
Figure 50. Empowerment and ICT for Health (B2) 97
Figure 51. Empowerment and ICT for Health (B3) 100
Figure 52. ICT for Health barriers (D13) 103
Figure 53. Social life of Internet health information (D2) 121
Figure 54. Internet health information and doctor's consultation (D3) 123
Figure 55. Internet health information and doctor's consultation (D4) 124
Figure 56. How useful was the health information you got online? (D5) 128
Figure 57. How useful was the health information you got online? (D5) by country 130
Figure 58. Did you learn anything NEW from the information you got online, or not? (D6) 131
Figure 59. Did you learn anything NEW from the information you got online, or not? (D6) by country 132
Figure 60. Did you later talk to a doctor or nurse about the information you got online? (D7) 132
Figure 61. Did you later talk to a doctor or nurse about the information you got online? (D7) by country 134
Figure 62. Did the information you got online affect any of your decisions about health treatments or the way you take care of yourself? (D8) 134
Figure 63. Did the information you got online affect any of your decisions about health treatments or the way you take care of yourself? (D8) by country 136
Figure 64. Did the information you got online affect the way you eat or exercise? (D9) 136
Figure 65. Did the information you got online affect the way you eat or exercise? (D9) by country 138
Figure 66. Internet website evaluation (D12) 139
Figure 67. Internet health information consequences (D14) 143
Figure 68. ICT for Health consequences (D15) 146
Figure 69. Dimensions of Social determinants of ICT for Health 163
Figure 70. Complexity of Social determinants of ICT for Health dimensions 164

List of Tables

Table 1. Technical information 27
Table 2. Target population, by country, gender and age 28
Table 3. Sample by country, gender and age 29
Table 4. Sampling error, by country, gender and age 30
Table 5. Weighting factors by country 31
Table 6. Sample by region 31
Table 7. Sampling errors by region 32
Table 8. Weighting factors by region 32
Table 9. Data collection schedule 33
Table 10. Indicators of the fieldwork process 34
Table 11. Socio-demographic comparison 45
Table 12. Socio-demographic comparison by country (I) 46

Table 13. Socio-demographic comparison by country (II)........47
Table 14. Health status (A4) by socio-demographics........50
Table 15. Long-standing illness of health problem (A5) by socio-demographics........52
Table 16. Long-term medical treatment (A6) by socio-demographics........54
Table 17. Limited in activities people normally do, because of a health problem (A7)........56
Table 18. Health problems (A8) by socio-demographic........59
Table 19. Health problems (A8) by country........60
Table 20. Someone close to you, currently experiencing long-term illness or disability (A9) by socio- demographic........61
Table 21. Taking care of a person experience long-term illness or disability (A10) by socio-demographic........63
Table 22. Health and social care demand (A1, A2, A3) by socio-demographic........65
Table 23. In general, how often does your usual source of care (doctor or nurse) (A11) by socio-demographic........67
Table 24. In general, how often do you ask your usual source of care (doctor or nurse) (A12) by socio-demographic........71
Table 25. Internet access and use (C1) by socio-demographics........77
Table 26. Internet access and use at home (C1) by country........78
Table 27. Internet access and use at work (C1) by country........78
Table 28. Internet access and use somewhere else(C1) by country........79
Table 29. Internet activities (C2) by socio-demographics........81
Table 30. Internet activities (C2) by socio-demographics........82
Table 31. Internet activities (C2) by country........83
Table 32. Health information sources (B4) by socio-demographics........86
Table 33. Health information sources (B4) by country........87
Table 34. Trust (B5) by socio-demographics........89
Table 35. Trust (B5) by country........90
Table 36. Information and Communication Technology motivations and triggers (D11) by socio-demographics........92
Table 37. Information and Communication Technology motivations and triggers (D11) by socio-demographics........93
Table 38. Empowerment and ICT for Health (B1) by socio-demographic........95
Table 39. Empowerment and ICT for Health (B1) by socio-demographic........96
Table 40. Empowerment and ICT for Health (B2) by socio-demographic........98
Table 41. Empowerment and ICT for Health (B2) by country........99
Table 42. Empowerment and ICT for Health (B3) by socio-demographics........101
Table 43. Empowerment and ICT for Health (B3) by country........102
Table 44. ICT for Health barriers (D13) by socio-demographics........104
Table 45. ICT for Health barriers (D13) by country........105
Table 46. Internet for Health utilisation (D1a)........108
Table 47. Internet for Health utilisation (D1a) by socio-demographics (I)........110
Table 48. Internet for Health utilisation (D1a) by socio-demographics (II)........111
Table 49. Internet for Health utilisation (D1a) by country........112
Table 50. Information and Communication Technology utilisation (D10a)........114
Table 51. Information and Communication Technology utilisation (D10a) by socio-demographics........115
Table 52. Information and Communication Technology utilisation (D10a) by socio-demographics........116
Table 53. Information and Communication Technology utilisation (D10a) by socio-demographics by country........117
Table 54. Internet for Health utilisation (D1b)........118
Table 55. Information and Communication Technology willingness (D10b)........119
Table 56. Internet for Health utilisation (D2) by socio-demographics........122

Table 57. *Internet for Health utilisation (D2) by country* ... 123
Table 58. *Did you happen to go looking for this health information for yourself? by socio-demographic variables (D3) by socio-demographics* ... 125
Table 59. *Did you happen to go looking for this health information for yourself?, by socio-demographic variables (D3) by country* ... 126
Table 60. *Did you happen to go looking for this health information for another person?, by socio-demographic variables (D3) by socio-demographics* ... 127
Table 61. *Did you happen to go looking for this health information for another person?, by country (D3) by country* ... 128
Table 62. *How useful was the health information you got online? (D5) by socio-demographics* ... 129
Table 63. *Did you learn anything NEW from the information you got online, or not? (D6) by socio-demographics* ... 131
Table 64. *Did you later talk to a doctor or nurse about the information you got online? (D7) by socio-demographics* ... 133
Table 65. *Did the information you got online affect any of your decisions about health treatments or the way you take care of yourself? (D8) by socio-demographics* ... 135
Table 66. *Did the information you got online affect the way you eat or exercise? (D9) by socio-demographics* ... 137
Table 67. *Internet website evaluation (D12) by socio-demographics* ... 140
Table 68. *Internet website evaluation (D12) by country* ... 141
Table 69. *Internet health information consequences (D14) by socio-demographics* ... 144
Table 70. *Internet health information consequences (D14) by country* ... 145
Table 71. *ICT for Health consequences (D15) by socio-demographics* ... 148
Table 72. *ICT for Health consequences (D15) by socio-demographics (II)* ... 149
Table 73. *Internet health information consequences (D15) by country* ... 150
Table 74. *Factor analysis - Internet related activities* ... 153
Table 75. *Factor analysis – Triggers* ... 154
Table 76. *Factor analysis – Empowerment* ... 155
Table 77. *Factor analysis – Barriers* ... 156
Table 78. *Factor analysis – Importance of Health information sources* ... 156
Table 79. *Factor analysis – Importance of Health information sources* ... 157
Table 80. *Factor analysis – ICT for Health readiness* ... 158
Table 81. *Factor analysis – ICT for Health willingness* ... 159
Table 82. *Factor analysis – ICT for Health willingness* ... 160
Table 83. *Factor analysis – ICT for Health willingness* ... 161
Table 84. *Cronbach's a (alpha) values* ... 191
Table 85. *Internet access (C2) comparison* ... 193
Table 86. *Internet activities (C3) comparison* ... 193
Table 87. *Internet activities (C3) comparison* ... 194
Table 88. *Internet activities (C3) comparison* ... 195
Table 89. *Internet related activities - Correlation matrix* ... 196
Table 90. *Triggers - Correlation matrix* ... 198
Table 91. *Empowerment - Correlation matrix* ... 199
Table 92. *Barriers - Correlation matrix* ... 201
Table 93. *Health information sources - Correlation matrix* ... 201
Table 94. *Trust - Correlation matrix* ... 202
Table 95. *ICT for Health readiness - Correlation matrix* ... 203
Table 96. *ICT for Health willingness - Correlation matrix* ... 205
Table 97. *ICT for Health assessment - Correlation matrix* ... 210
Table 98. *ICT for Health impact - Correlation matrix* ... 210

SUMMARY

Background

The Citizen Panel Survey carried out in SIMPHS2 to better assess users and patients' needs and expectations with regard to ICT for health, directly supports the objectives of the Digital Agenda in the area of eHealth which are to both cope with societal challenges and create opportunities for innovation and economic growth by reducing health inequalities, promoting active and healthy ageing and increasing empowerment. It also contributes to the goals of the European Innovation Partnership on Active and Healthy Aging which addresses the societal challenge of an ageing population focusing on the main areas of life events (Prevention, Care and cure and Independent living) with the following expected results:

- An improvement of the health status and quality of life of Europeans, especially older people;
- An improvement of the sustainability and efficiency of health and social care systems;
- Boosted EU competitiveness through an improved business environment for innovation

In this policy context the analysis of users' demand undertaken through the SIMPHS2 Citizen panel survey aims to:

- develop typologies of digital healthcare users and measure the impact of ICT and the Internet on health status, health care demand and health management.
- identify factors that can enhance or inhibit the role and use of Personal Health Systems from a citizen' s perspective with special emphasis on mHealth, RMT, disease management, Telecare, Telemedicine and Wellness.

To reach these objectives, we started by defining a theoretical framework for policy-making, which was used to design and gather relevant information. A multivariate statistical analysis was subsequently carried out to identify the underlying conceptual dimensions emerging from the data collected. Key relationships between concepts (underlying dimensions) were identified to understand ICT for Health as a complex ecosystem. We concluded with some lessons learned.

Conceptual framework: Towards social determinants of ICT for Health

Two frameworks are at the root of our own conceptual framework "**Towards social determinants of ICT for Health".** One is the WHO Commission on Social Determinants of Health Framework which summarises how *“social, economic and political mechanisms give rise to a set of socioeconomic positions, whereby populations are stratified according to income, education, occupation, gender, race/ethnicity and other factors; these socioeconomic positions in turn shape specific determinants of health status (intermediary determinants) reflective of people’s place within social hierarchies; based on their respective social status, individuals experience differences in exposure and vulnerability to health-compromising conditions*”. While this framework does not relate directly to ICT for Health, the structural determinants perfectly overlap the core argument of personal and positional categories of and distribution of resources in van Dijk's "Causal and Sequential Model of Digital Technology Access by individuals in Contemporary Societies" which is the second framework in which our approach is rooted.

As a result and as illustrated in the next we defined "**Towards social determinants of ICT for Health"** as follows:

- Social determinants of health and health inequalities, therefore structural and intermediary determinants produce different levels of ICT access (motivation, material, skills and usage).
- Unequal access to ICT will generate different levels of ICT for Health access as well as different levels of willingness to use ICT for Health.
- ICT for Health access depends on the properties of ICT and the relationship among Motivation; ICT for Health readiness and Internet Health information.

- Motivation includes Triggers, Empowerment and Barriers
- ICT for Health readiness includes Awareness, Material access; Skills and Usage
- ICT for Health Assessment includes how individuals use and evaluate this type of technologies for themselves or for others (social life of information) as well as their perception about usefulness and learning.
- ICT for Health Access gives rise to different level of Participatory Health through the utilisation (individually and socially) of ICT for Health in daily life and behavioural changes due to the ICT for Health impact on: Health management; Health care demand and Health care quality
- These impacts could modify both structural and intermediary determinants and distribution of health and well-being.

Social Determinants of Health and ICT for Health conceptual framework

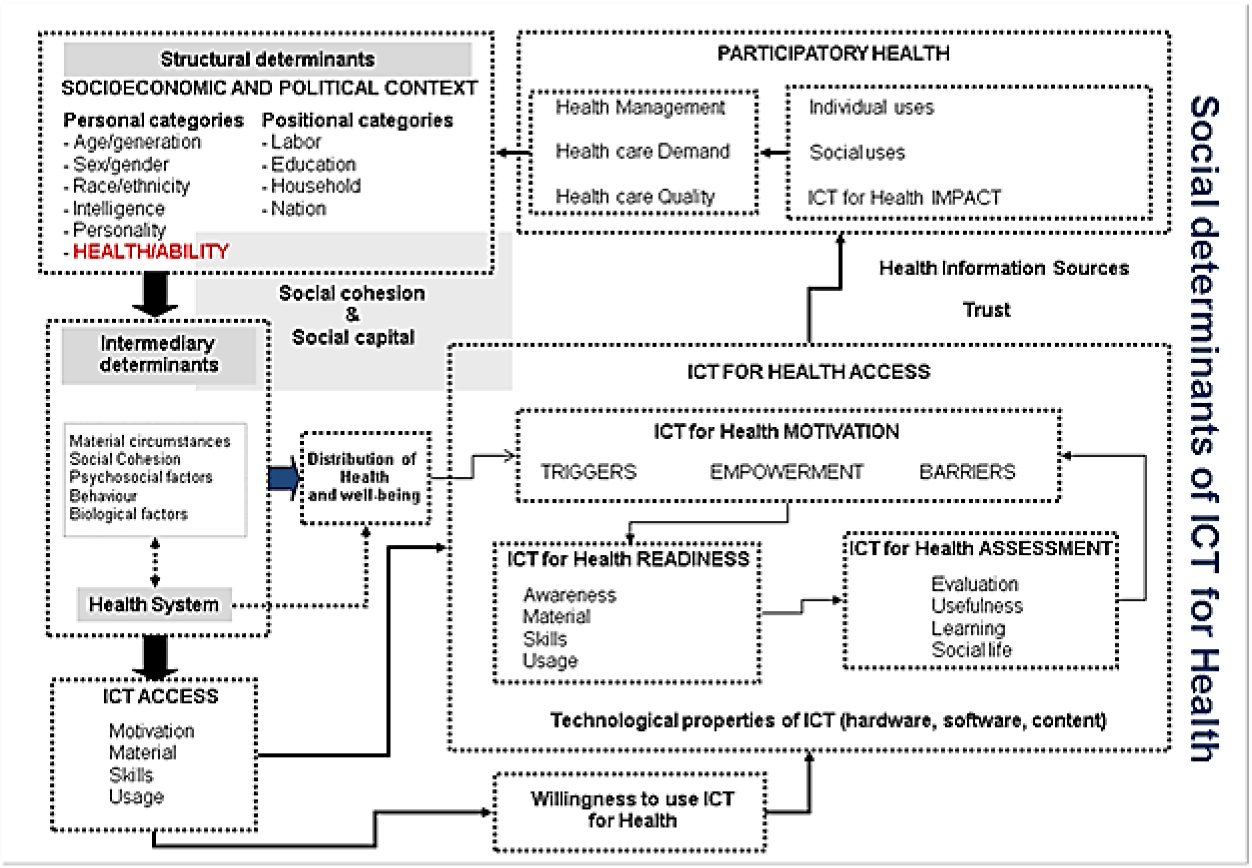

Source: Authors' elaboration based on WHO and van Dijk.

<u>Online panel survey technical information</u>

Based on the above framework, we gathered data through a questionnaire which we designed and structured around five main blocks[1]:

- Block A: Health status and health care and social care services use
- Block B: ICT for Health Motivation and Health Information sources
- Block C: ICT Access

[1] Questionnaire items are listed in supplementary materials at Annex 1. Questionnaire and coding manual.

- Block D: ICT for Health Readiness and Evaluation
- Block E: Socio demographic profile of participants

To reach our target population, we have used the Internet as a methodological tool. Survey research is becoming a frequently used methodology due to the advancement of computer hardware, software and increasing access to the Internet. Furthermore, online surveys offer a valid alternative to the postal, telephone or face-to-face surveys as long as technical, methodological, ethical and legal considerations are taken into account. Table 1 resumes the technical characteristics of the study.

Technical information

Population	Citizens aged from 16 to 74 years old who have used the Internet in the last three months.
Scope of countries	Austria, Belgium, Germany, Denmark, Estonia, Finland, France, Italy, Netherlands, Sweden, Slovenia, Slovakia, Spain, United Kingdom
Type of survey	Online
Sample size	1,000 interviews per country.<br>14,000 interviews in total.
Quotas	Country<br>Gender (Female/Male)<br>Age Group (16-24/ 25-54/ 55-74)
Sampling error	±0.85% for overall data and ±3.16% for country-specific data. In all cases, a maximum indeterminate probability (p=q=50), for a confidence level of 95.5% is applicable for each one of the reference populations
Weighting	Proportional allocation for each country.<br>Weighting by country to be able to interpret the overall data.
Sampling	Individuals have been sampled in a completely random manner.
Fieldwork period	20 July 2011 to 20 August 2011

Source: Authors' elaboration.

It should be noted that the data analysed in this report relates to an Internet user population which also forms part of online panels. Accordingly, it can be deduced that the respondents' profile in terms ICT uses is slightly more advanced than that of the general population of the surveyed countries. However the underlying dimensions identified and their relationship remain valid.

ICT access

With respect to Internet based activities, the sampled population mainly uses it to search for information (68% every day), sending e-mails with attachments (41%), online banking (20%), social networks (39%) and instant messaging (23%). Internet activities are linked with the male gender, the youngest age groups, a university education, self-employment and entrepreneurs, students, population density and a good state of health.

The factor analysis helped identify the main underlying dimensions of Internet activities. Four factors have emerged:

- Basic uses,
- Individual uses,
- Social – Web 2.0 uses,

- Tech uses.

These factors represent a social gradient of Internet activities from the easiest use of the Internet (basic uses) to the most sophisticates activities (tech uses).

ICT for Health Motivation

Individuals were asked about the **triggers** to utilise ICT for Health. More than a third of the sampled European population indicates a significant use of ICTs in health to better understand a health problem or disease (39%), to find additional sources of information (36%) and to develop knowledge and personal satisfaction (35%). A little further behind, but still with a relevant frequency, there is the perception that ICTs in health are very useful to help a family member or a friend who is ill (31%), to prevent illnesses or to adopt a more healthy lifestyle (28%), to find a solution to or a treatment for a health problem (28%), to obtain different points of view about an issue (22%), and to access an online health service (21%). Finally, and as a counterpoint, only 11% of European citizens give much importance to the use of ICTs in health for participating in online discussions.

With respect to the socio-demographic characteristics of the population, the perception of the importance of ICT in health as triggers is much more positive for women, young people, the middle aged, those with a tertiary education, the employed, students, and people in a bad state of health or with long standing illnesses.

From these items two factors have emerged:

- social and services oriented, and
- individual oriented uses.

Empowerment, broadly understood as the development of personal involvement and responsibility is one of the goals of prevention, promotion and protection in health. This definition assumes that responsibility is a more active form of **control** while **competence** refers to aptitudes or qualities that make it possible to be more autonomous and take a role in decision-making. Factor analysis identified these two dimensions of empowerment Moreover, three different perspectives of personal empowerment seem to coexist with respect to Health:

- ability to comply with expert advice (professional perspective),
- Self-reliance through individual choice (consumer perspective),
- Social inclusion through the development of collective support (community perspective).

Overall, this greater digital empowerment for the European citizens when it comes to their health and the healthcare professionals is linked with higher education levels, the worst states of health and the existence of long-standing illnesses

Finally, individuals were asked about the **barriers to utilise ICT for Health.** Lack of privacy (52%), security (51%), reliability (47%) and trust (46%) were the four main barriers for ICT uses for health indicated by the sampled European population to be very important. Other justifications were the lack of liability (38%), health literacy (36%), knowledge (33%), access to ICTs for health (29%), motivation and interest (28%), and the lack of digital skills (24 %).

Firstly, women are much more sensitive to barriers to the ICT use for health than men, particularly in terms of a lack of confidence. Similarly, the perception of barriers to ICT use for health is also much more evident in older people, those with lower levels of education and the inactive. Lastly, it is also worth highlighting that the presence of long standing illnesses is also very sensitive to lack of confidence.

The underlying dimensions of these items are:

- Lack of confidence, and
- Lack of readiness.

ICT for Health usage

When it comes to specifically using the Internet for health and wellness, the research has provided interesting information, with notable relative differences. The main use of the Internet for health is for individual information searches, rather than sharing information, communicating or interacting about health and more particularly information searches about physical illnesses or conditions.

Over half of the sampled European citizens have never used the Internet to buy medicine or vitamins online (56% of the total); participated in online support groups for people with the same health issue (60%), used social networking sites for health and wellness issues (58%); used e-mail or websites to communicate with a doctor or their office (58%); analysed the privacy policy for personal information in medical websites (52%); explained a medical issue online in order to make contact with an e-health medical service (61%) or with other users (58%); disclosed medical information on social networking sites (67%); or disclosed medical information on websites to share pictures, videos, or movies (67%).

The specific use of ICTs in the health sector is still quite limited among the sampled European citizens. Around three-quarters of the sampled population have never experienced any of the specified ICTs for health uses: 79% of individuals have never made an online consultation through videoconference with healthcare professionals. 75% have not received medical or clinical tests online either. 77% have not accessed or uploaded medical results via a specialist provider, such as Google Health or Microsoft Vault. 76% have not accessed or uploaded medical results via an Internet application provided by a health organisation. 76.6% have not used health or wellness applications on mobile telephones either. And 73.6% of the sampled population has not used ICT applications to transmit vital signs and other clinical information anytime or anywhere.

With respect to the remaining socio-demographic factors, the analysis shows homogeneity in terms of the overall use of ICT for health, which is more frequent in the young population, those with a tertiary education, students and the employed, those in densely populated urban areas, people in a bad state of health and those with long standing illnesses.

The factor analysis of ICT for health activities reveals two underlying dimensions:

- ICT for Health oriented towards Information and Communication, and
- ICT for Health oriented towards services and devices.

Finally, these items allow us to analyse individuals' level of awareness, skills and willingness. First of all, individuals were directly asked about their level of awareness. Second, the number of activities carried out by individuals was considered as a proxy for skilled individuals. Third, individuals who stated they never carry out these activities or were not aware of them were asked about their willingness to carry out these activities. The factor analysis of willingness reveals three underlying dimensions:

- Willingness to use Internet Health information,
- Willingness to use Web 2.0,
- Willingness to use services and devices.

These factors are consistent with the underlying dimensions of ICT readiness mentioned before.

ICT for Health Impact

The study has also provided evidence about the consequences of ICT for Health utilisation. It has to be said that the perceptions are positive overall. 58% of the sampled European population state they agree that ICT use for health allows savings in terms of cost of travel and time. 56% state that they would be willing to share personal health information with their doctor despite the privacy issue. 55% state that ICTs for health can improve the possibilities for caring for themselves and monitoring their state of health. 55% agree with the fact that ICT use for health leads to greater patient satisfaction. 54% agree that e-health can improve the quality of the medical services

received. 50% of the European citizens consider that ICT use for health can change their behaviour towards a healthy lifestyle.

Slightly under half of the sample of European citizens, 43%, agrees that ICT use for health can improve their state of health. 42% consider that they would feel more comfortable and safe if they used a remote monitoring system for their health condition. 42% consider that ICT use for health increases ICT use in other fields of daily life. 32% agree that the use of health services through the Internet substitutes face-to-face consultations with doctors. 32% agree that online health services and face-to-face services are of equal quality. And lastly, 23% of European citizens would be willing to pay for access to Internet health services to improve their state of health or that of their relatives.

Positive attitudes about the impact of ICT for health are more prominent among the youngest population, those with a tertiary education, and those that live in densely populated areas. The only notable difference between individuals with bad state of health and those with good state of health is the perception by the former that ICT uses for health can improve the quality of health services received (57%). Meanwhile, citizens with long standing illnesses clearly state their favourable perceptions of ICT use for health, as opposed to citizens that do not have long standing illnesses. In particular, they state that ICT use can improve patient satisfaction (56%), improve caring and health condition monitoring skills (57%), save travelling costs and time (60%), and that they are willing to share personal information through the Internet with doctors and health organisations despite privacy issues (60%).

Finally the factor analysis reveals two underlying dimensions:

- Impact on quality of healthcare and healthy behaviour,
- Impact on healthcare access.

Social determinants of ICT for Health: key dimensions

All items gathered were grouped into underlying dimensions through multivariate statistics following our conceptual framework. This exercise allows us to transform items into concepts and therefore understand the complexity of the ICT for Health ecosystem.

Underlying dimensions of Social determinants of ICT for Health

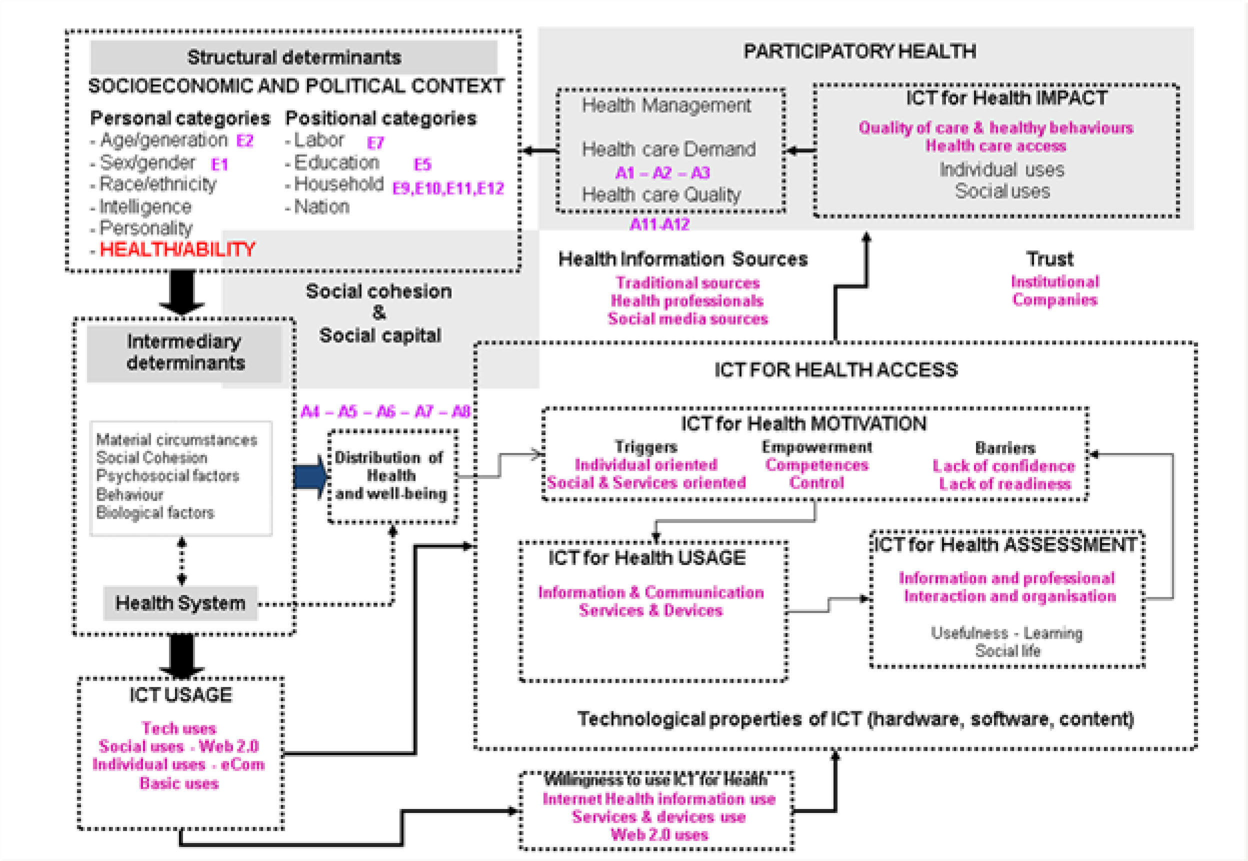

Source: Authors' elaboration.

All above mentioned unveiled the complexity of ICT for Health. To tackle this complexity, correlation analyses of all dimensions have been performed. The main results of these analyses are summarised in the following figure:

Key relationships of Social determinants of ICT for Health

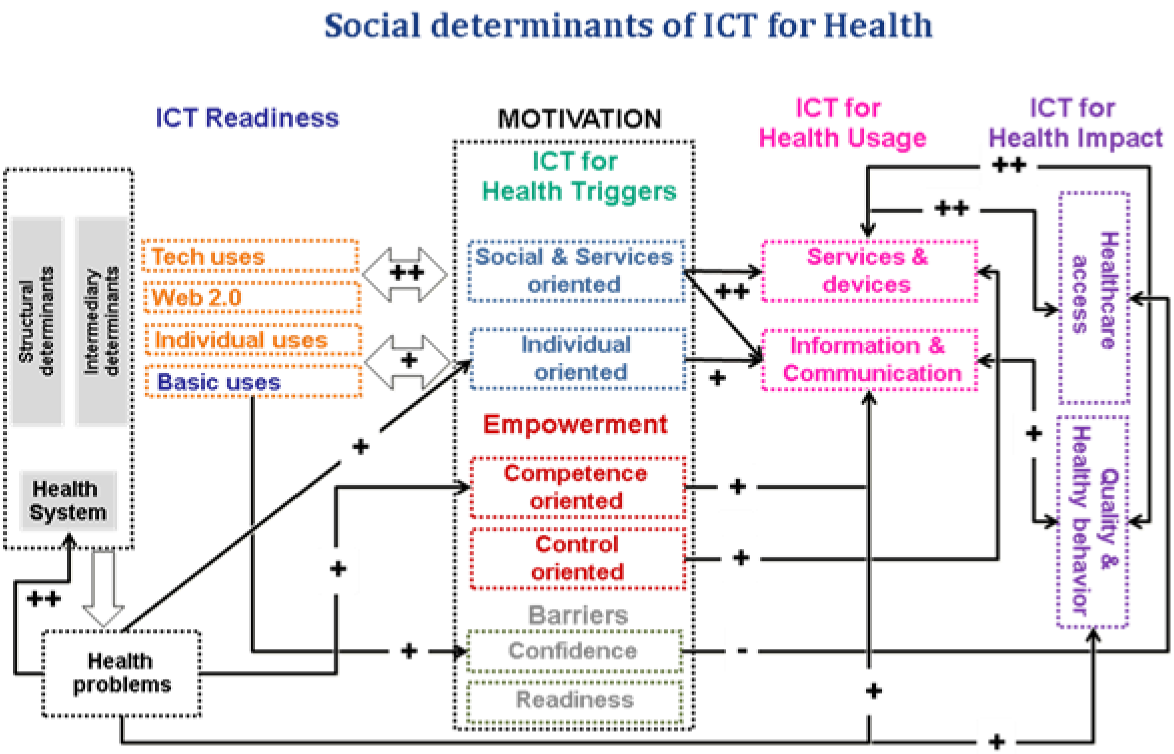

Source: Authors' elaboration.

- Social determinants of Health (structural and intermediary), especially education and age, produces different levels of ICT readiness. Advance uses of the Internet such as Tech and Web 2.0 uses are more likely to be carried out by the young, the healthy and the well-educated population while basic uses are mostly performed by the elderly, therefore individuals with worse health status (chronic patients and individuals having reported higher numbers of health problems).
- Unequal ICT readiness generates different levels of motivation. Individuals making more advance uses are triggered by the potential of ICT to facilitate social interaction and services related to health while individuals whose uses are basic or individual are triggered mainly by Internet health information for personal proposes. Furthermore, individuals with the lowest level of readiness (basic uses) and having reported more health problems lack confidence in the use of ICT for Health. Nevertheless, this lack of confidence is counterbalanced by a higher level of empowerment (competence oriented).
- Both ICT for Health usages (Services and Devices and Information and Communication) are specially driven by social and services triggers while individual triggers are only slightly correlated with Information and Communication usages, therefore less advanced uses.
- Both dimensions of Empowerment push ICT for Health usage. Individuals who are more competence-oriented are more inclined to Information and Communication usage while individuals who are more control-oriented are more likely to use Services and Devices. Thus individuals who feel more responsible for their health status are more likely to use Services and Devices while individuals who want to be more autonomous (competence refers to aptitudes or qualities that make it possible to be more autonomous) are more likely to utilise Information and Communication. If we consider individuals' education, age and health status it looks like Services and Devices are related with well-being and wellness practice, therefore with health prevention and promotion while Information and Communication are more related with illness, therefore with cure and independent living

- All individuals using ICT for Health faced the same barriers; therefore lack of confidence and lack of readiness are not correlated significantly with ICT for Health usages. Nevertheless, lack of confidence is negatively correlated with the ICT for Health impact on the access dimension. Individuals need a certain level of confidence in ICT for Health to go beyond information and communication and engage with services such as RMT, Personal Health Records or videoconference consultation.
- The utilisation of Services and devices is strongly correlated with the perception that ICT would have an impact on both healthcare access and quality and healthy behaviours while the utilisation of Information and Communication is slightly correlated with Quality and healthy behaviours only.
- The number of health problems reported by individuals is only slightly correlated with Information and Communication Usage and it is unrelated to Services and devices utilisation. Therefore, individuals who could take more advantage of Services and devices, due to their health status, are more likely to be oriented towards information and communication usage only.

Lessons learned

The study reported here reveals the potential of ICT for Health **to promote active and healthy individuals** and increase empowerment. Even though our findings relate to Internet users, it is worth pointing out that new health inequalities are emerging due to the impact of the "traditional determinants of heath" on ICT readiness.

Therefore, eInclusion policies related to ICT for Health are needed to ensure that individuals with low socio-economic status and more health problems are able to benefit from these types of technologies. These ICT for Health divides specially impact on the elderly. However, there is an opportunity for them to engage with the Information Society through ICT for Health due to the importance of health issues in their daily life.

The relationship between the different typologies of ICT readiness and ICT for Health Motivation and Impact reveal that:

- Young individuals are already using this type of technologies mostly in relation with wellness and healthy life style. These uses enable an entire world of possibilities related with **health promotion and prevention**, especially considering that young individuals are heavy Web 2.0 users.
- Middle age individuals are also active users of ICT for Health acting as **gatekeepers of this type of technologies within the household**. Therefore these individuals could act as enablers for others i.e. both for the elderly and the young within households.
- The elderly are basically using ICT for Health for information and communication purposes. There is a gap between this type of use and services and devices uses which could be more effective in relation with **cure and chronic conditions**.

Individuals between 16-54 with chronic conditions, going under long-term treatment and with more than one health problems are more likely to use ICT for Health than individuals without these types of health problems. Individuals between 55-74 who are healthy are more likely to use ICT for Health, especially for Information and Communication, than individuals with worse health status. Therefore, in the short term, this group of individuals will be pushing for health systems to provide them with new solutions (services and devices) when they need to tackle a health problem. This pressure will increase during the next decade when middle age individuals become elderly. Therefore health systems are facing the **challenge of having to promote further ICT innovation to answer these new demands**. While this is an opportunity to improve both sustainability and efficiency of healthcare system, it is associated with a number of challenges linked to eHealth deployment.

Further, during this transition, health systems cannot leave out the elderly who are not active and healthy: this group of individuals cannot be omitted as they are the current intensive users of healthcare systems. There is an **opportunity to include** them in the Information Society by **improving ICT readiness and ICT for Health willingness and awareness**.

1 INTRODUCTION

1.1 Background and rationale of SIMPHS2

SIMPHS 2, the Strategic Intelligence Mapping on Personal Health Systems phase 2 (SIMPHS2), is a project carried out by the IPTS in cooperation with DG INFSO. Taking a demand side approach, SIMPHS 2 aims to further expand the fact findings from SIMPHS 1.

The conclusions drawn upon completion of SIMPHS phase 1 in May 2010 identified the following set of areas that deserve further research and analysis:

- to enlarge the scope of our focus from PHS to IPHS (Integrated Personal Health/Care Services) as a result of emerging trends of convergence between health and social care also in the provision of ICT enabled services
- to adopt a demand driven research design as opposed to the supply-driven one that characterised SIMPHS Phase 1;
- to include a fact finding component, beyond RMT, focusing on Telecare (and its more sophisticated versions such as Ambient Assisted Living AAL, or Independent Living, IL), Mobile Health, and Wellness; and
- to produce empirical and prospective analysis of potential impacts which can support the Impact Assessment for relevant INFSO policy activities (such as European Large Scale Actions (ELSAs) or European Research and Innovation Partnerships (ERIPs)), and also with the purpose of raising awareness and creating consensus among the different stakeholders through the sharing of the knowledge base.

In light of the above, DG INSFO/H1 requested JRC-IPTS to expand the scope of the research developed during SIMPHS Phase 1 to new areas of interest (Telecare, Mobile Health, and Wellness) and study the integration between disease management and RMT as well as health and social care in order to extract strategic intelligence and quantitative evidence to support the policy process. SIMPHS 2 investigates the use of Personal Health Systems (PHS), starting with the Remote Patient Monitoring and Treatment (RMT) segment for chronic disease management. The specific diseases of SIMPHS 2 focus are diabetes, Cardio Vascular Disease (CVD) and Chronic Obstructive Pulmonary Diseases (COPD). Expected results aim at supporting policy making by providing evidence on the current development and use of RMT from the perspective of the demand side (policy makers, hospitals, health care professionals and end-users) identifying drivers and barriers to its large-scale take up in Europe using three axes: diffusion of innovation, governance and health impact assessment. Thus, impact on quality of life and treatment costs will be at the core of the study. In addition, it will also look at current reimbursement systems for RMT and coordination between health and social care services for the use of these applications.

Within this background to gain more insights from the perspective of the demand supply an online panel survey to Internet users has been carrying out on 14 EU countries about Health and ICT.

1.2 Policy context

1.2.1 From eEurope to Digital Agenda for Europe

The European Commission eHealth Action Plan defines eHealth as "the application of information and communications technologies across the whole range of functions that affect the health sector' and including 'products, systems and services that go beyond simply Internet-based applications" [1]. This definition has been expanded by the eHealth task force in support of the Lead Market Initiative [2] to encompass four categories of applications:

1. Clinical information systems (specialized tools for health professionals within care institutions, tools for primary care and/or for outside the care institutions);
2. Telemedicine and homecare systems and services;

3. Integrated regional/national health information networks and distributed electronic health record systems and associated services;
4. Secondary usage non-clinical systems (systems for health education and health promotion of patients/citizens; specialised systems for researchers and public health data collection and analysis; support systems for clinical processes not used directly by patients or health care professionals.

eHealth has figured high in the European Commission Information Society policy agenda for a decade: starting with the eEurope framework,[2] continuing into *i2010 strategy* [6], and today as part of Pillar 7 (ICT for Societal Challenges) the new *Digital Agenda for Europe* (DAE) for the period 2010-2015 [7:29-30]. Actually, Commission support to what we call eHealth today (and earlier went under different names such as health telematics) predates its systematisation into general information society policy as it started in the early 1990s through co-funded research in the framework programmes and has continued since 2007 both through FP7 and through the Competitiveness and Innovation Programme (CIP) deployment instruments. eHealth in 2007 was part of the *Lead Market Initiatives* and as of 2011 is one of the first DAE Flagship initiatives with the European Innovation Partnership on Active and Healthy Ageing. It must also be stressed that the healthcare challenges and the potential of innovation through ICT to tackle them are expressly grounded in the 'smart pillar' of the overall *EU2020 Strategy* [8:10].

Stated in very compact form the objective pursued by eHealth policy is to '*improve the quality of care and reduce medical costs*' [7:29]. This objective summarises the various promises of eHealth that have been heralded for more than a decade (and very effectively reviewed in Lapointe [9]), which include amongst others:

- Reducing medical errors, drugs adverse events and associated costs (i.e. through adverse events computerised reporting systems, ePrescription of diagnostic procedures, electronic health records, etc);
- Improving adherence to prescriptions (through reminders and telemonitoring);
- Reducing in-patient costs while improving health outcomes (telemonitoring);
- Supporting and improving the work of professionals in various ways (Picture Archiving and Communication Systems, tele-radiology, Computerised Physicians Order Entry, online transmission of clinical tests results);
- Streamlining and making the administration of hospitals more efficient (Integrated computerised systems for billing, order entry, discharging, etc.);
- Increasing access and convenience for users (eBooking, access to their electronic health records, portability of their information across the system, etc.).

1.2.2 Healthcare and ageing in the new policy context toward 2020

Toward the end of 2009 the first report of the European Research Area Board (ERAB) placed ageing and healthcare among the grand challenges on the road toward Europe's Renaissance.[3] Ageing and health figure prominently in the new EU2020 Strategy,[4] and the implications from the perspective

[2] This framework, whose open volley was the 1999 joint European Council and Commission initiative [3], saw first in 2000 the launch of eEurope 2002 [4] and then in 2002 that of eEurope 2005[5].

[3] European Commission, *Preparing Europe for a New Renaissance: A Strategic View of the European Research Area*, First Report of the European Research Area Board, Brussels, European Commission, DG Research, EUR 23905, 2009, p. 7.

[4] European Commission, Europe 2020. A European Strategy for smart, sustainable and inclusive growth, Brussels. COM (2010) 2020, 2010.

of ICT (i.e. eHealth) are clearly underlined in the Spanish Presidency Granada declaration[5] and the new European Digital Agenda.[6]

EU2020 includes as sources of structural weaknesses in Europe,[7] the acceleration of demographic ageing and the low workforce participation of older workers and considers ageing among the long-term global challenges that the European social market model is facing.[8] In the 'smart pillar' of the strategy, ageing is among the objectives of the flagship initiative "Innovation Union"[9] (i.e. 'technologies to allow older people to live independently and be active in society' will be one of the first "European Innovation Partnerships" to be funded), whereas within the 'inclusive growth pillar' an important reference is made to the need for reducing health inequalities and for promoting active and healthy ageing, thus, contributing to social cohesion and higher productivity.[10] Last but not least, EU2020 stresses the strategic importance of leveraging the full potential of ICT in pursuing smart, sustainable and inclusive growth.[11] In sum, the new EU strategy provides full policy support, and actually calls for, the kind of two-fold approach that ICT can enable in the domain of health and social care: coping with societal challenges while creating new inclusive market opportunities.

Such an approach is further reinforced in the new Digital Agenda for Europe (see footnote 6). The Digital Agenda stresses how "by harnessing the full potential of ICT, Europe could much better address some of its most acute societal challenges: climate change and other pressures on our environment, an ageing population and rising health costs".[12] The Digital Agenda devotes also an entire paragraph to "Sustainable healthcare and ICT-based support for dignified and independent living",[13] where it underlines how the action in this area will contribute to the earlier mentioned European Innovation Partnership foreseen by EU2020 and also stresses that previously launched policy actions such as the Lead Market Initiative[14] will play a key role in further catalysing the deployment of eHealth with an explicit mention of those services and applications addressing the needs of chronic patients (telemedicine, Telemonitoring, mobile health) and of the elderly (Independent Living and Ambient Assisted Living).

As such, the contents devoted by the Digital Agenda to eHealth fully support the two-fold view of the potential of ICT in health and social care which is to both cope with societal challenges and create opportunities for innovation and economic growth. Hence, the new policy context confirms and reinforces the support to ICT enabled innovation in the domain of health and social care that were already present in the previous policy antecedents, such as the eHealth Action Plan, the Lead Market Initiative, the Ambient Assisted Leaving Joint Programme and various other communications, studies and research projects. Considering that one of the key pillar of the new Digital Agenda is the deployment and adoption of Next Generation Access [10] networks throughout Europe, ICT enabled health and social care services can be among the added-value 'contents' to be conveyed through these new fast and very fast "pipes" valorising the investments in infrastructure. As illustrated in the outer part of Figure 1 below, a virtuous cycle of the digital economy could be unleashed between increase demand for digital services, roll out of NGA networks, and creation of content and borderless services. Personalised digital health and care services could very well be among the key contributor to such a cycle. Mobile health, for instance, is one of the potential sources of spill over

---

5 We refer here to the contents of the Granada Ministerial Declaration, adopted by the Council on April 19, 2010. See also Spanish Presidency of the EU, Spanish Proposal for a Digital Europe: The Granada Strategy, February 24th 2010 (http://www.eu2010.es/es/documentosynoticias/noticias/abr19telec.html).

6 European Commission, *A Digital Agenda for Europe*, Brussels. COM (2010)245, 2010.

7 European Commission, *Europe 2020, op. cit.*, p. 5.

8 European Commission, *Europe 2020, op. cit.*, p. 6.

9 European Commission, *Europe 2020, op. cit.*, p. 10.

10 European Commission, *Europe 2020, op. cit.*, p. 16.

11 European Commission, *Europe 2020, op. cit.*, pp. 9-10.

12 A Digital Agenda for Europe, op. cit., p. 6.

13 *Ibid.*, pp. 29-30.

14 http://ec.europa.eu/enterprise/leadmarket/doc/com_07_en.pdf

benefits from faster and very fast networks that can justify the public investments needed to build this new infrastructure.[15] Yet, the inner part of the figure also highlight the vicious cycle that has blocked so far the realisation of the full potential for a European digital economy and society.

Figure 1: The Virtuous Cycle of the digital economy

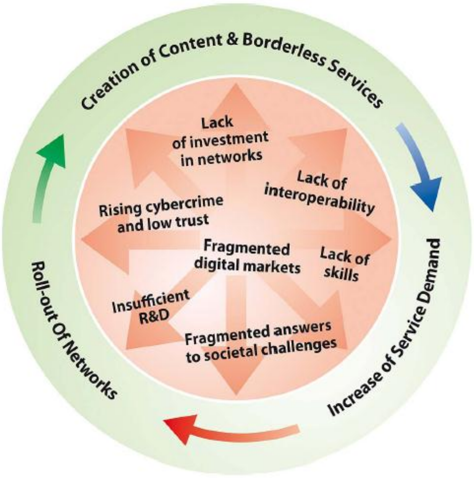

Source: A Digital Agenda for Europe, p. 4.

The main eHealth related target of the DAE (and the corresponding actions described in the scoreboard are the following (the first two are split into separate targets):

<u>Action 75a: Give Europeans secure online access to their medical health data</u>[16]

Objectives: increase empowerment and quality of life for citizens while contributing to healthcare system sustainability, contribute to EIPAHA

Target: undertake pilot actions to equip Europeans with secure online access to their medical health data by 2015

<u>Action 75a: achieve widespread telemedicine deployment</u>[17]

Objectives: increase empowerment and quality of life for citizens while contributing to healthcare system sustainability, contribute to EIPAHA

Target: achieve by 2020 widespread deployment of telemedicine services

---

[15] As clearly described in the EFII White paper, the European Future Internet PPP will seek commonality across application sectors to facilitate achieving critical mass in the creation of new European-scale markets for smart infrastructures, with integrated advanced, secure and trusted communications functionalities. One such sector is eHealth and Ambient Assisted Living (AAL) where developing and demonstrating large scale infrastructure of eHealth and mHealth services, by connecting the whole set of involved actors in the healthcare provision chain, is needed. The main challenges of the EFII PPP related to eHealth is to specify, design, prototype and test an eHealth Service Platform that will give doctors patients and applications unified, standard access to medical information and support service features such as tele-rehabilitation, vital signs monitoring (automatic monitoring with established thresholds that trigger alarms), alerts, tele-presence of health care professionals, remote medical administration monitoring, medication reminders, appointment reminders, location tracking, context information processing, etc. (see http://www.future-internet.eu/fileadmin/initiative_documents/Publications/White_Paper/EFII_White_Paper_2010_Public.pdf).

[16] http://ec.europa.eu/information_society/newsroom/cf/fiche-dae.cfm?action_id=233&pillar_id=49&action=Action%2075%3A%20Give%20Europeans%20secure%20online%20access%20to%20their%20medical%20health%20data

[17] Ibid.

Action 76: Propose a recommendation to define a minimum common set of patient data[18]

Objectives: establish minimum set of criteria to achieve inter-operability of patient records for cross-border access and/or exchange. Contribute to action 77

Target: to be achieved by 2012.

Action 77: Foster EU-wide standards, interoperability testing and certification of eHealth[19]

Objectives: unleash an EU eHealth market by overcoming local and market fragmentation;

Target: achieve the above by 2015 through stakeholder dialogue.

1.2.3 European Innovation Partnership on Active and Healthy Ageing

On 7 November 2011 the Steering Group of the pilot European Innovation Partnership on Active and Healthy Aging agreed on joint actions in response to the societal challenge of an ageing population.[20] The overarching objective is to ensure that the average European citizen has two more active and healthy years to live by 2020, focusing on the three main areas of life events:

- Prevention,
- Care and cure,
- Independent living.

and on five specific actions:

- Innovative ways to ensure patients follow their prescriptions – a concerted action in at least 30 European regions;
- Innovative solutions to prevent falls and support early diagnosis for older people;
- Co-operation to help prevent functional decline and frailty, with a particular focus on malnutrition;
- Spread and promote successful innovative integrated care models for chronic diseases amongst older patients, such as through remote monitoring. Action should be taken in a number of the EU's regions;
- Improve the uptake of interoperable ICT independent living solutions through global standards to help older people stay independent, mobile and active for longer.

Furthermore, the expected results would be threefold:

- An improvement of the health status and quality of life of Europeans, especially older people;
- An improvement of the sustainability and efficiency of health and social care systems;
- Boosted EU competitiveness through an improved business environment for innovation.

1.3 Conceptual framework: towards a social determinants of ICT for Health

The roots of a social approach to health are grounded in the recognition that social and environmental factors decisively influence people's health. This approach is ancient and has received the support from WHO since 1950.[21] The definition of Social Determinants of Health (SDH) encompasses the full set of social conditions in which people live and work; however, within the

---

[18] http://ec.europa.eu/information_society/newsroom/cf/fiche-dae.cfm?action_id=234&pillar_id=49&action=Action%2076%3A%20Propose%20a%20recommendation%20to%20define%20a%20minimum%20common%20set%20of%20patient%20data

[19] Ibid.

[20] European Innovation Partnership agrees on actions to turn ageing into an opportunity http://europa.eu/rapid/pressReleasesAction.do?reference=IP/11/1309&format=HTML&aged=0&language=EN&guiLanguage=en

[21] Irwin A, Scali E. Action on the Social Determinants of Health: learning from previous experiences. Social Determinants of Health Discussion Paper 1 (Debates).

field encompassed by this concept, not all factors have equal importance. Bringing different theoretical traditions[22] the Commission on Social Determinants of Health has summarised in Figure 2 how *"social, economic and political mechanisms give rise to a set of socioeconomic positions, whereby populations are stratified according to income, education, occupation, gender, race/ethnicity and other factors; these socioeconomic positions in turn shape specific determinants of health status (intermediary determinants) reflective of people's place within social hierarchies; based on their respective social status, individuals experience differences in exposure and vulnerability to health-compromising conditions"*.

Figure 2: Final form of the Commission on Social Determinants of Health conceptual framework

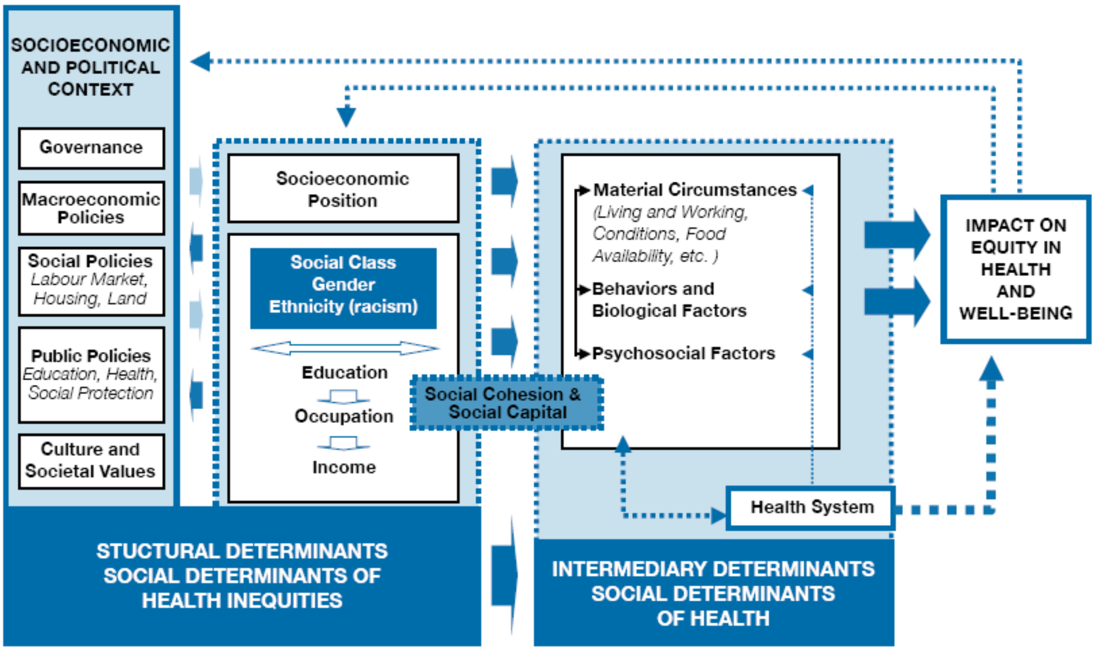

Source: WHO 2010 [11] (p.6).

Socioeconomic and political context is broadly defined to include all social and political mechanisms that generate, configure and maintains social hierarchies, including: the labour market, the educational system, political institutions and other cultural and societal values.

Context, structural mechanisms and the resulting socio-economic position of individuals (the most important structural stratifiers and their proxy indicators include Income, Education, Occupation, Social Class, Gender, Race/ethnicity) taken together make up "structural determinants" and in effect it is these determinants we refer to as the "social determinants of health inequities."

The underlying social determinants of health inequities operate through a set of intermediary determinants of health to shape health outcomes. The main categories of intermediary determinants of health are: material circumstances; psychosocial circumstances; behavioural and/or biological factors; and the health system itself as a social determinant.

The role of the health system becomes particularly relevant through the issue of access, which incorporates differences in exposure and vulnerability, and through intersectoral action led from within the health sector. The health system plays an important role in mediating the differential consequences of illness on people's lives.

This framework does not relate directly to ICT for Health, nevertheless the structural determinants perfectly overlap the core argument of personal and positional categories of and distribution of

[22] Solar O, Irwin A. A conceptual framework for action on the social determinants of health. Social Determinants of Health Discussion Paper 2 (Policy and Practice).

resources in van Dijk's "Causal and Sequential Model of Digital Technology Access by individuals in Contemporary Societies" (Figure 3).

Figure 3: A Causal and Sequential Model of Digital Technology Access by Individuals in Contemporary Societies

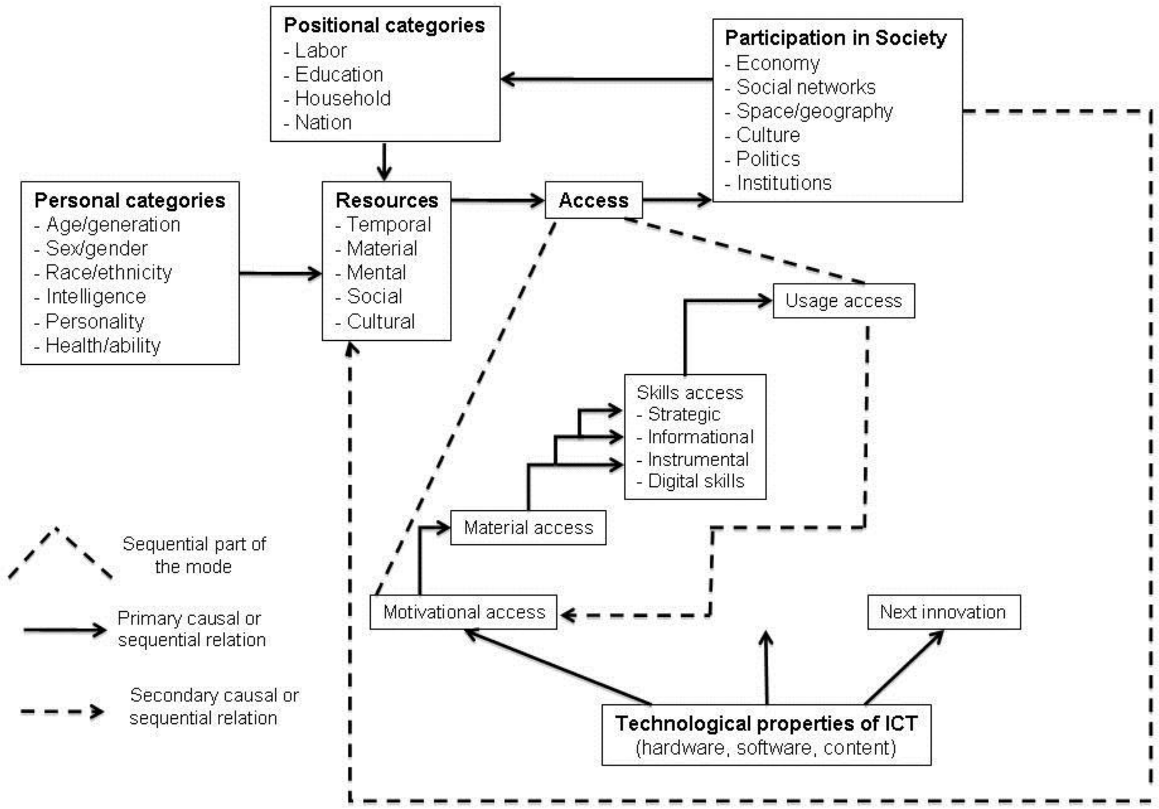

Source: van Dijk 2005 [12] p.24.

This framework has been summarised by van Dijk as follow:

- Categorical inequalities in society produce an unequal distribution of resources.
- An unequal distribution of resources causes unequal access to digital technologies.
- Unequal access to digital technologies also depends on the characteristics of theses technologies.
- Unequal access to digital technologies brings about unequal participation in society.
- Unequal participation in society reinforces categorical inequalities and unequal distribution of resources.

However the term access goes beyond broadband connectivity and refers to four stages:

- Motivation access (motivation to use digital technologies),
- Material or physical access (possession of computers and Internet connections or permission to use them and their contents),
- Skills access (possession of digital skills: operational, informational and strategic),
- Usage access (number and diversity of applications, usage time).

These two frameworks summarised in Figure 2 and Figure 3 are the roots of our conceptual framework **Towards social determinants of ICT for Health** (Figure 4):

- Social determinants of health and health inequalities, therefore structural and intermediary determinants produce different levels of ICT access (motivation, material, skills and usage).
- Unequal access to ICT will generate different levels of ICT for Health access as well as different levels of willingness to use ICT for Health.
- ICT for Health access depends on the properties of ICT and the relationship among Motivation; ICT for Health readiness and Internet Health information.
    - Motivation includes Triggers, Empowerment and Barriers,
    - ICT for Health readiness includes Awareness, Material access; Skills and Usage,
    - Internet Health information includes how individuals use and evaluate this type of information for themselves or for others (social life of information) as well as their perception about usefulness and learning.
- ICT for Health Access gives rise to different level of Participatory Health through the utilisation of health information (individual and social uses) and behavioural changes due to the ICT for Health impact on:
    - Health management,
    - Health care demand,
    - Health care quality.
- These impacts could modify both structural and intermediary determinants and distribution of health and well-being.

Figure 4: Social Determinants of Health and ICT for Health conceptual framework

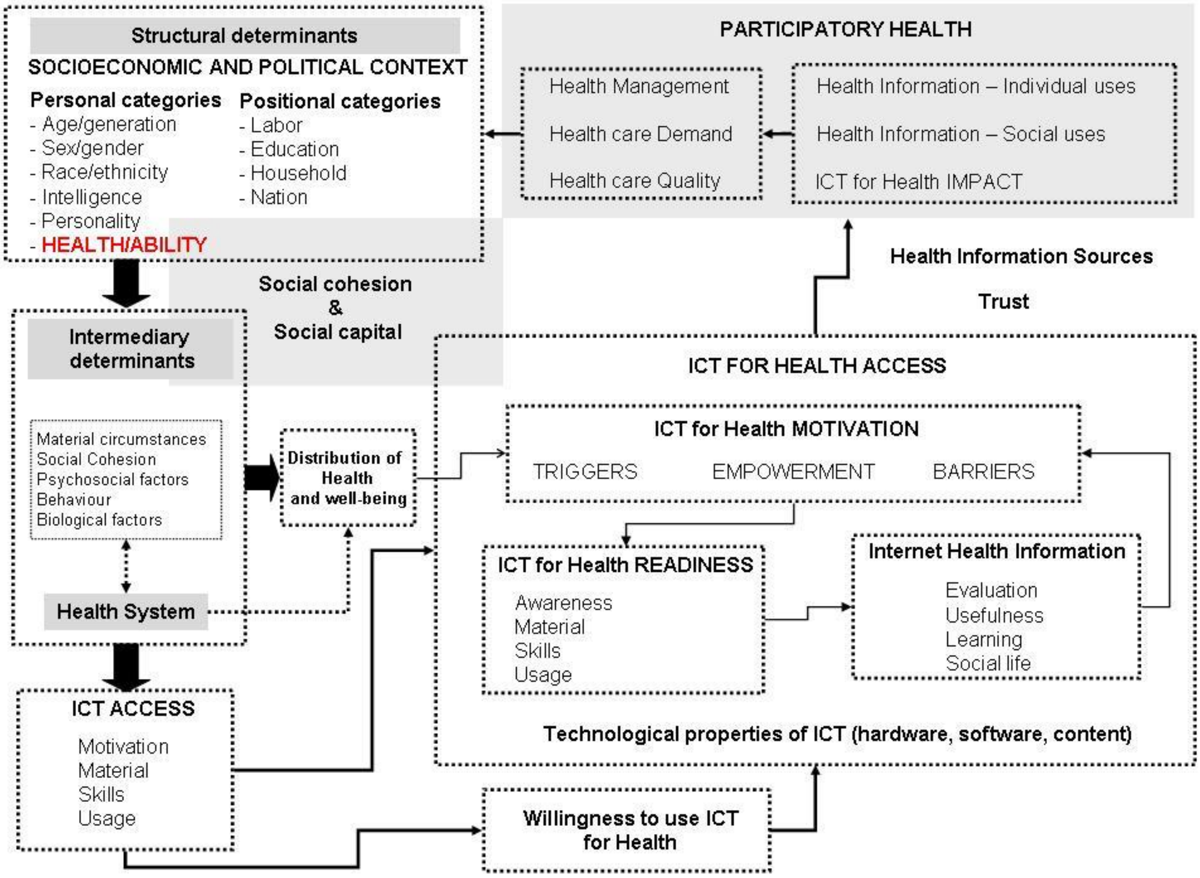

Source: Based on WHO [11] and van Dijk [12].

1.4 Outline of the report

This report is structured as follows:

- Chapter 1 provides a brief observation of the political context and the analytical framework around the main issue tackled by this study.
- Chapter 2 contains a description of the design methodology on which the research is based. This includes information about the scope of the population being researched, the sampling strategy and the sample used as well as the description of the survey design and field work process.
- Chapter 3 refers to the socio-demographic description of the population being researched. Beyond the sample quotas of gender and age, which are pre-defined, we obtained information about the characteristics of the individuals such as their level of education, employment situation or, type of household. Furthermore, a socio-demographics comparison between our sample and European population was carried out.
- Chapter 4 mainly refers to the general state of health of the European population surveyed and how they use health and social care services.
- Chapter 5 tackles Internet access, frequency of use and general activities carried out by individuals.
- Chapter 6 contains individuals' utilization of health information sources and perception of trust.
- Chapter 7 focuses on individuals' motivations to use ICT for Health (triggers and empowerment) as well as the barriers perceived.
- Chapter 8 refers with ICT for Health access, utilization, awareness and willingness to use these technologies in relation with Health.
- Chapter 9 provides insights on Internet health information and factors to evaluate Internet sites.
- Chapter 10 tackles individuals' perception of ICT for Health impact and behavioural change. Furthermore, this chapter analyses how individuals evaluate ICT for Health sites.
- Chapter 11 presents the results of the multivariate analysis carried out and how we move from questionnaire items to conceptual dimensions of the conceptual framework.
- Chapter 12 concludes with some lessons learned and policy recommendations.

2. METHODOLOGY

2.1 Questionnaire design

To reach our target population, we have used the Internet as a methodological tool. As argued elsewhere survey research[13] is becoming a frequently used methodology due to the advancement of computer hardware, software and increasing access to the Internet. Furthermore, online surveys offer a valid alternative to the postal, telephone or face-to-face surveys as long as technical, methodological, ethical and legal considerations are taken into account.[14, 15, 16, 17].

The questionnaire was designed considering our framework in Figure 4 as well as the policy context

The full questionnaire and the coding manual are available in *Annex 1. Questionnaire and coding manual* while *Annex 2. Online panel provider* describes the companies which carried out the fieldwork.

The questionnaire was structured in 5 blocks:

- Block A: Health status and health care and social care services use,
- Block B: Health attitude and Health information sources,
- Block C: Internet and Information and Communication Technologies uses,
- Block D: Health related use of Information and Communication Technologies and the Internet,
- Block E: Socio demographic profile of participants.

2.2 Survey design and sampling

It is appropriate at this point to explain the methodological design of the research. To obtain the objectives therefore, an ad-hoc research study has been designed to collect first hand information. Table 1 resumes the technical information about the study.

Table 1: Technical information

Population	Citizens aged from 16 to 74 years old who have used the Internet in the last three months.
Scope of countries	Austria, Belgium, Germany, Denmark, Estonia, Finland, France, Italy, Netherlands, Sweden, Slovenia, Slovakia, Spain, United Kingdom
Type of survey	Online
Sample size	1,000 interviews per country.<br>14,000 interviews in total.
Quotas	Country<br>Gender (Female/Male)<br>Age Group (16-24/ 25-54/ 55-74)
Sampling error	±0.85% for overall data and ±3.16% for country-specific data. In all cases, a maximum indeterminate probability (p=q=50), for a confidence level of 95.5% is applicable for each one of the reference populations
Weighting	Proportional allocation for each country.<br>Weighting by country to be able to interpret the overall data.
Sampling	Individuals have been sampled in a completely random manner.

Source: Authors' elaboration.

The demographic groups are organised by the cross-referenced quotas of gender and age group, as follows:

- Women aged between 16 and 24 years old,
- Women aged between 25 and 54 years old,
- Women aged between 55 and 74 years old,
- Men aged between 16 and 24 years old,
- Men aged between 25 and 54 years old,
- Men aged between 55 and 74 years old.

Table 2: Target population, by country, gender and age

TARGET POPULATION	FEMALE			MALE			TOTAL
	16-24	25-54	55-74	16-24	25-54	55-74	
Austria (AT)	483,635	1,500,590	297,082	489,822	1,612,517	428,987	4,812,634
Belgium (BE)	617,834	1,873,936	500,564	654,433	1,991,401	651,916	6,290,084
Germany (DE)	4,439,469	15,355,444	4,518,734	4,607,687	16,265,798	5,720,867	50,907,999
Denmark (DK)	325,791	1,059,924	425,239	339,448	1,058,849	444,122	3,653,373
Estonia (EE)	91,240	255,389	65,405	93,244	224,916	39,519	769,713
Finland (FI)	320,602	1,003,259	404,913	334,552	1,021,691	396,595	3,481,612
France (FR)	3,949,487	11,593,680	3,317,140	3,983,537	10,815,483	3,787,759	37,447,086
Italy (IT)	2,405,553	7,384,182	1,076,659	2,558,069	8,477,798	1,938,877	23,841,139
Netherlands (NL)	978,150	3,329,750	1,182,191	1,031,238	3,408,239	1,387,366	11,316,934
Sweden SE)	606,470	1,765,839	756,070	632,416	1,799,699	803,811	6,364,306
Slovenia (SI)	112,182	350,394	58,017	119,649	386,012	67,563	1,093,816
Slovakia (SK)	372,586	1,043,328	182,542	395,907	1,090,145	191,577	3,276,086
Spain (ES)	2,260,103	7,634,024	933,149	2,312,050	8,242,066	1,290,044	22,671,436
United Kingdom (UK)	3,938,640	11,404,155	3,751,330	4,074,594	11,315,916	3,900,419	38,385,055
TOTAL	20,901,742	65,553,894	13,402,176	21,626,645	67,710,531	21,049,423	210,244,411

Source: Eurostat, ICT Households Survey 2010.

Finally, having defined the object population of the study, the sample is displayed in Table 3. The sample has two essential characteristics:

- Firstly, an equal size sample has been chosen for each one of the countries being studied. This leads to an equal level of reliability in the results obtained in each of the countries.
- Secondly, the choice was made to use a fully representative sample for the distribution of the target population, according to gender and age group, which means that there is no need for any weighting to be applied to interpret the data.

Table 3: Sample by country, gender and age

SAMPLE	FEMALE			MALE			TOTAL
	16-24	25-54	55-74	16-24	25-54	55-74	
Austria (AT)	100	312	62	102	335	89	1,000
Belgium (BE)	98	298	79	104	317	104	1,000
Germany (DE)	87	301	89	91	320	112	1,000
Denmark (DK)	89	290	116	93	290	122	1,000
Estonia (EE)	119	332	85	121	292	51	1,000
Finland (FI)	92	288	116	96	294	114	1,000
France (FR)	105	310	89	106	289	101	1,000
Italy (IT)	101	310	45	107	356	81	1,000
Netherlands (NL)	86	294	105	91	301	123	1,000
Sweden SE)	95	278	119	99	283	126	1,000
Slovenia (SI)	103	320	53	109	353	62	1,000
Slovakia (SK)	114	318	56	121	333	58	1,000
Spain (ES)	100	337	41	102	363	57	1,000
United Kingdom (UK)	103	297	98	106	295	101	1,000
TOTAL	1,392	4,285	1,153	1,448	4,421	1,301	14,000

Source: Authors' elaboration.

Table 4 shows the study sampling errors (overall and by quotas). They are calculated for a probability no greater than 95.5%, and for the least desired context, i.e. a maximum indeterminate probability (p = q = 50%), for the reference population.

The sampling error is the error caused by observing a sample instead of the whole population. The sampling error can be found by subtracting the value of a parameter from the value of a statistic and is calculated with the formula given below:

$$e \sqrt{\frac{(Z^2 \text{ x } p \text{ x } q) \text{ x}}{(N-1)}}$$

Where:

e = Sampling error

Z= Confidence level. The value for selected alpha level of .0225 in each tail = 2. The value of *Z* is set to 2, representing a confidence level of 95.5%. We want the highest accuracy possible, with the smallest sample size. This confidence level gives us the best trade-off between these two goals.

The expected scenario is maximum indetermination (p=q=50) where:

p= the conversion rate we expect (estimate of the true conversion rate in the population)

q= The conversion rate we don't expect

N= Total population (GP's)

n= Proposed sample (GP's)

These sampling errors, in fact, determine the statistical reliability of the sample and, consequently, it is necessary to take them into consideration. The overall error margin, therefore, is ± 0.85%, with a country specific error margin of ±3.16%. These errors are in line with the statistical criteria that validate the sample design and, the sample being representative and reliable, it is possible to extrapolate the study results to the target population group in the selected countries.

Table 4: Sampling error, by country, gender and age

SAMPLE	FEMALE			MALE			TOTAL
	16-24	25-54	55-74	16-24	25-54	55-74	
Austria (AT)	±10.00	±5.66	±12.70	±9.90	±5.46	±10.60	±3.16
Belgium (BE)	±10.10	±5.79	±11.25	±9.81	±5.62	±9.81	±3.16
Germany (DE)	±10.72	±5.76	±10.60	±10.48	±5.59	±9.45	±3.16
Denmark (DK)	±10.60	±5.87	±9.28	±10.37	±5.87	±9.05	±3.16
Estonia (EE)	±9.17	±5.49	±10.85	±9.09	±5.85	±14.00	±3.16
Finland (FI)	±10.43	±5.89	±9.28	±10.21	±5.83	±9.37	±3.16
France (FR)	±9.76	±5.68	±10.60	±9.71	±5.88	±9.95	±3.16
Italy (IT)	±9.95	±5.68	±14.91	±9.67	±5.30	±11.11	±3.16
Netherlands (NL)	±10.78	±5.83	±9.76	±10.48	±5.76	±9.02	±3.16
Sweden SE)	±10.26	±6.00	±9.17	±10.05	±5.94	±8.91	±3.16
Slovenia (SI)	±9.85	±5.59	±13.74	±9.58	±5.32	±12.70	±3.16
Slovakia (SK)	±9.37	±5.61	±13.36	±9.09	±5.48	±13.13	±3.16
Spain (ES)	±10.00	±5.45	±15.62	±9.90	±5.25	±13.25	±3.16
United Kingdom (UK)	±9.85	±5.80	±10.10	±9.71	±5.82	±9.95	±3.16
TOTAL	±2.68	±1.53	±2.95	±2.63	±1.50	±2.77	±0.85

Source: Authors' elaboration.

As has been previously explained, the sample distribution is proportional and representative in each country, according to the proportion of individuals that have used the Internet in the last three months by gender and age group. This means it is not necessary to weight the sample to interpret the country-specific data.

However, as each country's population is clearly different, in spite of being sampled in equal measure, weighting has been applied to ensure a representative sample for interpretation of the overall data, i.e. for all the selected countries.

In this report, we analyse the results on three levels: the average for the 14 Member States, the differences according to the socio-demographic characteristics of the respondents and issues with respect to state of health and the national average. The overall analysis and the socio-demographic and the state of health analyses are based on the 14 Member States, i.e. the average of the results for the 14 Member States. This average is weighted to reflect the actual population of each of the Member States, as was previously explained.

Each country's weighting factor has been calculated by dividing the proportion of the country's population to the total population (210,244,411) by the proportion of individuals in each country's sample (1,000) to the total sample (14,000).

Table 5: Weighting factors by country

COUNTRY	Population	Population (Proportion)	Sample	Sample (proportion)	WEIGHT FACTOR
Austria (AT)	4,812,634	0,022891	1.000	0,07143	0,32047
Belgium (BE)	6,290,084	0,029918	1.000	0,07143	0,41885
Germany (DE)	50,907,999	0,242137	1.000	0,07143	3,38992
Denmark (DK)	3,653,373	0,017377	1.000	0,07143	0,24328
Estonia (EE)	769,713	0,003661	1.000	0,07143	0,05125
Finland (FI)	3,481,612	0,016560	1.000	0,07143	0,23184
France (FR)	37,447,086	0,178112	1.000	0,07143	2,49357
Italy (IT)	23,841,139	0,113397	1.000	0,07143	1,58756
Netherlands (NL)	11,316,934	0,053828	1.000	0,07143	0,75359
Sweden SE)	6,364,306	0,030271	1.000	0,07143	0,42379
Slovenia (SI)	1,093,816	0,005203	1.000	0,07143	0,07284
Slovakia (SK)	3,276,086	0,015582	1.000	0,07143	0,21815
Spain (ES)	22,671,436	0,107834	1.000	0,07143	1,50967
United Kingdom (UK)	38,385,055	0,182573	1.000	0,07143	2,55603
TOTAL	210,244,411	1	14.000	1	

Source: Authors' elaboration.

It is worth specifying at this point that a regional quota has been introduced in Spain to interpret the data for 3 Autonomous Regions in Spain with sufficient sample size. The Autonomous Regions are: Andalusia, Basque Country and Catalonia. Table 6shows the sample from these Autonomous Regions:

Table 6: Sample by region

SAMPLE	FEMALE			MALE			TOTAL
	16-24	25-54	55-74	16-24	25-54	55-74	
Andalusia	22	68	3	39	66	3	201
Basque Country	6	52	5	6	54	8	131
Catalonia	17	77	10	28	61	9	202
TOTAL Spain (ES)	100	337	41	102	363	57	1,000

Source: Authors' elaboration.

Table 7 shows the sampling errors for Andalusia, the Basque Country and Catalonia. The gender / age quota sampling errors are not shown as the sample size only allows for interpretation of the overall data for each one of the regions.

They are calculated for a probability no greater than 95.5%, and for the least desired context, i.e. a maximum indeterminate probability (p = q = 50%), for the reference population.

Table 7: Sampling errors by region

SAMPLING ERRORS	TOTAL
Andalusia	±7.05
Basque Country	±8.74
Catalonia	±7.04
TOTAL Spain (ES)	±3.16

Source: Authors' elaboration.

As gender or age quotas for the different Autonomous Regions were not established from the outset, each resulting sample must be weighted to allow for the interpretation of the specific data for Andalusia, the Basque Country and Catalonia. For this purpose, the population distribution in Spain, according to gender and age quotas, was used as a benchmark. Table 8 shows the weighting coefficients:

Table 8: Weighting factors by region

WEIGHTING FACTORS	FEMALE			MALE		
	16-24	25-54	55-74	16-24	25-54	55-74
Andalusia	0,9136	0,9961	2,7470	0,5257	1,1055	3,8190
Basque Country	1,1882	0,8841	0,8282	0,7359	1,2021	1,2793
Catalonia	2,1833	0,8490	1,0742	2,2270	0,8806	0,9334

Source: Authors' elaboration.

It should be noted that throughout the document, a (*) next to the data in the tables has been used to indicate statistically significant associations. These associations are positively indicated in the tables through analysis of the corrected standardised residuals. A statistically significant association is indicated in the cell when the statistical value is outside +1.96.

Finally, a brief reminder about the current research project is required. The data in the report refers to an Internet user population, which also forms part of online panels. Accordingly, it can be deduced that the respondents' profile as ICT users during the fieldwork process is more advanced than that the general population of the countries that were surveyed. In this sense, a new angle to the research project arises, which shouldn't be understated when indicating the future tendencies of the European population as a whole.

2.3 Field work process

The fieldwork period ran from 20 July 2011 to 20 August 2011. Three consecutive launches were established from the outset:

- The first launch took place in the United Kingdom (20.7.11) and Spain (21.7.11), which were the countries in which the pilot study took place.
- Secondly, and after having checked that no significant incidences existed, the launch went ahead in France and Italy on 26.7.11.
- Finally, a joint launch was to take place in the remaining countries on 29.7.11. Delays occurred in Finland (launched on 1.8.11), Slovenia (3.8.11) and Slovakia (4.8.11) due to issues with the optimisation of the questionnaire translations.

The fieldwork process included a pilot study to check the validity and reliability of the research design and the questionnaire (see Annex 3. Pilot study). The pilot study passed without notable incidences. The following table shows the data collection schedule for the different countries.

Table 9: Data collection schedule

COUNTRY	SAMPLE	COMPLETI ON	LAUNCH DATE	COMPLETIO N
Austria (AT)	1,000	100,0%	29.7.11	11.8.11
Belgium (BE)	1,000	100,0%	29.7.11	11.8.11
Germany (DE)	1,000	100,0%	29.7.11	4.8.11
Denmark (DK)	1,000	100,0%	29.7.11	15.8.11
Estonia (EE)	1,000	100,0%	29.7.11	10.8.11
Finland (FI)	1,000	100,0%	1.8.11	4.8.11
France (FR)	1,000	100,0%	26.7.11	8.8.11
Italy (IT)	1,000	100,0%	26.7.11	9.8.11
Netherlands (NL)	1,000	100,0%	29.7.11	15.8.11
Sweden SE)	1,000	100,0%	29.7.11	5.8.11
Slovenia (SI)	1,000	100,0%	3.8.11	17.8.11
Slovakia (SK)	1,000	100,0%	4.8.11	20.8.11
Spain (ES)	1,000	100,0%	21.7.11	5.8.11
United Kingdom (UK)	1,000	100,0%	20.7.11	1.8.11
TOTAL	14,000	100,0%	20.7.11	20.8.11

Source: Authors' elaboration.

Table 10 summarises the interview distribution by overall data and country within the fieldwork process:

- To achieve 14,000 responses, it was necessary to send 72,417 invitations to the panel, to which, 22,141 responses were received.
- 8,141 of 22,141 received responses were discarded, mainly as they did not fall into the required quotas (7,556), but because they have been rejected (585). The reason for rejecting a response was incompleteness and/or poor consistency of responses.

Table 10: Indicators of the fieldwork process

COUNTRY	Invitations	Non responses	Responses	Out of quota	Rejected	Sample
Austria (AT)	7,270	5,901	1,369	347	22	1,000
Belgium (BE)	7,158	5,907	1,251	221	30	1,000
Germany (DE)	3,068	1,253	1,815	759	56	1,000
Denmark (DK)	5,866	4,540	1,326	287	39	1,000
Estonia (EE)	4,164	2,943	1,221	202	19	1,000
Finland (FI)	3,898	2,448	1,450	399	51	1,000
France (FR)	5,346	3,132	2,214	1,147	67	1,000
Italy (IT)	5,095	3,116	1,979	924	55	1,000
Netherlands (NL)	5,125	3,471	1,654	621	33	1,000
Sweden SE)	3,013	1,701	1,312	283	29	1,000
Slovenia (SI)	4,050	2,807	1,243	234	9	1,000
Slovakia (SK)	4,264	2,895	1,369	357	12	1,000
Spain (ES)	6,809	5,281	1,528	455	73	1,000
United Kingdom (UK)	7,291	4,881	2,410	1,320	90	1,000
TOTAL	72,417	50,276	22,141	7,556	585	14,000

Source: Authors' elaboration.

The following graphs show the gross and net response rates respectively. Figure 5 shows the gross response rate. This corresponds to the proportion of received responses to the total number of invites. It can be observed that the average gross rate for all the countries is 30.6%, with relatively homogenous results, reaching a very high rate - 59.2% - in Germany.

Figure 5: Gross response rates

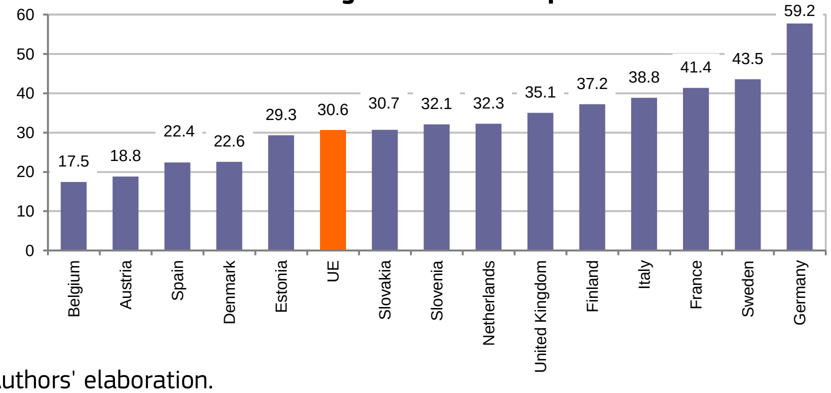

Source: Authors' elaboration.

Figure 6 shows the net response rate. This is obtained from the quotient between the validated interviews (1,000 per country, 14,000 in total) and the total number of invitations sent out (in each country and overall). The net response rate analysis excluded responses for over quota samples, and rejected interviews.

Figure 6: Net response rate

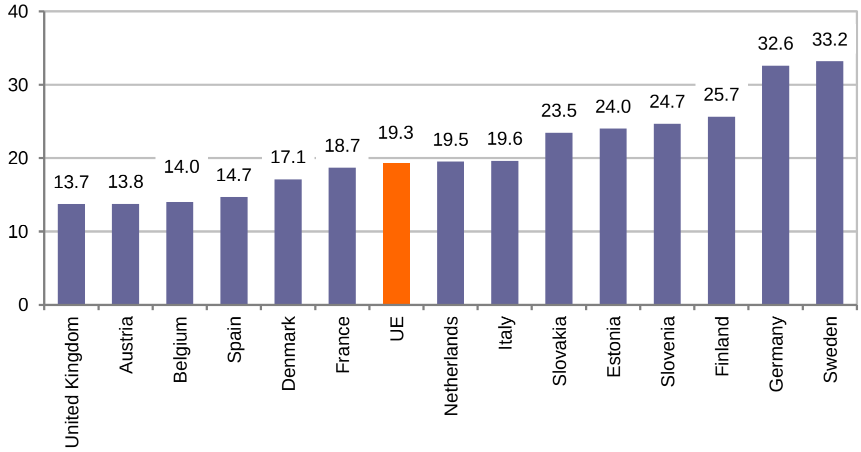

Source: Authors' elaboration.

Lastly, the average interview length was 23.2 minutes, with considerably homogenous results per country, varying between 20.5 minutes in the UK to almost 28 minutes in Estonia. Figure 7 summarises the interview length data per country:

Figure 7: Interview length (minutes)

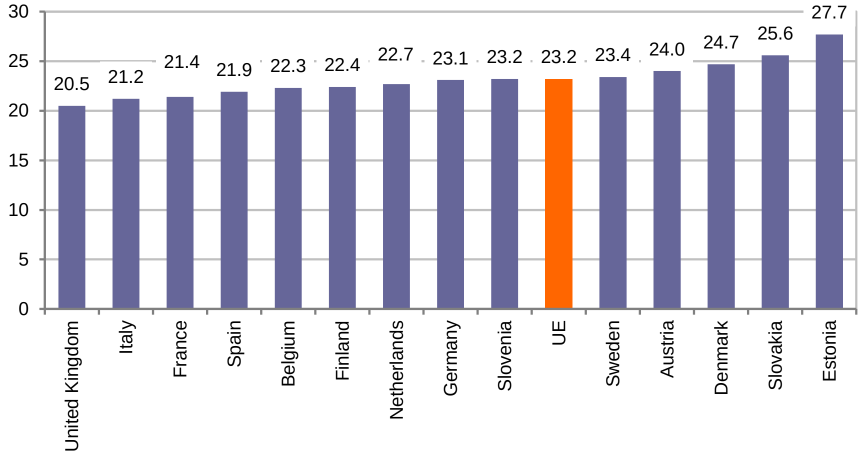

Source: Authors' elaboration.

2.4 Data analysis

Statistical analyses were performed using SPSS version 19.0 following three steps.

Firstly, descriptive statistical analysis was undertaken. This analysis includes frequencies of all items and cross tabulation with socio-demographics and health status. To attribute statistical significance to the differences obtained an associated Chi-square test was carried out.

Secondly, following our conceptual framework, in order to confirm the several internal complementarities of grouped items, the means and their significant correlation were checked. Then, factor analysis was used to assess item correlations and identify common relationships between similar items, allowing the items to be categorized into various themes or factors (dimensions). An analysis of the correlation matrix (KMO and Bartlett's test of sphericity) was carried out to check that the correlation matrixes were factorable. Data reductions were undertaken by principal components analysis using the Varimax option to identify possible underlying

dimensions. Factors identified reveal a pattern of correlations within a set of observed variables related to the main blocks of our conceptual framework.

Thirdly, ANOVA test and correlations were carried out to identify the relationship among the dimensions previously identified and to characterise different typologies of users, behaviours, motivations. To attribute statistical significance to the differences obtained associated tests were carried out.

3. SOCIO-DEMOGRAPHIC PROFILE OF THE PARTICIPANTS

3.1 Gender

Now that the sample characteristics of the citizens taken from the 14 European countries forming part of the research have been discussed in detail, we will now approach the explanation of their socio-demographic characteristics.

The sample of the European citizens being researched is split nearly evenly by gender, with slightly more women taking part (51.5%).

Figure 8: Gender (E1)

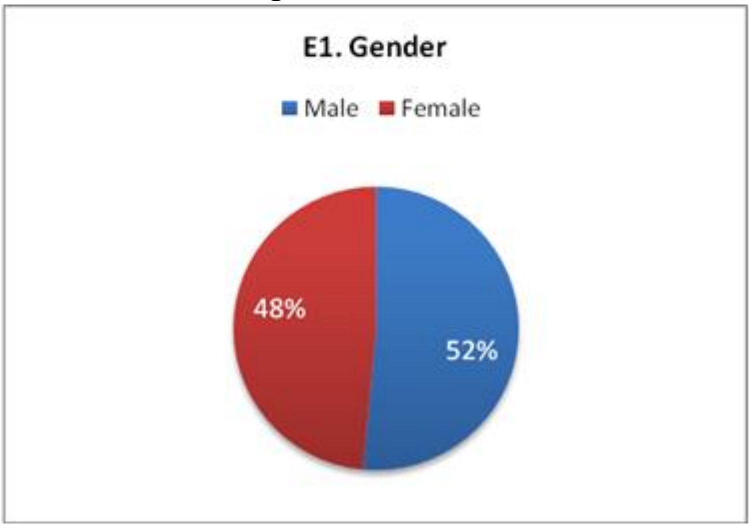

Base: Whole sample.

No significant differences are observed by country in terms of the sample distribution by gender. It is only worth mentioning the relatively higher number of men in Italy (54.4%) and women in Estonia (53.6%).

Figure 9: Gender (E1) by country

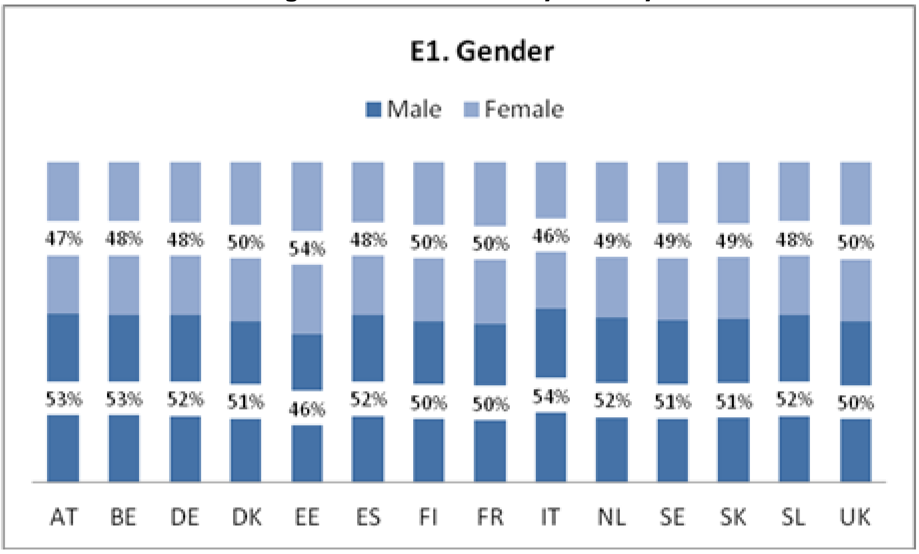

Base: Whole sample.

3.2 Age

As for the age structure of the sample, some relevant differences are observed here. Almost two-thirds of the total number of persons sampled (62.2%) fall within the middle age group (between 25 and 54 years old). Additionally, young citizens (between 16 and 24 years old) make up 19.8% of the sample, with 18% of the sample consisting of older citizens (between 55 and 74 years old).

Figure 10: Age (E2)

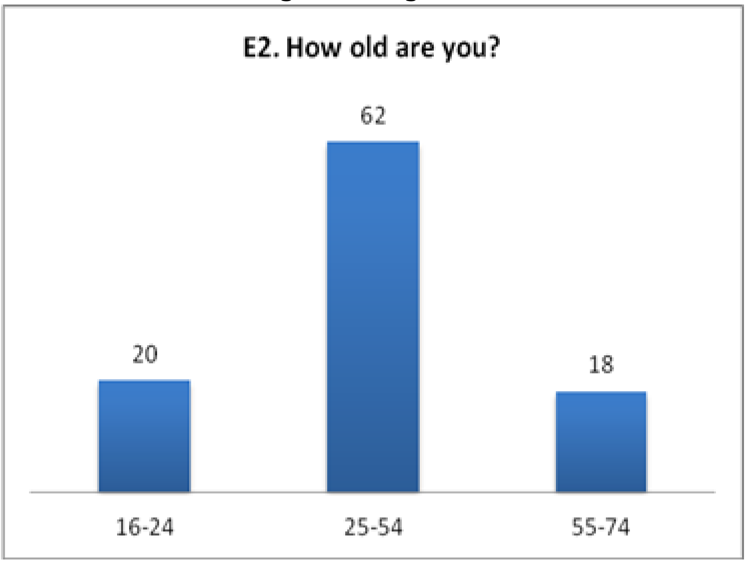

Base: Whole sample.

On a per-country basis, there are relatively more young citizens in the samples for Estonia (24.0%) and Slovakia (23.5%). It is also worth highlighting the presence of respondents from an older population (between 55 and 74 years old) in the Scandinavian countries: 23.8% in Denmark, 23.0% in Finland, and 24.5% in Sweden.

Figure 11: Age (E2) by country

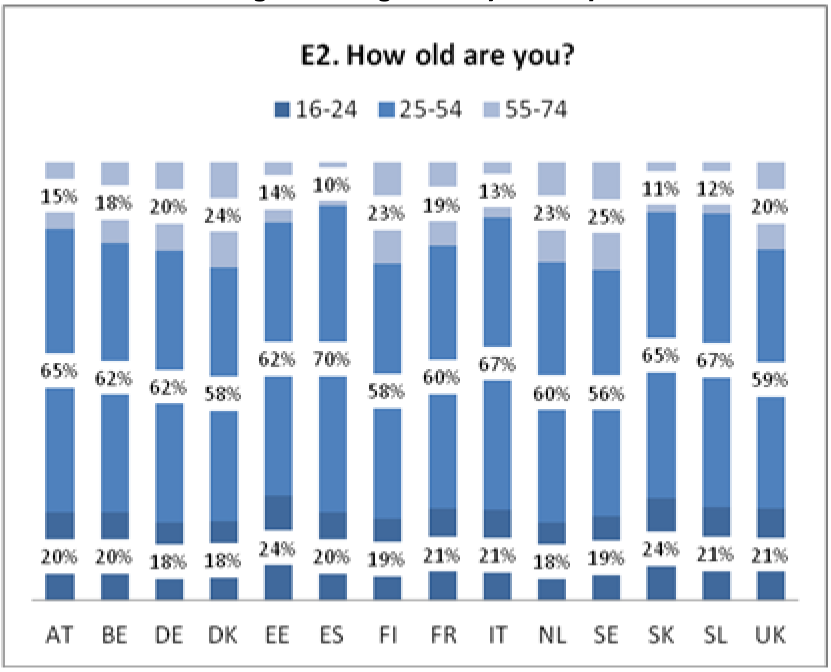

Base: Whole sample.

3.3 Country of citizenship

Virtually all the sampled European population are citizens of their country (95%), a percentage that rises to 98% for EU citizens. Therefore, only 2% of the sample relates to non-EU member state nationals.

Figure 12: Country of citizenship (E3)

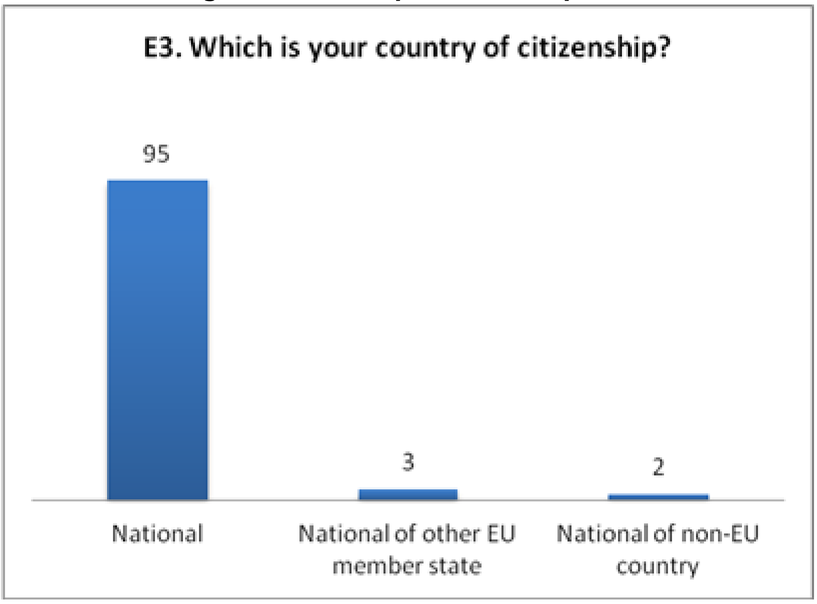

Base: Whole sample.

3.4 Country of birth

As with nationality, the large majority of the participating citizens are native to the country (93%) or born in EU countries (96%). Therefore, only 4% of the sample was born outside of the EU.

Figure 13: Country of birth (E4)

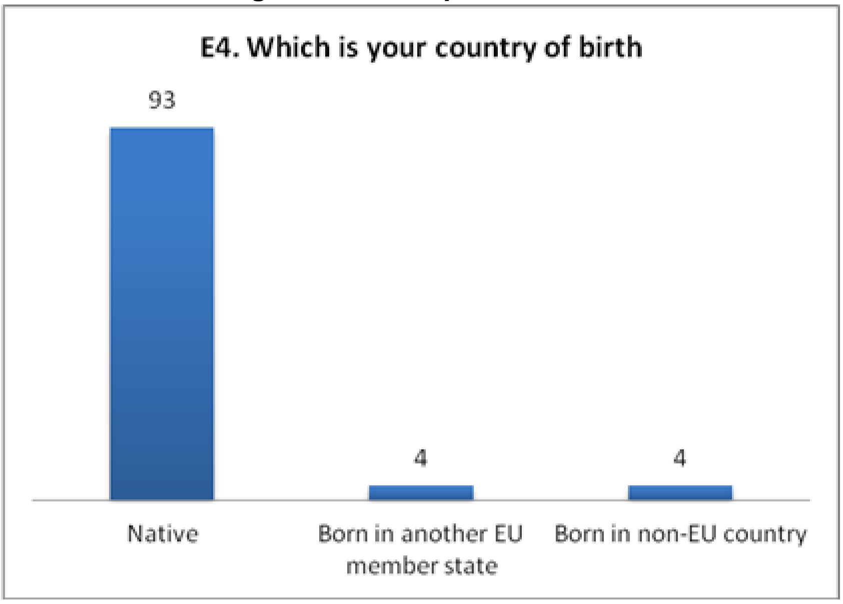

Base: Whole sample.

3.5 Level of education

With respect to the level of education, around half of the sampled European population (46%) attained the secondary education level, slightly more than the 38.8% of citizens who attained university level education. 15% of the sampled population attained the primary or lower secondary education level.

Figure 14: Level of education completed (E5)

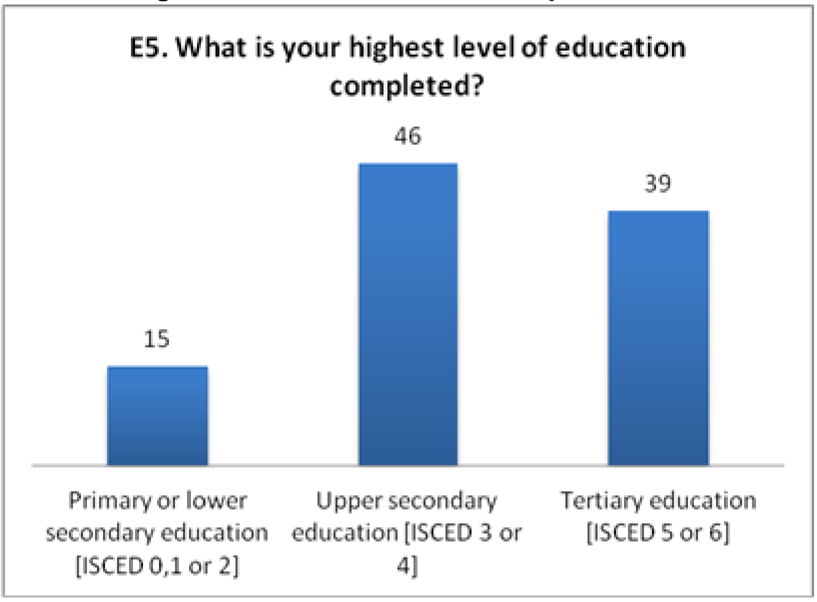

Base: Whole sample.

On a per-country basis, the following scenarios can be highlighted, considering that the sample is composed of Internet users which means that the lower the diffusion of the Internet in a given country, the higher the education level among respondents in that country

- Belgium (55.6%), Spain (53.7%), France (45%), Sweden (42%) and the United Kingdom (44%) stand out in terms of participating citizens with a university education;
- Austria (29%), Denmark (45%) and Finland (46%) stand out in terms of a greater relative presence of lower education levels, much higher than in their general population.

Figure 15: Level of education completed (E5) by country

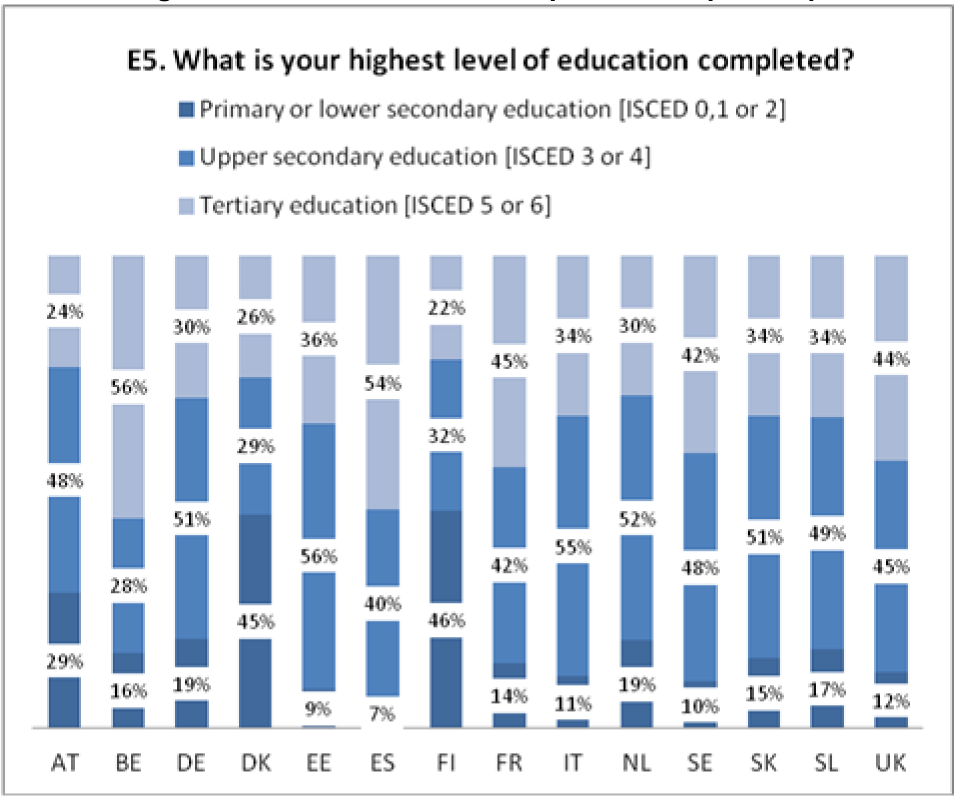

Base: Whole sample.

3.6 Labour position

With respect to entry into the workplace, more than half of the sampled population (58%) were employed or self-employed, 10% unemployed, 14% were students and 18% were not part of the labour force for different motives.

Figure 16: Labour position (E6)

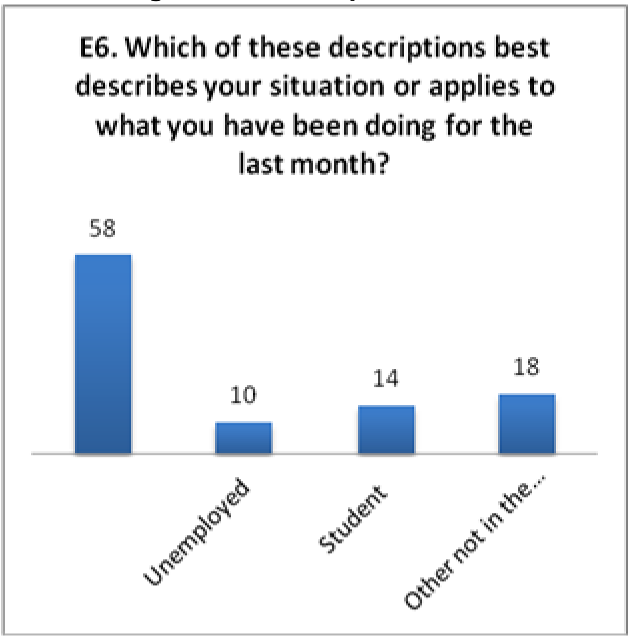

Base: Whole sample.

On a per-country basis, the high employment ratios in Estonia (73%), France (63%) and Sweden (62%) stand out. On the other hand, and in tune with the data for the country as a whole, 20% of the people sampled in Spain are unemployed. By job category, office clerks (19%), customer services clerks (10%), personal and social services (7%), associate professionals (6%) and small enterprise managers (6%) stand out.

Figure 17: Labour position (E6) by country

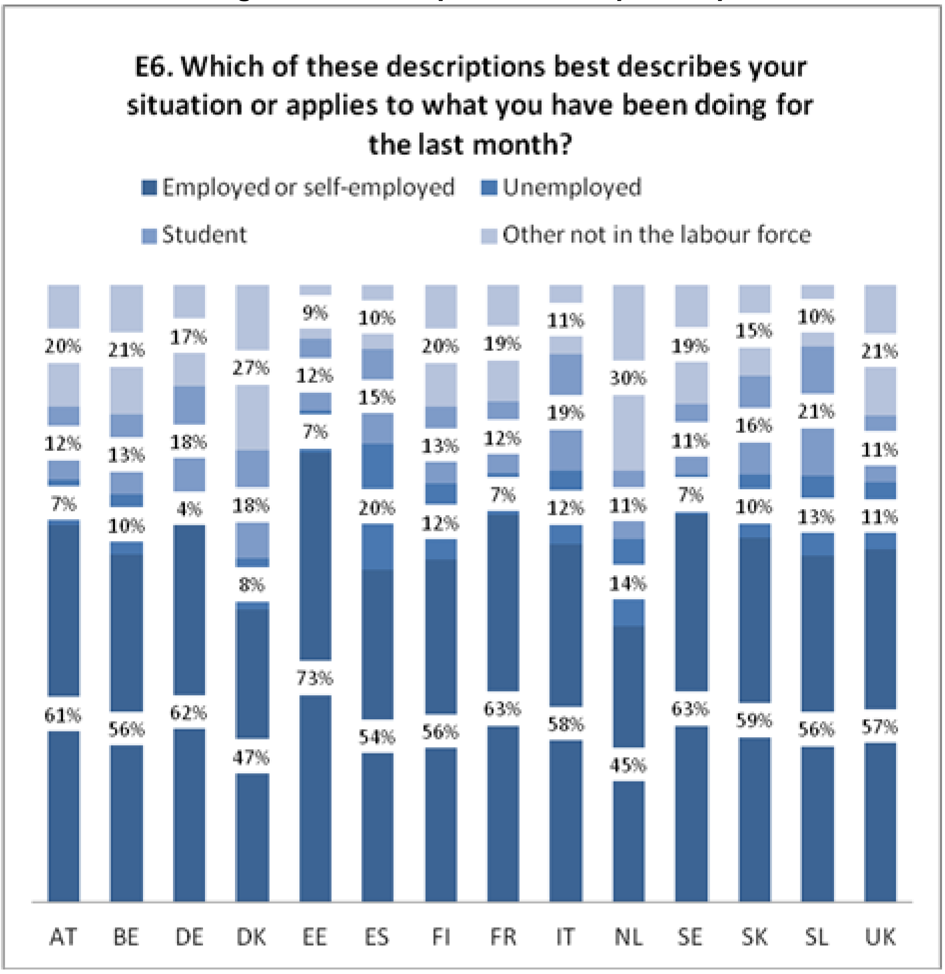

Base: Whole sample.

3.7 Type of location

With respect to the urban density of the sampled population, the following distribution is worth indicating: 39% live in densely populated areas, 39% in intermediate size cities, and 22% in thinly populated areas.

Figure 18: Type of locality (E9)

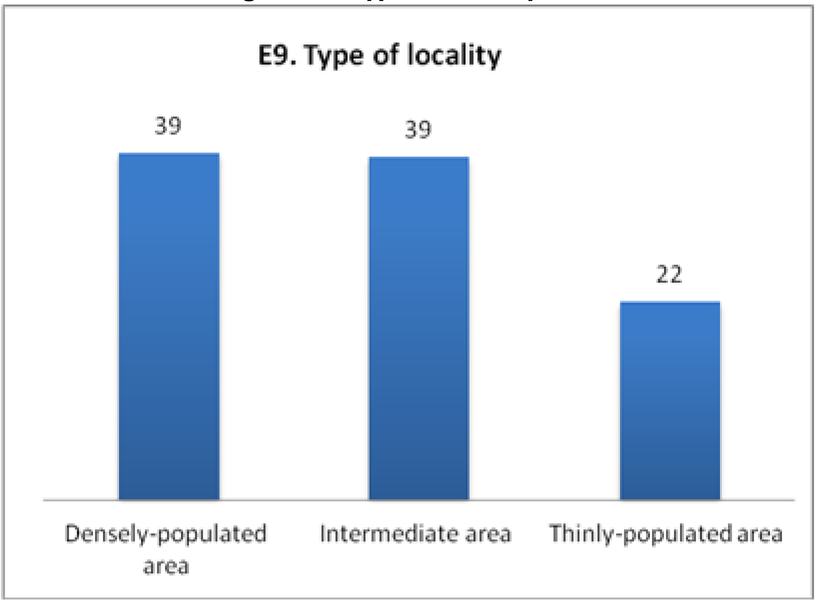

Base: Whole sample.

The samples in Estonia (65%), Spain (59%), Italy (46%), Holland (44%) and Sweden (49%) stand out for the significant proportion of citizens who live in densely populated areas.

Figure 19: Type of locality (E9) by country

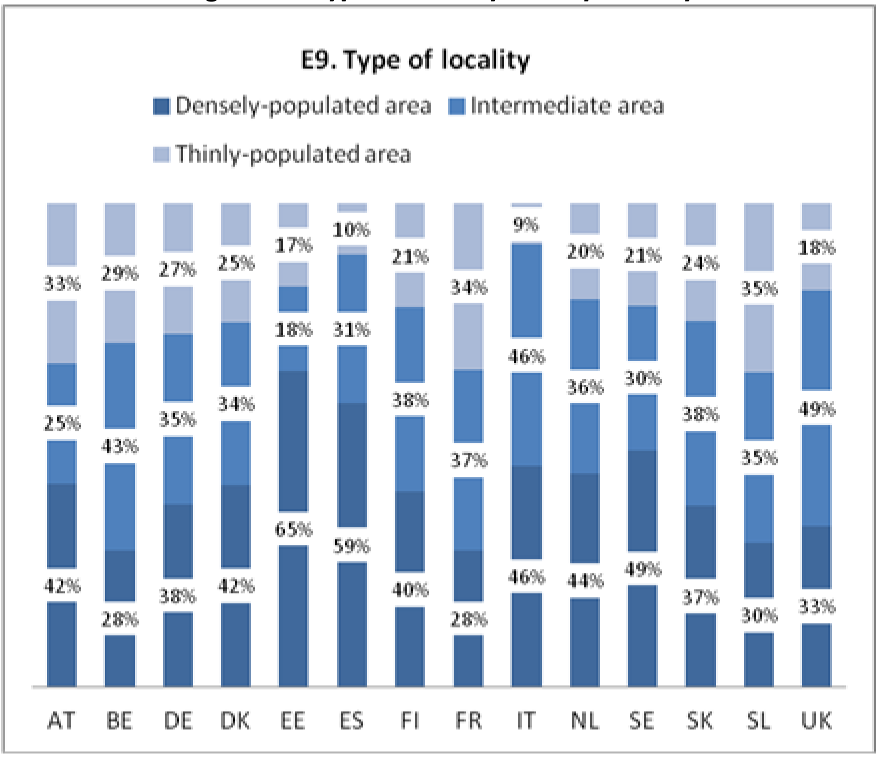

Base: Whole sample.

3.8 Members in the household

Around a third of the sampled population (31%) live in 2-member households, and 32% live in a household with 4 or more members. 16% of the citizens sampled live in single-parent households, and 22% in households with 3 members.

Figure 20: Members in the household (E19)

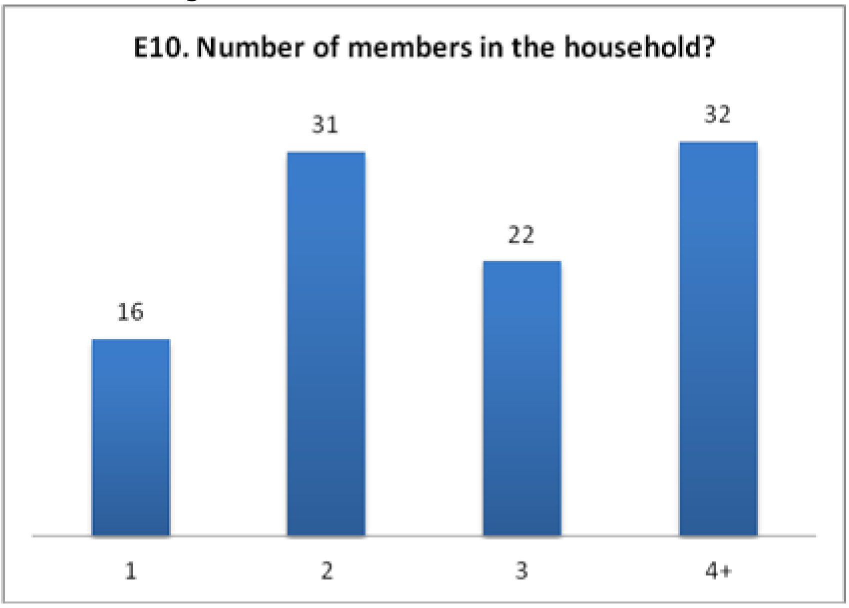

Base: Whole sample.

On a per-country basis, there are households with many members in Estonia (39%), Spain (45%), Italy (45%), Slovakia (50%) and Slovenia (47%). In turn, the Scandinavian countries, particularly Denmark and Finland, fewer member households are more prevalent than the sample average.

Figure 21: Members in the household (E19) by country

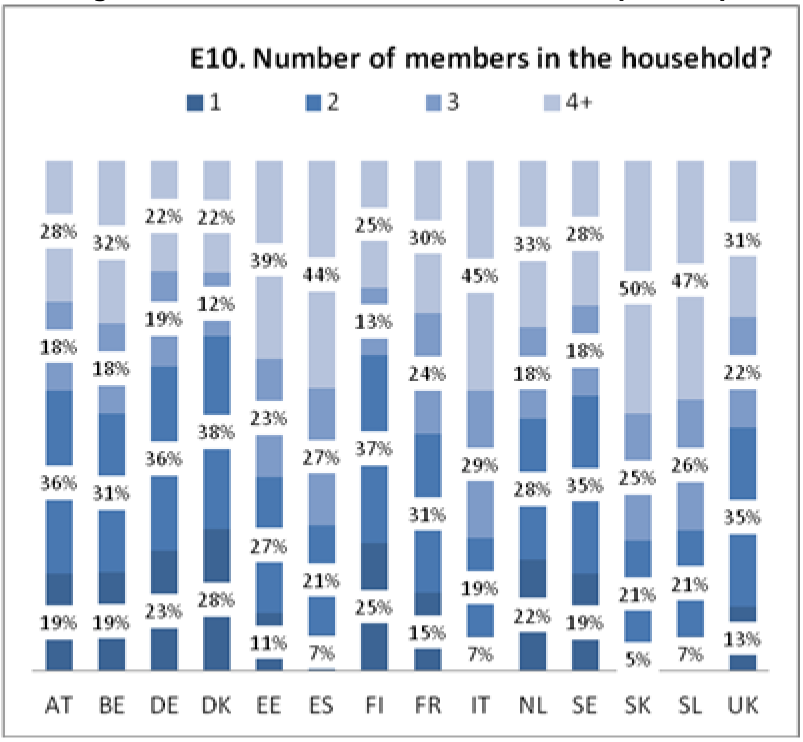

Base: Whole sample.

3.9 Socio-demographic comparison: Internet users (sample) and population

To be fully transparent a comparison of socio-demographic characteristics between our sample of Internet users and population has been carried out. As it was expected Internet users are more likely than the general population of 14 EU countries surveyed to be younger, have higher levels of education; and be employed.

Table 11: Socio-demographic comparison

		Sample	Population
Gender	Male	52	50
	Female	48	50
Age group	16-24	20	29
	25-54	62	56
	55-74	18	29
Country of birth	Native	93	77
	Born in another EU member state	4	7
	Born in non-EU country	4	16
Level of education completed	Primary or lower secondary education	15	34
	Upper secondary education	46	43
	Tertiary education	39	23
Situation	Employed or self-employed	58	58
	Unemployed	10	6
	Other	32	36
Type of locality	Densely-populated area	39	52
	Intermediate area	39	30
	Thinly-populated area	22	18

Base: Whole sample *Source*: EUROSTAT population.

The same comparison has been also carried out by country. It is worth pointing out that the differences are even more accentuated: the lowest the level of Internet use, the highest the differences.

Table 12: Socio-demographic comparison by country (I)

		AT		BE		DE		DK		EE		ES		FI	
		Sample	Population	Sample	Population	Sample	Population	Sample	Population	Sample	Population	Sample	Population	Sample	Population
Gender	Male	53	50	53	50	52	50	50	50	46	47	52	50	50	50
	Female	47	50	47	50	48	50	50	50	54	53	48	50	50	50
Age group	16-24	20	16	20	16	18	14	18	17	24	17	20	14	19	16
	25-54	65	57	62	56	62	55	58	53	62	55	70	61	58	52
	55-74	15	27	18	28	20	31	24	30	14	28	10	25	23	32
Level of education completed	Primary or lower secondary education	29	25	16	36	19	22	45	33	9	20	7	52	46	27
	Upper secondary education	48	59	28	35	51	56	29	40	56	51	40	22	32	43
	Tertiary education	24	16	56	29	30	22	26	27	35	29	53	26	22	31
Situation	Employed or self-employed	61	64	56	56	62	63	47	65	72	59	55	52	55	62
	Unemployed	7	3	10	4	4	4	8	5	7	9	20	14	12	6
	Other	32	33	34	40	34	33	45	30	21	32	25	34	33	32
Type of locality	Densely-populated area	42	36	28	54	38	49	41	34	65	46	59	52	41	26
	Intermediate area	25	25	43	42	35	36	34	42	18	3	31	22	38	14
	Thinly-populated area	33	39	29	4	27	15	25	25	17	51	10	27	21	61

Base: Whole sample *Source*: EUROSTAT population.

Table 13: Socio-demographic comparison by country (II)

		FR		IT		NL		SE		SL		SK		UK	
		Sample	Population	Sample	Population	Sample	Population	Sample	Population	Sample	Population	Sample	Population	Sample	Population
Gender	Male	50	49	54	49	51	50	51	51	52	51	51	49	50	50
	Female	50	51	46	51	49	50	49	49	48	49	49	51	50	50
Age group	16-24	21	17	21	13	18	16	19	18	21	14	24	18	21	18
	25-54	60	55	67	57	60	55	56	52	67	57	65	58	59	55
	55-74	19	28	12	30	22	29	25	30	12	28	11	25	20	27
Level of education completed	Primary or lower secondary education	14	35	11	51	19	34	10	27	17	23	15	18	12	25
	Upper secondary education	42	41	55	37	51	39	48	45	49	58	51	68	44	44
	Tertiary education	45	25	34	12	30	27	42	28	34	20	34	15	44	31
Situation	Employed or self-employed	62	57	58	50	45	67	63	66	56	58	59	55	57	63
	Unemployed	7	5	12	4	14	3	7	6	13	5	10	8	11	5
	Other	31	37	30	45	41	30	30	28	31	37	31	37	32	32
Type of locality	Densely-populated area	28	47	46	44	44	65	49	21	30	19	38	27	33	80
	Intermediate area	37	35	46	40	36	33	30	16	35	37	38	32	49	16
	Thinly-populated area	35	18	8	16	20	2	21	63	35	44	24	41	18	4

Base: Whole sample *Source*: EUROSTAT population.

4. HEALTH STATUS AND HEALTH AND SOCIAL CARE UTILISATION

4.1 Health status

Overall, the European population is in a favourable state of health. Around three-quarters of the sample (74%) state they are in a good state of health, 18% state that it is neither good nor bad, and 7% of the sample population state that they are in a poor state of health.

Figure 22: Health status (A4)

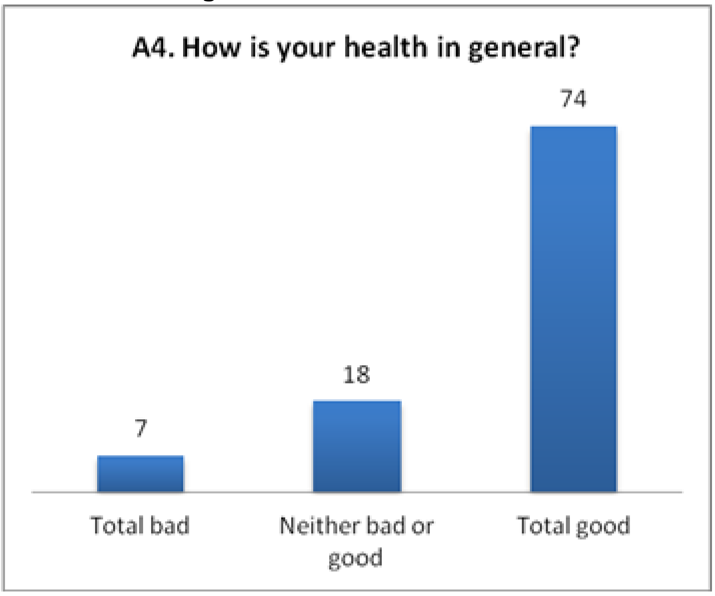

Base: Whole sample.

Poor state of health is associated with the older population (11%); the population with lower education levels (10.7%); the unemployed (9.5%) and inactive (17%); the population living in thinly populated areas (8.3%); single parent households (12%); and long-standing patients (17%). On the other hand, a positive state of health is related to men (76%); young people (88%); a university education (78%); entrepreneurs and the self-employed (78%), students (87%); the population living in densely populated areas (75%); households with several members; and the absence of long-standing illness (92%).

Table 14: Health status (A4) by socio-demographics

A4. How is your health in general?		Bad	Neither good or bad	Good
Gender	Male	7	18	76*
	Female	8*	19*	73
Age group	16-24	3	10	88*
	25-54	8*	18	74
	55-74	11*	28*	61
Level of education completed	Primary or lower secondary education	11*	23*	67
	Upper secondary education	7	19	74
	Tertiary education	6	16	78*
Situation	Employed or self-employed	5	17	78*
	Unemployed	10*	23*	68
	Student	3	10	87*
	Other not in the labour force	17*	28*	54
Type of locality	Densely-populated area	7	18	75*
	Intermediate area	7	19	74
	Thinly-populated area	8*	18	73
Members in the household	1	12*	22*	66
	2	8*	19	73
	3	7	19	75*
	4+	5	16	79*
Long standing illness	Yes	17*	33*	50
	No	1	7	92*

Base: Whole sample.

Among those countries sampled, Spain (82%), France (81%) and Slovakia (82%) stand out as having good states of health, whereas in Germany (11%), Denmark (12%) and Holland (10%), the worst states of health are clearly seen to be above the sample average.

Figure 23: Health status (A4) by country

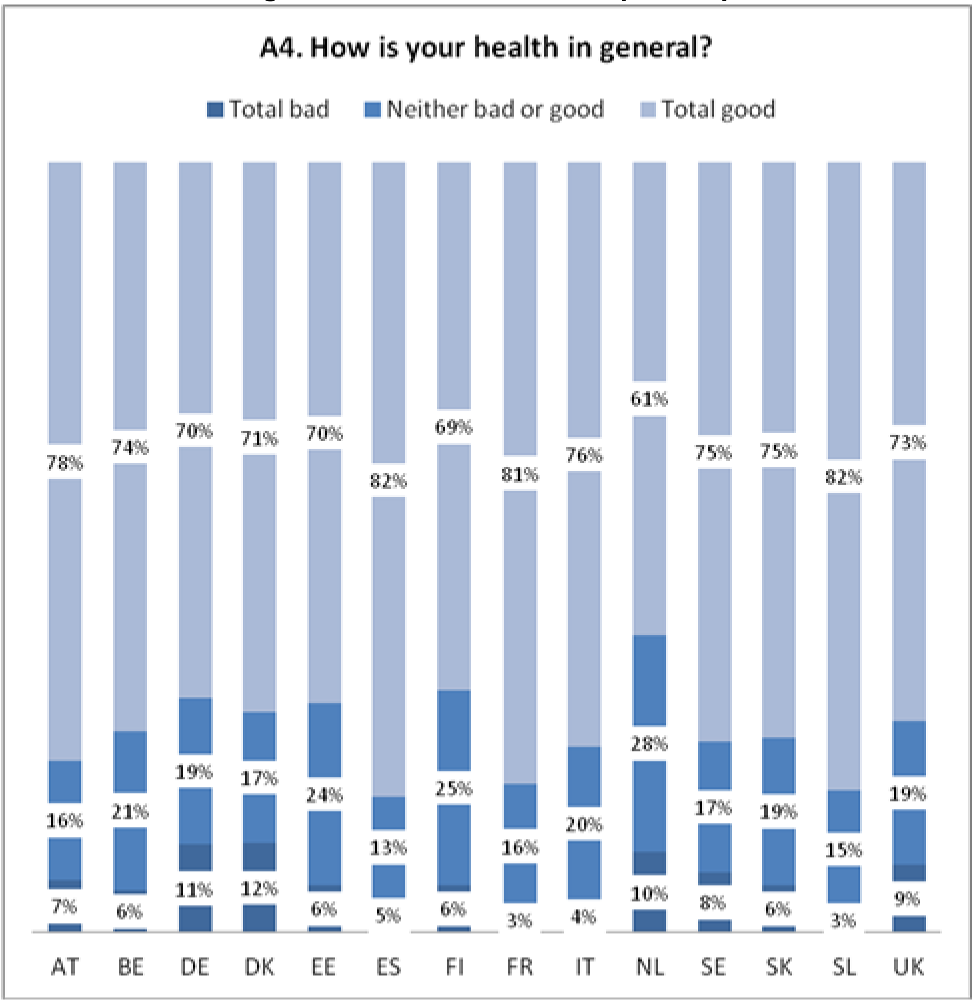

Base: Whole sample.

However, more than half of the sampled population (56%) stated that they have (or have had) a long-standing illness or health problem.

Figure 24: Long-standing illness or health problem (A5)

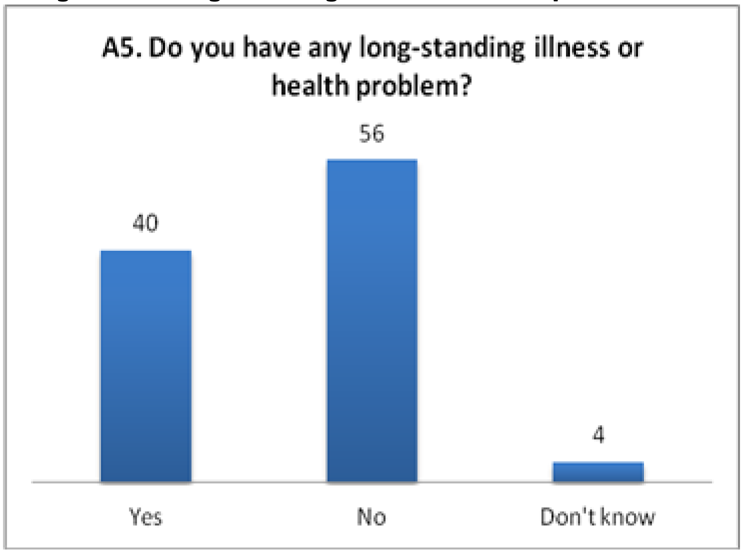

Base: Whole sample.

Through an analysis of descriptive statistics, it is possible to link the long-standing illnesses or health problems to women (42% of the total); the older population (62% of citizens aged 55 to 74 years old); the lower level of education; unemployment (44%) and inactivity (64%); the poor state of health (92%); and households with few members. On the other hand, the absence of a long-standing illness or health problems are associated with men (58%), the young (71%), a university education (60%), self-employment and entrepreneurs (60%), a good state of health overall (70%), and larger households.

Table 15: Long-standing illness of health problem (A5) by socio-demographics

		Yes	No	Don't Know
Gender	Male	39	58*	3
	Female	42*	55	3
Age group	16-24	23	71*	6
	25-54	40	57*	3
	55-74	62*	35	3
Level of education completed	Primary or lower secondary education	44*	52	4
	Upper secondary education	42*	55	3
	Tertiary education	37	59*	4
Situation	Employed or self-employed	37	60*	3
	Unemployed	44*	51	5
	Student	24	71*	5
	Other not in the labour force	64*	34	2
Health status	Bad	93*	5	2
	Neither good or bad	73	22	5
	Good	27	70*	3
Members in the household	1	47*	49	4
	2	45*	52	3
	3	39	58*	3
	4+	34	62*	4

Base: Whole sample.

On a per-country basis, the existence of long-standing illness and health problems is more frequent in Germany (48%), Denmark (45%) and Finland (45%), whilst the absence of long-standing illness and health problems is more prevalent in Belgium (63%), France (65%), Italy (61%) and Slovakia (59%).

Figure 25: Long-standing illness or health problem (A5) by country

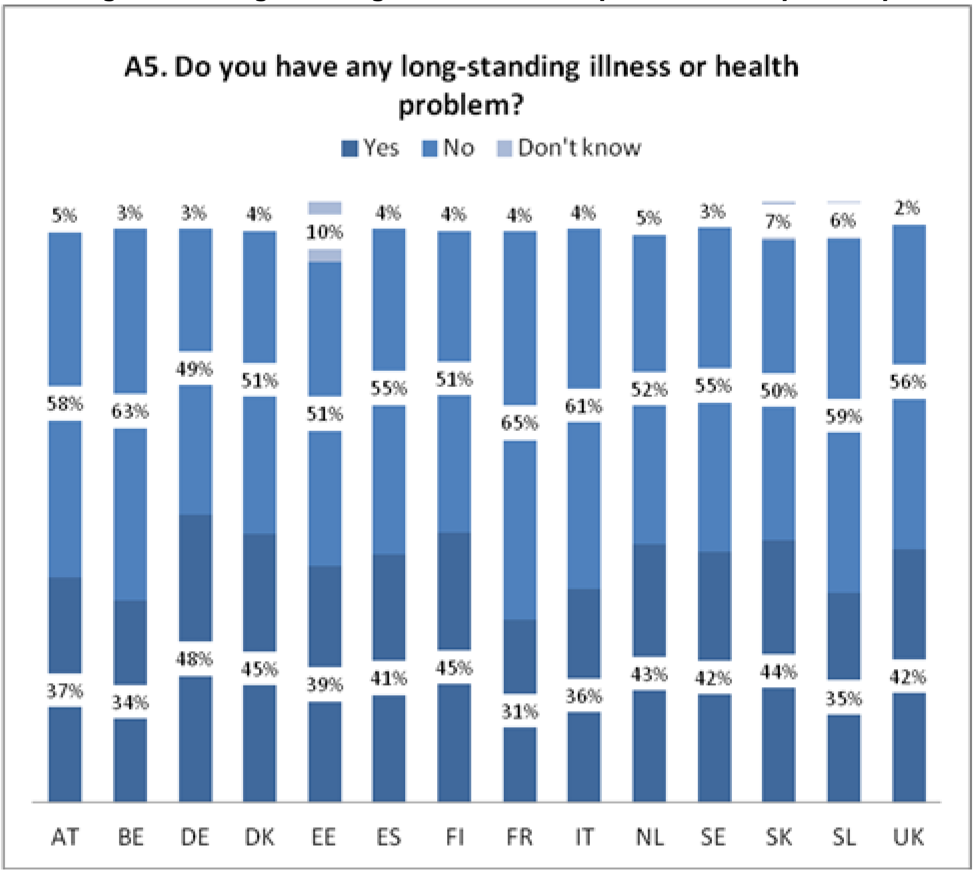

Base: Whole sample.

It is also worth highlighting that 65% of the sampled population state that they have undergone a long-term medical treatment.

Figure 26: Long-term medical treatment (A6)

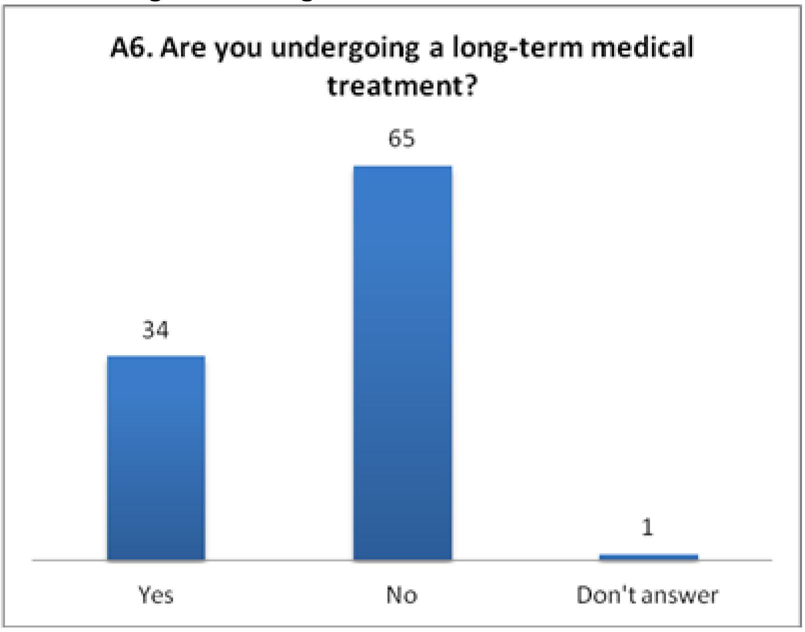

Base: Whole sample.

Again, women, the older population, lower education levels, the inactive, those residing in thinly populated areas, households with few members, a poor state of health and the existence of long-standing illnesses are statistically linked with long term medical treatments.

Table 16: Long-term medical treatment (A6) by socio-demographics

		Yes	No	Don't Know
Gender	Male	32	67*	1
	Female	35*	64	1
Age group	16-24	16	82*	2
	25-54	32	67*	1
	55-74	59*	40	1
Level of education completed	Primary or lower secondary education	39*	59	1
	Upper secondary education	35*	65	1
	Tertiary education	31	68*	1
Situation	Employed or self-employed	30	69*	1
	Unemployed	35	63	1
	Student	17	82*	2
	Other not in the labour force	60*	39	1
Type of locality	Densely-populated area	33	66*	1
	Intermediate area	34	65	1
	Thinly-populated area	36*	63	1
Members in the household	1	39*	60	1
	2	39*	60	1
	3	32	68*	1
	4+	27	72*	1
Health status	Bad	84*	15	1
	Neither good or bad	61*	38	2
	Good	22	77*	1
Long standing illness	Yes	72*	27	1
	No	7	93*	0

Base: Whole sample.

Germany (40% of the total), Spain (34%), Sweden (36%) and the United Kingdom (35%) stand out for having a greater relative population on long-term medical treatments.

Figure 27: Long-term medical treatment (A6) by country

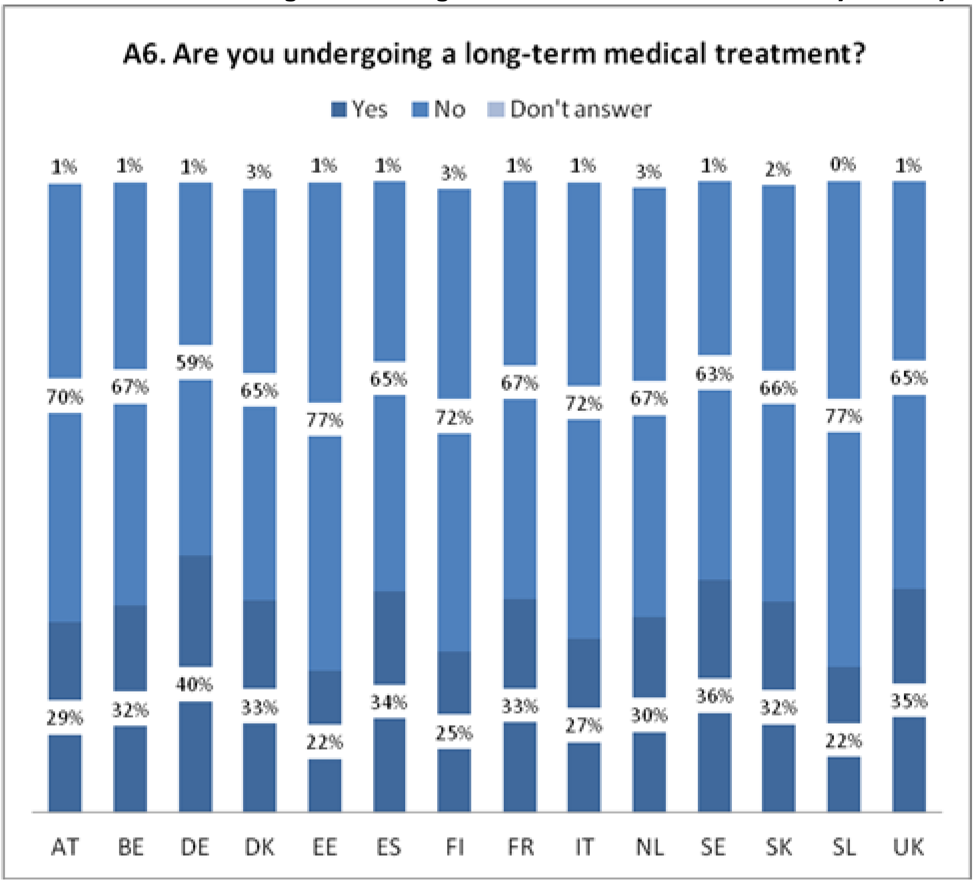

Base: Whole sample.

In the same way, 36% of the sampled population state that their regular life has been severely restricted due to a health problem.

Figure 28: Limited in activities people normally do due to a health problem (A7)

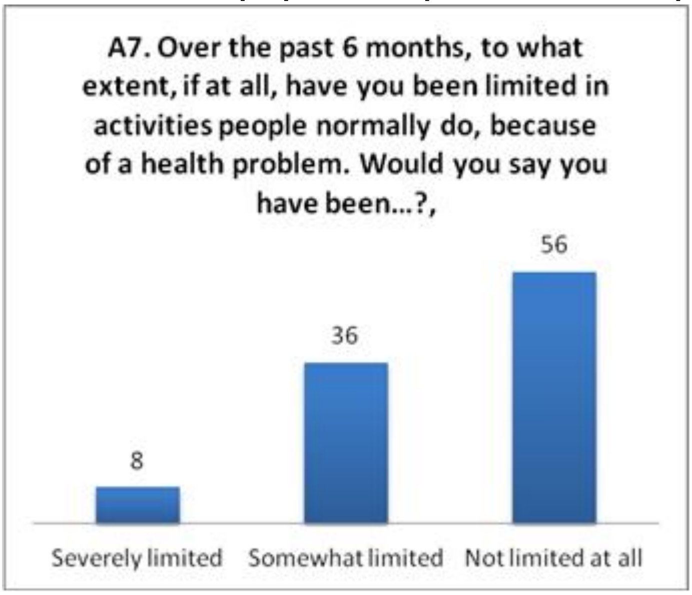

Base: Whole sample.

This severe restriction to normal life as a result of a health problem is linked with the older population, lower education levels, unemployment and inactivity, thinly populated areas, households with few members, a poor state of health and the presence of long-standing illnesses.

Table 17: Limited in activities people normally do, because of a health problem (A7)

		Severely limited	Somewhat limited	Not limited at all
Gender	Male	8	35	57
	Female	8	37	55
Age group	16-24	4	34	62*
	25-54	9*	35	57*
	55-74	11*	42*	47
Level of education completed	Primary or lower secondary education	12*	40*	49
	Upper secondary education	9*	36	55
	Tertiary education	6	34	60*
Situation	Employed or self-employed	6	34	60*
	Unemployed	10*	36	53
	Student	4	35	61*
	Other not in the labour force	18*	42*	40
Type of locality	Densely-populated area	7	35	57*
	Intermediate area	8	36	56
	Thinly-populated area	10*	36	54
Members in the household	1	12*	38	50
	2	9*	37	55
	3	7	35	58*
	4+	6	35	59*
Health status	Bad	50*	46*	5
	Neither good or bad	13*	60*	27
	Good	3	29	68*
Long standing illness	Yes	17*	52*	31
	No	2	23	75*

Base: Whole sample.

The countries with the greatest limitations on normal life as a result of health problems are Austria (42%), Germany (46%) and Estonia (45%).

Figure 29: Limited in activities people normally do due to a health problem (A7) by country

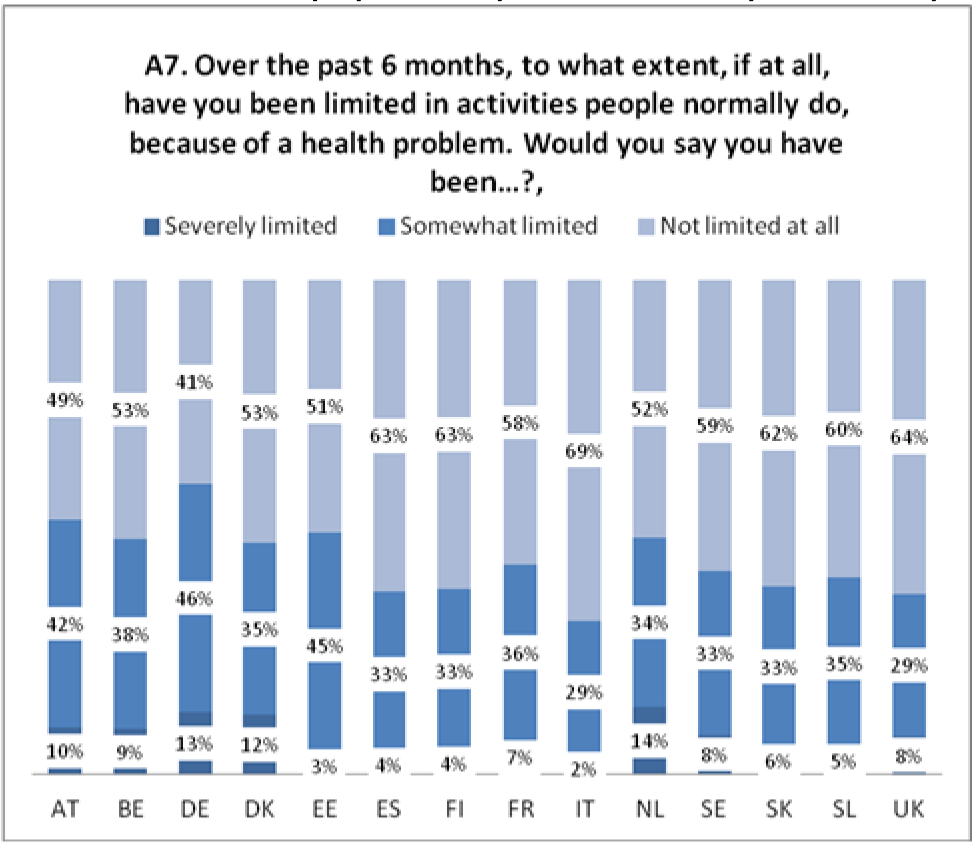

Base: Whole sample.

Among the health issues most widely experienced by the sampled population, allergies (35.0% of the total), migraines and headaches (30%), muscle pains (24%), anxiety and depression (20%), hypertension (18%) and asthma (12%) stand out.

Figure 30: Health problems reported (A8)

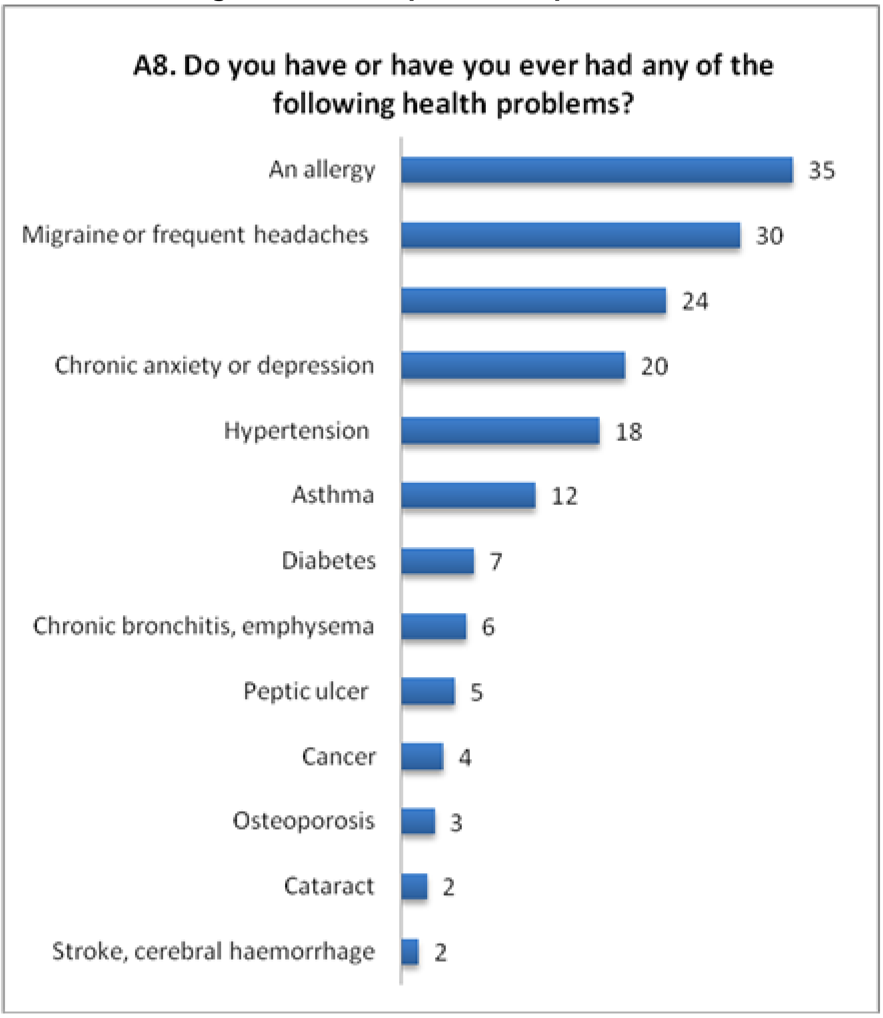

Base: Whole sample.

In general, these health problems are statistically linked with the female population, the older population, low education levels, unemployment and inactivity, poor states of health and long-standing illnesses.

Table 18: Health problems (A8) by socio-demographic

		Allergy	Migraine or frequent headaches	Troubles muscles, bones …	Chronic anxiety or depression	Hyper-tension	Asthma	Diabetes	Chronic bronchitis,	Peptic ulcer	Cancer	Osteo-porosis	Cataract	Stroke, cerebral haemorrhage
Gender	Male	31	22	21	16	20*	12	8*	6	6*	3	2	2	2*
	Female	39*	39*	26*	24*	15	13	5	6	4	4*	4*	2	1
Age group	16-24	44*	34*	11	18	5	16*	2	4	3	1	1	1	1
	25-54	36	32*	22	21*	15	12	5	6	5	3	2	1	1
	55-74	24	20	42	18	41	9	16*	8*	7*	9*	7*	8*	3*
Level of education completed	Primary or lower secondary education	30	32	29*	23*	22*	11	9*	7*	6*	5*	5*	3	2*
	Upper secondary education	33	31	25*	20	18*	12	6	6*	5	4	3	3	1
	Tertiary education	39*	29	20	19	15	13	6	5	4	3	2	2	2
Situation	Employed or self-employed	35	29	21	17	16	11	5	5	5	3	2	2	1
	Unemployed	36	39*	26*	31*	18	14*	7	8*	4	2	4*	3	2*
	Student	45*	32*	10	17	4	15*	1	4	2	1	1	1	1
	Other not in the labour force	28	28	43*	28*	35*	11	14*	9*	7*	9*	7*	6*	3*
Type of locality	Densely-populated area	38*	31	22	21*	17	14	6	7*	5*	4	3	2	2*
	Intermediate area	34	31	23	19	18	12	7	5	4	4	2	2	1
	Thinly-populated area	32	29	27*	19	20*	10	7	4	5	4	4	2	1
Health status	Bad	39*	46*	59*	50*	38*	22*	20*	16*	11*	12*	12*	6*	7*
	Neither good or bad	35	36*	41*	30*	30*	15*	13*	10	7*	6	6*	3	2
	Good	35	27	16	15	13	10	4	4	4	2	2	2	1
Long standing illness	Yes	39*	36*	41*	31*	31*	19*	14*	9*	8*	7*	6*	4*	3*
	No	32	26	11	12	8	7	1	3	3	2	1	1	1

Base: Whole sample.

On a per-country basis and main health issues, the high percentages of allergies in Finland and Sweden stand out (43% and 40% of the total respectively), migraines and headaches in Italy (40%), and chronic anxiety and depression in Spain (25%).

Table 19: Health problems (A8) by country

	AT	BE	DE	DK	EE	ES	FI	FR	IT	NL	SE	SK	SL	UK
Diabetes	7	7	9	9	4	4	8	5	6	10	4	5	5	7
An allergy	35	32	39	32	35	38	43	32	38	26	40	38	36	30
Asthma	8	7	11	14	4	13	14	11	11	11	14	7	8	16
Hypertension	19	16	22	17	20	14	22	15	16	19	19	26	18	17
Long-standing troubles with muscles, bones and joints	19	29	26	29	26	21	21	20	25	26	23	23	13	24
Cancer	4	3	4	6	3	2	3	3	3	6	4	2	2	5
Cataract	3	2	2	4	1	2	3	1	3	3	4	3	2	3
Migraine or frequent headaches	29	30	31	28	29	30	29	32	40	25	26	28	21	26
Chronic bronchitis, emphysema	8	7	8	5	7	5	5	5	7	10	2	4	5	2
Osteoporosis	4	4	3	4	2	3	3	3	4	5	1	5	2	3
Stroke, cerebral haemorrhage	2	1	2	3	1	2	2	0	2	4	2	1	1	2
Peptic ulcer	7	7	6	7	11	5	4	3	4	5	8	5	7	4
Chronic anxiety or depression	19	21	19	20	21	25	17	21	19	19	15	16	14	20

Base: Whole sample.

4.2 Informal carers

With respect to long-standing illnesses or health disabilities, more than half, specifically 55% of the sampled European population indicate that someone close to them has these problems.

Figure 31: Someone close to you currently experience long-term illness of disability (A9)

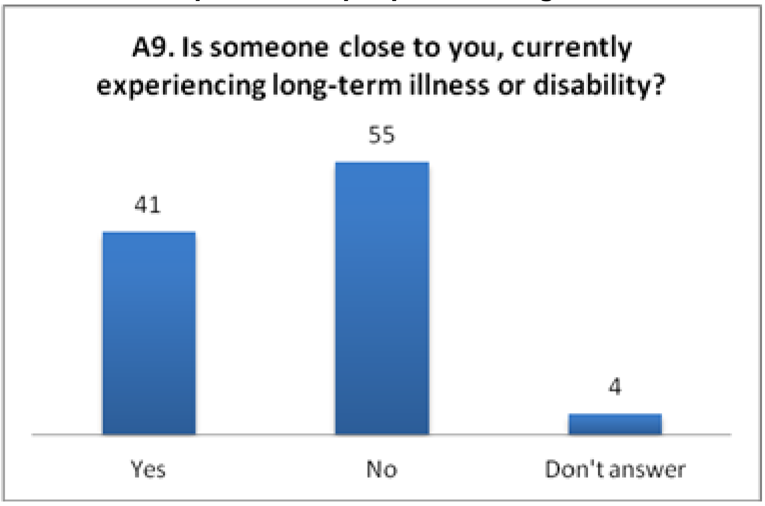

Base: Whole sample.

The closeness of long-standing illnesses or health disabilities is associated with women, young people, students, densely populated areas and large households.

Table 20: Someone close to you, currently experiencing long-term illness or disability (A9) by socio-demographic

		Yes	No	Don't know
Gender	Male	38	58*	5
	Female	44*	52	4
Age group	16-24	46*	48	7
	25-54	40	56*	4
	55-74	37	59*	4
Level of education completed	Primary or lower secondary education	42	52	6
	Upper secondary education	41	55	4
	Tertiary education	40	56	4
Situation	Employed or self-employed	39	58*	3
	Unemployed	43	51	6
	Student	47*	46	7
	Other not in the labour force	42	54	4
Type of locality	Densely-populated area	43*	53	4
	Intermediate area	40	56	4
	Thinly-populated area	38	57	4
Members in the household	1	37	58*	5
	2	40	56*	4
	3	43*	53	4
	4+	42*	53	5

Base: Whole sample.

On a per-country basis, this closeness is very clear in Denmark (47.2%), Estonia (50.1%), Finland (47.8%), Sweden (48.1%), Slovenia (54.5%) and Slovakia (49.0%).

Figure 32: Someone close to you currently experience long-term illness of disability (A9) by country

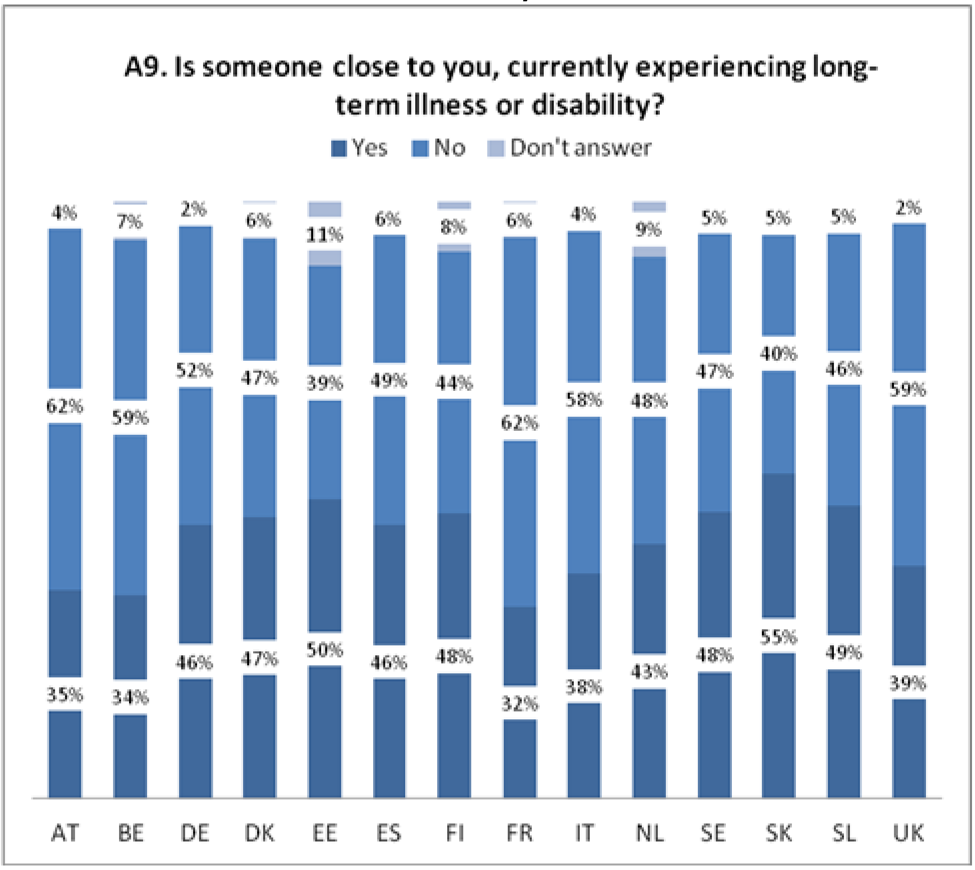

Base: Whole sample.

In the same way, around a third of the sampled European population (32%) take care of someone experience long-term illness or disability.

Figure 33: Taking care of a person experience long-term illness or disability (A10)

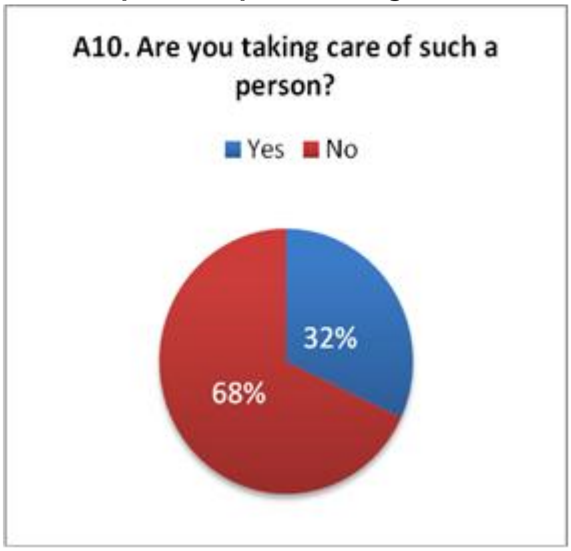

Base: A10 = Yes - 49% of whole sample.

The characterisation of those persons who take care of others, show us that this dependence situation is linked with the older population (38% of citizens between 55 and 74 years old care for another person) and inactivity (36%).

Table 21: Taking care of a person experience long-term illness or disability (A10) by socio-demographic

		Yes	No
Gender	Male	31	69
	Female	33	67
Age group	16-24	28	72
	25-54	32	68
	55-74	38*	62
Level of education completed	Primary or lower secondary education	28	72*
	Upper secondary education	33	67
	Tertiary education	33	67
Situation	Employed or self-employed	32	68
	Unemployed	33	67
	Student	26	74*
	Other not in the labour force	36*	64
Type of locality	Densely-populated area	33	67
	Intermediate area	32	68
	Thinly-populated area	29	71
Members in the household	1	12	88*
	2	31	69
	3	33	67
	4+	40*	60

Base: A10 = Yes, 49% of whole sample.

On a per-country basis, caring for a person stands out strongly in Estonia (52% of the total) and Italy (68%).

Figure 34: Taking care of a person experience long-term illness or disability (A10) by country

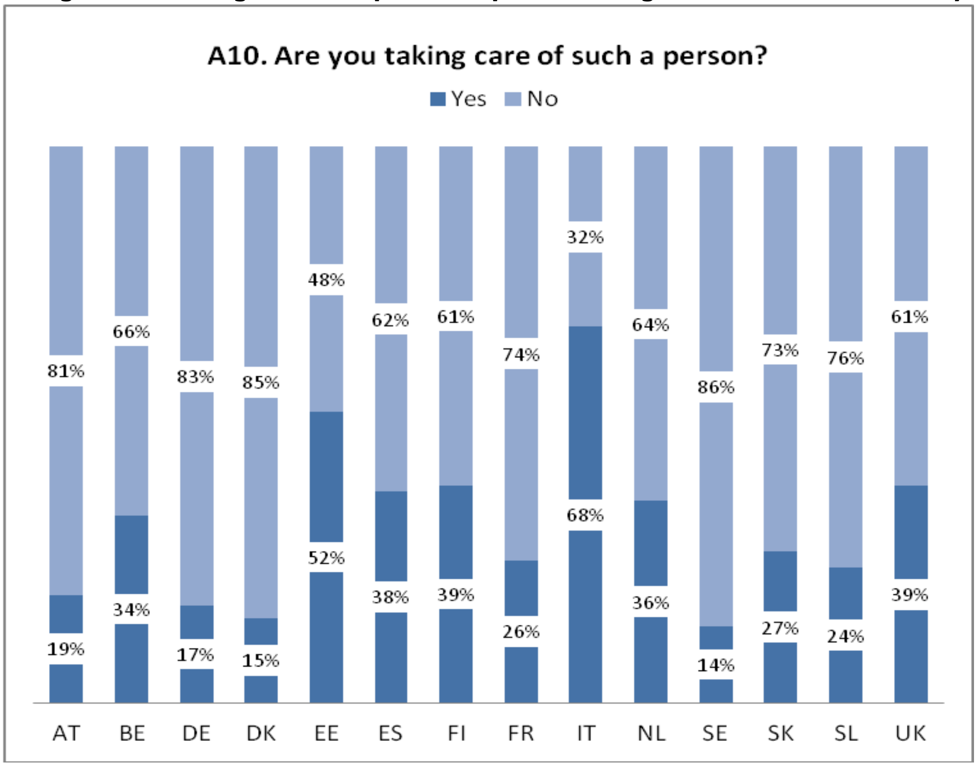

Base: A10 = Yes 49% of whole sample

4.3 Health and social care demand

On average, the sampled population has been seen by a doctor 5.2 times during the previous twelve months, a doctor or nurse has provided home care, 0.65 times; and a social worker, 0.48 times.

Figure 35: Health and social care demand (A1, A2, A3)

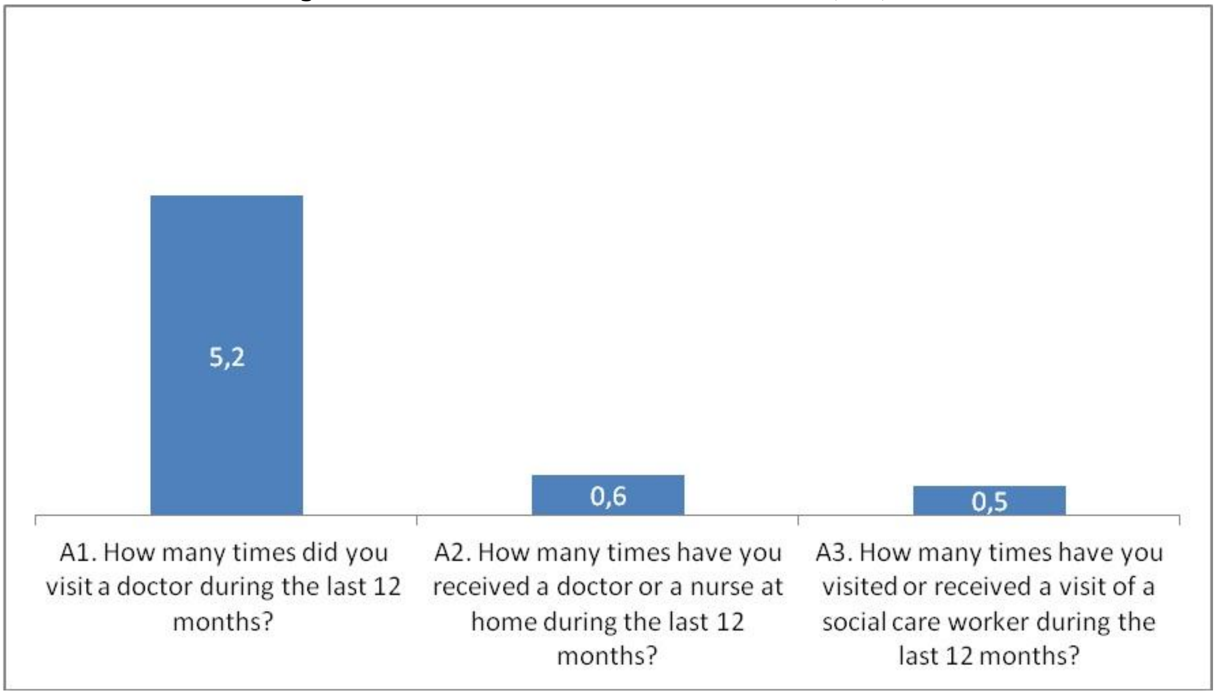

Base: Whole sample.

With respect to the sample populations' socio-demographic characteristics, it is worth mentioning the higher levels of medical care received by women (5.9 visits to the doctor during the last twelve months); the older population (6.12); the less educated population (5.6 visits among citizens with primary education); the inactive population (7.29); the densely populated areas (5.26); the citizens in a poor state of health (13.9 visits to the doctor by the section of the population in poor health); and the citizens with long-standing illnesses (7.89).

On a per-country basis, the higher levels of medical care in Denmark (around an average of 2 visits to the doctor in the last twelve months), Holland (1.45), Belgium (an average of 2.7 home medical visits) and France (1.71) stand out. Conversely, the lower levels of care occur in Estonia, Slovenia and Slovakia.

Table 22: Health and social care demand (A1, A2, A3) by socio-demographic

		Average - visit a doctor during the last 12 months	Average - received a doctor or a nurse at home	Average visit or received a visit of a social care worker
Gender	Male	4,54	,59	,53
	Female	5,90	,71	,41
Age group	16-24	4,03	,52	,32
	25-54	5,31	,60	,52
	55-74	6,12	,95	,49
Level of education completed	Primary or lower secondary education	5,64	,83	,51
	Upper secondary education	5,23	,56	,55
	Tertiary education	5,00	,68	,37
Situation	Employed or self-employed	4,79	,53	,31
	Unemployed	5,47	,53	,76
	Student	4,12	,37	,37
	Other not in the labour force	7,29	1,32	,95
Type of locality	Densely-populated area	5,26	,51	,47
	Intermediate area	5,20	,73	,42
	Thinly-populated area	5,09	,74	,59
Members in the household	1	5,36	,86	,95
	2	5,47	,50	,28
	3	5,12	,64	,37
	4+	4,91	,68	,50
Health status	Bad	13,92	1,83	1,55
	Neither good or bad	7,36	1,16	,88
	Good	3,81	,40	,27
Long standing illness	Yes	7,89	,99	,74
	No	3,31	,39	,30

Base: Whole sample.

4.4 Quality of care

With respect to the specific uses of medical services undertaken by doctors or nurses on the sample population, it is worth indicating that:

- 61% of participants state that they always or very frequently have the results of medical exams explained to them;
- 52% state that they always or very frequently have the different treatment options explained to them by the healthcare professionals; and
- 54% state that the healthcare professionals always or very frequently listen to their opinions and take their preferences into account.

Figure 36: In general, how often does your usual source of care (doctor or nurse)... (A11)

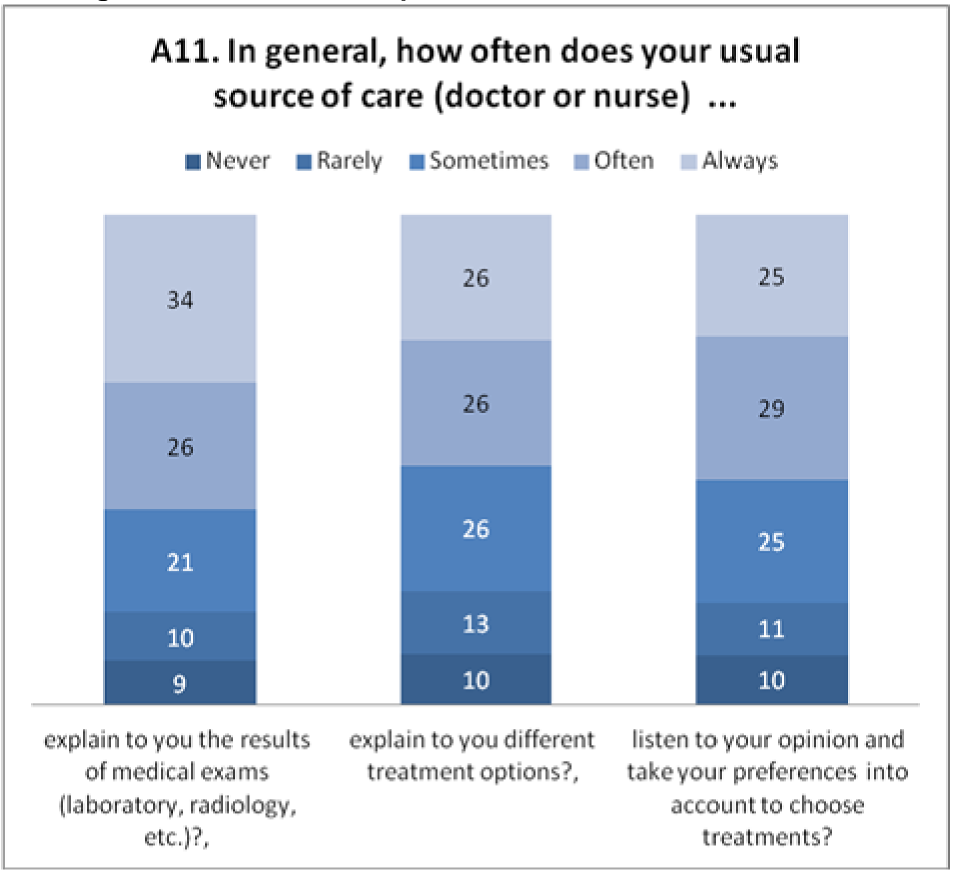

Base: Whole sample.

These favourable opinions about the relationship between the healthcare professional and the patients are statistically linked by some of patient's socio-demographic characteristics. In particular, being older, having a university education, being in self- employed or an entrepreneur, from the more densely populated areas, in poor state of health and having a long-standing illness.

Table 23: In general, how often does your usual source of care (doctor or nurse)... (A11) by socio-demographic

% Often and Always		explain to you the results of medical exams?	explain to you different treatment options?	listen to your opinion and take your preferences into account to choose treatments?
Gender	Male	60	52	53
	Female	60	51	55
Age group	16-24	53	46	51
	25-54	60	51	53
	55-74	69*	60	60
Level of education completed	Primary or lower secondary education	58	49	52
	Upper secondary education	59	51	52
	Tertiary education	62*	53	57*
Situation	Employed or self-employed	60*	51	53
	Unemployed	56	49	54
	Student	55	46	52
	Other not in the labour force	69*	57*	59*
Type of locality	Densely-populated area	59	50	53
	Intermediate area	59	51	54
	Thinly-populated area	63*	53*	56*
Members in the household	1	59	49	53
	2	62*	52	54
	3	60	52	54
	4+	59	52	55
Health status	Bad	66*	54	56*
	Neither good or bad	62	52	54
	Good	59	51	54
Long standing illness	Yes	66*	57*	60*
	No	57	48	50

Base: Whole sample.

On a per-country basis, the perception of service quality with respect to the patient-healthcare professional relationship is strongly evident in Belgium, Denmark, Spain and France.

Figure 37: In general, how often does your usual source of care (doctor or nurse) explain to you the results of medical exams (A11) by country

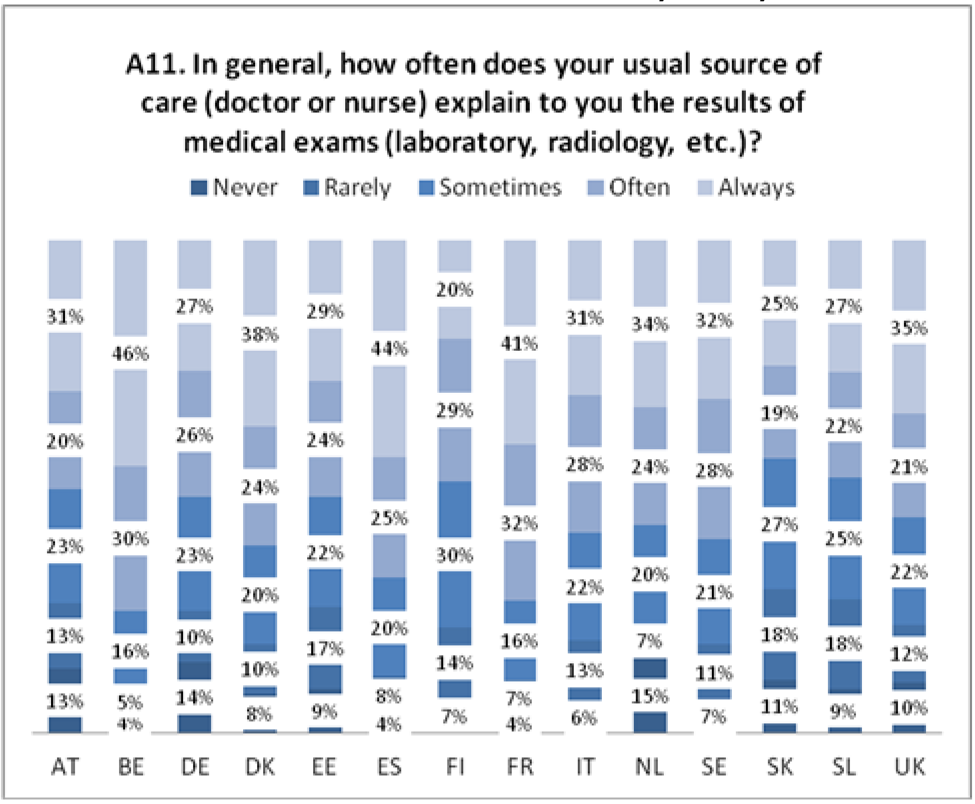

Base: Whole sample.

Figure 38: In general, how often does your usual source of care (doctor or nurse) explain to you different treatment options (A11) by country

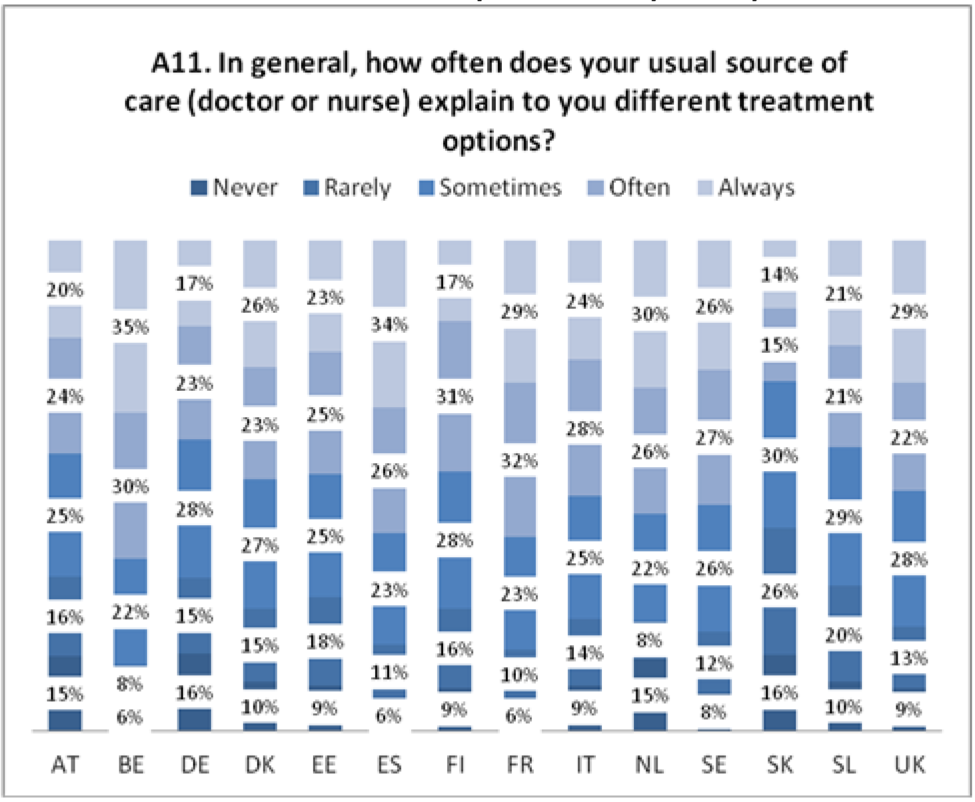

Base: Whole sample.

Figure 39: In general, how often does your usual source of care (doctor or nurse) listen to your opinion and take your preferences into account to choose treatments (A11) by country

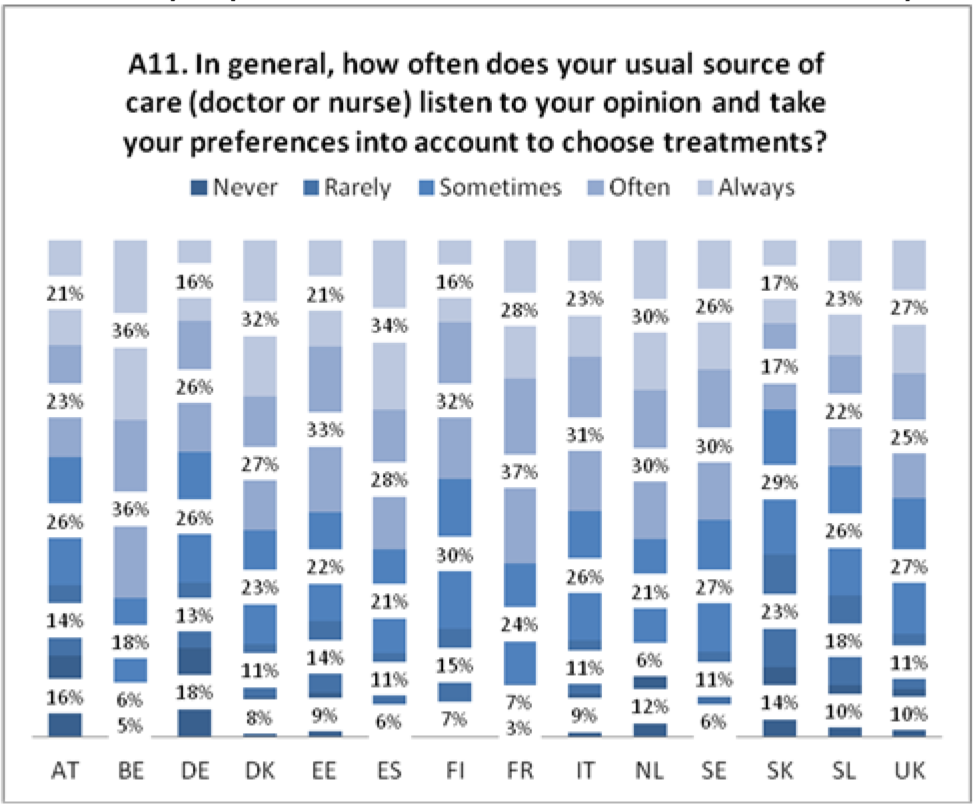

Base: Whole sample.

Individuals were also questioned on how often they ask their usual source of care (doctor or nurse) about their results of medical exams; the different treatment options and to consider their opinions.

- 59%% of participants state that they always or very frequently asked have about the results of medical exams;
- 52% state that they always or very frequently asked about the different treatment options; and
- 50% state they always or very frequently asked health professional to consider their opinions

Figure 40: In general, how often do you ask your usual source of care (doctor or nurse)...(A12)

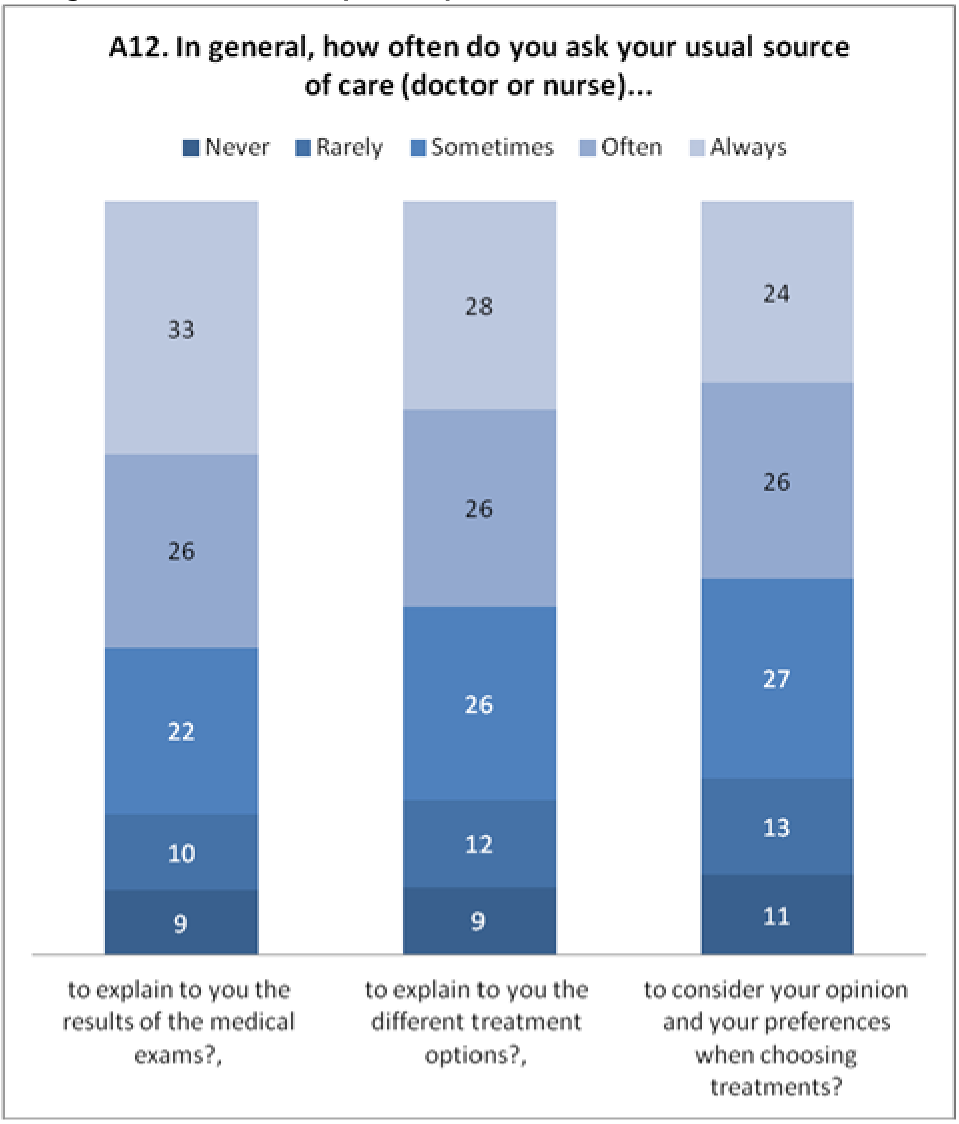

Base: Whole sample.

These favourable opinions about the relationship between the healthcare professional and the patients are statistically linked by some of patient's socio-demographic characteristics. In particular, being middle age, having a university education, being in a poor state of health and having a long-standing illness.

Table 24: In general, how often do you ask your usual source of care (doctor or nurse)... (A12) by socio-demographic

% Often and Always		to explain to you the results of the medical exams?	to explain to you the different treatment options?	to consider your opinion and your preferences when choosing treatments?
Gender	Male	58	52	48
	Female	60*	56*	52*
Age group	16-24	52	48	45
	25-54	60*	54*	51*
	55-74	66*	59*	54*
Level of education completed	Primary or lower secondary education	55	51	45
	Upper secondary education	59	53*	48
	Tertiary education	62*	56*	54*
Situation	Employed or self-employed	59*	53*	50
	Unemployed	59*	55*	51
	Student	52	48	44
	Other not in the labour force	66*	60*	55*
Type of locality	Densely-populated area	60	55*	51
	Intermediate area	59	53	49
	Thinly-populated area	60	54	50
Members in the household	1	58	52	49
	2	61	54	49
	3	60	54	51
	4+	58	54	51
Health status	Bad	65*	58*	55*
	Neither good or bad	62*	58*	53*
	Good	58	52	49
Long standing illness	Yes	65*	60*	56*
	No	56	50	46

Base: Whole sample.

Figure 41: In general, how often do you ask your usual source of care (doctor or nurse) to explain to you the results of the medical exams? (A12) by country

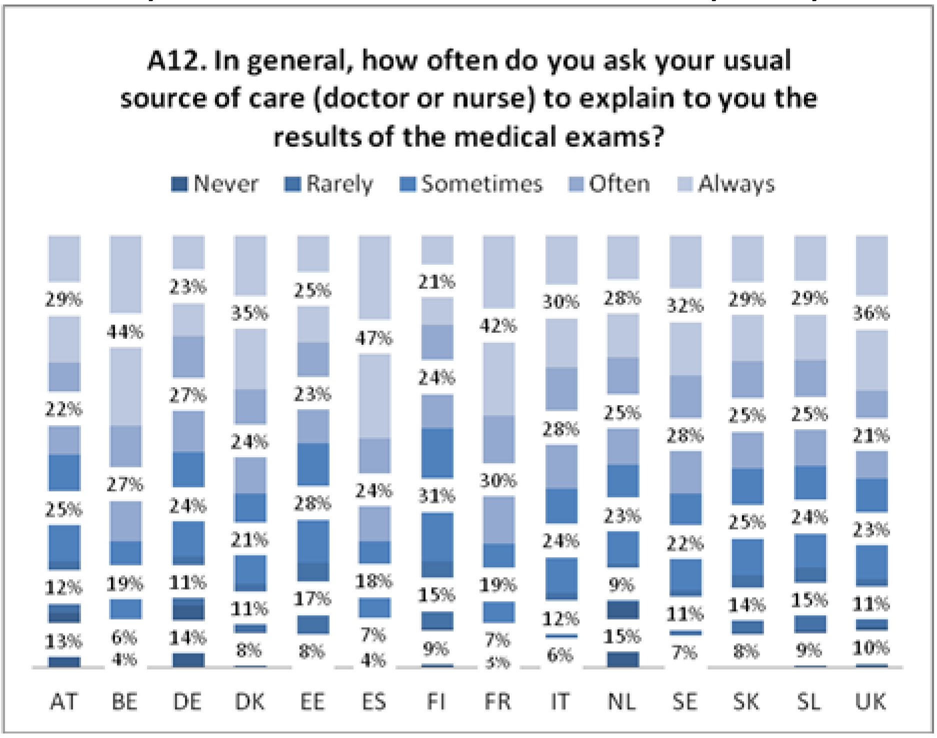

Base: Whole sample.

Figure 42: In general, how often do you ask your usual source of care (doctor or nurse) to explain to you the different treatment options? (A12), by country

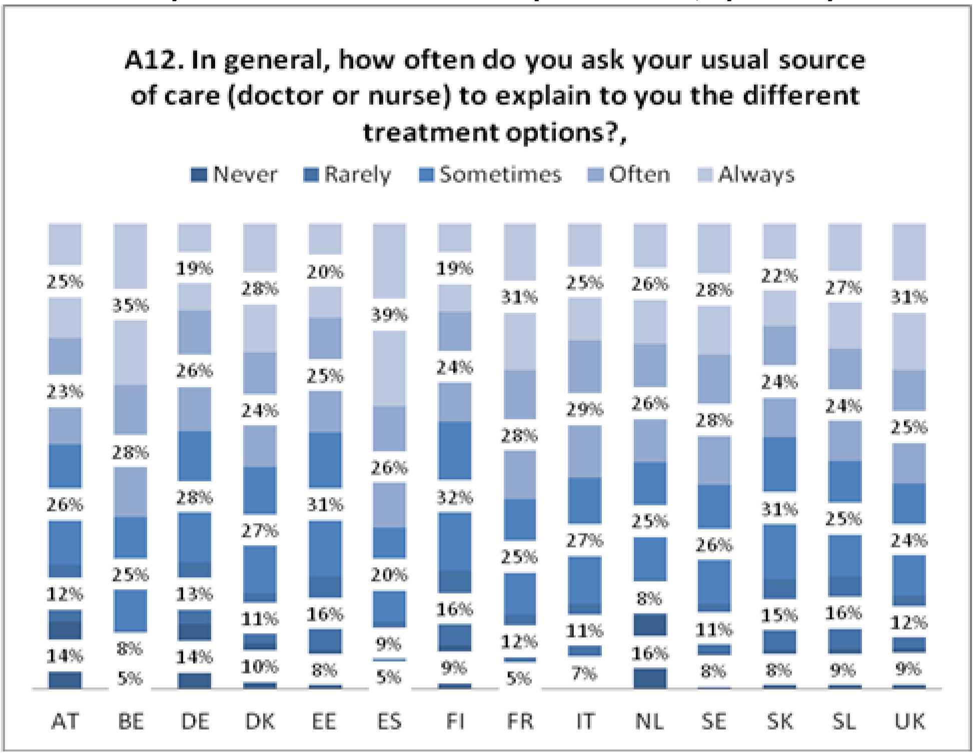

Base: Whole sample.

Figure 43: In general, how often do you ask your usual source of care (doctor or nurse) to consider your opinion and your preferences when choosing treatments? (A12), by country

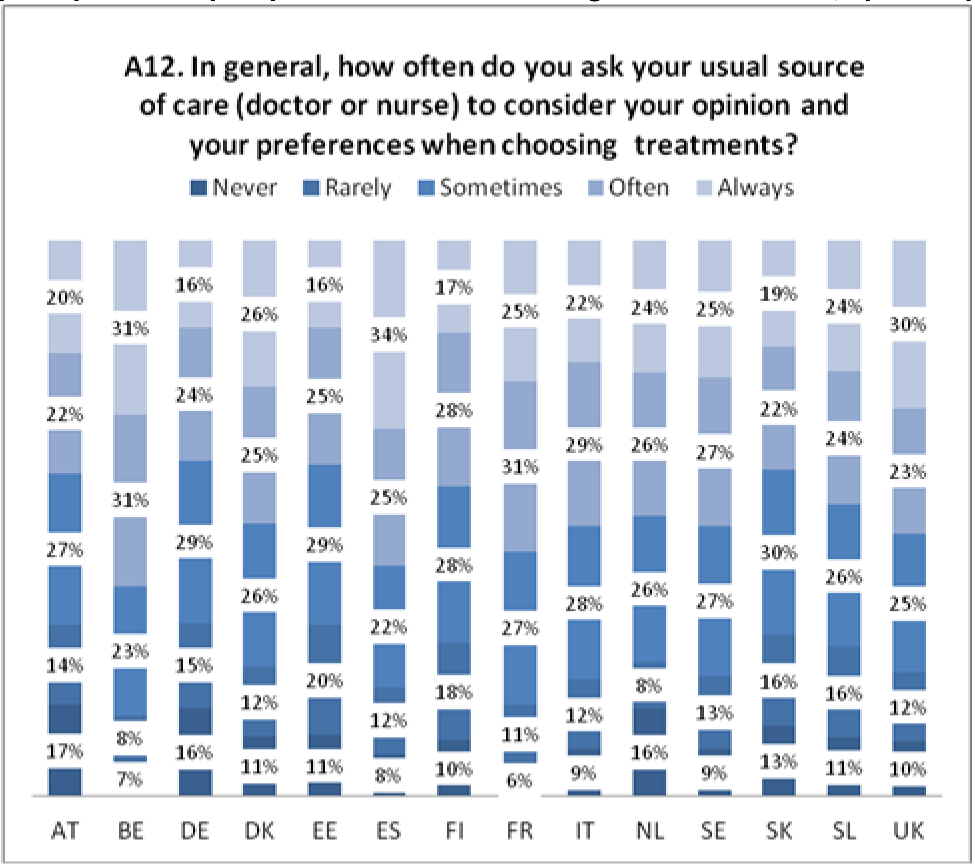

Base: Whole sample.

5. ICT ACCESS

5.1 Internet access and frequency of use

The use of the Internet and Information and Communication Technologies (ICT) are key for the advancement of the new uses in healthcare. This study also provides relevant information in this aspect. 93% of the surveyed population uses the Internet at home at least once a day, 42% do so at work, and 14% at least once a day in other locations.

Figure 44: Internet access and use (C1)

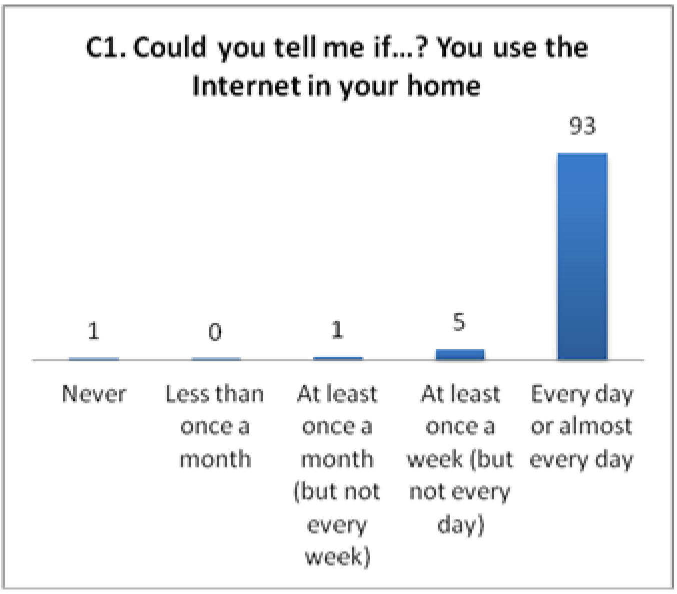

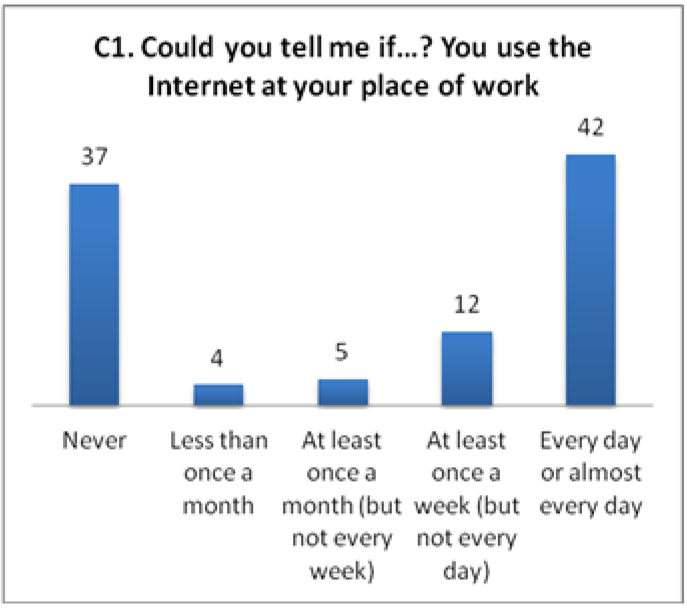

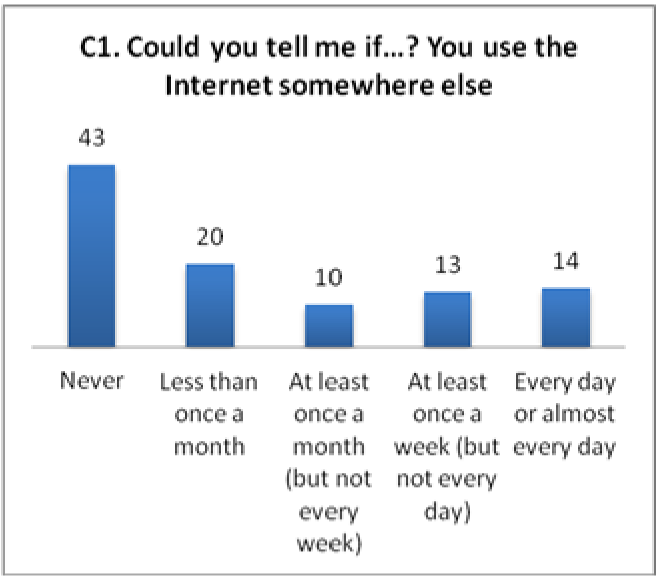

Base: Whole sample.

In terms of socio-demographic characteristics, the intensive uses of ICTs are statistically linked with men (64.4% of men use the Internet at least once a day at work), the youngest age group (70.7% of the sample population between 16 and 24 years old connects to the Internet away from the home and at work), a university education (73.2% of the participants with a university education use the Internet at work at least once a day), the student population and the population density.

Table 25: Internet access and use (C1) by socio-demographics

At least once a month (%)		You use the Internet in your home	You use the Internet at your place of work	You use the Internet somewhere else
Gender	Male	99	64*	44*
	Female	99	54	30
Age group	16-24	99	63*	71*
	25-54	99	66*	33
	55-74	100*	33	14
Level of education completed	Primary or lower secondary education	99	38	28
	Upper secondary education	99	54*	34
	Tertiary education	99	73	44*
Situation	Employed or self-employed	99	77*	34
	Unemployed	97	29	30
	Student	99	62	83*
	Other not in the labour force	100*	15	14
Type of locality	Densely-populated area	99	65*	44*
	Intermediate area	99	57	35
	Thinly-populated area	99	52	27
Members in the household	1	98	55	34
	2	99*	56	29
	3	99	63*	37
	4+	99	61	46*
Health status	Bad	99	36	25
	Neither good or bad	99*	50	31
	Good	99	64*	40*
Long standing illness	Yes	99*	52	30
	No	99	64*	42*

Base: Whole sample.

On a per-country basis and looking at Internet use, it is worth indicating the intensity of use whilst at work in Estonia (62%), Sweden (56%) and Slovenia (55%).

Table 26: Internet access and use at home (C1) by country

(%)	AT	BE	DE	DK	EE	ES	FI	FR	IT	NL	SE	SK	SL	UK
Never	1	1	0	0	0	1	1	0	1	2	1	0	0	1
Less than once a month	0	1	0	1	1	0	1	0	0	1	0	0	0	0
At least once a month (but not every week)	1	2	1	2	1	2	1	1	2	2	1	0	0	1
At least once a week (but not every day)	8	6	3	5	6	4	6	5	7	5	5	4	3	4
Every day or almost every day	90	91	95	92	92	92	92	94	89	89	93	95	96	95

Base: Whole sample.

Table 27: Internet access and use at work (C1) by country

(%)	AT	BE	DE	DK	EE	ES	FI	FR	IT	NL	SE	SK	SL	UK
Never	32	44	33	40	20	32	30	39	31	54	26	32	28	47
Less than once a month	4	4	4	4	5	3	7	4	3	4	2	5	4	3
At least once a month (but not every week)	4	4	6	6	3	5	5	5	4	4	4	4	3	2
At least once a week (but not every day)	12	12	15	10	10	12	13	11	14	10	13	13	10	11
Every day or almost every day	49	36	43	41	62	47	46	41	49	28	55	46	55	37

Base: Whole sample.

Table 28: Internet access and use somewhere else (C1) by country

(%)	AT	BE	DE	DK	EE	ES	FI	FR	IT	NL	SE	SK	SL	UK
Never	43	51	39	43	27	33	32	49	40	57	40	38	33	50
Less than once a month	22	19	23	20	26	19	31	18	14	13	24	22	23	21
At least once a month (but not every week)	12	8	12	8	12	13	11	9	10	7	9	11	12	8
At least once a week (but not every day)	12	12	15	10	12	15	12	12	15	11	13	14	12	12
Every day or almost every day	11	10	12	20	23	20	14	12	21	12	15	16	20	10

Base: Whole sample.

5.2 Internet-related activities

With respect to Internet based activities, the sampled population mainly uses it to search for information (67.6% every day), sending e-mails with attachments (40.6%), online banking (20.3%), social networks (38.6%) and instant messaging (22.8%).

Figure 45: Internet activities (C2)

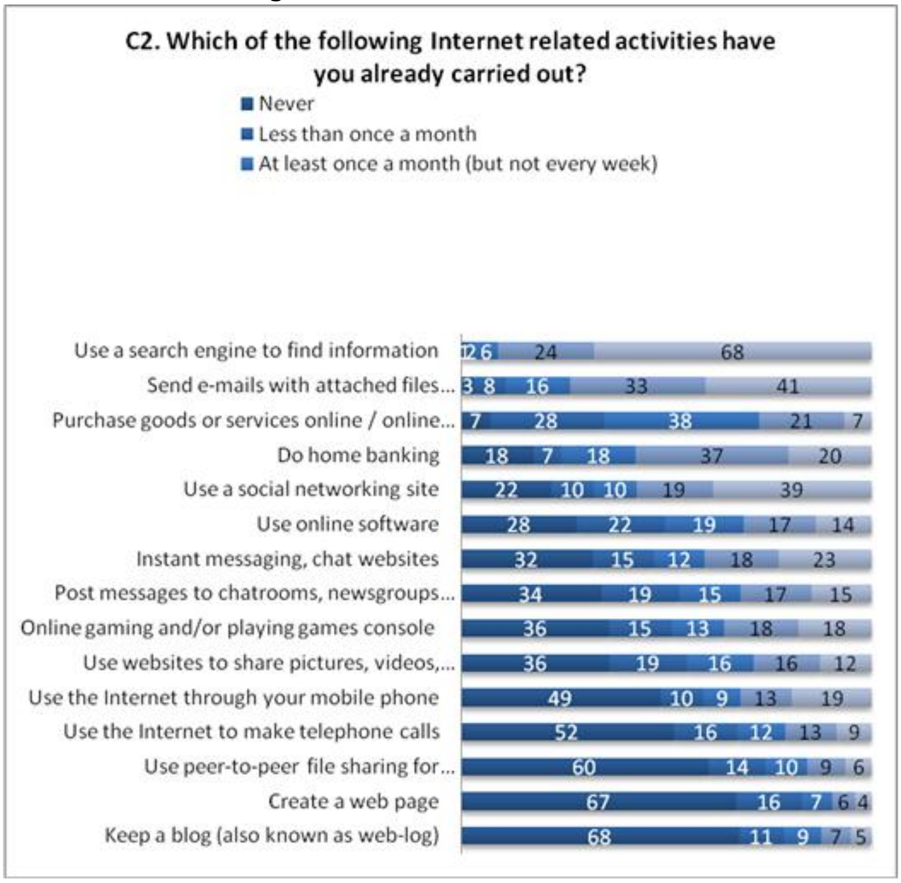

Base: Whole sample.

As with the general situation, the main uses of the Internet are linked with the male gender, the youngest age groups, a university education, self-employment and entrepreneurs, studying, population density and a good state of health.

To be fully transparent a comparison between Internet activities performed by individuals in our sample and Internet activities reported by a representative sample of EU27 population was carried out (see Annex 4. Internet activities **comparison**). The results of this comparison reveals that our sample is composed by slightly advance Internet users: the less the diffusion of the Internet by country the higher the differences.

Table 29: Internet activities (C2) by socio-demographics

At least once a month (%)		Use a search engine to find information	Send e-mails with attached files	Post messages to chatrooms, newsgroups or an online discussion forum	Use the Internet to make telephone calls	Use peer-to-peer file sharing for exchanging movies, music, etc	Create a web page	Use websites to share pictures, videos, movies, etc.	Use a social networking site
Gender	Male	97*	90*	50*	38*	32*	22*	47*	68
	Female	97	88	43	28	19	12	43	68
Age group	16-24	96	88	63*	41*	41*	25*	68*	88*
	25-54	97*	90*	47	31	25*	17*	43	68*
	55-74	96	87	29	30	11	10	27	46
Level of education completed	Primary or lower secondary education	95	79	45	27	22	16	41	63
	Upper secondary education	96	88	47	31	26*	16	45	67
	Tertiary education	98*	95*	48	37*	27*	19*	47*	71*
Situation	Employed or self-employed	98*	92*	46	34*	26	19*	44	67
	Unemployed	96	85	51	28	28	15	45	72
	Student	96	88	62*	40*	39*	22*	67*	88*
	Other not in the labour force	96	84	35	26	12	10	31	51
Type of locality	Densely-populated area	97	91	51*	36*	30*	20*	50*	71*
	Intermediate area	97	88	47	32	25	17	44	69*
	Thinly-populated area	97	88	39	28	19	13	38	60
Health status	Bad	96	85	46	28	17	13	38	62
	Neither good or bad	97	86	47	31	23	15	41	63
	Good	97	90*	47	34*	27*	18*	47*	70*
Long standing illness	Yes	97*	89	46	32	23	15	42	65
	No	97	89	47	33*	28*	18*	47*	70*

Base: Whole sample.

Table 30: Internet activities (C2) by socio-demographics

At least once a month (%)		Purchase goods or services online / online shopping	Keep a blog	Instant messaging, chat websites	Do home banking	Use online software	Use the Internet through your mobile phone	Online gaming and/or playing games console
Gender	Male	68*	24*	55*	79*	57*	47*	51*
	Female	63	19	51	72	44	35	46
Age group	16-24	66	34*	77*	66	61*	61*	65*
	25-54	67*	20	51*	78*	49	41	49
	55-74	58	13	35	77	42	19	30
Level of education completed	Primary or lower secondary education	57	20	50	70	47	34	53*
	Upper secondary education	65	22	53	74	48	39	51
	Tertiary education	69*	22	54	80*	54*	46*	45
Situation	Employed or self-employed	70*	20	51	81*	51	45	48
	Unemployed	51	21	58	64	44	34	55
	Student	65*	33*	76*	63	61*	56*	62*
	Other not in the labour force	59	14	39	73	41	21	38
Type of locality	Densely-populated area	66	23*	57*	76	55*	47	51
	Intermediate area	64	22	53	75	49	41	50
	Thinly-populated area	67	16	47	76	45	31	44
Health status	Bad	70*	18	49	77	45	31	45
	Neither good or bad	63	20	50	76	48	36	49
	Good	66	22	54	76	51*	43*	49
Long standing illness	Yes	66	19	49	77	49	36	46
	No	65	23*	56*	75	51	45*	50*

Base: Whole sample.

Table 31: Internet activities (C2) by country

At least once a month (%)	AT	BE	DE	DK	EE	ES	FI	FR	IT	NL	SE	SK	SL	UK
Use a search engine to find information	98	96	97	95	97	98	97	97	96	93	98	99	99	98
Send e-mails with attached files (documents, pictures, etc.)	92	89	89	86	94	93	80	91	90	82	89	94	97	88
Post messages to chatrooms, newsgroups or an online discussion forum	46	34	50	43	47	63	41	38	54	36	38	57	65	46
Use the Internet to make telephone calls	35	28	35	28	31	33	22	33	45	29	33	48	38	24
Use peer-to-peer file sharing for exchanging movies, music, etc	20	25	18	22	36	40	25	22	41	28	19	32	50	22
Create a web page	17	11	19	17	14	23	11	14	25	17	14	21	20	12
Use websites to share pictures, videos, movies, etc.	48	46	40	44	60	60	40	41	53	47	41	61	55	40
Use a social networking site	63	65	68	68	76	80	47	63	72	60	64	77	75	67
Purchase goods or services online / online shopping (e.g. travel & holiday, clothes, books, tickets, films, music, software, food)	71	43	76	62	42	54	55	61	55	54	60	55	51	80
Keep a blog (also known as web-log)	18	16	23	16	16	33	14	17	29	20	23	16	16	15
Instant messaging, chat websites	50	50	52	45	55	66	48	57	64	42	52	72	44	41
Do home banking	79	84	75	85	95	68	93	76	62	85	92	73	66	80
Use online software	51	44	52	49	68	54	50	43	55	48	52	56	59	51
Use the Internet through your mobile phone	42	24	38	40	33	46	42	39	40	38	49	36	53	48
Online gaming and/or playing games console	46	54	49	49	42	53	44	47	52	66	36	44	29	44

Base: Whole sample.

6. HEALTH INFORMATION SOURCES AND TRUST

Despite the importance of the Internet as an empowering tool for health, and with respect to the set of available information sources, the surveyed citizens continue to consider direct interaction with doctors (75%) and nurses (40%) to be most relevant. In turn, the growth of the Internet as a channel for health interaction stands out, if it is taken into account that its relevance (35%) is already greater than that of pharmacies (32%).

Figure 46: Health information sources (B4)

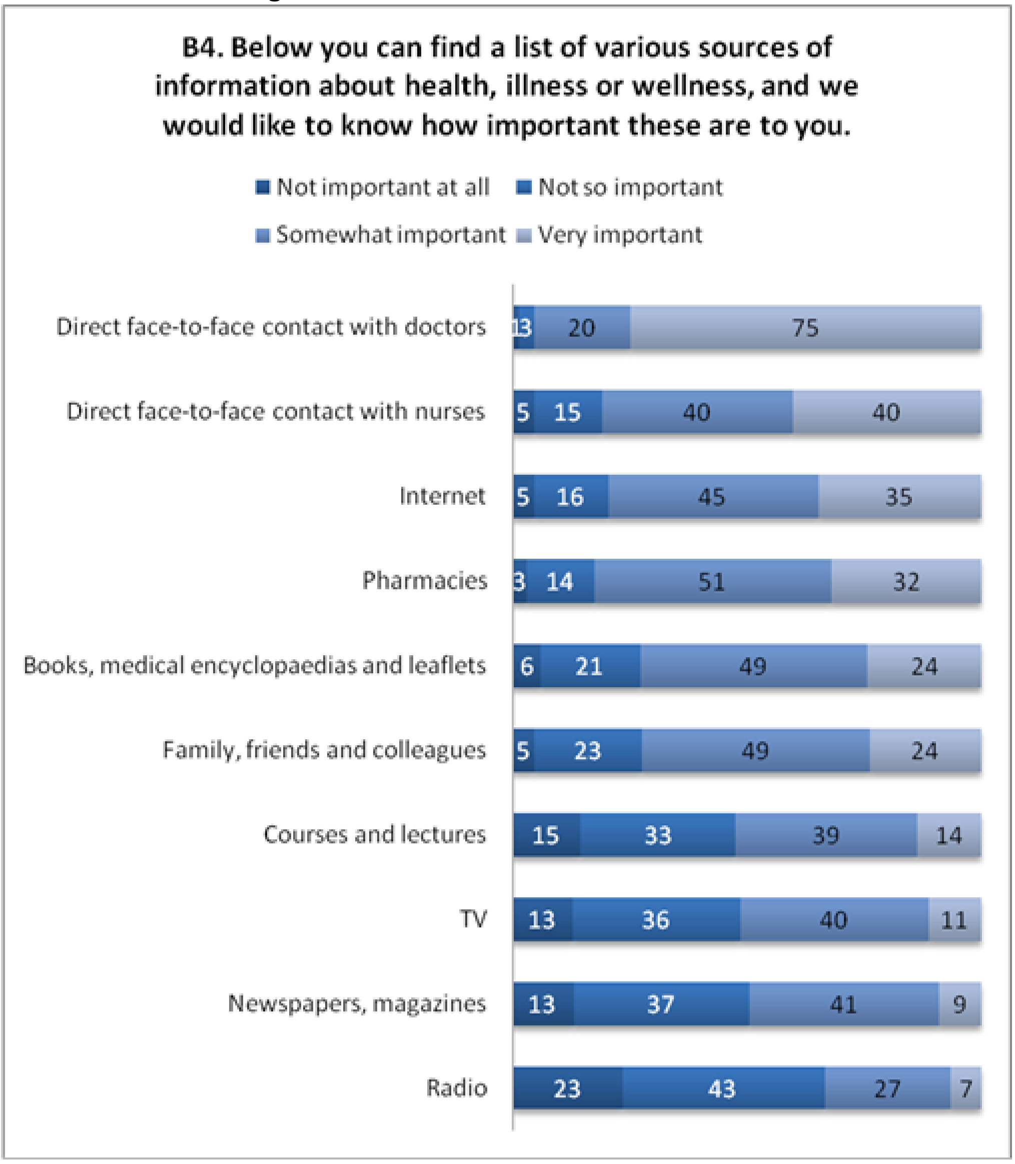

Base: Whole sample.

The perceived importance of the Internet as a main channel for health interaction is linked with women (81%), the middle population set (81% of people aged between 25 and 54 years old), the worst states of health, and the existence of long-standing illness (82%).

Table 32: Health information sources (B4) by socio-demographics

Important (%)		Internet	TV	Radio	Books, medical encyclopaedias and leaflets	Courses and lectures	Newspapers magazines	Family, friends and colleagues	Pharmacies	Direct face-to-face contact with doctors	Direct face-to-face contact with nurses
Gender	Male	78	49	34	70	50	47	68	80	95	79
	Female	81*	54*	33	76*	55*	52*	77*	85*	96*	83*
Age group	16-24	80	53	32	74	57*	52*	80*	85	93	81
	25-54	81*	52	34*	75*	52	50	74*	83	96	81
	55-74	76	46	33	65	48	46	60	80	98*	82
Level of education completed	Primary or lower secondary education	79	60	39*	68	52	51	77*	85*	95	82
	Upper secondary education	81	53	34	73	52	49	73	83	95	82
	Tertiary education	79	47	31	74*	53	50	70	81	96	79
Situation	Employed or self-employed	80	51	35*	74*	53	50	73	82	96	81
	Unemployed	81	58	34	74	52	49	75	84	96	83
	Student	79	51	28	75*	56*	53*	80*	84	93	77
	Other not in the labour force	78	49	32	68	48	46	64	83	97	83
Type of locality	Densely-populated area	81*	52	35*	73	54	52*	74*	82	96	80
	Intermediate area	80	53	32	74	51	48	72	83	95	82*
	Thinly-populated area	77	49	33	71	51	47	71	83	96	80
Health status	Bad	85*	47	26	69	51	40	67	78	97	79
	Neither good or bad	81*	55*	34	72	52	50	71	83	96*	81
	Good	79	51	34*	74	53	51*	73	83	95	81
Long standing illness	Yes	82*	51	32	72	52	48	70	82	97*	81
	No	78	52*	35*	74*	53	51	74	83	94	81

Base: Whole sample.

Estonia (87%), Slovakia (94%), Slovenia (93%) and the United Kingdom (89%) lead the Internet as source of health information.

Table 33: Health information sources (B4) by country

Important (%)	AT	BE	DE	DK	EE	ES	FI	FR	IT	NL	SE	SK	SL	UK
Internet	84	67	85	75	87	74	80	65	84	80	75	94	93	89
TV	49	46	53	38	66	50	44	48	57	56	35	64	59	54
Radio	32	29	31	19	50	40	27	37	40	35	23	44	32	29
Books, medical encyclopaedias and leaflets	74	74	74	52	77	77	57	71	76	69	61	76	77	75
Courses and lectures	54	61	52	28	68	62	35	57	67	48	36	48	58	39
Newspapers, magazines	53	44	55	27	66	52	44	48	60	48	31	52	46	43
Family, friends and colleagues	80	65	80	68	87	72	80	62	70	74	77	76	74	73
Pharmacies	82	89	76	66	88	89	80	88	82	89	79	81	74	83
Direct face-to-face contact with doctors	93	97	94	93	97	96	94	97	96	95	94	97	92	97
Direct face-to-face contact with nurses	67	87	62	83	86	92	87	87	78	87	87	82	78	92

Base: Whole sample.

Medical and health institutions continue to lead in terms of perceived trust with respect to the health information available to European citizens. 26% of participants fully trust medical and health institutions, with 55% trusting them somewhat. Something similar, although not as marked, occurs with the national health authorities. When it comes to online companies, the percentage of trust is very much lower. 4% of the European population trusts them fully, whilst a third trust them somewhat.

Figure 47: Trust (B5)

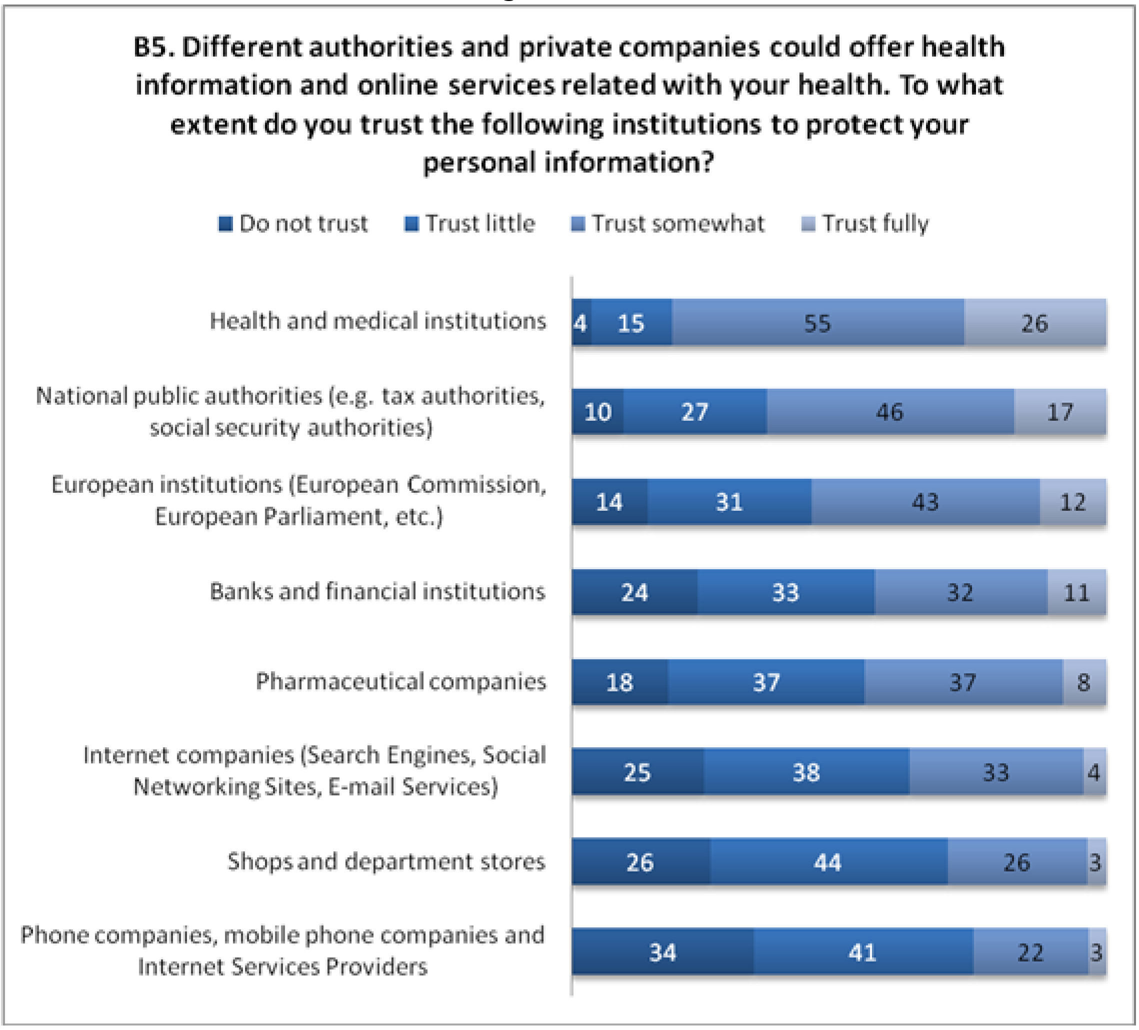

Base: Whole sample.

The perceived importance of the Internet as a main channel for health interaction is linked with women (81.3%), the middle population set (80.7% of people aged between 25 and 54 years old), the worst states of health, and the existence of long-standing illness (81.8%).

**Table 34: Trust (B5) by socio-demographics**<br>**Trust (%)**		National public authorities	European institutions	Banks and financial institutions	Health and medical institutions	Shops and department stores	Internet companies	Phone and mobile phone companies and ISP	Pharmaceutical companies
Gender	Male	63	55	41	81	30	36	26*	42
	Female	64	55	46*	82	30	38	24	49*
Age group	16-24	70*	68*	54*	84*	37*	37	29*	57*
	25-54	64	55	42	81	30	37	25	44
	55-74	55	41	35	78	23	36	20	37
Level of education complete d	Primary or lower secondary education	63	50	47	81	35*	42*	30*	50*
	Upper secondary education	63	53	44	82*	31	38	26	47
	Tertiary education	65*	59*	41	80	26	33	22	42
Situation	Employed or self-employed	64	55	43	81	30	38	25	44
	Unemployed	60	49	42	79	28	37	25	49
	Student	72*	70*	52*	85*	34	33	27	52*
	Other not in the labour force	57	44	39	79	25	36	23	41
Type of locality	Densely-populated area	66*	58*	45*	82	29	36	25	45
	Intermediate area	62	55	43	81	32*	38	26	47*
	Thinly-populated area	62	51	42	80	28	35	22	44
Health status	Bad	56	46	36	78	24	34	20	37
	Neither good or bad	57	46	38	80	28	40	24	43
	Good	66*	58*	45*	82*	31*	36	25*	46*
Long standing illness	Yes	61	51	40	81	27	37	23	42
	No	66*	59*	46*	82	32*	37	26*	48*

Base: Whole sample.

On a per-country basis, trust in Internet-based health is led by Denmark (43% of all participants), Italy (48%), Holland (41%) and the United Kingdom (40%).

Table 35: Trust (B5) by country

Trust (%)	AT	BE	DE	DK	EE	ES	FI	FR	IT	NL	SE	SK	SL	UK
National public authorities (e.g. tax authorities, social security authorities)	70	60	64	76	69	65	71	65	64	58	73	56	57	59
European institutions	52	57	57	56	59	57	49	60	70	43	49	56	58	42
Banks and financial institutions	56	43	47	68	67	38	74	33	37	48	60	61	54	43
Health and medical institutions	85	87	82	85	80	77	80	84	87	82	78	70	78	76
Shops and department stores	25	29	29	33	24	25	28	28	31	31	18	20	24	38
Internet companies	32	36	33	43	35	29	23	39	48	41	24	30	35	40
Phone companies, mobile phone companies and Internet Services Providers	26	22	22	38	34	18	30	23	27	23	20	33	28	31
Pharmaceutical companies	37	56	35	51	53	52	45	46	47	41	41	39	47	54

Base: Whole sample.

7. ICT FOR HEALTH: MOTIVATIONS AND BARRIERS

7.1 Triggers

Beyond specific uses of ICTs in the health sector, the research has also captured the thoughts of European citizens with respect to facilitators and barriers. With respect to the factors that motivate the use of ICTs in health, more than a third of the sampled European population indicates a significant use of ICTs in health to better understand a health problem or disease (39.2%), to find additional sources of information (36.1%) and to develop knowledge and personal satisfaction (34.7%). A little further behind, but still with a relevant frequency, there is the perception that ICTs in health are very useful to help a family member or a friend who is ill (30.7%), to prevent illnesses or to adopt a more healthy lifestyle (28.4%), to find a solution to or a treatment for a health problem (27.5%), to obtain different points of view about an issue (22.3%), and to access an online health service (20.9%). Finally, and as a counterpoint, only 10.6% of European citizens give much importance to the use of ICTs in health for participating in online discussions.

Figure 48: ICT for Health motivations and triggers (D11)

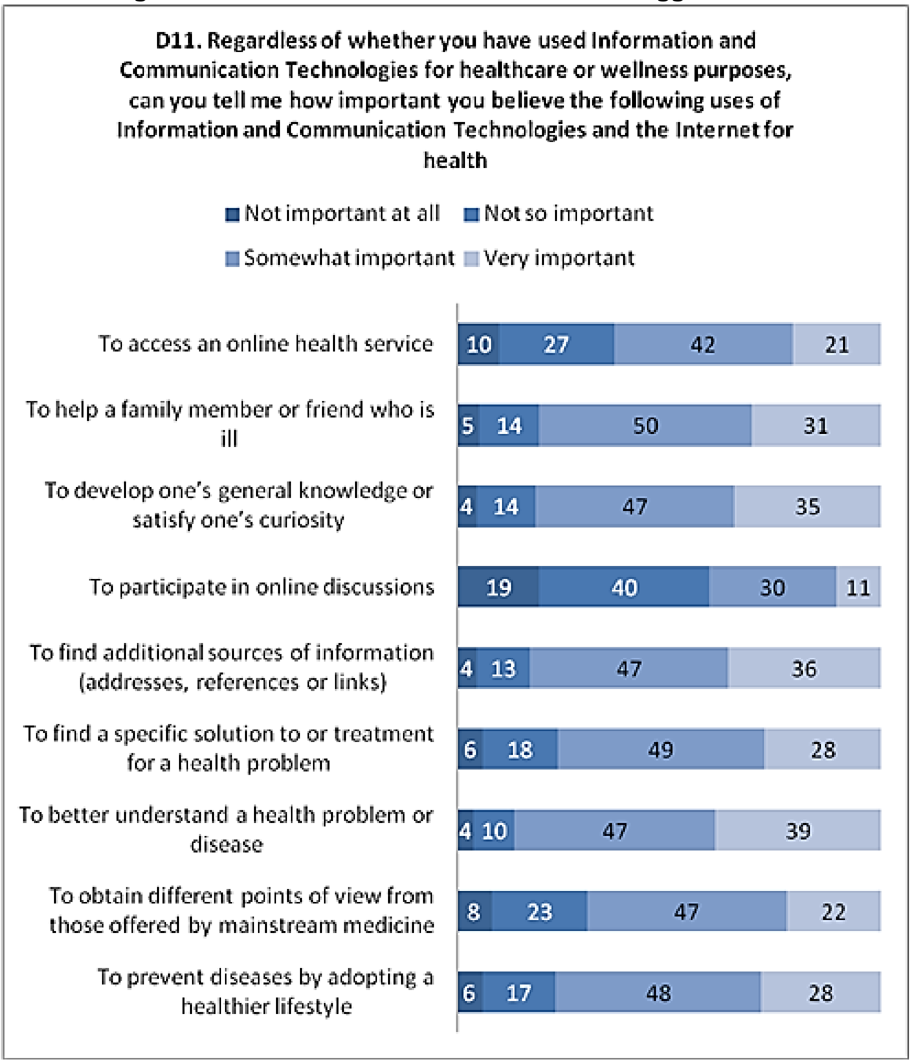

Base: Whole sample.

With respect to the socio-demographic characteristics of the population, the perception of the importance of ICTs in health for the health or wellness sector is much more positive for women, young people, the middle aged, those with a tertiary education, the employed, students, and people in a bad state of health or with long standing illnesses.

Table 36: Information and Communication Technology motivations and triggers (D11) by socio-demographics

Important (%)		To prevent diseases by adopting a healthier lifestyle	To obtain different points of view from those offered by mainstream medicine	To better understand a health problem or disease	To find a specific solution to or treatment for a health problem	To find additional sources of informati on	To participa te in online discussi ons	To develop one's general knowledge or satisfy one's curiosity	To help a family member or friend who is ill	To access an online health service
Gender	Male	75	66	84	74	80	41	80	78	62
	Female	79*	73*	90*	79*	86*	41	84*	84*	64
Age group	16-24	75	70*	84	72	80	52*	80	77	61
	25-54	78*	71*	88*	78*	84*	41*	83*	82*	65*
	55-74									
Level of education complete d	Primary or lower secondary education	75	67	85	77	79	39	79	81	61
	Upper secondary education	77	70*	87	77*	83	41	81	81	63
	Tertiary education	78	69	88*	75	85*	41	84*	80	64*
Situation	Employed or self-employed	77	71*	87	77	84*	41	83*	81	64*
	Unemployed	80*	71*	87	79*	82	42*	81	83*	68*
	Student	75	69	84	71	81	50*	81	75	57
	Other not in the labour force	78	64	88	77	83	31	80	83	61
Health status	Bad	79	72	91*	84*	87*	39	85	87*	66*
	Neither good or bad	79	70	88	78*	83	41	83	83	63
	Good	76	69	86	75	82	41	81	80	63
Long standing illness	Yes	79	71	90*	79*	85*	39	84*	83*	65
	No	75	68	85	74	82	42	80	79	62

Base: Whole sample.

On a per-country basis, the greatest perceptions of the relevance of ICTs for health use are found in Slovakia, Slovenia and the United Kingdom.

Table 37: Information and Communication Technology motivations and triggers (D11) by socio-demographics

Important (%)	AT	BE	DE	DK	EE	ES	FI	FR	IT	NL	SE	SK	SL	UK
To prevent diseases by adopting a healthier lifestyle	74	70	73	67	85	84	74	67	80	72	69	87	92	89
To obtain different points of view from those offered by mainstream medicine	74	52	75	54	74	72	63	60	75	53	57	78	82	76
To better understand a health problem or disease	86	80	87	81	92	87	83	84	88	81	81	91	95	91
To find a specific solution to or treatment for a health problem	79	67	81	68	82	75	68	66	78	72	69	85	91	84
To find additional sources of information (addresses, references or links)	87	74	87	78	93	84	83	76	87	71	74	85	92	87
To participate in online discussions	38	25	41	30	53	45	48	34	54	27	17	48	60	45
To develop one's general knowledge or satisfy one's curiosity	84	76	86	74	89	82	81	80	86	72	74	80	85	82
To help a family member or friend who is ill	79	75	80	72	89	83	74	76	81	80	79	85	92	87
To access an online health service	58	43	57	53	82	78	62	48	80	50	63	70	84	74

Base: Whole sample.

7.2 Empowerment

When it comes to attitudes towards health and health information sources, the research also provides empirical evidence in the case of the sample of European citizens. Overall, the sampled European citizens show they agree that ICTs, especially the Internet, improve their capacity for information and empower them with respect to their state of health. Around two thirds of the sampled citizens show they agree with the fact that the Internet improves their capacity for information and their relationships with other people. In the same manner, they consider that the Internet improves the understanding of the state of health, allows them to be more informed and to have a more proactive role in their relationship with the healthcare professionals, and gives them greater access to expert knowledge through interaction with more people.

Figure 49: Empowerment and ICT for Health (B1)

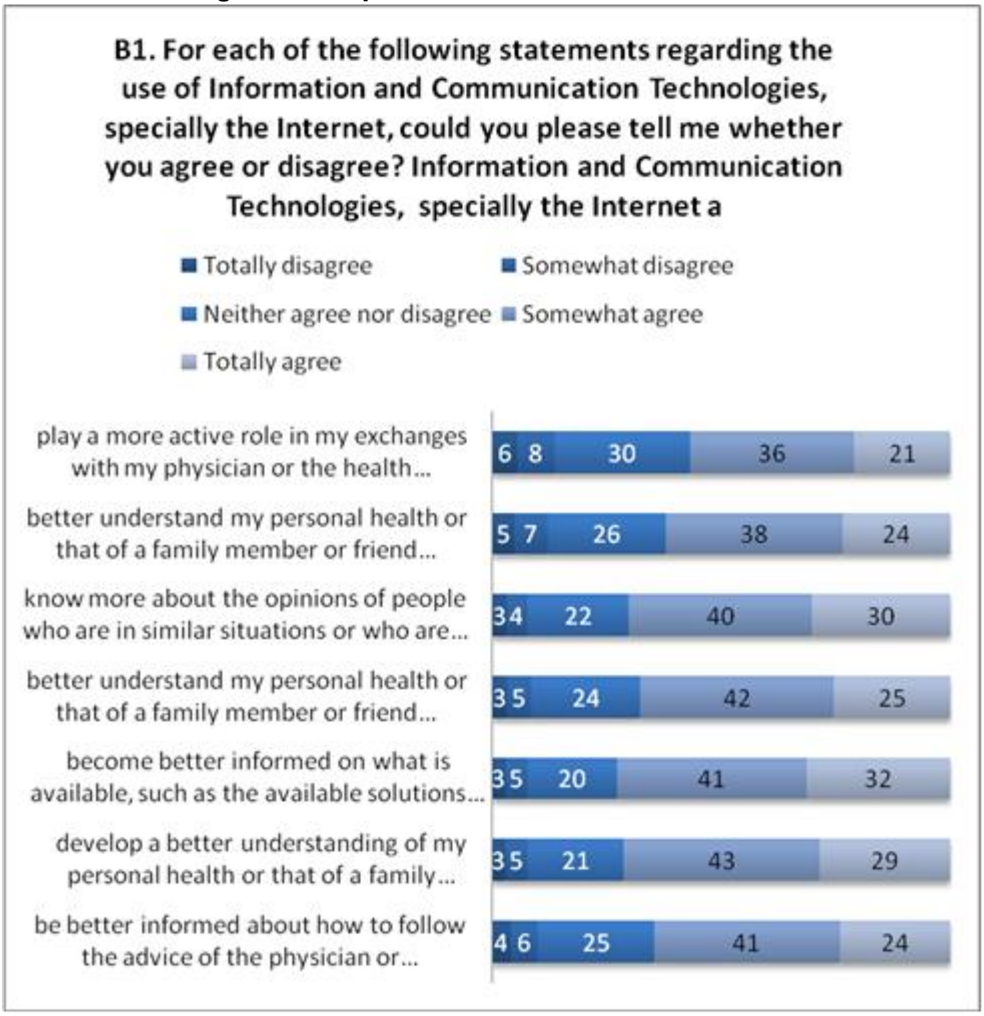

Base: Whole sample.

Overall, this greater digital empowerment for the European citizens when it comes to their health and the healthcare professionals is linked with higher education levels, the worst states of health and the existence of long-standing illnesses.

Table 38: Empowerment and ICT for Health (B1) by socio-demographic

Agree (%)		be better informed about how to follow the advice of the physician or professionals I consult	develop a better understanding of my personal health or that of a family member or friend by giving me access to recognized expert knowledge	become better informed on what is available, such as the available solutions and treatments, so that I can make my own choices	better understand my personal health or that of a family member or friend through my ability to determine what is relevant	know more about the opinions of people who are in similar situations or who are active in support groups	better understand my personal health or that of a family member or friend through online discussions or the opinions of people going through similar experiences	play a more active role in my exchanges with my physician or the health professionals I consult
Gender	Male	65	69	70	66	67	60	56
	Female	65	74*	76*	70*	74*	65*	58
Age group	16-24	61	69	71	64	72*	63	50
	25-54	66	72*	74*	69*	71*	64*	58
	55-74	66	71	72	68*	64	54	61*
Level of education complete d	Primary or lower secondary education	62	68	69	64	68	59	56
	Upper secondary education	66*	71	73	68*	70	62*	57
	Tertiary education	65	73*	74*	69*	72*	63	58
Situation	Employed or self-employed	66*	72	74*	69	71	63	58
	Unemployed	64	71	72	67	68	63	54
	Student	60	70	71	62	72*	63	48
	Other not in the labour force	66	71	72	69*	66	57	61*
Type of locality	Densely-populated area	66	73*	74	69	72	63	57
	Intermediate area	66*	71	73	68	71	63	57
	Thinly-populated area	61	70	70	66	67	59	55
Health status	Bad	71*	74*	78*	74*	74*	62	64*
	Neither good or bad	68*	72	74	69	68	62	60
	Good	64	71	72	67	70	62	55
Long standing illness	Yes	68*	74*	77*	72*	72*	63	61*
	No	63	69	70	65	69	62	54

Base: Whole sample.

On a per-country basis, this perception of greater empowerment with respect to health occurs in countries where the digital divide among citizens is more marked, particularly in Estonia, Slovenia and Slovakia.

Table 39: Empowerment and ICT for Health (B1) by socio-demographic

Agree (%)	AT	BE	DE	DK	EE	ES	FI	FR	IT	NL	SE	SK	SL	UK
be better informed about how to follow the advice of the physician or professionals I consult	65	55	61	64	71	69	63	59	73	55	64	81	84	71
develop a better understanding of my personal health or that of a family member or friend by giving me access to recognized expert knowledge	72	65	69	69	74	75	73	67	77	62	66	84	88	76
become better informed on what is available, such as the available solutions and treatments, so that I can make my own choices	75	64	73	72	81	72	77	67	80	61	72	84	87	77
better understand my personal health or that of a family member or friend through my ability to determine what is relevant	65	62	63	63	75	68	71	68	74	59	63	79	85	73
know more about the opinions of people who are in similar situations or who are active in support groups	75	63	71	66	79	75	72	69	75	57	63	85	86	68
better understand my personal health or that of a family member or friend through online discussions or the opinions of people going through similar experiences	60	54	59	57	69	64	67	64	69	49	57	78	85	63
play a more active role in my exchanges with my physician or the health professionals I consult	58	52	54	60	61	58	60	54	65	51	54	68	69	58

Base: Whole sample.

In the same way, the majority of the surveyed citizens consider that the Internet makes them better equipped for consultations and to relate with the healthcare professionals (64% and 62% respectively), it empowers them to make decisions with respect to their treatments and solutions (63%), and it makes them more confident in their health related exchanges with other people

(62%). Similarly, the Internet also appears to be an excellent tool for health decision making, independently of healthcare professionals or the conventional health system (alternative medicine).

Figure 50: Empowerment and ICT for Health (B2)

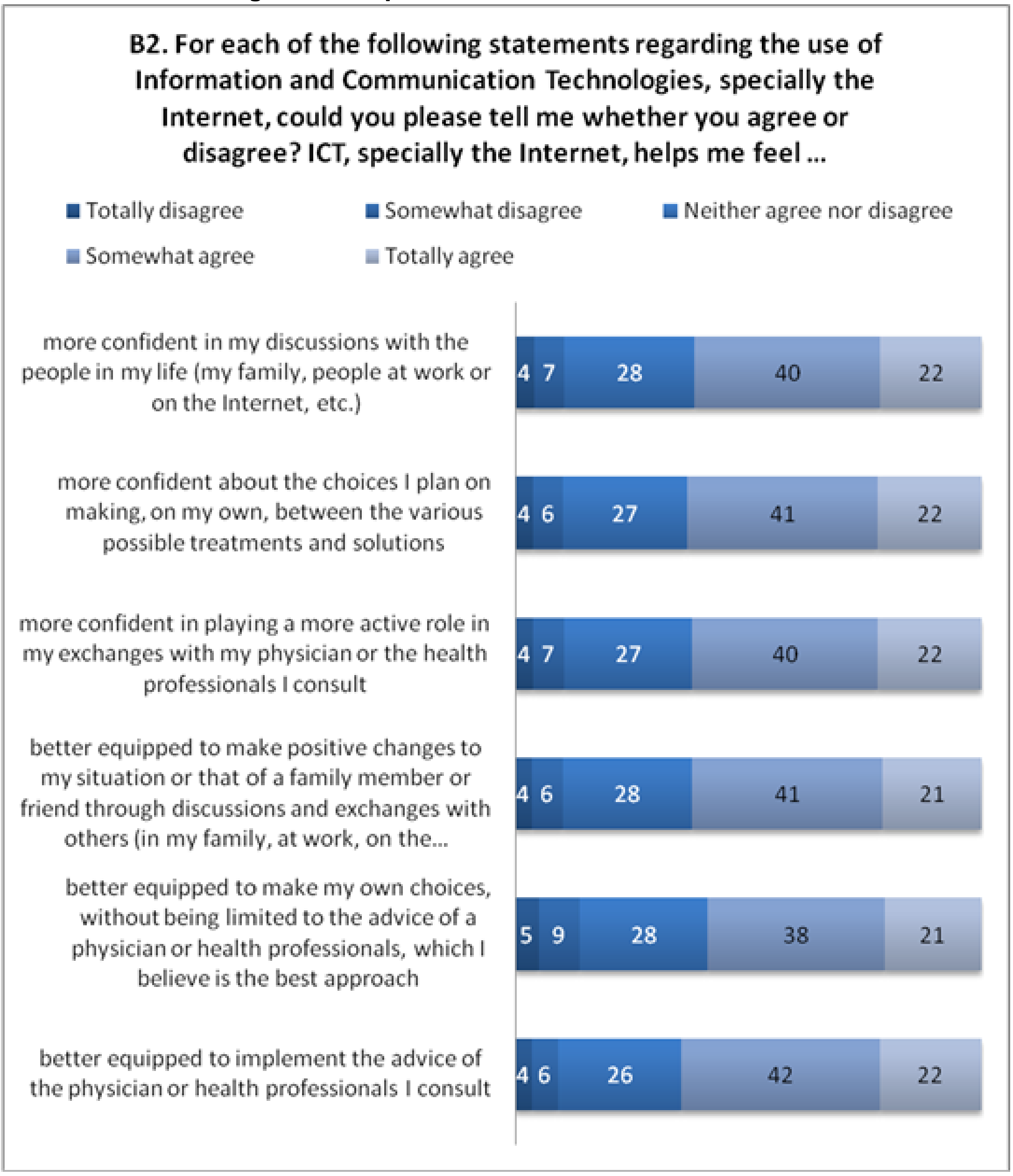

Base: Whole sample.

Again, education levels, the state of health and the persistence of long-standing illnesses, like in countries with a greater digital divide, appear linked with this greater perception of empowerment with respect to health.

Table 40: Empowerment and ICT for Health (B2) by socio-demographic

Agree (%)		better equipped to implement the advice of the physician or health professionals I consult	better equipped to make my own choices, without being limited to the advice of a physician or health professionals which I believe is the best approach	better equipped to make positive changes to my situation or that of a family member or friend through discussions and exchanges with others	more confident in playing a more active role in my exchanges with my physician or the health professionals I consult	more confident about the choices I plan on making, on my own, between the various possible treatments and solutions	more confident in my discussions with the people in my life
Gender	Male	63	57	60	60	61	59
	Female	66*	61*	64*	64*	65*	64*
Age group	16-24	61	55	62	55	62	60
	25-54	65*	60*	63*	64*	64*	62*
	55-74	67*	57	58	64	62	60
Level of education completed	Primary or lower secondary education	63	56	59	59	60	58
	Upper secondary education	65*	59	62	62	64*	62*
	Tertiary education	64	59	63*	63*	63	63*
Situation	Employed or self-employed	65	60*	64*	64	64*	63
	Unemployed	63	58	61	62	63	62
	Student	61	53	61	56	60	58
	Other not in the labour force	66*	57	58	62	63	60
Type of locality	Densely-populated area	65*	58	64*	63*	65	62
	Intermediate area	65	59	62	62	61	62
	Thinly-populated area	62	59	60	61	63	59
Health status	Bad	68*	64*	64*	66*	66	61
	Neither good or bad	65	59	62	63	63	60
	Good	64	58	62	61	63	62
Long standing illness	Yes	68*	61*	64*	66*	66*	63
	No	62	57	60	60	61	61

Base: Whole sample.

Table 41: Empowerment and ICT for Health (B2) by country

Agree (%)	AT	BE	DE	DK	EE	ES	FI	FR	IT	NL	SE	SK	SL	UK
better equipped to implement the advice of the physician or health professionals I consult	69	55	68	60	75	64	61	60	72	53	60	74	75	63
better equipped to make my own choices, without being limited to the advice of a physician or health professionals, which I believe is the best approach	61	50	60	57	70	52	52	55	62	51	57	65	78	65
better equipped to make positive changes to my situation or that of a family member or friend through discussions and exchanges with others (in my family, at work, on the Internet, etc.)	69	51	64	58	75	63	59	58	67	48	61	74	82	63
more confident in playing a more active role in my exchanges with my physician or the health professionals I consult	66	53	64	61	68	60	58	58	68	53	57	72	80	65
more confident about the choices I plan on making, on my own, between the various possible treatments and solutions	67	54	66	60	73	61	63	57	65	53	61	75	82	68
more confident in my discussions with the people in my life (my family, people at work or on the Internet, etc.)	57	53	56	60	73	68	59	61	66	50	60	77	82	67

Base: Whole sample.

Figure 51: Empowerment and ICT for Health (B3)

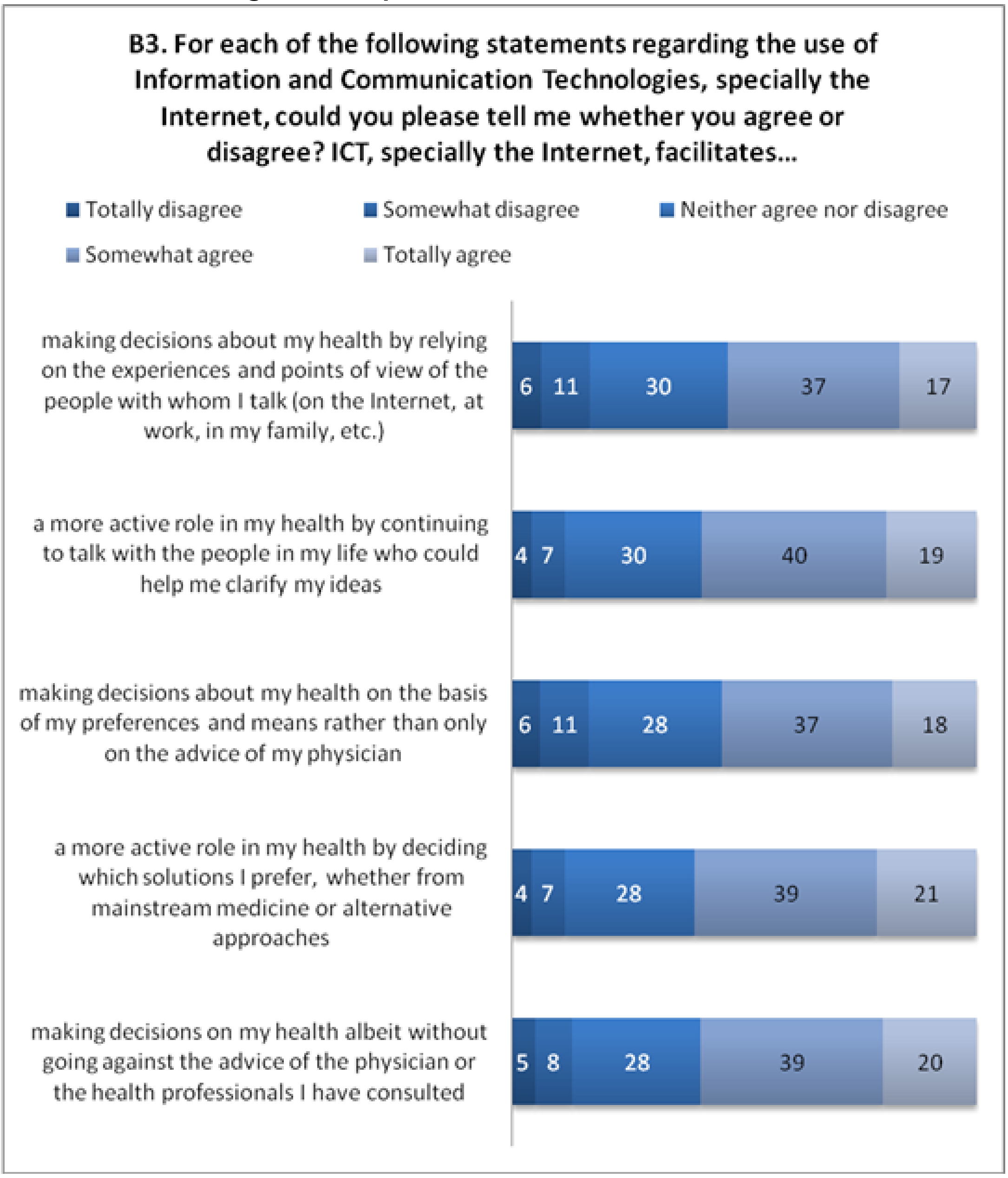

Base: Whole sample.

Table 42: Empowerment and ICT for Health (B3) by socio-demographics

Agree (%)		making decisions on my health albeit without going against the advice of the physician or the health professionals I have consulted	a more active role in my health by deciding which solutions I prefer, whether from mainstream medicine or alternative approaches	making decisions about my health on the basis of my preferences and means rather than only on the advice of my physician	a more active role in my health by continuing to talk with the people in my life who could help me clarify my ideas	making decisions about my health by relying on the experiences and points of view of the people with whom I talk
Gender	Male	58	59	52	57	52
	Female	61*	63*	58*	62*	55*
Age group	16-24	57	55	53	59	57*
	25-54	60*	63*	56*	60*	54
	55-74	59	61	53	56	48
Level of education completed	Primary or lower secondary education	58	60	54	58	51
	Upper secondary education	59	60	55	58	54*
	Tertiary education	60	62*	56	60*	54
Situation	Employed or self-employed	60	62*	57*	61	55
	Unemployed	60	60	51	61	52
	Student	56	56	54	56	55
	Other not in the labour force	59	61	53	56	49
Type of locality	Densely-populated area	60	62*	56	61*	55
	Intermediate area	59	60	54	59	53
	Thinly-populated area	58	60	55	57	52
Health status	Bad	65*	67*	58	63*	55
	Neither good or bad	59	61	57	60	53
	Good	59	60	54*	59	54
Long standing illness	Yes	63*	64*	57*	61*	54
	No	57	58	53	58	53

Base: Whole sample.

Table 43: Empowerment and ICT for Health (B3) by country

Agree (%)	AT	BE	DE	DK	EE	ES	FI	FR	IT	NL	SE	SK	SL	UK
making decisions on my health albeit without going against the advice of the physician or the health professionals I have consulted	59	52	60	65	75	62	61	56	65	48	53	66	57	61
a more active role in my health by deciding which solutions I prefer, whether from mainstream medicine or alternative approaches	68	51	67	60	67	58	57	53	65	52	57	73	74	62
making decisions about my health on the basis of my preferences and means rather than only on the advice of my physician	61	44	61	54	67	49	54	48	55	46	56	69	73	59
a more active role in my health by continuing to talk with the people in my life who could help me clarify my ideas	58	52	57	57	69	63	60	59	66	48	53	74	76	60
making decisions about my health by relying on the experiences and points of view of the people with whom I talk (on the Internet, at work, in my family, etc.)	53	45	58	50	71	51	50	51	53	42	54	69	76	56

Base: Whole sample.

7.3 Barriers

Just as there are factors that justify a good evaluation of health websites, the lack of privacy (51.9%), security (50.5%), reliability (47,2%) and trust (45.7%) were the four main barriers for ICT uses for health indicated by the sampled European population to be very important. Other justifications were the lack of liability (38.2%), health literacy (36.2%), knowledge (33.4%), access to ICTs for health (28.9%), motivation and interest (27.9%), and the lack of digital skills (24.4%).

Figure 52: ICT for Health barriers (D13)

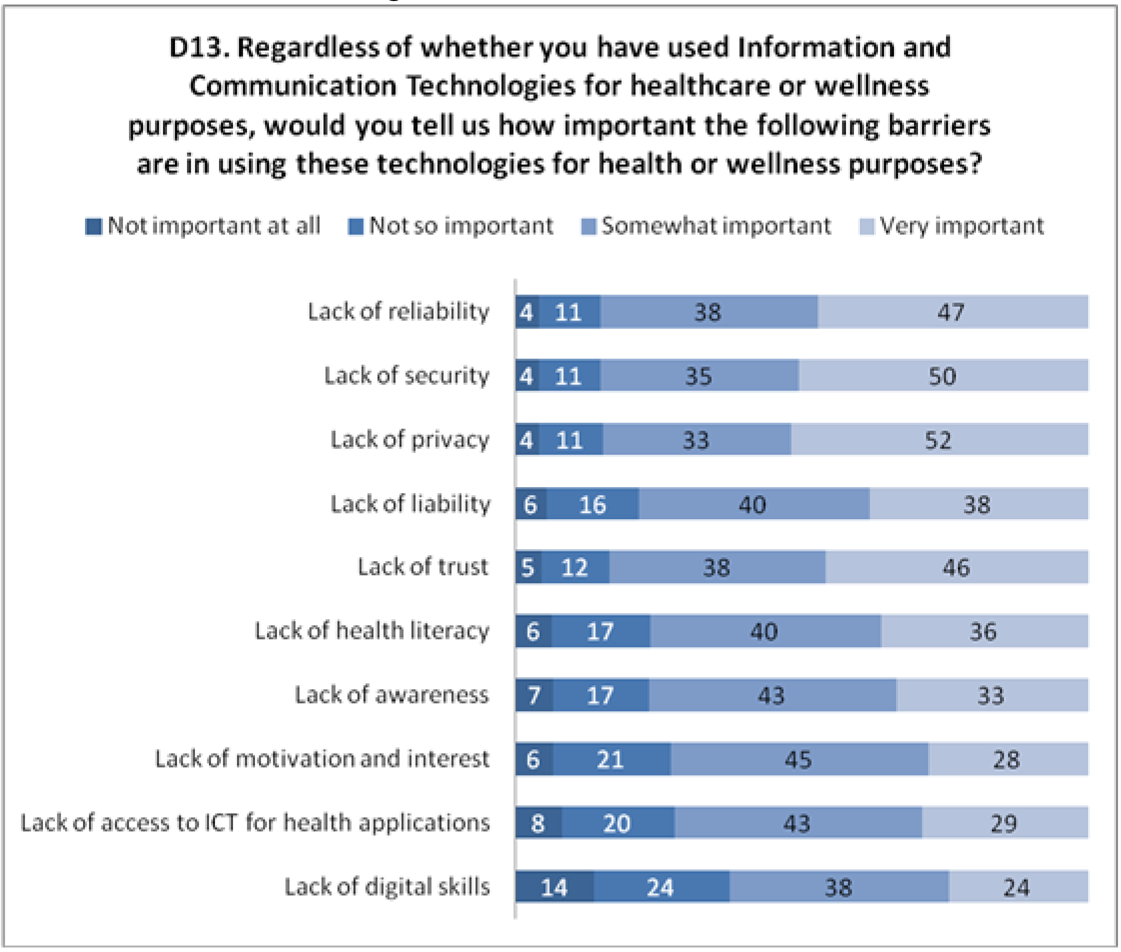

Base: Whole sample.

With respect to the socio-demographic structure of the sampled European population, the analysis of the barriers provides significant indications. Firstly, that women are much more sensitive to the barriers to the ICT use for health than men, particularly in terms of a lack of trust (87.2%), privacy (87.9%), security (87.9%) and liability (88.6%). Similarly, the demonstration of barriers to ICT use for health is also much more evident in older people, those with lower levels of education and the inactive. Lastly, it is also worth highlighting that the presence of long standing illnesses is also very sensitive to the barriers to ICT use, particularly the lack of trust (85.6%), privacy (86.8%), security (87.5%) and liability (87.5%).

Table 44: ICT for Health barriers (D13) by socio-demographics

Important (%)		Lack of digital skills	Lack of access to ICT for health applications	Lack of motivation and interest	Lack of awareness	Lack of health literacy	Lack of trust	Lack of liability	Lack of privacy	Lack of security	Lack of reliability
Gender	Male	60	70	71	74	74	80	76	82	83	82
	Female	66*	75*	75*	80*	80*	87*	81*	88*	88*	89*
Age group	16-24	59	67	71	74	74	79	74	79	80	81
	25-54	63	73*	73	77	77	84*	79*	86*	86*	85*
	55-74	66*	76*	74*	78*	78*	87*	82*	88*	88*	89*
Level of education completed	Primary or lower secondary education	67*	73	75*	79	80	84	80	85	85	85
	Upper secondary education	65*	75*	75	78	78	84	80*	85*	86*	85
	Tertiary education	58	69	70	74	73	83	77*	84	85	86
Situation	Employed or self-employed	62	72	71	75	76	83	79	84	85	85
	Unemployed	66	76*	76	81	80*	86	80	87	87	88
	Student	58	67	72	75	73	79	73	81	80	81
	Other not in the labour force	68*	77*	77*	80	80*	88*	82*	88*	89*	90*
Type of locality	Densely-populated area	64	73	73	77	77	84*	79	85	85	85
	Intermediate area	63	72	73	77	76	83	78	84	85	85
	Thinly-populated area	60	72	71	74	76	85	79	86	86	86
Health status	Bad	63	74	71	76	77	84	76	85	85	85
	Neither good or bad	66*	76*	75	81*	79*	85	81*	87*	88*	87*
	Good	62	71	72	76	76	83	78	84	85	85
Long standing illness	Yes	64*	75*	74	79*	78*	86*	80*	87*	88*	88*
	No	61	70	72	75	75	82	77	83	83	83

Base: Whole sample.

On a per-country basis, from those sampled, the highest percentages are observed for Estonia, Spain, Italy, Slovenia and Slovakia with respect to the proposed indicators in assessing the barriers to ICT uses for health.

Table 45: ICT for Health barriers (D13) by country

Important (%)	AT	BE	DE	DK	EE	ES	FI	FR	IT	NL	SE	SK	SL	UK
Lack of digital skills	58	59	55	66	74	71	71	54	77	70	62	78	77	64
Lack of access to ICT for health applications	73	66	70	63	82	81	77	66	82	72	62	81	84	71
Lack of motivation and interest	74	69	70	77	83	79	80	69	80	75	72	84	81	69
Lack of awareness	76	74	71	78	83	85	82	71	84	83	73	85	86	79
Lack of health literacy	78	76	76	78	80	83	83	71	82	81	77	84	83	73
Lack of trust	84	81	82	84	91	87	88	82	89	84	83	86	89	82
Lack of liability	77	76	75	80	89	84	84	78	85	80	79	84	86	74
Lack of privacy	87	85	85	79	89	89	86	85	85	85	79	82	88	83
Lack of security	85	83	84	83	92	88	86	85	87	86	82	88	88	85
Lack of reliability	85	85	82	83	92	88	86	85	89	87	83	90	91	84

Base: Whole sample.

8. ICT FOR HEALTH ACCESS

8.1 ICT for Health utilisation

When it comes to specifically using the Internet for health and wellness, the research has provided interesting information, with notable relative differences. The main use of the Internet for health is for individual information searches, rather than sharing information, communicating or interacting about health. Information searches about physical illnesses or conditions (40% of the sampled European citizens use the Internet this way at least once a month, and 25% of the citizens at least once a month, but not every week); information searches about wellness and lifestyles (33% less than once a month, and 25% at least once a month, but not every week); bookmarking a health website as a favourite to pay regular visits (20% less than once a month, and 13% at least once a month, but not every week); to look which company or organisation provided the advice or information that appears on a health website (24% less than once a month, and 14% less than once a month, but not every week); and to look for information about a mental health issue like depression or anxiety (23% less than once a month, and 12% less than once a month, but not every week). In fact, individual searches for health information using the Internet make up one of the most frequently mentioned uses by the sampled European citizens. 13% of the respondents look for information about physical illnesses or conditions; 14% look for information about wellness and lifestyles; 13% bookmark a health website as a favourite in their browser to pay regular visits; 14% look which company or organisation provided the advice or information that appears on a health website; and 12% look for information about a mental health issue like depression or anxiety.

Over half of the sampled European citizens have never used the Internet to buy medicine or vitamins online (56% of the total); participated in online support groups for people with the same health issue (60%), used social networking sites for health and wellness issues (58%); used e-mail or websites to communicate with a doctor or their office (58%); analysed the privacy policy for personal information in medical websites (52%); explained a medical issue online in order to make contact with an e-health medical service (61%) or with other users (58%); disclosed medical information on social networking sites (67%); or disclosed medical information on websites to share pictures, videos, or movies (67%).

Table 46: Internet for Health utilisation (D1a)

(%)	Never	Less than once a month	At least once a month (but not every week)	At least once a week (but not every day)	Every day or almost every day	I was not aware of it
looked for information about a physical illness or condition that you or someone you know has	15	40	25	13	4	3
looked for information about wellness or lifestyle	21	33	24	14	4	4
bought medicine or vitamins online	56	17	9	5	2	11
participated in an online support group for people who are concerned about the same health or medical issue	60	12,6	8	5	2	12
participated in Social Networking Sites talking about health and wellness	58	14,2	9	6	3	11
used email or gone to a web site to communicate with a doctor or a doctor's office	58	14	8	5	2	14
clicked on a health or medical web site's privacy policy to read about how the site uses personal information	52	17	9	6	3	12
described a medical condition or problem online in order to get advice from an online doctor	61	13	7	4	2	13
described a medical condition or problem online in order to get advice from other online users (peers)	58	16	9	5	2	10
kept a health web site "bookmarked", or saved as a "favourite place", so you can go back to it regularly	45	20	13	10	4	9
looked to see what company or organization is providing the advice or information that appears on a health web site	44	24	14	7	3	9
looked for information about a mental health issue like depression or anxiety	46	23	12	7	3	9
disclosed medical information on Social Networking Sites	66	8	6	4	2	13
disclosed medical information on websites to share pictures, videos, movies, etc.	67	7	6	5	2	14

Base: Whole sample.

The following observations are notable in terms of the socio-demographic characteristics of the sampled European population. With respect to gender, and establishing significant statistical differences, women stand out for carrying out individual information searches more often. 85% look for information about physical illnesses or conditions, and 79% look for information about wellness or lifestyles. Men, on the other hand, are characterised by a deeper and more interactive use of the Internet for health. 48% of the men sampled bookmarked health websites as favourites in their browser to visit them regularly. 24% of the men sampled disclose medical information on social networking sites, and 23% of the men sampled disclose medical information on health websites using pictures, videos or movies. With respect to the remaining socio-demographic factors, the analysis shows homogeneity in terms of the overall use of the Internet for health, which is more frequent in the young population, those with a tertiary education, students and the employed, those in densely populated urban areas, people in a bad state of health and those with long standing illnesses.

.

Table 47: Internet for Health utilisation (D1a) by socio-demographics (I)

At least once (%)		looked for information about a physical illness or condition that you or someone you know has	looked for information about wellness or lifestyle	bought medicine or vitamins online	participated in an online support group for people who are concerned about the same health or medical issue	participated in Social Networking Sites talking about health and wellness	used email or gone to a web site to communicate with a doctor or a doctor's office	clicked on a health or medical web site's privacy policy to read about how the site uses personal information
Gender	Male	78	72	34*	28	33	31	38*
	Female	85*	79*	31	28	30	27	33
Age group	16-24	83*	80*	35*	36*	44*	36*	40*
	25-54	83*	76*	32	28	31	29	36
	55-74	77	68	30	16	16	20	28
Level of education completed	Primary or lower secondary education	76	67	33	27	29	24	33
	Upper secondary education	81*	74	33*	28	31	29	37*
	Tertiary education	85*	80*	32	28	32	31*	35
Situation	Employed or self-employed	82*	77*	35*	28	32	30*	37
	Unemployed	80	76	27	28	31	26	34
	Student	82*	78	32	34*	39*	33*	38*
	Other not in the labour force	80	68	29	22	22	22	30
Type of locality	Densely-populated area	84*	78*	32	31	34*	33*	38*
	Intermediate area	82	76	33	28	31	29	35
	Thinly-populated area	78	70	33	22	25	23	31
Health status	Bad	86*	72	38*	38*	33*	37*	40*
	Neither good or bad	84	76*	34	32*	34	30	36
	Good	81	76*	32	26	30	28	35
Long standing illness	Yes	85*	77*	36*	31*	33	31*	37*
	No	79	74	30	25	30	27	35

Base: Whole sample.

Table 48: Internet for Health utilisation (D1a) by socio-demographics (II)

At least once (%)		described a medical condition or problem online in order to get advice from an online doctor	described a medical condition or problem online in order to get advice from other online users (peers)	kept a health web site "bookmarked", or saved as a "favourite place", so you can go back to it regularly	looked to see what company or organization is providing the advice or information that appears on a health web site	looked for information about a mental health issue like depression or anxiety	disclosed medical information on Social Networking Sites	disclosed medical information on websites to share pictures, videos, movies, etc.
Gender	Male	28	33	48*	48	43	24*	23*
	Female	24	30	45	46	47	18	16
Age group	16-24	38*	43*	54*	49	55*	32*	31*
	25-54	26	32	48	48	46	21	19
	55-74	13	17	37	41	31	10	9
Level of education completed	Primary or lower secondary education	24	31	42	42	42	21	19
	Upper secondary education	27	32*	46	47*	45	22	20*
	Tertiary education	26	31	49*	48*	46*	20	18
Situation	Employed or self-employed	27	33	48	49*	44	22	20
	Unemployed	26	32	46	43	48	21	17
	Student	35*	39*	52	50*	55*	29	27*
	Other not in the labour force	17	22	39	40	38	13	11
Type of locality	Densely-populated area	30*	34*	50*	51*	49*	24*	22*
	Intermediate area	27	31	47	46	45	22	20
	Thinly-populated area	20	27	41	41	39	16	14
Health status	Bad	25	35*	50*	52*	59*	24*	17
	Neither good or bad	28	33	48	51*	49*	22	20
	Good	26	31	46	46	43	21	19
Long standing illness	Yes	26	33	49*	51*	49*	21	18
	No	26	31	45	44	41	21	20*

Base: Whole sample.

On a per-country basis, Slovenia, Slovakia and the United Kingdom stands out due to a more intensive use of the available online health practices, in particular information searches about physical illness or conditions, about wellness or quality of life, and particularly, e-commerce in health.

Table 49: Internet for Health utilisation (D1a) by country

At least once (%)	AT	BE	DE	DK	EE	ES	FI	FR	IT	NL	SE	SK	SL	UK
looked for information about a physical illness or condition that you or someone you know has	79	75	80	75	78	84	81	75	87	73	78	92	93	89
looked for information about wellness or lifestyle	74	71	71	68	76	79	82	71	84	61	69	91	91	81
bought medicine or vitamins online	29	15	56	24	26	22	20	20	25	21	22	36	32	35
participated in an online support group for people who are concerned about the same health or medical issue	23	20	30	18	17	34	21	22	35	25	14	40	41	28
participated in Social Networking Sites talking about health and wellness	28	24	34	29	36	41	27	29	39	24	23	38	44	24
used email or gone to a web site to communicate with a doctor or a doctor's office	28	23	28	50	37	33	23	19	46	23	37	35	36	27
clicked on a health or medical web site's privacy policy to read about how the site uses personal information	35	32	41	35	36	34	22	35	46	31	26	34	28	28
described a medical condition or problem online in order to get advice from an online doctor	22	18	24	25	26	34	16	22	41	22	16	33	36	24
described a medical condition or problem online in order to get advice from other online users (peers)	27	26	36	21	32	37	25	31	39	30	20	40	38	23
kept a health web site "bookmarked",	45	46	49	60	34	43	28	55	67	31	40	45	54	33

or saved as a "favourite place", so you can go back to it regularly														
looked to see what company or organization is providing the advice or information that appears on a health web site	57	41	57	39	62	45	42	39	60	41	42	60	57	36
looked for information about a mental health issue like depression or anxiety	44	39	49	47	48	52	49	38	51	39	40	41	41	42
	AT	BE	DE	DK	EE	ES	FI	FR	IT	NL	SE	SK	SL	UK
disclosed medical information on Social Networking Sites	21	18	24	15	17	22	14	16	34	22	14	18	21	17
disclosed medical information on websites to share pictures, videos, movies, etc.	20	16	24	15	12	20	8	16	32	20	9	17	15	13

Base: Whole sample.

Beyond the use of information, the specific use of ICTs in the health sector, particularly the Internet, is still quite limited among the sampled European citizens. Around three-quarters of the sampled population have never used any of the specified ICTs for health uses. 78.9% of the total have never made an online consultation through videoconference with healthcare professionals. 74.8% haven't received medical or clinical tests online either. 77.2% haven't accessed or uploaded medical results via a specialist provider, such as Google Health or Microsoft Vault. 76.4% haven't accessed or uploaded medical results via an Internet application provided by a health organisation. 76.6% haven't used health or wellness applications on mobile telephones either. And 73.6% of the sampled population have not used ICT applications to transmit vital signs and other clinical information anytime or anywhere.

On the other hand, there are some specific ICTs for health uses that are used more, although the majority are not used either. 16.0% of the sampled European population has made, cancelled or changed an appointment with their family doctor, specialist or any other health professional at least once a month, which becomes 6.5% when the frequency becomes once a month, but not every week. In the same manner, around 20% of the sampled population has sent or received an email from a doctor, nurse and health organisation at least once a month, or at least once a month, but not every week. Along the same lines, around a fifth of the sampled population (16.5% less than once a month, and 9.2% at least once a month, but not every week) have received an email message about a health promotion or health prevention. The research results conclude, therefore, a quite basic usage of ICTs in health, which are centred on appointments with professionals, and the sending/receiving of emails with health professionals or health promotions/prevention.

Table 50: Information and Communication Technology utilisation (D10a)

(%)	Never	Less than once a month	At least once a month (but not every week)	At least once a week (but not every day)	Every day or almost every day	I was not aware of it
Made, cancelled or changed an appointment with your family doctor, specialist or other health professionals online	66,7	16,0	6,5	3,7	2,0	6,1
Sent or received an email from your doctor, nurse or health care organization	68,2	14,6	5,5	3,7	1,5	6,4
Made an online consultation through videoconference with your doctor or nurse	78,9	3,5	3,8	2,6	1,4	9,8
Received online the results of your clinical or medical test.	74,8	8,3	4,4	3,3	1,4	7,9
Accessed or uploaded your (or any other family member) medical information or health record through an Internet provider (ex. Google Health, Microsoft Vault…)	77,2	4,7	4,2	3,1	1,3	9,5
Accessed or uploaded your (or any other family member) medical information or health record through an Internet application provided by your healthcare organization	76,4	5,4	4,3	3,2	1,4	9,3
Used a game console to play games related with your health or your wellness	71,8	8,5	6,1	4,1	1,7	7,8
Used a health/wellness application on your mobile phone	76,6	6,4	4,9	3,8	1,4	6,8
Used devices (as pulse meter, glucose meter…) to transmit vital signs or other clinical information and/or received alarms or follow-up about your health anytime, anywhere	73,6	7,6	5,6	4,0	2,0	7,2
Received any message about health promotion and/or health prevention	61,2	16,5	9,2	5,5	2,4	5,1

Base: Whole sample.

With respect to the socio-demographic categories of the sampled population, the following results stand out. Firstly, in clear contrast to what occurs with information searches, and unlike men, women do not stand out for their use of ICTs in the health sector. ICTs for health use are mainly used by men. Secondly, it is also worth highlighting that uses of ICTs in health are different in the youngest population compared to older age groups. Thirdly, a higher education level is associated with more intensive uses of ICTs in health. Around a third of the sampled population that have completed tertiary education have made, cancelled or changed an appointment with a healthcare professional, have sent or received an email from a health professional or organization, or have received an online message about a health promotion or health prevention. Fourthly, students and those living in densely populated areas also stand out for one of the most frequent uses of ICTs in health with respect to other labor situations or types of urban living. And fifthly, and in general terms, a good state of health and a lack of long standing illnesses is linked with the most intensive use of ICTs in health.

Table 51: Information and Communication Technology utilisation (D10a) by socio-demographics

% At least once		Made, cancelled or changed an appointment with your family doctor, specialist or other health professionals online	Sent or received an email from your doctor, nurse or health care organization	Made an online consultation through videoconfere nce with your doctor or nurse	Received online the results of your clinical or medical test.	Accessed or uploaded your medical information or health record through an Internet provider
Gender	Male	34	33*	16*	23*	19*
	Female	28	23	8	14	11
Age group	16-24	36*	33*	23*	28*	24*
	25-54	31*	28	12	18	14
	55-74	21	21	3	10	7
Level of education complete d	Primary or lower secondary education	26	26	12	19	15
	Upper secondary education	30	27	13	19	15
	Tertiary education	33*	29*	12	19	14
Situation	Employed or self-employed	32*	29*	13	20	15
	Unemployed	34	23	11	16	13
	Student	35*	32*	21*	26*	23*
	Other not in the labour force	21	23	5	11	8
Type of locality	Densely-populated area	37*	33*	15*	23*	17*
	Intermediate area	29	25	12	17	15
	Thinly-populated area	22	23	8	14	11
Health status	Bad	31*	36*	8	15	9
	Neither good or bad	30	29	11	18	13
	Good	31	27	13*	19	16
Long standing illness	Yes	33*	31*	11	18	13
	No	29	25	14	19	16*

Base: Whole sample.

Table 52: Information and Communication Technology utilisation (D10a) by socio-demographics

% At least once		Accessed or uploaded your information or health record through an Internet application provided by your healthcare organization	Used a game console to play games related with your health or your wellness	Used a health/wellness application on your mobile phone	Used devices to transmit vital signs or other clinical information and/or received alarms or follow-up about your health	Received any message about health promotion and/or health prevention
Gender	Male	20*	26*	23*	26*	41*
	Female	12	19	14	16	33
Age group	16-24	26*	38*	33*	30*	47*
	25-54	15	22	17	20	36
	55-74	7	5	4	16	29
Level of education completed	Primary or lower secondary education	15	20	16	22	33
	Upper secondary education	17	23	19	22*	37
	Tertiary education	15	22	19	19	39*
Situation	Employed or self-employed	16	23	19	21	38
	Unemployed	14	20	15	18	34
	Student	23*	36*	31*	30*	48*
	Other not in the labour force	8	9	7	17	28
Type of locality	Densely-populated area	18*	25*	21*	22*	41*
	Intermediate area	15	22	18	20	36
	Thinly-populated area	11	18	13	20	32
Health status	Bad	12	14	12	22	36
	Neither good or bad	15	18	16	21	36
	Good	16	24*	19*	21	38
Long standing illness	Yes	14	18	15	22*	37*
	No	17*	25*	20*	20	37

Base: Whole sample.

On a per-country basis, the greater intensity of use of the majority of ICTs for health is clearly evident in Italy, which leads the way for online consultations through videoconferencing with health professionals, accessing and obtaining medical information through an Internet provider, the use of consoles or games related to health or wellness, the use of health applications on mobile

telephones, and for having received online health promotions or health preventions. Furthermore, the high level of appointments made, changed or cancelled at health centres in Spain also has to be highlighted (53.9%), or the sending or receiving of emails by health professionals and organizations in Denmark (50.7%).

Table 53: Information and Communication Technology utilisation (D10a) by socio-demographics by country

At least once (%)	AT	BE	DE	DK	EE	ES	FI	FR	IT	NL	SE	SK	SL	UK
Made, cancelled or changed an appointment with your family doctor, specialist or other health professionals online	27	24	26	39	39	54	47	17	32	21	32	17	15	25
Sent or received an email from your doctor, nurse or health care organization	28	22	28	51	32	25	30	19	36	25	31	19	20	19
Made an online consultation through videoconference with your doctor or nurse	7	8	11	10	6	17	7	10	19	9	4	10	7	9
Received online the results of your clinical or medical test.	18	15	16	34	21	24	17	16	31	14	9	11	11	9
Accessed or uploaded your medical information or health record through an Internet provider	12	13	14	13	12	19	9	12	20	15	5	11	10	9
Accessed or uploaded your medical information or health record through an Internet application provided by your healthcare organization	13	13	15	17	18	19	7	14	22	13	6	11	10	8
Used a game console to play games related with your health or your wellness	18	15	22	16	11	27	18	21	25	20	12	16	14	14
Used a health/wellness application on your mobile phone	17	10	18	13	12	20	12	15	22	12	14	16	10	14
Used devices to transmit vital signs or other clinical information and/or received alarms or follow-up about your health anytime, anywhere	23	20	24	15	19	25	12	14	23	24	13	27	24	12
Received any message about health promotion and/or health prevention	32	30	37	26	49	43	32	35	47	23	22	38	45	20

Base: Whole sample.

8.2 ICT for Health willingness and awareness

Individuals who stated they never carry out these activities or they were not aware of them were asked about their willingness to carry out these activities.

Table 54: Internet for Health utilisation (D1b)

	Likely (%)	Base % of whole sample*
look for information about a physical illness or condition that you or someone you know has	40	84
use email or gone to a web site to communicate with a doctor or a doctor's office	32	29
look for information about wellness or lifestyle	29	75
click on a health or medical web site's privacy policy to read about how the site uses personal information	25	35
describe a medical condition or problem online in order to get advice from an online doctor	24	26
keep a health web site "bookmarked", or saved as a "favourite place", so you can go back to it regularly	23	47
look to see what company or organization is providing the advice or information that appears on a health web site	23	48
participate in an online support group for people who are concerned about the same health or medical issue	18	28
look for information about a mental health issue like depression or anxiety	16	45
describe a medical condition or problem online in order to get advice from other online users (peers)	15	31
buy medicine or vitamins online	14	32
participate in Social Networking Sites talking about health and wellness	14	31
disclose medical information on Social Networking Sites	7	21
disclose medical information on websites to share pictures, videos, movies, etc.	6	19

* 'Never' or 'I was not aware of it' in the equivalent question in D1a.

Table 55: Information and Communication Technology willingness (D10b)

	Likely	Base<br>(% of whole sample)
Make, cancel or change an appointment with your family doctor, specialist or other health professionals online	49	28
Send or receive an email from your doctor, nurse or health care organization	43	25
Make an online consultation through videoconference with your doctor or nurse	20	21
Receive online the results of your clinical or medical test.	43	17
Access or upload your medical information or health record through an Internet provider (ex. Google Health, Microsoft Vault…)	22	13
Access or upload your medical information or health record through Internet application provided by your healthcare organization	28	14
Use a game console to play games related with your health or your wellness	12	20
Use a health/wellness application on your mobile phone	14	16
Use devices (as pulse meter, glucose meter…) to transmit vital signs or other clinical information and/or received alarms or follow-up about your health anytime, anywhere	25	19
Receive any message about health promotion and/or health prevention	28	34

* 'Never' or 'I was not aware of it' in the equivalent question in D10a.

9. INTERNET HEALTH INFORMATION

9.1 Internet health information utilisation

When it comes to the nature of the health or wellness information that is being searched online, it is important to indicate that the large majority of the sampled European population (84.5%) looks for information for their own use. Information searches for other people, such as parents (39.1%), children (29.1%), other relatives (39.4%) and people other than relatives (39.4%) fall very short of information searches for personal use.

Figure 53: Social life of Internet health information (D2)

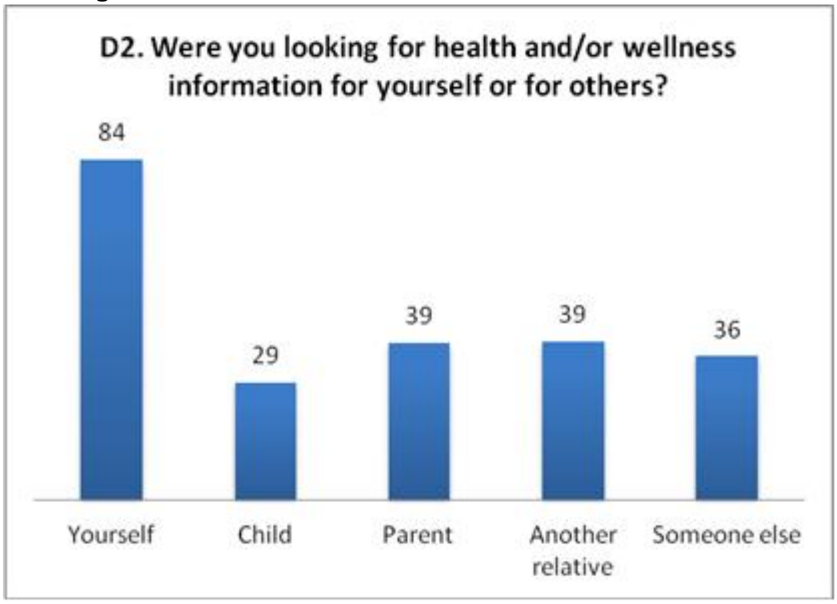

Base: Looked for information about a physical illness or about wellness or lifestyle (88% of whole sample).

With respect to the socio-demographic categories of the surveyed population, the individual or collective nature of the health information searches leads to some significant conclusions. Firstly, that woman are characterized by their greater usage of the Internet for health than men, both in terms of individual information (87%), and particularly, when it comes to information for other people (32% for children and 42% for parents). Secondly, to highlight that the youngest population tends to look for information for themselves (86% for the population aged between 16 and 24 years old) or for their parents (43%), whilst the oldest population is characterized by information searching for their children (36% of the 25 to 54 years old sample) or for their partners (42% of the 55 and 74 years old sample). Thirdly, greater uses of the Internet for health can be seen, both for personal use and for that of other people, in larger households. Finally, with respect to state of health, two arguments are evident. Firstly, the population in a bad state of health tends to look for information for personal use (95%). Secondly, the sampled population with long standing illnesses combines their use mainly for personal information (91%) with the use to find information for other people, in particular their partner (42%) and people other than relatives (37%).

Table 56: Internet for health utilisation (D2) by socio-demographics

% Yes		Yourself	Child	Parent	Another relative	Someone else
Gender	Male	82	26	36	38	33
	Female	87*	32*	42*	41*	38*
Age group	16-24	86	12	43*	40	44
	25-54	85	36*	42*	39	35
	55-74	81	25	25	42*	28
Level of education completed	Primary or lower secondary education	84	30	30	35	31
	Upper secondary education	85	29	40*	41*	35
	Tertiary education	84	29	42*	40	38
Situation	Employed or self-employed	84	34*	41*	39	35
	Unemployed	84	30	42*	39	36
	Student	86*	10	45*	41	46*
	Other not in the labour force	85	30	27	41	28
Type of locality	Densely-populated area	86*	27	40	40	40*
	Intermediate area	84	30	40	40	34
	Thinly-populated area	82	31*	35	37	30
Members in the household	1	86	9	30	26	39
	2	85	16	33	41*	34
	3	84	42*	45*	43*	35
	4+	84	42*	46	42*	36
Health status	Bad	95*	27	33	38	34
	Neither good or bad	90*	28	40	39	35
	Good	82	30	39	40	36
Long standing illness	Yes	91*	29*	38	42*	37*
	No	79	29	39	37	34

Base: Looked for information about a physical illness or or about wellness or lifestyle (88% of whole sample).

On a per-country basis, intense use of the Internet for health can be seen, both from an individual perspective and for other people, in Estonia, Slovakia and Slovenia, whilst Finland stands out in terms of information for personal use and for children, the United Kingdom for information for personal use, and Spain and Italy for information for parents and other relatives.

Table 57: Internet for Health utilisation (D2) by country

Yes (%)	AT	BE	DE	DK	EE	ES	FI	FR	IT	NL	SE	SK	SL	UK
Yourself	87	74	88	84	89	85	89	78	85	72	83	88	90	89
Child	32	30	23	26	56	30	39	33	29	32	36	47	44	28
Parent	39	33	36	23	55	51	39	41	56	28	27	54	61	29
Another relative	39	34	35	34	44	48	32	40	52	38	29	52	46	34
Someone else	41	29	42	28	49	37	37	27	47	31	35	44	49	28

Base: Looked for information about a physical illness or or about wellness or lifestyle (88% of whole sample).

When it comes to motives for using the Internet for health for personal use or for others, it is important to indicate two basic conclusions. The first is that the use of online personal health information is directly associated with visiting the doctor. 51% of the sampled European citizens consulted the Internet for personal health information before visiting the doctor, and 51% of the sampled European citizens consulted the Internet for personal health information after visiting the doctor. The second is that the use of online health information for other people is mainly related to a visit to the doctor that has already taken place (46%) or is unrelated to visiting the doctor (44%).

Figure 54: Internet health information and doctor's consultation (D3)

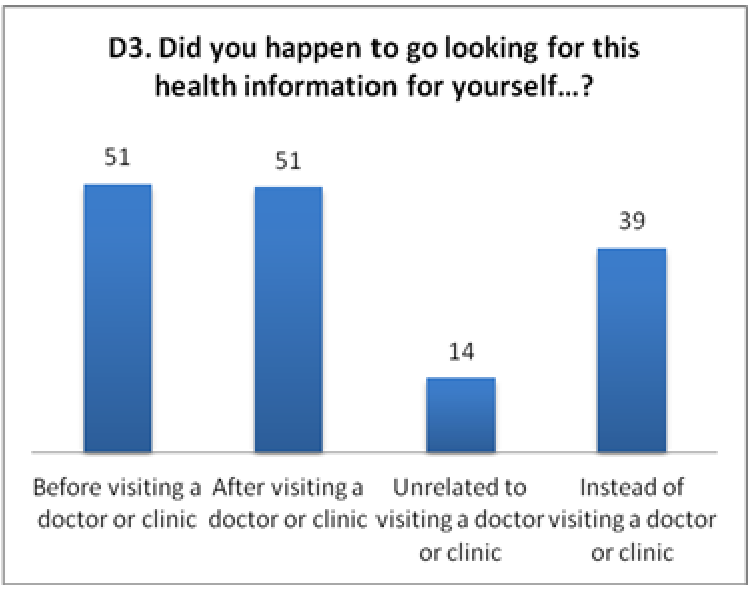

Base: Looked for information about a physical illness or about wellness or lifestyle (88% of whole sample).

Figure 55: Internet health information and doctor's consultation (D4)

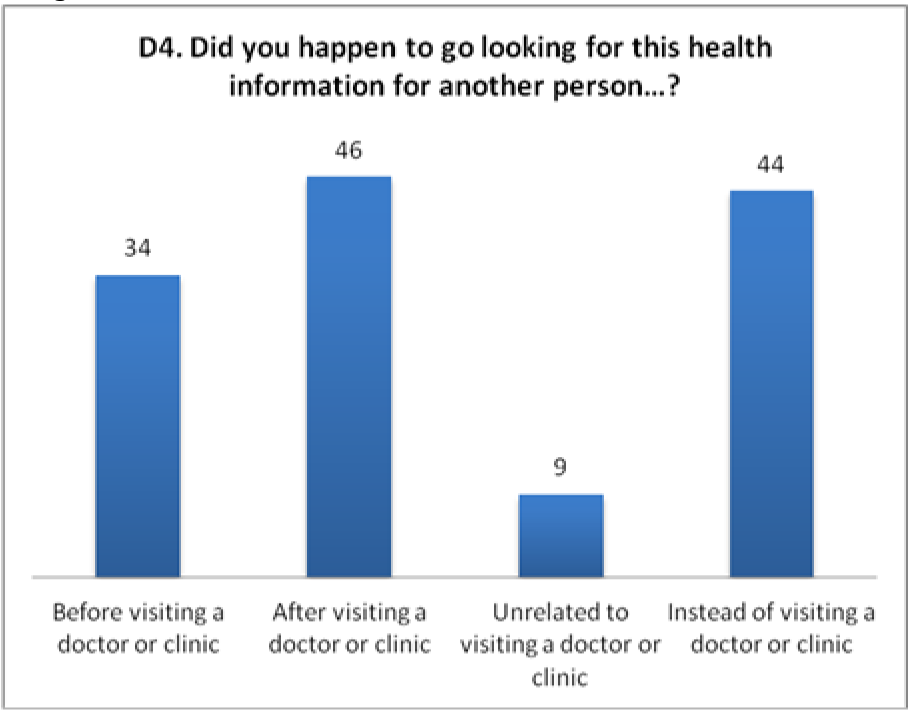

Base: Looked for information about a physical illness or about wellness or lifestyle (88% of whole sample).

With respect to the socio-demographic characteristics of the sampled European population, the motives for the use of the personal online health information are linked with visiting the doctor and are carried out differentially by women, young people, those with a tertiary education, students, those in densely populated areas, and households with many members. When it comes to state of health, 69% of the sampled population in a bad state of health uses online information for personal use after visiting the doctor. In the same manner, 60% of the sampled population with a long-standing illness use online information for personal use after visiting the doctor.

Table 58: Did you happen to go looking for this health information for yourself…?, by socio-demographic variables (D3) by socio-demographics

% Yes		Before visiting a doctor or clinic	After visiting a doctor or clinic	Instead of visiting a doctor or clinic	Unrelated to visiting a doctor or clinic
Gender	Male	51	49	13	39
	Female	51	52*	15	39
Age group	16-24	61*	43	22*	41
	25-54	51	53*	13	37
	55-74	39	51	8	44*
Level of education completed	Primary or lower secondary education	45	45	15	39
	Upper secondary education	49	50	14	39
	Tertiary education	56*	53*	15	39*
Situation	Employed or self-employed	52	51	14	38
	Unemployed	51	50	15	33
	Student	60*	44	21*	44
	Other not in the labour force	42	55*	9	41
Type of locality	Densely-populated area	55*	50	16*	39*
	Intermediate area	50	51	13	39
	Thinly-populated area	48	50	12	38
Members in the household	1	47	48	12	48*
	2	49	52	14	40*
	3	53*	52	14	35
	4+	55*	50	15*	36
Health status	Bad	48	69*	10	37
	Neither good or bad	48	58	13	37
	Good	53*	46	15*	40
Long standing illness	Yes	49	60	12	38
	No	53*	43*	16*	39

Base: Looked for information about a physical illness or about wellness or lifestyle (88% of whole sample).

On a per-country basis, Estonia, Finland, Slovakia and Slovenia again stand out for the medical visit motive for their personal and collective uses of online health information. Spain can be mentioned

as a stand out case, leading the way in terms of use of e-health information for personal use and for other people, after visiting the doctor (59% and 61%, respectively), as can the cases of Slovakia and Slovenia, where a quarter of the population that makes personal use of online health information, doing so independently of the medical visit.

Table 59: Did you happen to go looking for this health information for yourself...?, by socio-demographic variables (D3) by country

Yes (%)	AT	BE	DE	DK	EE	ES	FI	FR	IT	NL	SE	SK	SL	UK
Before visiting a doctor or clinic	49	41	52	49	64	51	62	51	56	41	49	54	60	50
After visiting a doctor or clinic	48	54	49	45	48	59	53	53	51	50	36	54	49	48
Unrelated to visiting a doctor or clinic	14	8	19	12	19	11	20	8	9	11	22	25	26	17
Instead of visiting a doctor or clinic	50	42	48	38	48	18	53	41	46	36	46	48	42	29

Base: Looked for information about a physical illness or about wellness or lifestyle (88% of whole sample).

With respect to the use of health information and the Internet for other people, the analysis of statistical differences again suggests intensive use linked with a medical visit by women, young people, households with many members, and a population that is in a bad state of health or has long-standing illnesses. As a differentiating factor, the use of online medical information for non-personal use, which is not linked to a medical visit, is evident in the older population (50.9% of people between 55 and 74 years old), the inactive (49.8%) and single member households (55.1%).

Table 60: Did you happen to go looking for this health information for another person? by socio-demographic variables (D3) by socio-demographics

% Yes		Before visiting a doctor or clinic	After visiting a doctor or clinic	Instead of visiting a doctor or clinic	Unrelated to visiting a doctor or clinic
Gender	Male	35	44	10	44
	Female	34	47*	9	44
Age group	16-24	41*	42	15*	43
	25-54	35	48*	9	43
	55-74	26	39	5	51*
Level of education completed	Primary or lower secondary education	31	39	9	45
	Upper secondary education	33	47	10	44
	Tertiary education	38*	47	9	44*
Situation	Employed or self-employed	36	47	9	43
	Unemployed	38	43	9	40
	Student	35	45	15*	43
	Other not in the labour force	25	43	8	50*
Type of locality	Densely-populated area	36*	47	10	43
	Intermediate area	33	44	10	44
	Thinly-populated area	33	45	8	44
Members in the household	1	27	41	7	55*
	2	32	45	9	44
	3	36*	44	9	43
	4+	38*	48	11*	40
Health status	Bad	30	52*	10	47
	Neither good or bad	31	46	8	45
	Good	36*	45	10	44
Long standing illness	Yes	33	50*	9	44
	No	36*	42	10	43

Base: Looked for information about a physical illness or about wellness or lifestyle (88% of whole sample).

Table 61: Did you happen to go looking for this health information for another person…?, by country (D3) by country

Yes (%)	AT	BE	DE	DK	EE	ES	FI	FR	IT	NL	SE	SK	SL	UK
Before visiting a doctor or clinic	31	26	34	28	41	37	49	33	38	24	35	35	36	35
After visiting a doctor or clinic	43	39	46	34	41	61	47	44	48	35	40	43	47	40
Unrelated to visiting a doctor or clinic	9	7	12	9	12	7	13	6	7	10	9	13	13	13
Instead of visiting a doctor or clinic	50	55	47	49	49	24	52	49	48	51	46	50	45	39

Base: Looked for information about a physical illness or about wellness or lifestyle (88% of whole sample).

With respect to the usefulness of the health information obtained online, around two-thirds (65%) of the sampled European population consider it to be somewhat useful. Furthermore, an additional fifth part of the sample (20%) considers it to be very useful.

Figure 56: How useful was the health information you got online? (D5)

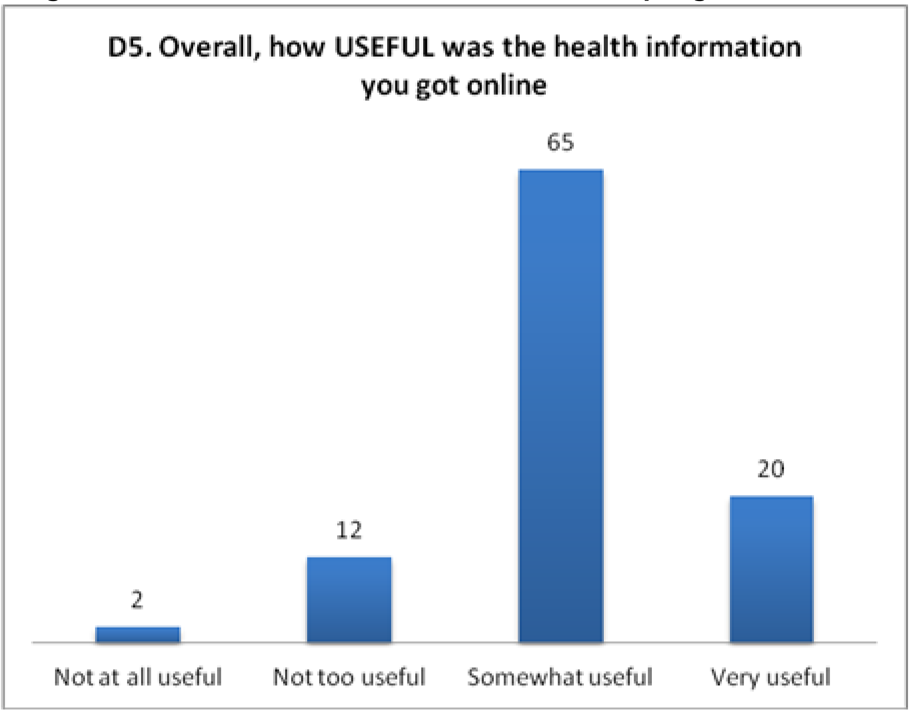

Base: Looked for information about a physical illness or about wellness or lifestyle (88% of whole sample).

With respect to the socio-demographic characteristics of the population, the perception of the usefulness of the online health information stands out in the employed (66.0%) and students (67.0%), in the same manner that the information is perceived to have a greater usefulness for the population with secondary education (20.9%), the unemployed (22.0%), households with many members (21.5%) and people with long standing illnesses (22,5%). As a negative counterpoint, 5.0% of the 55 to 74 year old population who consulted online health information do not find it useful.

Table 62: How useful was the health information you got online? (D5) by socio-demographics

		Not at all useful	Not too useful	Somewhat useful	Very useful
Gender	Male	2	12	66	19
	Female	2	12	65	22
Age group	16-24	2	13	67	18
	25-54	2	11	66	22
	55-74	5*	13	63	19
Level of education completed	Primary or lower secondary education	3	11	64	22
	Upper secondary education	2	12	65	21*
	Tertiary education	2	12	66	19
Situation	Employed or self-employed	2	11	66*	21
	Unemployed	2	11	64	22*
	Student	2	15	67*	16
	Other not in the labour force	4*	12	63	21
Type of locality	Densely-populated area	2	11	66	21
	Intermediate area	2	12	66	20
	Thinly-populated area	3	13	64	20
Members in the household	1	3	11	66	21
	2	3	13	65	20
	3	2	12	66	20
	4+	2	12	66	22*
Long standing illness	Yes	2	11	64	23*
	No	3	12	67	19

Base: Looked for information about a physical illness or about wellness or lifestyle (88% of whole sample).

On a per-country basis, Germany (24%), Spain (24%), Holland (20%), Sweden (22%), and particularly, Austria (26.5%), Slovenia (30.6%) and the United Kingdom (23.5%) stand out for a very positive perception of the usefulness of the medical information consulted online.

Figure 57: How useful was the health information you got online? (D5) by country

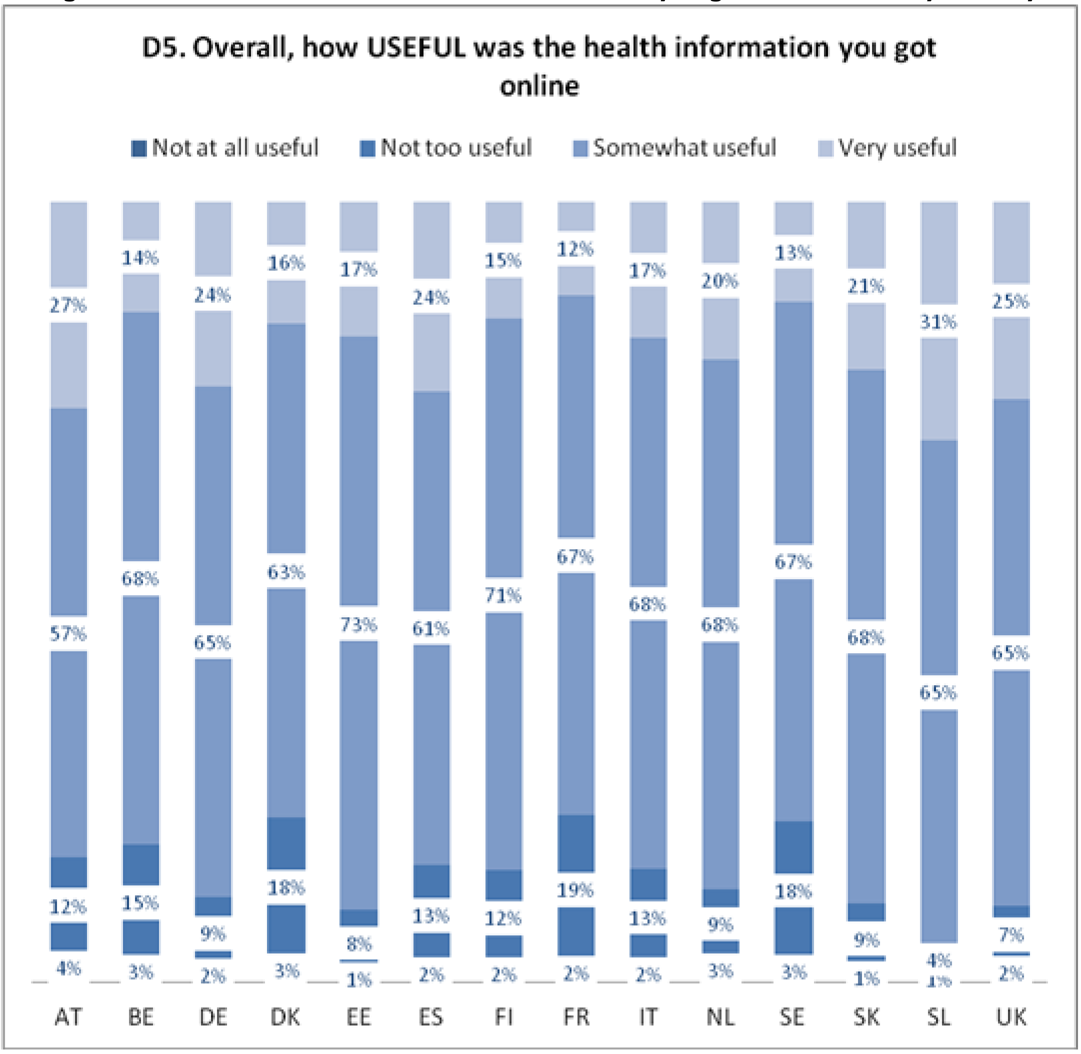

Base: Looked for information about a physical illness or about wellness or lifestyle (88% of whole sample).

Another way of discovering the usefulness of online medical information is that it can lead to users gaining new knowledge. Relevant information of this type has also been obtained from the research. Three-quarters of the sampled European population indicate that they have found online medical information to be useful for learning something.

Figure 58: Did you learn anything NEW from the information you got online, or not? (D6)

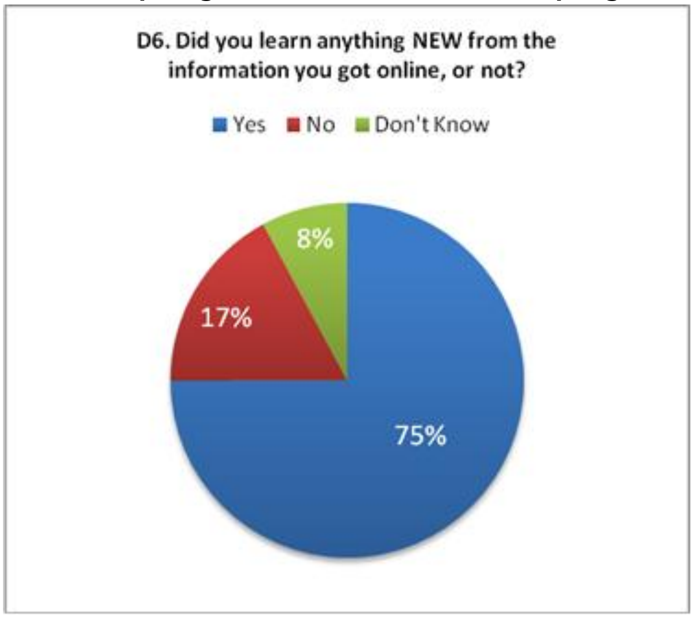

Base: Looked for information about a physical illness or about wellness or lifestyle (88% of whole sample).

This learning is characterized by young people (78.5%), the middle age group (76.3% aged between 25 and 54 years old), those with a tertiary education (76.8%), students (78.3%), and households with more members. On the other hand, the inability to learn through the use of online medical information is characterized by older people (25.2% of the population aged between 55 and 74 years old), those with primary or lower secondary education (22.5%), the inactive (21.9%), those that live in thinly populated areas (20.6%) and households with few members (10.7% in single member households).

Table 63: Did you learn anything NEW from the information you got online, or not? (D6) by socio-demographics

		Yes	No	Don't know
Gender	Male	76	17	8
	Female	74	18	8
Age group	16-24	79*	13	9
	25-54	76*	16	8
	55-74	66	25*	9
Level of education completed	Primary or lower secondary education	70	23*	8
	Upper secondary education	75	17	8
	Tertiary education	77*	16	8
Situation	Employed or self-employed	76	17	8
	Unemployed	75	17	8
	Student	78*	14	8
	Other not in the labour force	70	22*	9
Type of locality	Densely-populated area	77	16	8
	Intermediate area	76	17	8
	Thinly-populated area	71	21*	8
Members in the household	1	72	20*	9
	2	73	19	8
	3	77*	15	8
	4+	77*	15	7
Long standing illness	Yes	76	18	7
	No	75	17	8

Base: Looked for information about a physical illness or about wellness or lifestyle (88% of whole sample).

On a per-country basis, learning through online medical information stands out in Spain (82.6%), Italy (82%), Slovakia (85%) and Slovenia (91%).

Figure 59: Did you learn anything NEW from the information you got online, or not? (D6) by country

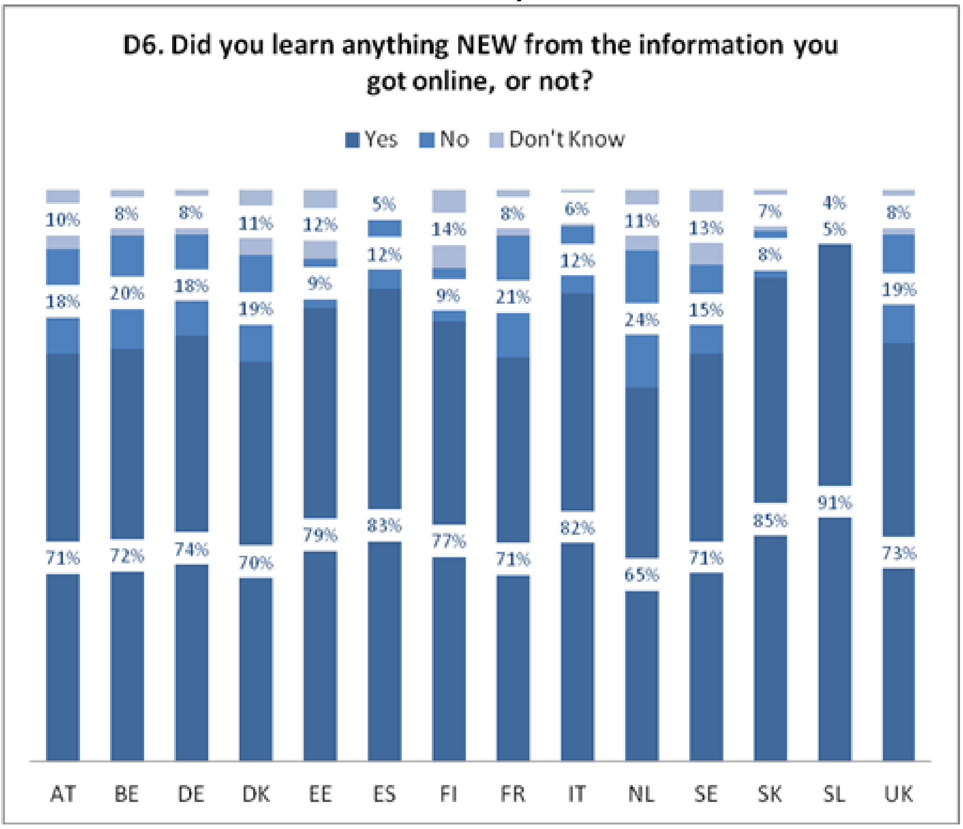

Base: Looked for information about a physical illness or about wellness or lifestyle (88% of whole sample).

The capacity for user interaction with online health information is also an element of this research. In this respect, a little under half of the sampled European citizens (46.8%) had spoken with a doctor or a nurse about information obtained online.

Figure 60: Did you later talk to a doctor or nurse about the information you got online? (D7)

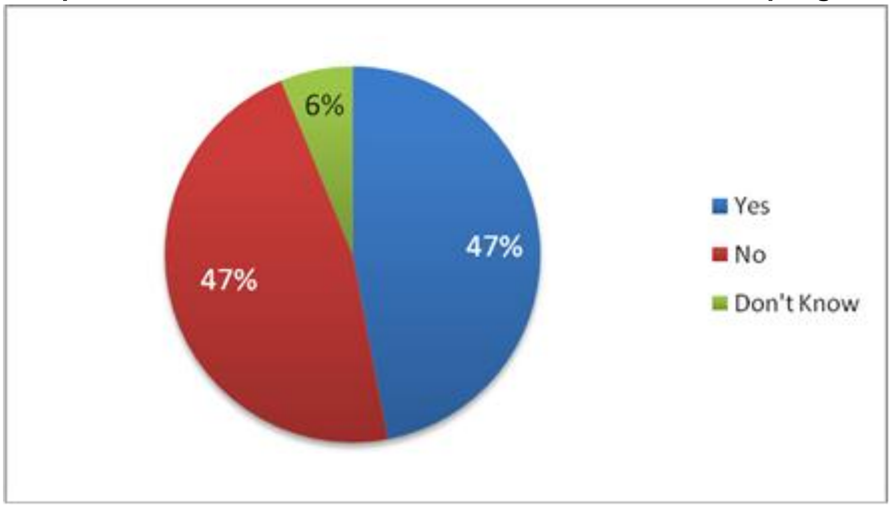

Base: Looked for information about a physical illness or about wellness or lifestyle (88% of whole sample).

Among those that stand out having interacted with health professionals after consulting online medical information are people aged between 25 and 54 years old (48.0%), those with a tertiary education (49.2%), the employed (48.3%) the inactive (47.8%), those from 3 member households (48.6%) and people with long standing illnesses (54,8%). On the other hand, non-interaction with health professionals after consulting for online health information is characterized by people having attained low levels of education (51.8%), the unemployed (48.7%) and students (49.5%), and those without long standing illnesses (52.0%).

Table 64: Did you later talk to a doctor or nurse about the information you got online? (D7) by socio-demographics

		Yes	No	Don't know
Gender	Male	47	46	6
	Female	47	47	6
Age group	16-24	44	46	9
	25-54	48*	47	5
	55-74	46	48	6
Level of education completed	Primary or lower secondary education	42	52*	6
	Upper secondary education	47	47	7
	Tertiary education	49*	45	6
Situation	Employed or self-employed	48*	46	6
	Unemployed	47	49*	5
	Student	41	50*	10
	Other not in the labour force	48*	47	5
Type of locality	Densely-populated area	48	46	7
	Intermediate area	48	46	7
	Thinly-populated area	45	51	5
Members in the household	1	44	49	8
	2	47	47	6
	3	49*	45	6
	4+	48	47	5
Long standing illness	Yes	55*	40	5
	No	42	52*	6

Base: Looked for information about a physical illness or about wellness or lifestyle (88% of whole sample).

On a per-country basis, interaction with professionals with respect to the use of online medical information stands out in Belgium (49.5%), Spain (48.9%), Slovenia (53.0%) and, above all, in Italy (60.5%).

Figure 61: Did you later talk to a doctor or nurse about the information you got online? (D7) by country

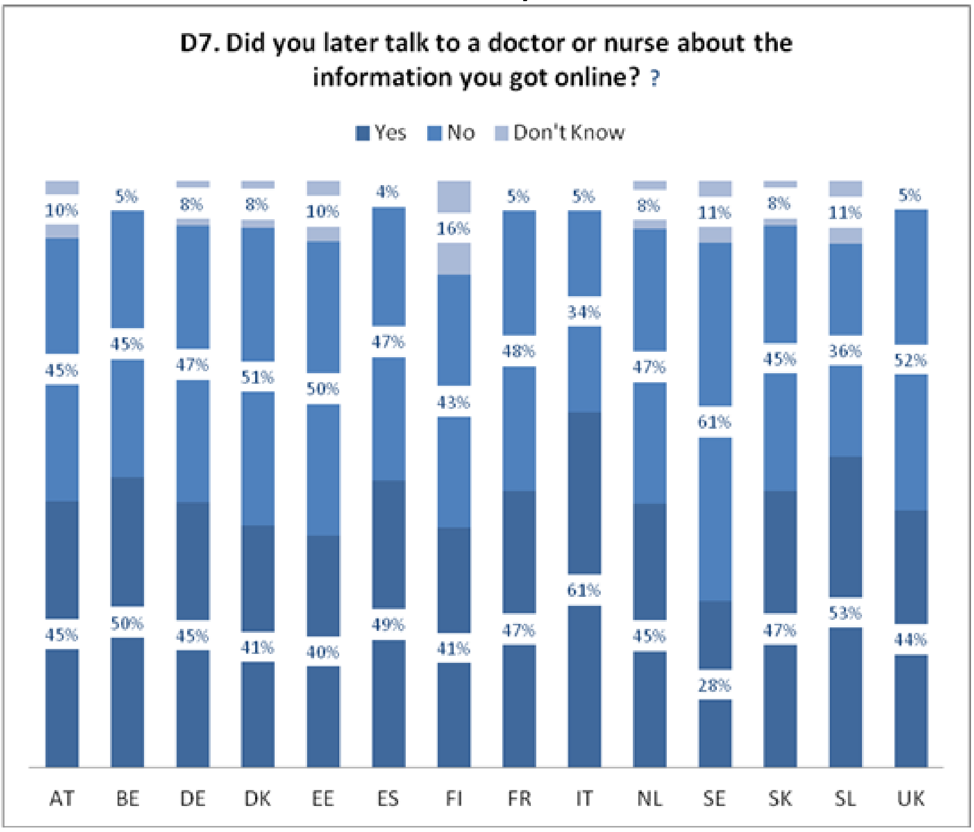

Base: Looked for information about a physical illness or about wellness or lifestyle (88% of whole sample).

Information has also been obtained about whether getting online health information had changed individual decisions about treatments or the way citizens care for themselves. 44.2% of the sampled European population stated that the use of the online medical information affected their decisions about health treatments or the way they take care of themselves.

Figure 62: Did the information you got online affect any of your decisions about health treatments or the way you take care of yourself? (D8)

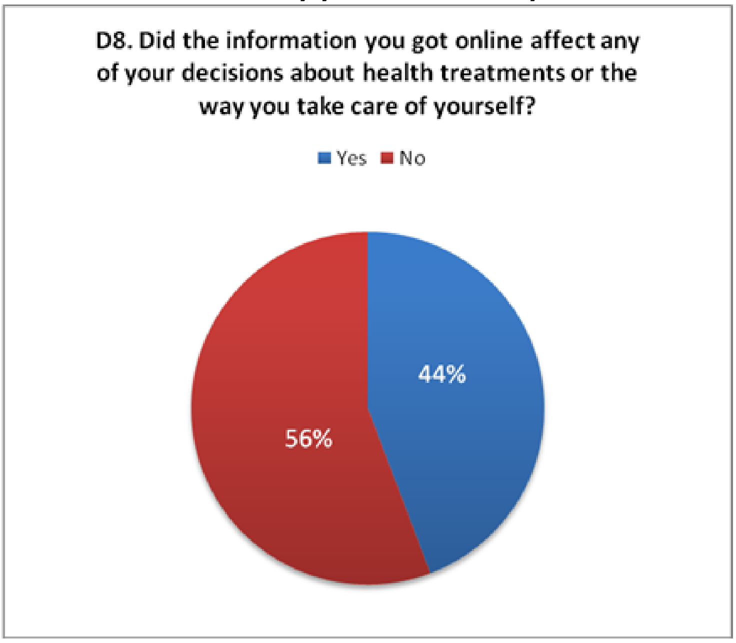

Base: Looked for information about a physical illness or about wellness or lifestyle (88% of whole sample).

The changing of health decisions as a consequence of online medical information is characterized by the young (48.9% of the sampled citizens aged between 16 and 24 years old have changed their health decisions as a result of using e-health information), students (49.9%), those living in densely populated areas (46.7%), households with many members, and people with long standing illnesses (47.3%). With respect to the characteristics of the people that have not changed their health decisions as a result of consulting online medical information, the following stand out: the older population (64.3% of citizens aged between 55 and 74 years old), those with lower education levels (58.8%), the inactive (60.7%), those residing in thinly populated areas (60.1%), households with few members and without long standing illnesses (58.2%).

Table 65: Did the information you got online affect any of your decisions about health treatments or the way you take care of yourself? (D8) by socio-demographics

		Yes	No
Gender	Male	44	56
	Female	44	56
Age group	16-24	49*	51
	25-54	45	55
	55-74	36	64*
Level of education completed	Primary or lower secondary education	41	59*
	Upper secondary education	43	57
	Tertiary education	46	54
Situation	Employed or self-employed	44	56
	Unemployed	44	56
	Student	50*	50
	Other not in the labour force	39	61*
Type of locality	Densely-populated area	47*	53
	Intermediate area	44	56
	Thinly-populated area	40	60*
Members in the household	1	42	58
	2	42	58*
	3	46*	54
	4+	47*	54
Long standing illness	Yes	47*	53
	No	42	58*

Base: Looked for information about a physical illness or about wellness or lifestyle (88% of whole sample).

On a per-country basis, and as is now becoming the norm, Estonia (57.6%), Finland (52.2%), Slovakia (68.4%) and Slovenia (68.2%) stand out for changing their health decisions due to the use of online medical information.

Figure 63: Did the information you got online affect any of your decisions about health treatments or the way you take care of yourself? (D8) by country

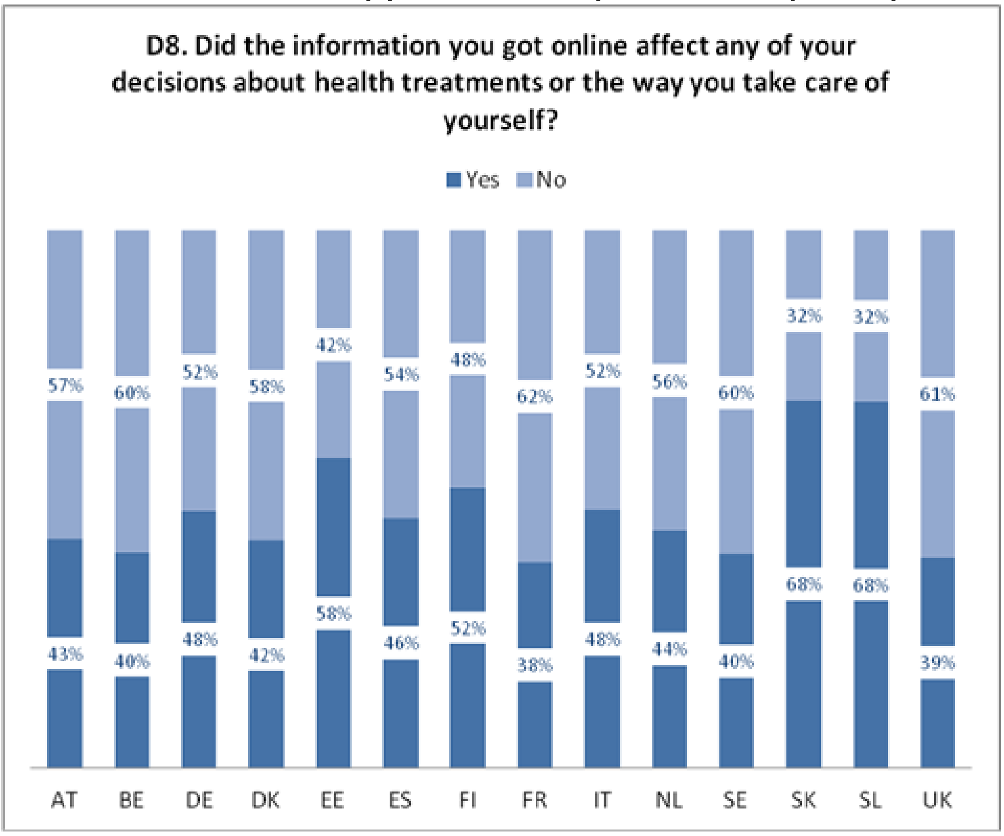

Base: Looked for information about a physical illness or about wellness or lifestyle (88% of whole sample).

Lastly, information has also been collected on whether the use of online health information affects the way the sampled citizens eat or exercise. A little over a third of the European population (37.2%) states that to be the case.

Figure 64: Did the information you got online affect the way you eat or exercise? (D9)

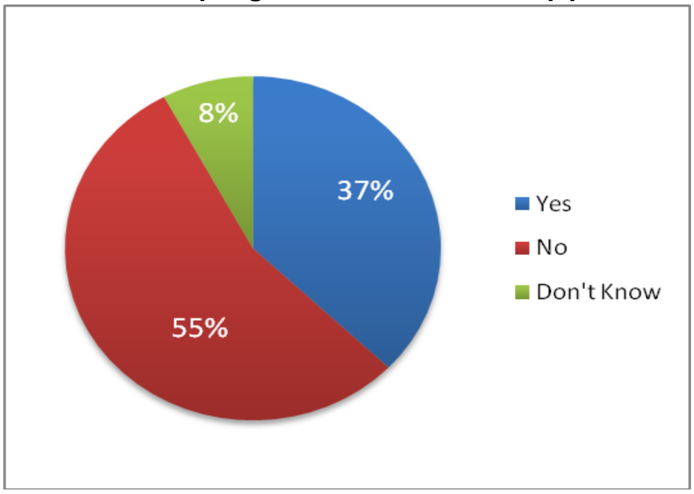

Base: Looked for information about a physical illness or about wellness or lifestyle (88% of whole sample).

Again, the young population (41.2% of the population aged between 16 and 24 years old), those with a tertiary education (40.2%), students (41.2%), and residents of densely populated areas (40.9%) lead the way with respect to changing eating and exercise habits due to the use of online health information. On the other hand, a lack of change of eating and exercise habits due to the use of online information is characterised by the older population (60.6%), the inactive (61.7%), those residing in thinly populated areas (61.7%) and households with few members (58,0%).

Table 66: Did the information you got online affect the way you eat or exercise? (D9) by socio-demographics

		Yes	No	Don't know
Gender	Male	38	54	8
	Female	36	56	8
Age group	16-24	41*	48	11
	25-54	37	56	7
	55-74	33	61*	6
Level of education completed	Primary or lower secondary education	32	61*	7
	Upper secondary education	36	55	9
	Tertiary education	40*	53	7
Situation	Employed or self-employed	38	55	7
	Unemployed	36	55	8
	Student	41*	48	11
	Other not in the labour force	31	62*	7
Type of locality	Densely-populated area	41*	50	9
	Intermediate area	37	56	8
	Thinly-populated area	32	63*	6
Members in the household	1	34	58*	9
	2	36	57	7
	3	38	54	8
	4+	40*	52	9
Long standing illness	Yes	38	55	7
	No	37	55	8

Base: Looked for information about a physical illness or about wellness or lifestyle (88% of whole sample).

On a per-country basis, the change of eating and exercise habits due to e-health information is more effective in Spain (50.3%), Finland (51.2%), Slovakia (53.9%) and Slovenia (58.3%).

Figure 65: Did the information you got online affect the way you eat or exercise? (D9) by country

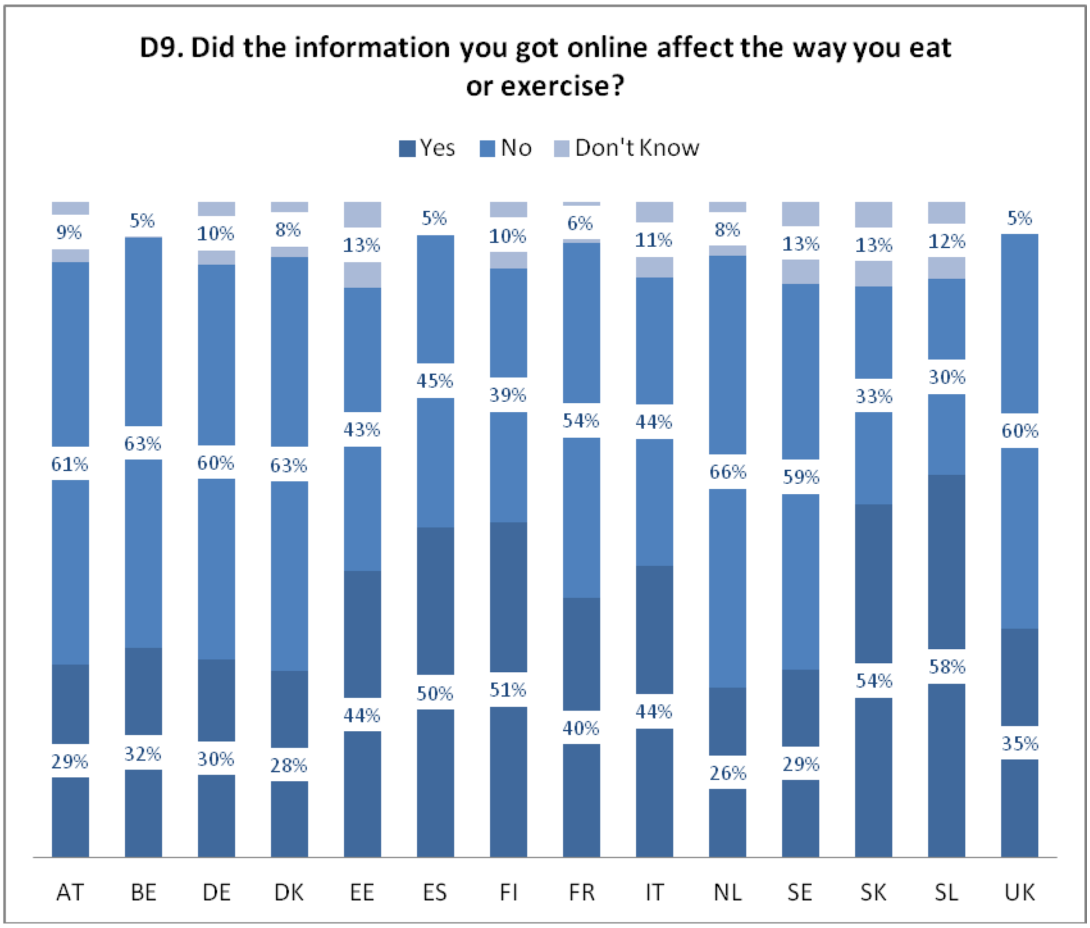

Base: Looked for information about a physical illness or about wellness or lifestyle (88% of whole sample).

9.2 Factors for the evaluation of an Internet health site

The research has also captured the motives considered to be important by the sampled European citizens when it comes to evaluating a health website. 70.2% of the sampled population considers it to be very important that personal information is securely handled, 63.0% that the information is provided in the user's own language, 62.4% that the information should be updated, and 54.1% that health professionals should be involved online. Some distance behind, the population places a high level of importance on the fact that the website clearly states who is responsible for it (39.7%), that there are health organisations involved (36,1%), that there is interactivity (22,4%) and that governments are involved (18.4%).

Figure 66: Internet website evaluation (D12)

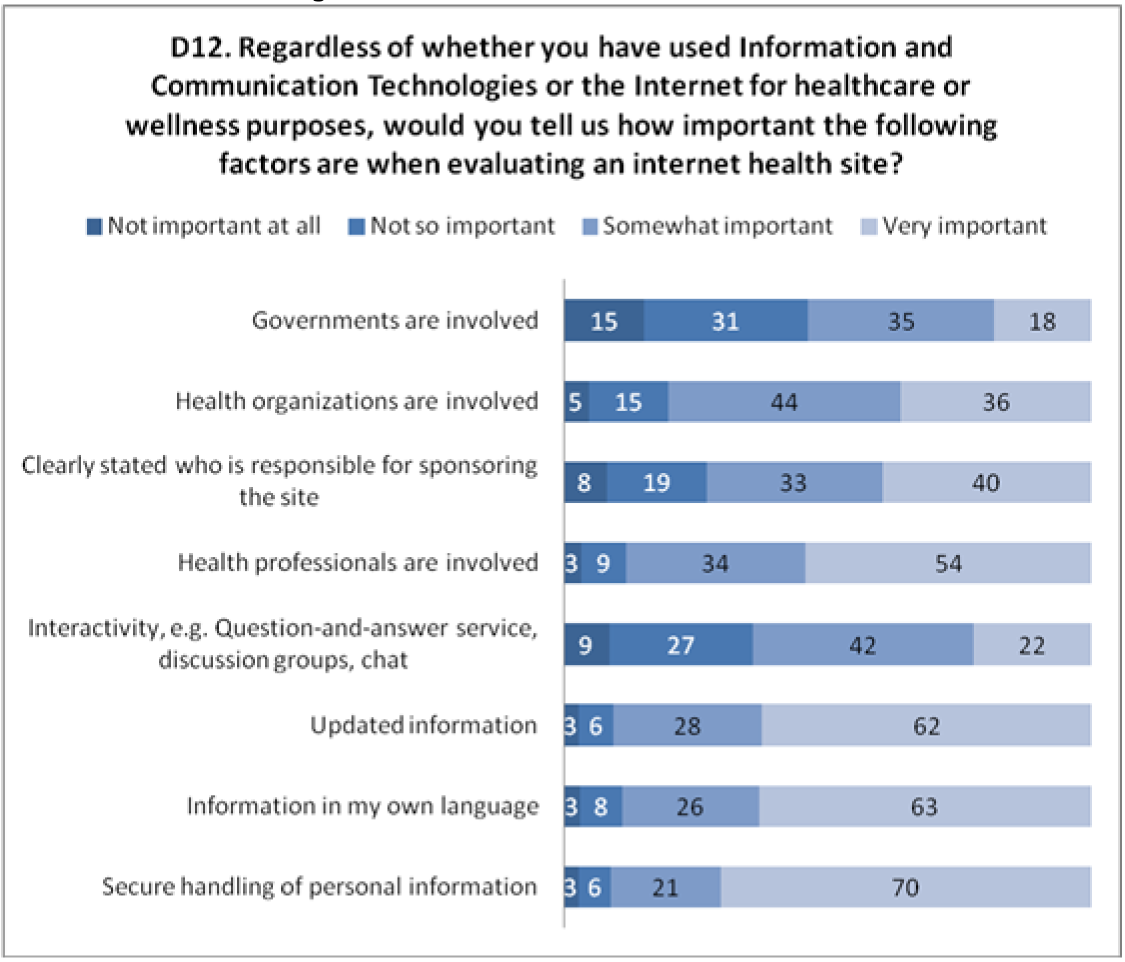

Base: Whole sample.

With respect to the socio-demographic characteristics of the population, women stand out for awarding much more relevance to the defined factors for evaluating a health website (over 90% of women consider personal information, language adaptation and updating as very important). Men only stand out for their preference for government involvement (55.2%). The middle age groups, higher levels of education, population density, and the presence of long standing illnesses are associated with the defined indicators when it comes to assessing the effectiveness of a health website.

Table 67: Internet website evaluation (D12) by socio-demographics

Important (%)		Secure handling of personal information	Information in my own language	Updated information	Interactivity	Health professionals are involved	Clearly stated who is responsible for sponsoring the site	Health organizations are involved	Governments are involved
Gender	Male	90	87	89	63	87	73	79	55*
	Female	92*	91*	92*	65*	90*	73	81*	52
Age group	16-24	88	84	86	67*	84	66	77	56
	25-54	91	90*	92*	66*	89*	74*	81	54
	55-74	93	92*	91	54	90*	78*	82	51
Level of education completed	Primary or lower secondary education	90	89	88	64	86	68	78	50
	Upper secondary education	91	90*	91	64	88	73	80	54
	Tertiary education	91	87	92*	64	89*	74*	82	55
Situation	Employed or self-employed	91	89*	91	64	88	73	80	53
	Unemployed	91	90	91	70*	89	77	81	58*
	Student	89	84	87	68*	85	66	77	52
	Other not in the labour force	93*	92	92	58	89*	77*	82	54
Type of locality	Densely-populated area	92	89	92*	67*	89*	77*	82	56
	Intermediate area	91	90	90	65	88	73	80	55
	Thinly-populated area	90	88	90	58	87	66	77	48
Health status	Bad	93	92	91	65	89	73	79	48
	Neither good or bad	92	91	92	62	91	76	82	54*
	Good	91	88	90	65	87	72	80	54
Long standing illness	Yes	93*	91*	93*	65*	91*	77	82	52
	No	89	87	89	64	86	70	79	55

Base: Whole sample.

On a per-country basis, and as in the previous case, Slovakia, Slovenia and the United Kingdom stand out from other countries in the sample in the majority of the defined indicators when assessing the perceived importance of health websites.

Table 68: Internet website evaluation (D12) by country

Important (%)	AT	BE	DE	DK	EE	ES	FI	FR	IT	NL	SE	SK	SL	UK
Secure handling of personal information	93	88	94	89	95	91	93	86	88	86	91	96	97	96
Information in my own language	88	86	89	83	94	91	90	86	87	85	85	91	89	94
Updated information	91	85	92	89	96	93	93	84	91	85	90	96	97	96
Interactivity, e.g. Question-and-answer service, discussion groups, chat	65	47	66	44	80	79	65	54	73	52	46	79	83	66
Health professionals are involved	88	80	89	85	93	92	91	81	89	84	87	94	96	93
Clearly stated who is responsible for sponsoring the site	65	65	68	72	60	88	64	56	80	77	79	58	75	86
Health organizations are involved	76	73	76	75	90	84	75	71	85	80	82	81	91	90
Governments are involved	34	67	35	40	62	62	43	58	63	77	33	45	49	63

Base: Whole sample.

10. ICT FOR PARTICIPATORY HEALTH

The research has also obtained and assessed information about the attitudes of citizens with respect to health information on the Internet. Specifically, the sampled population was asked what action came out of looking for information about health or illnesses on the Internet. 57.6% of the sample indicated that the health information obtained from the Internet was used to propose suggestions or queries about diagnosis or treatment to the family doctor. 56.6% indicated that they had an increased feeling of reassurance and relief. 54.3% suggested that their willingness to change diet or lifestyle habits improved. 46.7% suggested that they have used online medical information to make, cancel or change an appointment with the family doctor.

Some distance behind, 29.1% of the sampled citizens confirmed that the use of medical information for health improved their feelings of anxiety; and 17.7% of citizens have changed their use of medicine without consulting with their family doctor.

Figure 67: Internet health information consequences (D14)

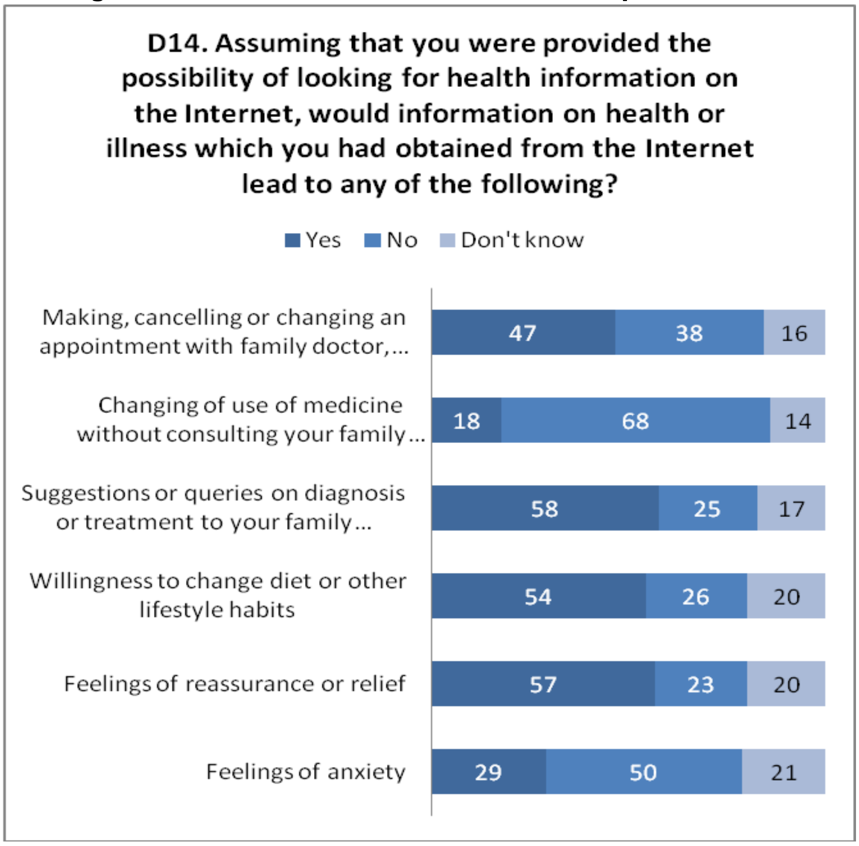

Base: Whole sample.

As is becoming the norm, women are seen to be much more sensitive to changes in attitude as a result of the use of medical information on the Internet, particularly in the proposal of suggestions or queries to the family doctor (60.5%) and in increased feelings of reassurance and relief. Men, however, are more predisposed than women to changing the use of medicine without consulting the doctor as a result of medical information from the Internet (19.0%).

The change in attitudes derived from the use of medical information for health on the Internet is more intense in the youngest population, those with a tertiary education, and those that live in densely populated areas. Lastly, and with respect to state of health and the presence of long standing illnesses, the use of medical information for health from the Internet improves the feeling of anxiety of the population in a bad state of health (32.5%); increases the feeling of reassurance and relief (58.9%) and the willingness to change diet and lifestyle habits (57.0%) in the population with long standing illnesses.

Table 69: Internet health information consequences (D14) by socio-demographics

Yes (%)		Feelings of anxiety	Feelings of reassurance or relief	Willingness to change diet or other lifestyle habits	Suggestions or queries on diagnosis or treatment to your family doctor, specialist or other health professional	Changing of use of medicine without consulting your family doctor, specialist or other health professional	Making, cancelling or changing an appointment with family doctor, specialist or other health professional
Gender	Male	26	53	51	55	19*	45
	Female	33*	60*	58*	61*	16	49*
Age group	16-24	37*	57	55	51	20*	41
	25-54	29	58*	54	60*	18	50*
	55-74	21	52	55	59	12	43
Level of education completed	Primary or lower secondary education	28	54	49	52	19	46
	Upper secondary education	27	56	53	57	17	44
	Tertiary education	32*	58*	58*	61*	19	50
Situation	Employed or self-employed	28*	57	54	58	18	48*
	Unemployed	33	60*	52	61*	17	45
	Student	35*	57	55	50	21*	45
	Other not in the labour force	26	55	55	60	13	45
Type of locality	Densely-populated area	29	58*	56	59*	20*	50*
	Intermediate area	29	56	55	57	17	46
	Thinly-populated area	28	55	51	55	15	43
Health status	Bad	33*	60	57	67	22	49
	Neither good or bad	29	57	54	60	16	47
	Good	29	56	54	56	18	46
Long standing illness	Yes	29	59*	57*	63	19	50*
	No	29	55	53	54	17	45

Base: Whole sample.

On a per-country basis, Estonia, Slovakia and Slovenia lead the way in terms of highest frequency of attitude change with respect to the use of medical information for health. With respect to changes in diet and lifestyles, and the proposal of suggestions and queries to the family doctor as a result of the use of online medical information for health also stands out in Spain and Holland. Lastly, the population from Austria and Germany are among the most willing to change medicine without consulting the family doctor, as a result of using medical information for health from the Internet.

Table 70: Internet health information consequences (D14) by country

Yes (%)	AT	BE	DE	DK	EE	ES	FI	FR	IT	NL	SE	SK	SL	UK
Feelings of anxiety	23	33	23	16	40	30	25	37	26	35	17	26	24	34
Feelings of reassurance or relief	57	55	56	40	75	60	56	58	55	57	46	61	66	58
Willingness to change diet or other lifestyle habits	55	51	53	53	64	62	57	49	49	56	57	66	68	58
Suggestions or queries on diagnosis or treatment to your family doctor, specialist or other health professional	55	59	52	53	64	68	57	54	66	63	61	64	66	55
Changing of use of medicine without consulting your family doctor, specialist or other health professional	22	13	24	14	24	20	14	15	16	15	18	21	25	14
Making, cancelling or changing an appointment with family doctor, specialist or other health professional	59	44	56	39	70	59	62	39	39	54	67	37	53	34

Base: Whole sample.

Lastly, the research has also obtained evidence about the beliefs of the European citizens with respect to the use of ICTs for health. It has to be said that the perceptions are positive overall. 58.3% of the sampled European population state they agree that ICT use for health allows for savings in the cost of travel and time. 55.9% state that they would be willing to share personal health information with the doctor despite the privacy issue. 55.0% state that ICTs for health can improve the possibilities for caring for themselves and monitoring their state of health. 54.5% state they agree with the fact that ICT use for health leads to greater patient satisfaction. 53.5% state they agree that e-health can improve the quality of the medical services received. 50.3% of the European citizens consider that ICT use for health can change their behaviour towards a healthy lifestyle.

Slightly under half of the sample of European citizens, 43.0%, agrees that ICT use for health can improve their state of health. 41.8% consider that they would feel more comfortable and safe if they used a remote monitoring system for their health condition. 41.7% consider that ICT use for health increases ICT use in other fields of daily life. 32.2% agree that the use of health services through the Internet substitutes face-to-face consultations with doctors. 31.6% agree that online health services and face-to-face services are of equal quality. And lastly, 22.8% of European citizens agree that they would be willing to pay for access to Internet health services to improve their state of health or that of their relatives.

Figure 68: ICT for Health consequences (D15)

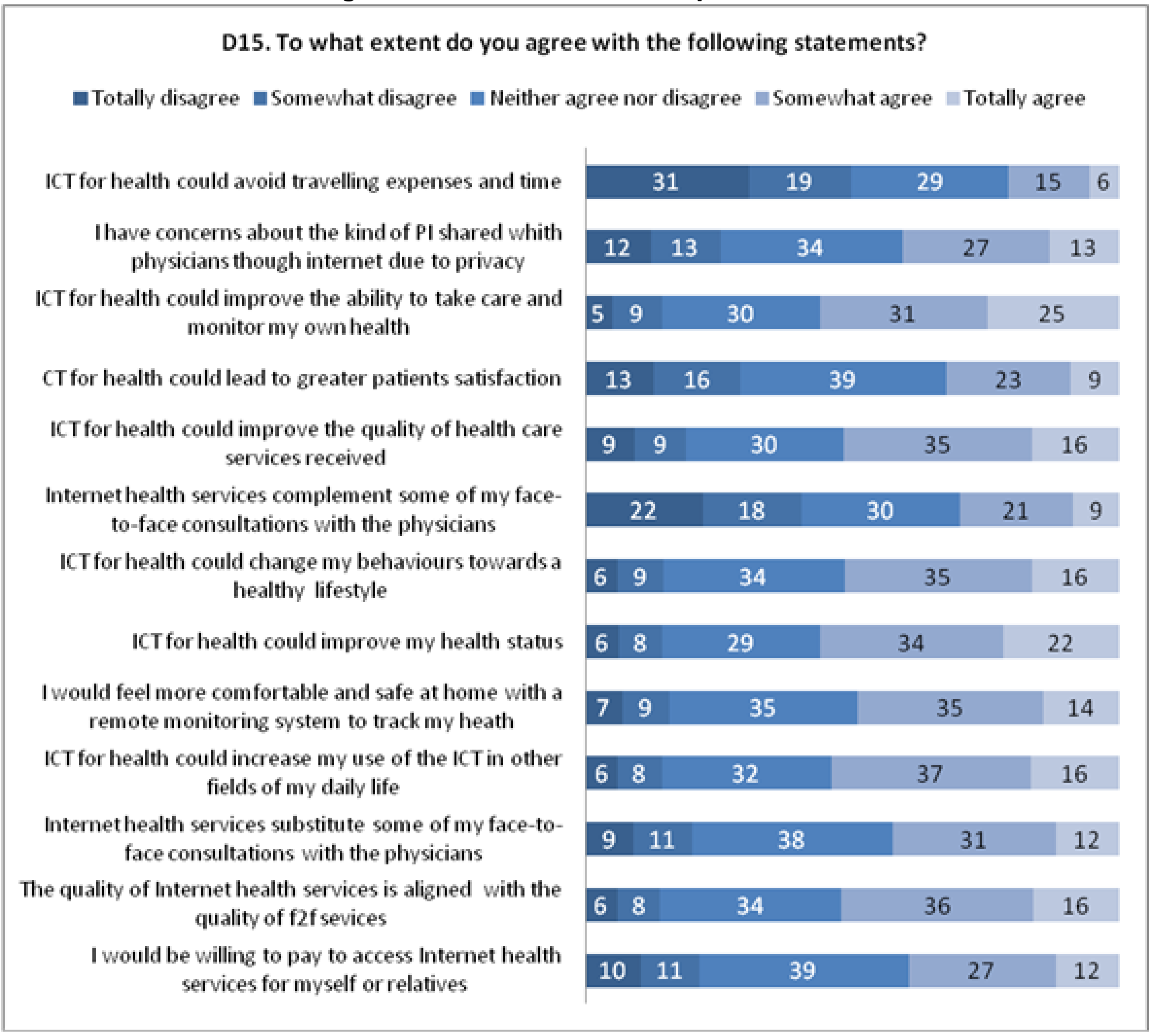

Base: Whole sample.

With respect to socio-demographic characteristics of the population, women are in greater agreement than men that ICT use for health complements face-to-face use (52.7%) and that they are willing to share information with the doctor online despite privacy issues (58.1%). On the other hand, men differ from women on considering ICT use for health improves their state of health (43.2%), they advocate digital monitoring systems for their health condition (42.1%), and are willing to pay to access Internet health systems (22.5%). Positive attitudes towards ICT uses for health are also characterised in the youngest population, those with a tertiary education, and those that live in densely populated areas. With respect to bad states of health, the only notable difference from a good state of health is that ICT use for health can improve the quality of health services received (56.6%). Meanwhile, citizens with long standing illnesses clearly state their favourable perceptions of ICT use for health, with respect to citizens that don't have long standing illnesses. In particular, they state that ICT use can improve patient satisfaction (55.5%), improve caring and health condition monitoring skills (57.4%), save travelling costs and time (59.9%), and that they are willing to share personal information through the Internet with doctors and health organisations despite privacy issues (60,1%).

Table 71: ICT for Health consequences (D15) by socio-demographics

Agree (%)		ICT for health could increase my use of the ICT in other fields of my daily life	ICT for health could lead to greater patients satisfaction	ICT for health could improve my health status	ICT for health could improve the ability to take care and monitor my own health	ICT for health could change my behaviours towards a healthy lifestyle	ICT for health could avoid travelling expenses and time	ICT for health could improve the quality of health care services received
Gender	Male	40	51	43*	53	48	56	52
	Female	38	53	41	54	50	56	51
Age group	16-24	41	51	44*	55	52*	54	49
	25-54	40	53*	44	55*	50*	58*	52*
	55-74	34	48	35	49	44	53	50
Level of education completed	Primary or lower secondary education	39	48	39	48	46	51	48
	Upper secondary education	39	51	42	54	48	55	50
	Tertiary education	40	54*	44	56	51	59	54*
Situation	Employed or self-employed	41*	53*	43	55*	49*	57*	52*
	Unemployed	41	53	44	53	49	58	52
	Student	40	50	44	55	52	56	50
	Other not in the labour force	34	48	37	50	44	52	50
Type of locality	Densely-populated area	42*	55*	45*	56*	52*	60*	55*
	Intermediate area	40	52	43	54	48	55	51
	Thinly-populated area	34	46	36	49	44	51	45
Health status	Bad	42	59	45	61	51	59	57*
	Neither good or bad	39	53	42	53	50	55	52
	Good	39	51	42	53	48	56	51
Long standing illness	Yes	41*	56*	45*	57*	51*	60*	55*
	No	38	50	41	51	47	54	49

Base: Whole sample.

Table 72: ICT for health consequences (D15) by socio-demographics (II)

Agree (%)		Internet health services substitute some of my face-to-face consultations with the physicians	Internet health services complement some of my face-to-face consultations with the physicians	The quality of Internet health services is aligned with the quality of face-to-face services	I have concerns about the kind of personal information shared with physicians or health organizations through the Internet due to privacy and confidentiality issues	In case of need, I would feel more comfortable and safe at home with a remote monitoring system to track my health	I would be willing to pay to access Internet health services for myself or my relatives
Gender	Male	30	50	32	54	42*	23*
	Female	29	53*	33	58*	39	19
Age group	16-24	33*	49	33*	55	39	25*
	25-54	30	54*	32	56	41	21
	55-74	24	47	30	59	42*	15
Level of education completed	Primary or lower secondary education	29	45	34	52	39	18
	Upper secondary education	29	51	31	55	39	20
	Tertiary education	31	54	32	59*	43*	23
Situation	Employed or self-employed	30	53*	32	56	40	22*
	Unemployed	32*	49	33*	56	46*	20
	Student	31	50	32	54	36	23
	Other not in the labour force	26	48	32	59	41	15
Type of locality	Densely-populated area	32*	54*	35*	57	42*	24*
	Intermediate area	31	51	32	56	42	21
	Thinly-populated area	25	47	28	55	36	16
Health status	Bad	32	56	37	59	41	21
	Neither good or bad	28	52	33	57	45*	19
	Good	30	51	32	56	39	21*
Long standing illness	Yes	32	55*	35*	60*	43*	21
	No	29	49	31	53	39	21

Base: Whole sample.

On a per-country basis, clear data is obtained. Estonia, Spain, Slovakia and Slovenia clearly lead from the European countries with respect to the frequency of positive perceptions of the use of the Internet for health.

Table 73: Internet health information consequences (D15) by country

Agree (%)	AT	BE	DE	DK	EE	ES	FI	FR	IT	NL	SE	SK	SL	UK
I would be willing to pay to access Internet health services for myself or relatives	19	15	19	17	33	31	19	15	30	17	22	30	35	18
The quality of Internet health services is aligned with the quality of f2f services	41	18	40	27	36	41	28	15	37	20	18	32	52	38
Internet health services substitute some of my face-to-face consultations with the physicians	30	17	30	34	31	41	35	18	32	20	29	44	54	36
ICT for health could increase my use of the ICT in other fields of my daily life	44	25	41	26	52	59	38	31	46	26	29	66	66	36
I would feel more comfortable and safe at home with a remote monitoring system to track my heath	32	36	34	32	59	55	33	39	57	34	25	56	55	38
ICT for health could improve my health status	45	27	45	28	59	54	41	26	50	31	32	56	58	49
ICT for health could change my behaviours towards a healthy lifestyle	53	35	54	34	69	60	52	34	54	37	36	67	67	54
Internet health services complement some of my face-	55	40	55	42	73	60	53	41	56	43	48	57	69	53

to-face consultations with the physicians														
ICT for health could improve the quality of health care services received	57	36	54	36	66	66	56	38	63	46	42	67	72	50
CT for health could lead to greater patients satisfaction	60	33	53	39	68	65	56	36	59	35	43	77	80	60
ICT for health could improve the ability to take care and monitor my own health	60	37	57	38	73	65	56	40	61	36	43	69	75	61
I have concerns about the kind of PI shared with physicians though internet due to privacy	60	55	58	41	68	65	64	59	43	50	51	58	56	56
ICT for health could avoid travelling expenses and time	62	37	62	44	77	67	63	40	63	44	53	70	77	58

Base: Whole sample.

11. FROM QUESTIONNAIRE ITEMS TO CONCEPTUAL DIMENSIONS: MULTIVARIATE ANALYSIS:

11.1 ICT access dimensions

Following data analysis strategy defined in the Methodology section 2.4 a factor analysis was used to assess 14 Internet-related activities (see Section 5.2) correlations[23] and identify common relationships between similar items, allowing the items to be categorized into various dimensions.

Table 74: Factor analysis - Internet related activities

	Web 2.0 uses	Tech uses	Individual uses	Basic uses
Use a social networking site	**.751**			
Instant messaging, chat websites	**.697**			
Post messages to chatrooms, newsgroups or an online discussion forum	**.663**			
Use websites to share pictures, videos, movies, etc..	**.610**	.417		
Online gaming and/or playing games console	**.571**		.447	-.421
You use the Internet through your mobile phone	**.414**		.406	
Create a web page		**.793**		
Keep a blog (also known as web-log)		**.754**		
Use the Internet to make telephone calls		**.604**		
Use peer-to-peer file sharing for exchanging movies, music,...		**.552**		
Do home banking			**.742**	
Purchase goods or services online / online shopping			**.584**	
Use online software			**.459**	
Send e-mails with attached files				**.740**
Use a search engine to find information				**.659**
Auto values	5.003	1.449	1.252	.884
% Variance explained	33.351	9.661	8.348	5.895

Notes: Rotated components matrix; Sampling method: factor analysis by main components; Rotation method: Varimax with Kaiser-Meyer-Olkin 0,904; Bartlett's test of sphericity p=0.000; Convergence in 8 itenerations; Minimum eigenvalue 0.88.

The factor analysis helped identify the main underlying dimensions of Internet activities. Four factors have emerged: **Basic uses; Individual uses; Web 2.0 uses and Tech uses**. These factors represent a social gradient of Internet activities from the easiest use of the Internet (basic uses) to the most sophisticates activities (tech uses).

---

[23] See Annex 5: Table 89: Internet related activities - Correlation **matrix**.

11.2 ICT for Health Motivation dimensions

11.2.1 Triggers dimensions

Individuals were asked 9 questions about the **triggers** to utilise ICT for Health (see Section 7.1). Factor analysis was performed with all these items.[24] From these items two factors have emerged: **Individual** oriented and **Social and services** oriented.

Table 75: Factor analysis – Triggers

	Individual oriented	Social and services oriented
To better understand a health problem or disease	**0.848**	
To help a family member or friend who is ill	**0.786**	
To find a specific solution to treatment for a health problem	**0.758**	
To develop one's general knowledge or satisfy one's curiosity	**0.724**	
To find additional sources of information	**0.706**	
To prevent diseases by adopting a healthier lifestyle	**0.701**	
To obtain different points of view from those offered by mainstream medicine	**0.599**	0.512
To participate in online discussions		**0.912**
To access an online health service		**0.674**
Auto values	5.139	0.832
% Variance explained	57.099	9.247

Notes: Rotated components matrix; Sampling method: factor analysis by main components; Rotation method: Varimax with Kaiser-Meyer-Olkin 0.932; Bartlett's test of sphericity p=0.000; Convergence in 3 itenerations; Minimum eigenvalue 0.8.

11.2.2 Empowerment dimensions

Empowerment, broadly understood as the development of personal involvement and responsibility, is one of the goals of prevention, promotion and protection in health. This definition assumes that responsibility is a more active form of control while competence refers to aptitudes or qualities that make it possible to be more autonomous and take a role in decision-making. Moreover, there are three different perspectives of personal empowerment, which seems to coexist with respect to Health:

- An aptitude to comply with expert advice (professional perspective)
- Self-reliance through individual choice (consumer perspective)
- Social inclusion through the development of collective support (community perspective)

With these premise, factor analysis was carried out with 18 questions (see Section 7.2) related with empowerment.[25]

[24] See Annex 5: Table 90: Triggers - Correlation **matrix**.

[25] See Annex 5: Table 91: Empowerment - Correlation **matrix**.

Table 76: Factor analysis – Empowerment

	Competence	Control
ICT allow me to develop a better understanding of my personal health…by giving me access to recognized expert knowledge	**.786**	
ICT allow me to better understand my personal health…through my ability to determine what is relevant	**.751**	
ICT allow me to become better informed on what is available…so that I can make my own choices	**.738**	
ICT allow me to be better informed about how to follow the advice of the physician or professionals I consult	**.723**	
ICT allow me to know more about the opinions of people who are in similar situations or who are active in support groups	**.720**	
ICT allow me to better understand my personal health through online discussions or the opinions of people going through similar experiences	**.710**	
ICT allow me to play a more active role in my exchanges with my physician or the health professionals I consult	**.675**	
ICT helps me feel more confident in playing a more active role in my exchanges with my physician…	**.589**	.582
ICT facilitates making decisions about my health on the basis of my preferences and means rather than only on the advice of my physician		**.815**
ICT facilitates a more active role in my health by deciding which solutions I prefer…mainstream medicine or alternative approaches		**.773**
ICT facilitates making decisions about my health by relying on the experiences…with the people with whom I talk		**.769**
ICT facilitates making decisions on my health albeit without going against the advice of the physician….		**.738**
ICT facilitates a more active role in my health by continuing to talk with the people in my life who could help me clarify my ideas		**.707**
ICT helps me feel better equipped to make my own choices without being limited to the advice of a physician…		**.691**
ICT helps me feel more confident about the choices I plan on making between the various possible treatments and solutions		**.653**
ICT helps me feel better equipped to make positive changes to my situation through discussions and exchanges with others		**.597**
ICT helps me feel better equipped to implement the advice of the physician or health professionals I consult		**.583**
ICT helps me feel more confident in my discussions with the people in my life		**.580**
Auto values	11.033	1.047
% Variance explained	61.294	5.816

Notes: Rotated components matrix; Sampling method: factor analysis by main components; Rotation method: Varimax with Kaiser-Meyer-Olkin 0.975; Bartlett's test of sphericity p=0.000; Convergence in 3 iterations; Minimum eigenvalue 1.

With all these items two underlying dimensions have emerged: **control**, which is related with responsibility; and **competence**; which is related with aptitudes and skills.

112.3 Barriers dimensions

Individuals were asked about 10 different types of barriers to utilise ICT for health (see Section 7.3). Factor analysis was performed with all these items.[26] From these items two factors have emerged: **Lack of confidence** and **Lack of Readiness**.

Table 77: Factor analysis – Barriers

	Lack of Confidence	Lack of Readiness
Lack of security	**.858**	
Lack of privacy	**.855**	
Lack of reliability	**.798**	
Lack of trust	**.785**	
Lack of liability	**.676**	
Lack of digital skills		**.833**
Lack of access to ICT for health applications		**.759**
Lack of motivation and interest		**.737**
Lack of awareness		**.718**
Lack of health literacy		**.637**
Auto values	6.158	1.016
% Variance explained	61.585	10.160

Notes: Rotated components matrix; Sampling method: factor analysis by main components; Rotation method: Varimax with Kaiser-Meyer-Olkin 0.946; Bartlett's test of sphericity p=0.000; Convergence in 3 itenerations; Minimum eigenvalue 1.

11.3 Health information sources and trust dimensions

11.3.1 Health information sources dimensions

Individuals were asked about the importance of 10 different information sources related with their health (see Section 6). These items were analysed using factor analysis.[27] This analysis revealed three underlining dimensions: **Traditional media**; **Health professionals**; and **Social media**.

Table 78: Factor analysis – Importance of Health information sources

	Traditional media	Health professionals	Social media
Courses and lectures	.784		
Radio	.677		
Newspapers, magazines	.655		
Books, medical encyclopaedias and leaflets	.639		

[26] See Annex 5: Table 92: Barriers - Correlation **matrix**.

[27] See Annex 5: Table 93: Health information sources - Correlation **matrix**.

Direct face to face contact with doctors		.809	
Direct face to face contact with nurses		.774	
Pharmacies		.666	
Family, friends and colleagues			.737
Internet			.727
TV			.580
Auto values	3.445	1.553	0.925
% Variance explained	34.445	15.532	9.255

Notes: Rotated components matrix; Sampling method: factor analysis by main components; Rotation method: Varimax with Kaiser-Meyer-Olkin 0.814; Bartlett's test of sphericity p=0.000; Convergence in 6 itenerations; Minimum eigenvalue 0.9

11.3.2 Trust dimensions

Individuals were asked to what extend they trust 8 different actors to manage their personal health information (see Section 6). Factor analysis was carried out with all these items.[28] This analysis revealed two main dimensions: **Companies Trust** and **Institutional Trust**:

Table 79: Factor analysis – Importance of Health information sources

	Institutional trust	Companies trust
National public authorities	0.850	
European institutions	0.801	
Health and medical institutions	0.705	
Banks and financial institutions	0.628	
Shops and department stores		0.828
Internet companies		0.818
Phone companies, mobile phone companies and ISP		0.811
Pharmaceutical companies		0.568
Auto values	3.789	1.304
% Variance explained	47.358	16.303

Notes: Rotated components matrix; Sampling method: factor analysis by main components; Rotation method: Varimax with Kaiser-Meyer-Olkin 0.838; Bartlett's test of sphericity p=0.000; Convergence in 3 itenerations; Minimum eigenvalue 1

11.4 ICT for Health access dimensions

11.4.1 ICT for Health readiness dimensions

Individuals were asked about 24 activities related with ICT for Health (see Section 8.1). Factor analysis was performed with all these items,[29] excluding individuals who were not aware of these

[28] See Annex 5: Table 94: Trust - Correlation **matrix**.

[29] See Annex 5: Table 95: ICT for Health readiness - Correlation **matrix**.

types of activities. This analysis revealed two factors or dimensions: **ICT for Health Services and Devices** and **ICT for Health Information and Communication**:

Table 80: Factor analysis – ICT for Health readiness

	ICT for Health Services and Devices	ICT for Health Information and Communication
Made an online consultation through videoconference with your doctor or nurse	.880	
Accessed or uploaded your medical information or health record through an IP	.869	
Accessed or uploaded your medical information or health record through an Internet application provided by your healthcare organization	.866	
Received online the results of your clinical or medical test	.851	
Used a health/wellness application on your mobile phone	.809	
Sent or received an email from your doctor, nurse or healthcare organization	.784	
Used devices to transmit clinical information, received alarms, follow-up about your health anytime, anywhere	.784	
Made, cancelled or changed an appointment with your family doctor, specialist or other health professionals online	.765	
Used a game console to play games related with your health or your wellness	.757	
Received any message about health promotion and/or health prevention	.629	
Looked for information about a physical illness or condition that you or someone you know has		.785
Looked for information about wellness or lifestyle		.773
Participated in Social Networking Sites talking about health and wellness		.737
Kept a health web site "bookmarked", or saved as a "favourite place", so you can go back to it regularly		.722
Described a medical condition or problem online in order to get advice from other online users		.714
Participated in an online support group for people who are concerned about the same health or medical issue		.708
Clicked on a health or medical web site's privacy policy to read about how the site uses PI		.699
Described a medical condition or problem online in order to get advice from an online doctor		.681
Used email or gone to a web site to communicate with a doctor's office		.638
Bought medicine or vitamins online		.576
Auto values	12.825	1.616
% Variance explained	64.124	8.080

Notes: Rotated components matrix; Sampling method: factor analysis by main components; Rotation method: Varimax with Kaiser-Meyer-Olkin 0.980; Bartlett's test of sphericity p=0.000; Convergence in 3 itenerations; Minimum eigenvalue 1.

11.4.2 ICT for Health willingness dimensions

Individuals who answered they were not aware of the ICT for Health activities before mentioned and/or they never used were asked how likely it is that they would carry out these activities during the next year (see Section 8.2). These responses revealed their willingness to use ICT for Health. Factor analysis of these items was performed.[30] The results of this analysis revealed three dimensions: **Web 2.0 uses**; **Services and Devices uses**; **Internet Health Information uses**.

Table 81: Factor analysis – ICT for Health willingness

	Web 2.0 uses	Services and Devices uses	Internet Health Information uses
disclose medical information on websites to share pictures, videos, movies, etc.	0.916		
describe a medical condition or problem online in order to get advice from other online users	0.908		
participate in Social Networking Sites talking about health and wellness	0.907		
disclose medical information on Social Networking Sites	0.904		
look to see what company or organization is providing the advice or information that appears on a health website	0.876		
participate in an online support group for people who are concerned about the same health or medical issue	0.874		
describe a medical condition or problem online in order to get advice from an online doctor	0.851		
look for information about a mental health issue like depression or anxiety	0.838		
keep a health web site "bookmarked", or save as a "favourite place", so you can go back to it regularly	0.832		
click on a health or medical web site's privacy policy to read about how the site uses PI	0.796		
buy medicine or vitamins online	0.706		
use email or go to a web site to communicate with a doctor's office	0.702		
look for information about wellness or lifestyle	0.651		
Access or upload your medical information or health record through an IP		0.877	
Receive any message about health promotion and/or health prevention		0.875	
Use a health/wellness application on your mobile phone		0.874	
Make an online consultation through videoconference with your doctor or nurse		0.871	
Use devices to transmit clinical information, receive alarms, follow-up about your health anytime, anywhere		0.870	
Access or upload your medical information or health record through an Internet application provided by		0.865	

30 See Annex 5: Table 96: ICT for Health willingness - Correlation **matrix**.

your healthcare organization			
Use a game console to play games related with your health or your wellness		0.823	
Receive online the results of your clinical or medical test		0.788	0.422
Send or receive an email from your doctor, nurse or healthcare organization		0.768	0.442
Make, cancel or change an appointment with your family doctor, specialist or other health professionals online		0.678	0.527
Look for information about a physical illness or condition that you or someone you know has			0.576
Auto values	14.229	3.994	1.026
% Variance explained	59.287	16.641	4.276

Notes: Rotated components matrix; Sampling method: factor analysis by main components; Rotation method: Varimax with Kaiser-Meyer-Olkin 0.958; Bartlett's test of sphericity p=0.000; Convergence in 5 itenerations; Minimum eigenvalue 1.

11.4.3 ICT for Health assessment dimensions

Individuals were asked about their preferences to evaluate a health website (see Section 9.2). Factor analysis was carried out with seven items included in this question.[31] This analysis revealed two underling dimensions: **Information and professionals** and **Interaction and organisations**.

Table 82: Factor analysis – ICT for Health willingness

	Information and professionals	Interaction and organisation
Updated information	**0.848**	
Secure handling of PI	**0.843**	
Information in my own language	**0.799**	
Health professionals are involved	**0.756**	
Governments are involved		**0.899**
Health organizations are involved		**0.677**
Clearly stated who is responsible for sponsoring the site		**0.625**
Auto values	3.918	1.032
% Variance explained	55.973	14.744

Notes: Rotated components matrix; Sampling method: factor analysis by main components; Rotation method: Varimax with Kaiser-Meyer-Olkin 0.878; Bartlett's test of sphericity p=0.000; Convergence in 3 itenerations; Minimum eigenvalue 1

[31] See Annex 5: Table 97: ICT for Health assessment - Correlation **matrix**.

11.4.4 ICT for Health impact dimensions

Individuals were asked 12 questions about their perception on ICT for Health impact (see Section 10). These items were analysed using Factor analysis.[32] Results revealed two dimensions: **Quality of healthcare and Healthy behaviours** and **Healthcare access**

Table 83: Factor analysis – ICT for Health willingness

	Quality of healthcare and Healthy behaviours	Healthcare access
ICT for health could improve the ability to take care and monitor my own health	.826	
ICT for health could improve the quality of health care services received	.798	
ICT for health could lead to greater patients satisfaction	.793	
ICT for health could change my behaviours towards a healthy lifestyle	.775	
ICT for health could avoid travelling expenses and time	.750	
ICT for health could improve my health status	.722	
ICT for health could increase my use of the ICT in other fields of my daily life	.646	.
Internet health services complement some of my face-to-face consultations with the physicians	.641	
I would feel more comfortable and safe at home with a remote monitoring system to track my heath	.487	.486
I would be willing to pay to access Internet health services for myself or relatives		.846
Internet health services substitute some of my face-to-face consultations with the physicians		.749
The quality of Internet health services is aligned with the quality of f2f sevices	.	.643
Auto values	6.808	0.943
% Variance explained	56.736	7.856

Notes: Rotated components matrix; Sampling method: factor analysis by main components; Rotation method: Varimax with Kaiser-Meyer-Olkin 0.958; Bartlett's test of sphericity p=0.000; Convergence in 3 itenerations; Minimum eigenvalue 0.9

[32] See Annex 5: Table 98: ICT for Health impact - Correlation **matrix**.

12. CONCLUSIONS

Factor analyses described in Section 11 were carried out following our 1.3 Conceptual framework: towards a social determinants of ICT for Health (see Section 1.3.) This analytical exercise has facilitated the synthesis of questionnaire items gathered into underlying dimensions or concepts. Figure 69 summarised all the dimensions:

Figure 69: Dimensions of Social determinants of ICT for Health

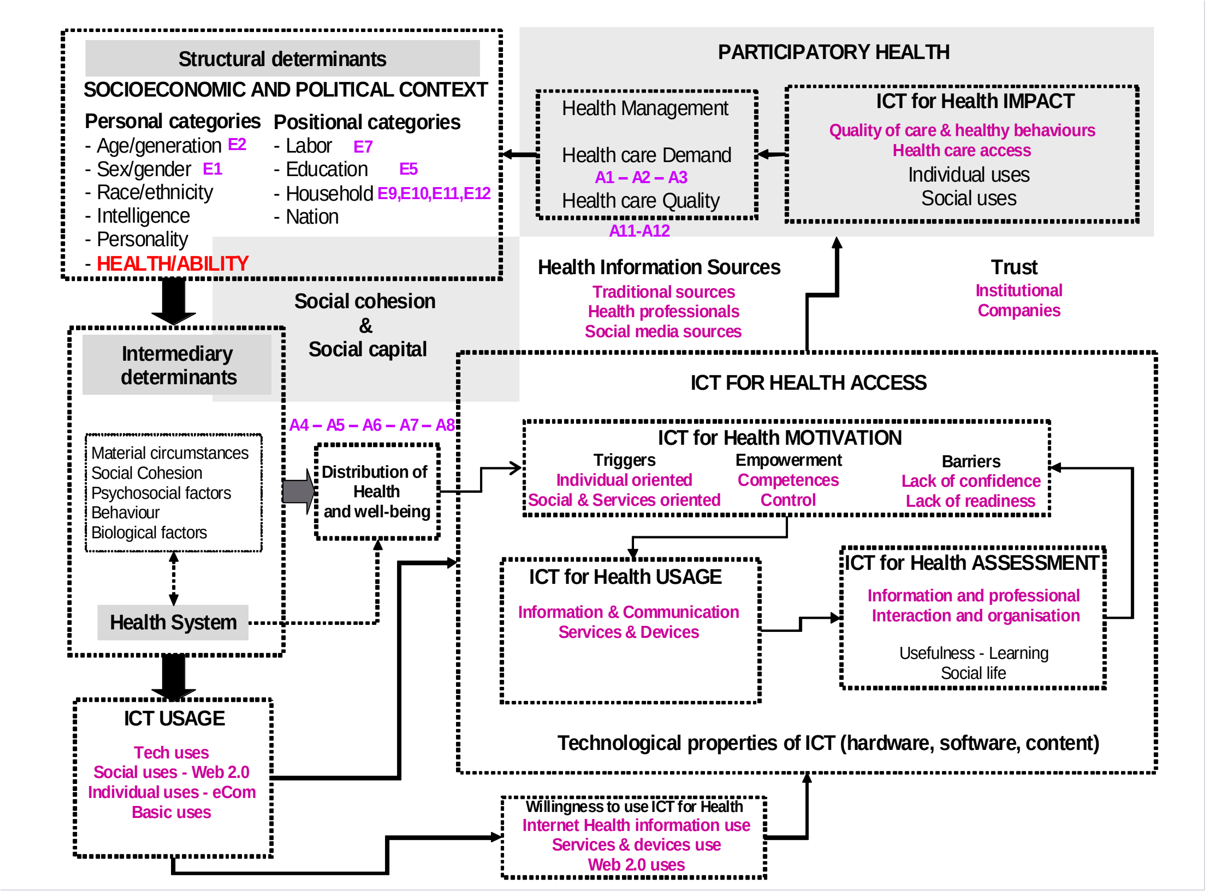

Structural and intermediary determinants of Health also produce different levels of **ICT usage** from Tech uses to Basis uses. This typology of uses represents an unequal access to ICT which will generate different levels of **ICT for Health Access** as well as different levels of **willingness to use ICT for Health**. Both blocks could be analysed in-depth detail. On the one hand, three different dimensions of willingness have been identified: Internet Health Information, Web 2.0 uses and Services and devices uses. These dimensions represent different level of complexity: from basic use of Internet Health information to the complex ecosystem of Services and devices. On the other hand, ICT for Health Access is comprised of three different blocks. Firstly, **ICT for Health Motivation** split up into three concepts with their related dimensions: Triggers (individual oriented and social and services oriented); Empowerment (competence oriented and control oriented) and Barriers (lack of confidence and lack of readiness). Secondly, **ICT for Health Usage** made up of Information and Communication usage and Services and Devices usage. Thirdly, **ICT for Health Assessment** tackled how individuals evaluate websites paying special attention to information and professionals involved and interaction and organisation involved.

The interrelationship between these three blocks gave rise to different level of **Participatory Health** through the individual and social use of ICT for Health and their impacts perceived. These

impacts could be related with health management; healthcare demand or healthcare quality and, moreover, could have the potential to modify both structural and intermediary determinants and distribution of health and well-being.

All above mentioned unveiled the complexity of ICT for Health. To tackle this complexity, correlation analyses of all dimensions have been performed. The main results of these analyses are summarised in the following figure:

Figure 70: Complexity of Social determinants of ICT for Health dimensions

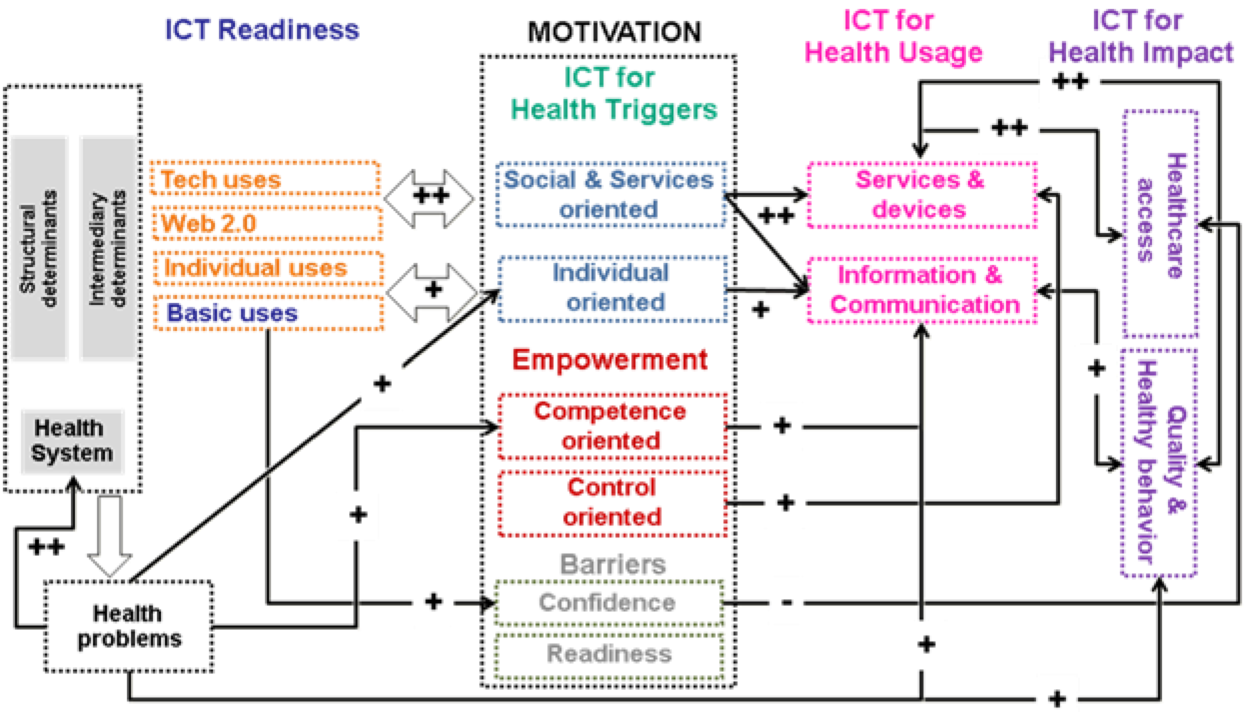

- Social determinants of Health (structural and intermediary), especially education and age, produces different levels of ICT readiness. Advance uses of the Internet such as Tech and Web 2.0 uses are more likely to be carried out by the young, the healthy and the well-educated population while basic uses are mostly performed by the elderly, therefore individuals with worse health status (chronic patients and individuals having reported higher numbers of health problems).

- Unequal ICT readiness generates different levels of motivation. Individuals making more advance uses are triggered by the potential of ICT to facilitate social interaction and services related to health while individuals whose uses are basic or individual are triggered mainly by Internet health information for personal proposes. Furthermore, individuals with the lowest level of readiness (basic uses) and having reported more health problems lack confidence in the use of ICT for Health. Nevertheless, this lack of confidence is counterbalanced by a higher level of empowerment (competence oriented).

- Both ICT for Health usages (Services and Devices and Information and Communication) are specially driven by social and services triggers while individual triggers are only slightly correlated with Information and Communication usages, therefore less advanced uses.

- Both dimensions of Empowerment push ICT for Health usage. Individuals who are more competence-oriented are more inclined to Information and Communication usage while individuals who are more control-oriented are more likely to use Services and Devices. Thus individuals who feel more responsible for their health status are more likely to use Services and Devices while individuals who want to be more autonomous (competence refers to aptitudes or qualities that make it possible to be more autonomous) are more likely to

utilise Information and Communication. If we consider individuals' education, age and health status it looks like Services and Devices are related with well-being and wellness practice, therefore with health prevention and promotion while Information and Communication are more related with illness, therefore with cure and independent living

- All individuals using ICT for Health faced the same barriers; therefore lack of confidence and lack of readiness are not correlated significantly with ICT for Health usages. Nevertheless, lack of confidence is negatively correlated with the ICT for Health impact on the access dimension. Individuals need a certain level of confidence in ICT for Health to go beyond information and communication and engage with services such as RMT, Personal Health Records or videoconference consultation.
- The utilisation of Services and devices is strongly correlated with the perception that ICT would have an impact on both healthcare access and quality and healthy behaviours while the utilisation of Information and Communication is slightly correlated with Quality and healthy behaviours only.
- The number of health problems reported by individuals is only slightly correlated with Information and Communication Usage and it is unrelated to Services and devices utilisation. Therefore, individuals who could take more advantage of Services and devices, due to their health status, are more likely to be oriented towards information and communication usage only.

The study reported here reveals the potential of ICT for Health **to promote active and healthy individuals** and increase empowerment. Even though our findings relate to Internet users, it is worth pointing out that new health inequalities are emerging due to the impact of the "traditional determinants of heath" on ICT readiness.

Therefore, eInclusion policies related to ICT for Health are needed to ensure that individuals with low socio-economic status and more health problems are able to benefit from these types of technologies. These ICT for Health divides specially impact on the elderly. However, there is an opportunity for them to engage with the Information Society through ICT for Health due to the importance of health issues in their daily life.

The relationship between the different typologies of ICT readiness and ICT for Health Motivation and Impact reveal that:

- Young individuals are already using this type of technologies mostly in relation with wellness and healthy life style. These uses enable an entire world of possibilities related with **health promotion and prevention**, especially considering that young individuals are heavy Web 2.0 users.
- Middle age individuals are also active users of ICT for Health acting as **gatekeepers of this type of technologies within the household**. Therefore these individuals could act as enablers for others i.e. both for the elderly and the young within households
- The elderly are basically using ICT for Health for information and communication purposes. There is a gap between this type of use and services and devices uses which could be more effective in relation with **cure and chronic conditions**.

Individuals between 16-54 with chronic conditions, going under long-term treatment and with more than one health problems are more likely to use ICT for Health than individuals without these type of health problems. Individuals between 55-74 who are healthy are more likely to use ICT for Health, especially for Information and Communication, than individuals with worse health status. Therefore, in the short term, this group of individuals will be pushing for health systems to provide them with new solutions (services and devices) when they need to tackle a health problem. This pressure will increase during the next decade when middle age individuals become elderly. Therefore health systems are facing the **challenge of having to promote further ICT**

innovation to answer these new demands. While this is an opportunity to improve both sustainability and efficiency of healthcare system, it is associated with a number of challenges linked to eHealth deployment.

However, during this transition, health systems cannot leave out the elderly, who are not active and healthy. This group of individuals, who are the current intensive users of healthcare systems, cannot be omitted. There is an **opportunity to include** them in the Information Society by **improving ICT readiness and ICT for Health willingness and awareness**.

REFERENCES

1. European Commission, *e-Health - making healthcare better for European citizens: An action plan for a European e-Health Area*. 2004, European Commission: COM (2004) 356 final, Brussels.

2. European Commission, *Accelerating the development of the eHealth market in Europe. eHealth Taskforce report 2007: Composed in preparation for the Lead Market Initiative*. 2007, European Commission: Luxembourg.

3. European Commission, *eEurope, an Information Society for All, Communication on A Commission Initiative for the Special European Council of Lisbon, 23-24 March 2000*. 1999, European Commission: COM(1999) 687 final, Brussels.

4. European Council and Commission, *eEurope 2002, an Information Society for All: Action Plan prepared by the Council and the European Commission for the Feira European Council*. 2000: Brussels.

5. European Commission, *eEurope 2005, An information society for all: An Action Plan to be presented in view of the Seville European Council*. 2002, European Commission: COM(2002) 263 final, Brussels.

6. European Commission, *i2010 - A European Information Society for growth and employment*. 2005: COM (2005) 229 final, Brussels.

7. European Commission, *A Digital Agenda for Europe*. 2010, European Commission: COM (2010)245 final, Brussels.

8. European Commission, *Europe 2020. A European Strategy for smart, sustainable and inclusive growth*. 2010, European Commission: COM(2010) 2020 final, Brussels.

9. Lapointe, L., M. Mignerat, and I. Vedel, *The IT productivity paradox in health: a stakeholder's perspective.* Int J Med Inform, 2011. 80(2): p. 102-15.

10. Plantenga, J. and J. Hansen, *Assessing equal opportunities in the European Union.* International Labour Review, 1999. 138(4): p. 351-379.

11. World Health Organization., *A conceptual framework for action on the social determinants of health*. Discussion Paper Series on Social Determinants of Health,. 2010, Geneva: World Health Organization. 76 p.

12. van Dijk, J.A.G.M., *The Deeping Divide: Inequalities in the Information Society*. 2005, Thousand Oaks, CA: Sage

13. Austin, T.M., R.R. Richter, and M.F. Reinking, *A primer on web surveys.* J Allied Health, 2008. 37(3): p. 180-6.

14. Wyatt, J.C., *When to use web-based surveys.* J Am Med Inform Assoc, 2000. 7(4): p. 426-9.

15. Eysenbach, G. and J. Wyatt, *Using the Internet for surveys and health research.* J Med Internet Res, 2002. 4(2): p. E13.

16. Mann, C.J. and F. Stewart, *Internet communication and qualitative research : a handbook for researching online*. 2000, London: SAGE. 256p.

17. Dillman, D.A., et al., *Internet, mail, and mixed-mode surveys : the tailored design method*. 3rd ed. / Don A. Dillman, Jolene D. Smyth and Leah Melani Christian. ed. 2009, Hoboken, N.J.: Wiley ; Chichester : John Wiley [distributor]. xii, 499 p.

ANNEXES

Annex 1. Questionnaire and coding manual

We are currently conducting an International research study on behalf of the Institute for Prospective Technological Studies (IPTS), one of the seven scientific institutes of the European Commission's Joint Research Centre (JRC). The objective of the study is to analyze the use of Information and Communication Technologies (ICT), specially the Internet, for healthcare purposes. In this regard, we would like to ask for 20 minutes of your time to complete this survey. We would very much appreciate your opinion.

Please rest assured the survey is anonymous and the data gathered strictly confidential.

Block A: Health status and health care and social care services use

A1. How many times did you visit a doctor during the last 12 months? (include hospitalisation or visits to the outpatient department; do not include visits to the dentist)

Number:_______________	A1

A2. How many times have you received a doctor or a nurse at home during the last 12 months?

Number:_______________	A2

A3. How many times have did you visit or received a visit of a social care worker during the last 12 months?

Number:_______________	A3

A4. How is your health in general?

	A4
Very good	5
Good	4
Neither good or bad	3
Bad	2
Very bad	1

A5. Do you have any long-standing illness or health problem?

	A5
Yes	1
No	2
Don’t Know	99

A6. Are you undergoing a long-term medical treatment?

	A6
Yes	1
No	2
Don't Know	99

A7. Over the past 6 months, to what extent, if at all, have you been limited in activities people normally do, because of a health problem. Would you say you have been...?

	A7
Severely limited	1
Somewhat limited	2
Not limited at all	3

A8. Do you have or have you ever had any of the following health problems?

	Yes	No	
Diabetes	1	2	A8_1
An allergy	1	2	A8_2
Asthma	1	2	A8_3
Hypertension (high blood pressure)	1	2	A8_4
Long-standing troubles with your muscles, bones and joints (rheumatism, arthritis)	1	2	A8_5
Cancer	1	2	A8_6
Cataract	1	2	A8_7
Migraine or frequent headaches	1	2	A8_8
Chronic bronchitis, emphysema	1	2	A8_9
Osteoporosis	1	2	A8_10
Stroke, cerebral haemorrhage	1	2	A8_11
Peptic ulcer (gastric or duodenal ulcer)	1	2	A8_12
Chronic anxiety or depression	1	2	A8_13

A9. Is someone close to you, currently experiencing long-term illness or disability?

	A9
Yes	1
No	2
Don't know	99

If A9 = 1 -> A10

If A9 =2 or A9 =99 -> A11

A10. Are you taking care of such a person?

	A10
Yes	1
No	2

A11. In general, how often does your usual source of care (doctor or nurse)...

	Always	Often	Sometimes	Rarely	Never	
...explain to you the results of medical exams (laboratory, radiology, etc.)?	5	4	3	2	1	A11_1
...explain to you different treatment options?	5	4	3	2	1	A11_2
...listen to your opinion and take your preferences into account to choose treatments?	5	4	3	2	1	A11_3

A12. In general, how often do you ask your usual source of care (doctor or nurse)...

	Always	Often	Sometimes	Rarely	Never	
... to explain to you the results of the medical exams?	5	4	3	2	1	A12_1
... to explain to you the different treatment options?	5	4	3	2	1	A12_2
... to consider your opinion and your preferences when choosing treatments?	5	4	3	2	1	A12_3

Block B: Health attitude and Health information sources

B1. For each of the following statements regarding the use of Information and Communication Technologies, specially the Internet, could you please tell me whether you agree or disagree?

Information and Communication Technologies, specially the Internet, allow me to…

	Totally agree	Somewhat agree	Neither agree nor disagree	Somewhat disagree	Totally disagree	
be better informed about how to follow the advice of the physician or professionals I consult	5	4	3	2	1	B1_1
develop a better understanding of my personal health or that of a family member or friend by giving me access to recognized expert knowledge	5	4	3	2	1	B1_2
become better informed on what is available, such as the available solutions and treatments, so that I can make my own choices	5	4	3	2	1	B1_3
better understand my personal health or that of a family member or friend through my ability to determine what is relevant	5	4	3	2	1	B1_4
know more about the opinions of people who are in similar situations or who are active in support groups	5	4	3	2	1	B1_5
better understand my personal health or that of a family member or friend through online discussions or the opinions of people going through similar experiences	5	4	3	2	1	B1_6
play a more active role in my exchanges with my physician or the health professionals I consult	5	4	3	2	1	B1_7

B2. For each of the following statements regarding the use of Information and Communication Technologies, specially the Internet, could you please tell me whether you agree or disagree? ICT, specially the Internet, helps me feel …

	Totally agree	Somewhat agree	Neither agree nor disagree	Somewhat disagree	Totally disagree	
better equipped to implement the advice of the physician or health professionals I consult	5	4	3	2	1	B2_1
better equipped to make my own choices, without being limited to the advice of a physician or health professionals, which I believe is the best approach	5	4	3	2	1	B2_2
better equipped to make positive changes to my situation or that of a family member or friend through discussions and exchanges with others (in my family, at work, on the Internet, etc.)	5	4	3	2	1	B2_3
more confident in playing a more active role in my exchanges with my physician or the health professionals I consult	5	4	3	2	1	B2_4
more confident about the choices I plan on making, on my own, between the various possible treatments and solutions	5	4	3	2	1	B2_5
more confident in my discussions with the people in my life (my family, people at work or on the Internet, etc.)	5	4	3	2	1	B2_6

B3. For each of the following statements regarding the use of Information and Communication Technologies, specially the Internet, could you please tell me whether you agree or disagree? ICT, specially the Internet, facilitates…

	Totally agree	Somewhat agree	Neither agree nor disagree	Somewhat disagree	Totally disagree	
making decisions on my health albeit without going against the advice of the physician or	5	4	3	2	1	B3_1

the health professionals I have consulted						
a more active role in my health by deciding which solutions I prefer, whether from mainstream medicine or alternative approaches	5	4	3	2	1	B3_2
making decisions about my health on the basis of my preferences and means rather than only on the advice of my physician	5	4	3	2	1	B3_3
a more active role in my health by continuing to talk with the people in my life who could help me clarify my ideas	5	4	3	2	1	B3_4
making decisions about my health by relying on the experiences and points of view of the people with whom I talk (on the Internet, at work, in my family, etc.)	5	4	3	2	1	B3_5

B4. Below you can find a list of various sources of information about health, illness or wellness, and we would like to know how important these are to you.

	Very important	Somewhat important	Not so important	Not important at all	
Internet	4	3	2	1	B4_1
TV	4	3	2	1	B4_2
Radio	4	3	2	1	B4_3
Books, medical encyclopaedias and leaflets	4	3	2	1	B4_4
Courses and lectures	4	3	2	1	B4_5
Newspapers, magazines	4	3	2	1	B4_6
Family, friends and colleagues	4	3	2	1	B4_7
Pharmacies	4	3	2	1	B4_8
Direct face-to-face contact with doctors	4	3	2	1	B4_9
Direct face-to-face contact with nurses	4	3	2	1	B4_10

B5. Different authorities (government departments, local authorities, agencies) and private companies could offer health information and online services related with your health. To what extent do you trust the following institutions to protect your personal information?

	Trust fully	Trust somewhat	Trust little	Do not trust	
National public authorities (e.g. tax authorities, social security authorities)	4	3	2	1	B5_1
European institutions (European Commission, European Parliament, etc.)	4	3	2	1	B5_2
Banks and financial institutions	4	3	2	1	B5_3
Health and medical institutions	4	3	2	1	B5_4
Shops and department stores	4	3	2	1	B5_5
Internet companies (Search Engines, Social Networking Sites, E-mail Services)	4	3	2	1	B5_6
Phone companies, mobile phone companies and Internet Services Providers	4	3	2	1	B5_7
Pharmaceutical companies	4	3	2	1	B5_8

Block C: Internet and Information and Communication Technologies, uses

C1. Could you tell me if…?

	Every day or almost every day	At least once a week (but not every day)	At least once a month (but not every week)	Less than once a month	Never	
You use the Internet in your home	5	4	3	2	1	C1_1
You use the Internet at your place of work	5	4	3	2	1	C1_2
You use the Internet somewhere else (school, university, cyber-café, etc.)	5	4	3	2	1	C1_3

C2. Which of the following Internet related activities have you already carried out?

	Every day or almost every day	At least once a week (but not every day)	At least once a month (but not every week)	Less than once a month	Never	
Use a search engine to find information	5	4	3	2	1	C2_1
Send e-mails with attached files (documents, pictures, etc.)	5	4	3	2	1	C2_2
Post messages to chatrooms, newsgroups or an online discussion forum	5	4	3	2	1	C2_3
Use the Internet to make telephone calls	5	4	3	2	1	C2_4
Use peer-to-peer file sharing for exchanging movies, music, etc	5	4	3	2	1	C2_5
Create a web page	5	4	3	2	1	C2_6
Use websites to share pictures, videos, movies, etc.	5	4	3	2	1	C2_7
Use a social networking site	5	4	3	2	1	C2_8
Purchase goods or services online / online shopping (e.g. travel & holiday, clothes, books, tickets, films, music, software, food)	5	4	3	2	1	C2_9
Keep a blog (also known as web-log)	5	4	3	2	1	C2_10
Instant messaging, chat websites	5	4	3	2	1	C2_11
Do home banking	5	4	3	2	1	C2_12
Use online software	5	4	3	2	1	C2_13
Use the Internet through your mobile phone	5	4	3	2	1	C2_14
Online gaming and/or playing games console	5	4	3	2	1	C2_15

Block D: Health related use of Information and Communication Technologies, and the Internet

D1a. Regarding health, wellness and the Internet, how often have you….?

	Every day or almost every day	At least once a week (but not every day)	At least once a month (but not every week)	Less than once a month	Never	I was not aware of it	
looked for information about a physical illness or condition that you or someone you know has	5	4	3	2	1	9	D1a_1
looked for information about wellness or lifestyle	5	4	3	2	1	9	D1a_2
bought medicine or vitamins online	5	4	3	2	1	9	D1a_3
participated in an online support group for people who are concerned about the same health or medical issue	5	4	3	2	1	9	D1a_4
participated in Social Networking Sites talking about health and wellness	5	4	3	2	1	9	D1a_5
used email or gone to a web site to communicate with a doctor or a doctor's office	5	4	3	2	1	9	D1a_6
clicked on a health or medical web site's privacy policy to read about how the site uses personal information	5	4	3	2	1	9	D1a_7
described a medical condition or problem online in order to get advice from an online doctor	5	4	3	2	1	9	D1a_8
described a medical condition or problem online in order to get advice from other online users (peers)	5	4	3	2	1	9	D1a_9
kept a health web site "bookmarked", or saved as	5	4	3	2	1	9	D1a_10

a "favourite place", so you can go back to it regularly							
looked to see what company or organization is providing the advice or information that appears on a health web site	5	4	3	2	1	9	D1a_11
looked for information about a mental health issue like depression or anxiety	5	4	3	2	1	9	D1a_12
disclosed medical information on Social Networking Sites	5	4	3	2	1	9	D1a_13
disclosed medical information on websites to share pictures, videos, movies, etc.	5	4	3	2	1	9	D1a_14

For each reply where D1a_x=1 or 9 do the same for D1b_x

If D1a_1 = 1 and D1a_2= 1 -> D10

If D1a_1 = (2 to 5) or D1a_2 = (2 to 5) ->D2

D1b. Assuming that you were provided the possibility, state how likely it is that you would do the following during the next year?

	Very likely			Very unlikely	
look for information about a physical illness or condition that you or someone you know has	4	3	2	1	D1b_1
look for information about wellness or lifestyle	4	3	2	1	D1b_2
buy medicine or vitamins online	4	3	2	1	D1b_3
participate in an online support group for people who are concerned about the same health or medical issue	4	3	2	1	D1b_4
participate in Social Networking Sites talking about health and wellness	4	3	2	1	D1b_5
use email or gone to a web site to communicate with a doctor or a doctor's office	4	3	2	1	D1b_6
click on a health or medical web site's privacy policy to read	4	3	2	1	D1b_7

about how the site uses personal information					
describe a medical condition or problem online in order to get advice from an online doctor	4	3	2	1	D1b_8
describe a medical condition or problem online in order to get advice from other online users (peers)	4	3	2	1	D1b_9
keep a health web site "bookmarked", or saved as a "favourite place", so you can go back to it regularly	4	3	2	1	D1b_10
look to see what company or organization is providing the advice or information that appears on a health web site	4	3	2	1	D1b_11
look for information about a mental health issue like depression or anxiety	4	3	2	1	D1b_12
disclose medical information on Social Networking Sites	4	3	2	1	D1b_13
disclose medical information on websites to share pictures, videos, movies, etc.	4	3	2	1	D1b_14

D2. Were you looking for health and/or wellness information for yourself or for others? (multiple choice)

	Yes	No	
Yourself	1	2	D2_1
Child	1	2	D2_2
Parent	1	2	D2_3
Another relative	1	2	D2_4
Someone else	1	2	D2_5

If D2_1 = 1 -> D3

If D2_2 =1 or D2_3=1 or D2_4=1 or D2_5=1 ->D4

D3. Did you happen to go looking for this health information for yourself…?

	D3
Before visiting a doctor or clinic	1
After visiting a doctor or clinic	2

Instead of visiting a doctor or clinic	3
Unrelated to visiting a doctor or clinic	4

D4. Did you happen to go looking for this health information for another person…?

	D4
Before visiting a doctor or clinic	1
After visiting a doctor or clinic	2
Instead of visiting a doctor or clinic	3
Unrelated to visiting a doctor or clinic	4

D5. Overall, how USEFUL was the health information you got online

	D5
Very useful	4
Somewhat useful	3
Not too useful	2
Not at all useful	1

D6. Did you learn anything NEW from the information you got online, or not?

	D6
Yes	1
No	2
Don’t know	99

D7. Did you later talk to a doctor or nurse about the information you got online?

	D7
Yes	1
No	2
Don’t know	99

D8. Did the information you got online affect any of your decisions about health treatments or the way you take care of yourself?

	D8
Yes	1
No	2

D9. Did the information you got online affect the way you eat or exercise?

	D9
Yes	1
No	2
Don't know	99

D10a. Regarding health and Information and Communication Technologies, specially the Internet, how often have you….?

	Every day or almost every day	At least once a week (but not every day)	At least once a month (but not every week)	Less than once a month	Never	I was not aware of it	
Made, cancelled or changed an appointment with your family doctor, specialist or other health professionals online	5	4	3	2	1	9	D10_1
Sent or received an email from your doctor, nurse or health care organization	5	4	3	2	1	9	D10_2
Made an online consultation through videoconference with your doctor or nurse	5	4	3	2	1	9	D10_3
Received online the results of your clinical or medical test.	5	4	3	2	1	9	D10_4
Accessed or uploaded your (or any other family member) medical information or health record through an Internet provider (ex. Google Health, Microsoft Vault…)	5	4	3	2	1	9	D10_5
Accessed or uploaded your	5	4	3	2	1	9	D10_6

(or any other family member) medical information or health record through an Internet application provided by your healthcare organization							
Used a game console to play games related with your health or your wellness	5	4	3	2	1	9	D10_7
Used a health/wellness application on your mobile phone	5	4	3	2	1	9	D10_8
Used devices (as pulse meter, glucose meter…) to transmit vital signs or other clinical information and/or received alarms or follow-up about your health anytime, anywhere	5	4	3	2	1	9	D10_9
Received any message about health promotion and/or health prevention	5	4	3	2	1	9	D10_10

For each reply where D10a_x=1 or 9 do the same for D10b_x

D10b. Assuming that you were provided the possibility, state how likely it is that you would do the following during the next year?

	Very likely			Very unlikely	
Make, cancel or change an appointment with your family doctor, specialist or other health professionals online	4	3	2	1	D10b_1
Send or receive an email from your doctor, nurse or health care organization	4	3	2	1	D10b_2
Make an online consultation through videoconference with your doctor or nurse	4	3	2	1	D10b_3
Receive online the results of your clinical or medical test.	4	3	2	1	D10b_4
Access or upload your medical information or health record through an Internet provider (ex. Google Health, Microsoft Vault…)	4	3	2	1	D10b_5
Access or upload your medical	4	3	2	1	D10b_6

information or health record through Internet application provided by your healthcare organization					
Use a game console to play games related with your health or your wellness	4	3	2	1	D10b_7
Use a health/wellness application on your mobile phone	4	3	2	1	D10b_8
Use devices (as pulse meter, glucose meter…) to transmit vital signs or other clinical information and/or received alarms or follow-up about your health anytime, anywhere	4	3	2	1	D10b_9
Receive any message about health promotion and/or health prevention	4	3	2	1	D10b_10

D11. Regardless of whether you have used Information and Communication Technologies for healthcare or wellness purposes, can you tell me how important you believe the following uses of Information and Communication Technologies and the Internet for heath or wellness purposes might be?

	Very important	Somewhat important	Not so important	Not important at all	
To prevent diseases by adopting a healthier lifestyle	4	3	2	1	D11_1
To obtain different points of view from those offered by mainstream medicine	4	3	2	1	D11_2
To better understand a health problem or disease	4	3	2	1	D11_3
To find a specific solution to or treatment for a health problem	4	3	2	1	D11_4
To find additional sources of information (addresses, references or links)	4	3	2	1	D11_5
To participate in online discussions	4	3	2	1	D11_6
To develop one's general knowledge or satisfy one's curiosity	4	3	2	1	D11_7
To help a family member or friend who is ill	4	3	2	1	D11_8
To access an online health service	4	3	2	1	D11_9

D12. Regardless of whether you have used Information and Communication Technologies or the Internet for healthcare or wellness purposes, would you tell us how important the following factors are when evaluating an internet health site?

	Very important	Somewhat important	Not so important	Not important at all	
Secure handling of personal information	4	3	2	1	D12_1
Information in my own language	4	3	2	1	D12_2
Updated information	4	3	2	1	D12_3
Interactivity, e.g. Question-and-answer service, discussion groups, chat	4	3	2	1	D12_4
Health professionals are involved	4	3	2	1	D12_5
Clearly stated who is responsible for sponsoring the site	4	3	2	1	D12_6
Health organizations are involved	4	3	2	1	D12_7
Governments are involved	4	3	2	1	D12_8

D13. Regardless of whether you have used Information and Communication Technologies for healthcare or wellness purposes, would you tell us how important the following barriers are in using these technologies for health or wellness purposes?

	Very important	Somewhat important	Not so important	Not important at all	
Lack of digital skills	4	3	2	1	D13_1
Lack of access to ICT for health applications	4	3	2	1	D13_2
Lack of motivation and interest	4	3	2	1	D13_3
Lack of awareness	4	3	2	1	D13_4
Lack of health literacy	4	3	2	1	D13_5
Lack of trust	4	3	2	1	D13_6
Lack of liability	4	3	2	1	D13_7
Lack of privacy	4	3	2	1	D13_8
Lack of security	4	3	2	1	D13_9
Lack of reliability	4	3	2	1	D13_10

D14. Assuming that you were provided the possibility of looking for health information on the Internet, would information on health or illness which you had obtained from the Internet lead to any of the following?

	Yes	No	Do not know	
Feelings of anxiety	1	2	99	D14_1
Feelings of reassurance or relief	1	2	99	D14_2
Willingness to change diet or other lifestyle habits	1	2	99	D14_3
Suggestions or queries on diagnosis or treatment to your family doctor, specialist or other health professional	1	2	99	D14_4
Changing of use of medicine without consulting your family doctor, specialist or other health professional	1	2	99	D14_5
Making, cancelling or changing an appointment with family doctor, specialist or other health professional	1	2	99	D14_6

D15. To what extent do you agree with the following statements?

	Totally agree	Somewhat agree	Neither agree nor disagree	Somewhat disagree	Totally disagree	
ICT for health could increase my use of the ICT in other fields of my daily life	5	4	3	2	1	D15_1
ICT for health could lead to greater patients satisfaction	5	4	3	2	1	D15_2
ICT for health could improve my health status	5	4	3	2	1	D15_3
ICT for health could improve the ability to take care and monitor my own health	5	4	3	2	1	D15_4
ICT for health could change my behaviours towards a healthy lifestyle	5	4	3	2	1	D15_5
ICT for health could avoid travelling expenses and time	5	4	3	2	1	D15_6
ICT for health could improve the quality of health care services received	5	4	3	2	1	D15_7

Internet health services substitute some of my face-to-face consultations with the physicians	5	4	3	2	1	D15_8
Internet health services complement some of my face-to-face consultations with the physicians	5	4	3	2	1	D15_9
The quality of Internet health services is aligned with the quality of face-to-face services	5	4	3	2	1	D15_10
I have concerns about the kind of personal information shared with physicians or health organizations through the Internet due to privacy and confidentiality issues	5	4	3	2	1	D15_11
In case of need, I would feel more comfortable and safe at home with a remote monitoring system to track my health	5	4	3	2	1	D15_12
I would be willing to pay to access Internet health services for myself or my relatives	5	4	3	2	1	D15_13

Block E: Socio demographic profile of participants

E1. Gender

	E1
Male	1
Female	2

E2. How old are you?

Age:_______________	E2

E3. Which is your country of citizenship?

	E3
National to UK	1

National of other EU member state	2
National of non-EU country	3

E4. Which is your country of birth?

	E4
UK Native	1
Born in another EU member state	2
Born in non-EU country	3

E5. What is your highest level of education completed?

	E5
Primary or lower secondary education [ISCED 0,1 or 2]	1
Upper secondary education [ISCED 3 or 4]	2
Tertiary education [ISCED 5 or 6]	3

E6. Which of these descriptions best describes your situation or applies to what you have been doing for the last month?

	E6
Employed or self-employed (incl. family workers)	1
Unemployed	2
Student (not in the labour force)	3
Other not in the labour force<br>(retired, inactive, in compulsory military service, etc.)	4

If E6= 1 -> E7

If E6 = (2 to 4) -> E8

E7. What is your occupation?

____E7

(Recoded into at least 2-digit ISCO-88 categories)

E8. Region of residence:

Description:_________(Recoded) E8

E9. Type of locality:

	E9
Densely-populated area (Cities and Large towns)	1
Intermediate area (Towns)	2
Thinly-populated area (Villages and Rural)	3

E10. Number of members in the household?

Number:_____________	E10

E11. Of which, number of children under 16 years?

Number:_____________	E11

E12. Of which, number of members over 65 years?

Number:_____________	E12

E13. Which is your average net monthly income?

	E13
GBP: ____________________	1
Do not want to answer	99

Annex 2. Online panel providers

Cint is a privately owned software company that produces and sells market leading, innovate online research products for businesses, organizations and individuals involved in market research. The company specializes in SaaS, web-based software solutions offering efficient, user friendly online sample management and access, as well as online panel management products that are accessible worldwide 24/7. Headquartered in Stockholm, Sweden, Cint has offices across Europe and the USA. The company has an extensive list of clients and partners spanning most of the large market research groups, media and web-based companies, branding and advertising agencies, plus medium and small market research agencies and other organisations involved in market research. Cint's goal is to be the main provider of sampling solutions for online research, through efficient solutions that improve accuracy and reduce both time and cost. The company has launched a whole series of industry firsts that have dramatically reduced clients operating costs and raised standards in transparency and quality. Cint's products comply with ESOMAR, MRS, CASRO, MRA&ARF quality and personal integrity standards, as well as offering additional functions designed to enhance quality. All publicized panels operate within this controlled framework. Cint's Survey Quality Assurance Program ensures all projects by sample buyers are set up correctly and that the questionnaire is of the required standard. Since most data errors in research are made in the survey creation phase, Cint puts an emphasis on quality checking every survey reaching the Cint Panel Exchange network. All major and most minor languages issues are forced to be corrected before the project is launched. Cint's Quality Features:

- Panellist rating: all panellists are scored by their level of survey activity. A high score shows active behaviour, while a lower score shows lower levels of activity. If a score drops to a certain agreed level, panel owners can use this scoring system to automatically clean their panels.
- Automatic cleaning: all panels in Cint Panel Exchange are automatically cleaned on hard bounces, where the email address is proven not to function.
- Random & Stratified Sampling: within the required targets, sample is randomly generated as well as being stratified by high, medium and low responders.
- Quarantine settings: Both panellists and panel owners can set the maximum number of surveys received.
- Exclusions: Panellists are automatically excluded from taking part in surveys in the same subject category or project regardless of panel they belong to.
- Re-invitations: Re-minder send outs to non-participants increase response and sampling efficiency.
- De-duping: Cint de-duping technology is able to detect and remove duplicates when inviting respondents to complete a specific survey.
- Professional panellists: At the registration stage personal information including name, address and other specific information is collected to assist in the validation process. Depending on incentive method used, unique identification data is required to redeem incentives such as: id number, home address and bank details.
- Panel Blending: Sample can be drawn from multiple panels simultaneously to reach hard to find target groups and eliminate source bias, and therefore reaching panellists with different motivation factors. It also allows users to benefit from selecting sample generated by different recruitment methods from CATI recruited panels to panels built from natural online communities, where members have a relationship with the panel owner's brand.
- Panellists survey rating: Panellist can rate every survey on length, language and logic and other errors in surveys. Panellist longevity is reached by respecting their feedback and their experience in taking surveys. This feedback can help buyers to improve the quality of their surveys, which in turn generates high quality results.

- Increased performance and security: As user of a SaaS system all users will get continuous updates and security patches and monitoring.
- Independent study on panel quality: Cint is a contributor to a major industry study on panel quality, conducted by Mkting Inc. The objective of the study is measure panel quality from different providers through asking panellists about their survey behaviour and to measure how buying behaviour results correlates between panels. The early findings are showing that a blended sample, using multiple panel sources, is a more reliable way to conduct online research.

Furthermore, CINT provides the following software and hardware security features:

- All users require username and password secure logins
- The ASP environment has been designed with security, high-availability and performance in mind.
- All servers, services and network are monitored 24/7 by both Cint and the hosting partner with operation teams on stand-by.

Annex 3. Pilot study

A pilot test was conducted to ensure the questionnaire functioned correctly. The test was carried out between July 1 and July 6, 2011. In the end, a total of 231 interviews were completed in Spain and the UK (116 in Spain and 115 in the UK)

The reliability and validity of the questionnaire was tested. The reliability of the questionnaire was assessed in terms of consistency (using Cronbach's α (alpha) analysis as a coefficient of reliability). Cronbach's α (alpha) varies from zero to 1. Higher alpha values are more desirable. It is commonly accepted that a reliability of 0.70 or higher is required before using a tool. Table 11 shows Cronbach's α (alpha) values for the selected variables:

Table 84. Cronbach's α (alpha) values

Name	Question	Cronbach's Alpha Value	Conc.
A11_A_1 to A11_A_3	In general, how often does your usual source of care (doctor or nurse)…	0,928	Valid
A12_A_1 to A12_A3	In general, how often do you ask your usual source of care (doctor or nurse)…	0,962	Valid
B1_A_1 to B1_A_4	For each of the following statements regarding the use of Information and Communication Technologies, specially the Internet, could you please tell me whether you agree or disagree? ICT allows me to…	0,927	Valid
B2_A_1 to B2_A_3	For each of the following statements regarding the use of Information and Communication Technologies, specially the Internet, could you please tell me whether you agree or disagree? ICT helps me …	0,936	Valid
B3_A_1 to B3_A_5	For each of the following statements regarding the use of Information and Communication Technologies, specially the Internet, could you please tell me whether you agree or disagree? CT facilitates…	0,926	Valid
B4_A_1 to B4_A_10	Below you can find a list of various sources of information about health, illness or wellness, and we would like to know how important these are to you…	0,752	Valid
B5_A_1 to B5_A_8	Different authorities (government departments, local authorities, agencies) and private companies could offer health information and online services related with your health. To what extent do you trust the following institutions to protect your personal information?	0,875	Valid
C2_A_1 to C2_A_15	Which of the following Internet related activities have you already carried out?	0,872	Valid
D1_A_1 to D1_A_14	Regarding health, wellness and the Internet, how often you ….?	0,960	Valid
D10A_1 to D10A_10	Regarding health and Information and Communication Technologies, specially the Internet, how often have you….?	0,970	Valid
D11_1 to	Regardless of whether you have used Information and Communication Technologies for healthcare or	0,923	Valid

D11_9	wellness purposes, can you tell me how important you believe the following uses of Information and Communication Technologies and the Internet for heath or wellness purposes might be		
D12_1 to D12_8	Regardless of whether you have used Information and Communication Technologies or the Internet for healthcare or wellness purposes, would you tell us how important the following factors are when evaluating an internet health site?	0,858	Valid
D13_1 to D13_10	Regardless of whether you have used Information and Communication Technologies for healthcare or wellness purposes, would you tell us how important the following barriers are in using these technologies for health or wellness purposes	0,958	Valid
D15_1 To D15_13	To what extent do you agree with the following statements?	0,910	Valid

The validity is the degree to which the questionnaire actually measures what is expected, or serves the purpose for which it has been prepared, and the analysis was carried out according to the content validity, construct validity, and criterion-related validity. After telephone contact with (approximately) 10% of the pilot study sample, the following conclusions were reached:

- ✓ The questionnaire is rather long and repetitive due to the use of many scales
- ✓ The questionnaire deals with an interesting topic that motivates the respondent to answer.
- ✓ There are no relevant problems of understanding

In this sense, the only significant change remarkable in the final questionnaire in relation to the pilot questionnaire is:

- ✓ The inclusion of the option "I was not aware of it" to avoid forcing an answer that would not reflect the real circumstances.

Annex 4. Internet activities comparison

Table 85: Internet access (C2) comparison

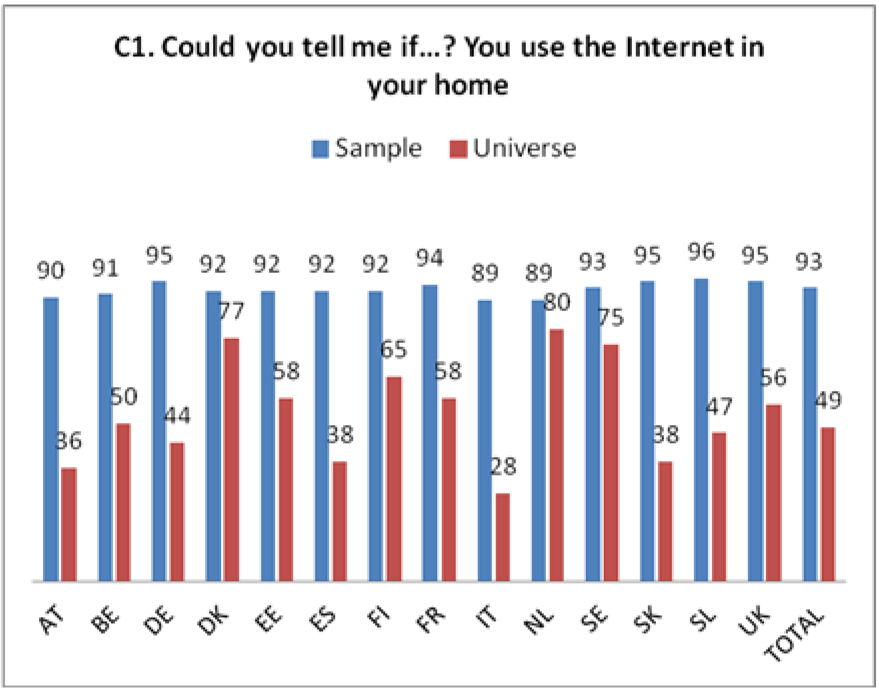

Source: Special Eurobarometer 359.

Table 86: Internet activities (C3) comparison

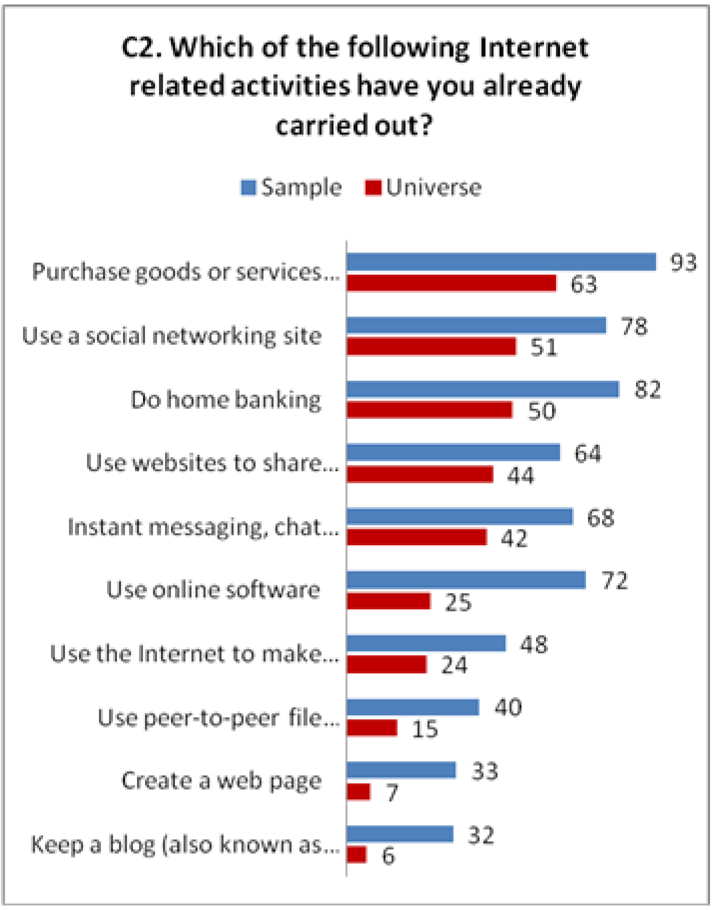

Source: Special Eurobarometer 359.

Table 87: Internet activities (C3) comparison

Yes (%)	AT		BE		DE		DK		EE		ES		FI	
	Sample	Universe	Sample	Universe	Sample	Universe	Sample	Universe	Sample	Universe	Sample	Universe	Sample	Universe
Use the Internet to make telephone calls	57	28	45	22	52	20	44	33	55	35	48	19	51	26
Use peer-to-peer file sharing for exchanging movies, music, etc	36	20	40	16	30	6	34	23	58	14	56	25	46	10
Create a web page	38	13	27	10	39	6	36	12	35	6	39	6	37	10
Use websites to share pictures, videos, movies, etc.	69	45	68	43	60	32	65	40	81	49	77	53	66	35
Use a social networking site	76	49	78	52	80	37	80	63	89	59	88	56	66	51
Purchase goods or services online / online shopping (e.g. travel & holiday, clothes, books, tickets, films, music, software, food)	95	62	86	53	97	72	96	81	86	43	88	39	95	69
Keep a blog (also known as web-log)	33	9	29	8	35	3	27	6	26	7	47	8	25	8
Instant messaging, chat websites	69	31	68	37	69	26	62	39	75	46	80	69	72	34
Do home banking	85	59	88	64	81	47	89	86	97	69	78	40	95	89
Use online software	80	34	71	28	78	29	71	40	88	44	73	17	75	29

Table 88: Internet activities (C3) comparison

Yes (%)	FR		IT		NL		SE		SK		SL		UK	
	Sample	Universe	Sample	Universe	Sample	Universe	Sample	Universe	Sample	Universe	Sample	Universe	Sample	Universe
Use the Internet to make telephone calls	45	33	61	21	42	28	49	28	69	43	57	23	38	18
Use peer-to-peer file sharing for exchanging movies, music, etc	36	16	57	18	43	18	35	26	49	15	68	30	36	11
Create a web page	24	8	40	5	33	12	32	13	35	4	40	7	26	6
Use websites to share pictures, videos, movies, etc.	60	39	71	47	64	46	61	42	80	58	73	50	58	49
Use a social networking site	73	50	80	48	72	53	77	58	86	66	88	53	75	57
Purchase goods or services online / online shopping (e.g. travel & holiday, clothes, books, tickets, films, music, software, food)	93	66	85	35	90	81	96	78	91	52	90	39	98	79
Keep a blog (also known as web-log)	26	8	43	6	30	7	36	10	24	4	31	2	21	4
Instant messaging, chat websites	72	52	76	45	57	25	69	39	83	58	67	47	53	33
Do home banking	84	58	72	27	89	84	95	80	79	41	73	38	85	44
Use online software	63	31	75	15	71	29	74	37	72	16	79	33	69	19

Annex 5: Correlation matrix

Table 89: Internet related activities – Correlation matrix

	Mean	1	2	3	4	5	6	7	8	9	10	11	12	13	14
Use a search engine to find information	4.54														
Send e-mails with attached files	4.00	.357													
Post messages to chatrooms, newsgroups or an online discussion forum	2.59	.180	.205												
Use the Internet to make telephone calls	2.11	.101	.218	.305											
Use peer-to-peer file sharing for exchanging movies, music,...	1.88	.098	.172	.410	.371										
Create a web page	1.64	.036	.161	.356	.381	.434									
Use websites to share pictures, videos, movies, etc.	2.50	.181	.227	.501	.326	.473	.383								
Use a social networking site	3.42	.231	.188	.472	.178	.263	.205	.466							
Purchase goods or services online / online shopping	2.93	.188	.269	.266	.261	.258	.301	.275	.159						
Keep a blog (also known as web-log)	1.71	.044	.144	.437	.371	.426	.581	.431	.286	.269					

Instant messaging, chat websites	2.85	.177	.200	.527	.327	.350	.299	.458	.465	.174	.364				
Do home banking	3.36	.153	.247	.081	.174	.134	.141	.104	.065	.315	.096	.060			
Use online software	2.67	.213	.264	.394	.355	.410	.362	.419	.270	.321	.366	.355	.231		
You use the Internet through your mobile phone	2.44	.165	.194	.319	.304	.360	.298	.363	.334	.285	.293	.321	.192	.356	
Online gaming and/or playing games console	2.67	.089	.041	.302	.190	.291	.217	.293	.237	.157	.249	.301	.066	.310	.233
*p<0,001															

Table 90: Triggers - Correlation matrix

	Mean	1	2	3	4	5	6	7	8
To prevent diseases by adopting a healthier lifestyle	2.99								
To obtain different points of view from those offered by mainstream medicine	2.84	.512							
To better understand a health problem or disease	3.22	.579	.574						
To find a specific solution to treatment for a health problem	2.98	.565	.581	.639					
To find additional sources of information	3.15	.487	.556	.632	.552				
To participate in online discussions	2.32	.367	.484	.346	.386	.405			
To develop one's general knowledge or satisfy one's curiosity	3.12	.494	.532	.637	.519	.610	.389		
To help a family member or friend who is ill	3.06	.578	.525	.621	.627	.522	.339	.518	
To access an online health service	2.74	.507	.503	.473	.516	.493	.500	.444	.485
*p<0,001									

Table 91: Empowerment - Correlation matrix

	Mean	1	2	3	4	5	6	7	8	9	10	11	12	13	14	15	16	17
ICT allow me to be better informed about how to follow the advise of the physician or professionals I consult	3.75																	
ICT allow me to develop a better understanding of my personal health...by giving me access to recognized expert knowledge	3.89	.659																
ICT allow me to become better informed on what is available...so that I can make my own choices	3.94	.639	.672															
ICT allow me to better understand my personal health...through my ability to determine what is relevant	3.81	.650	.696	.654														
ICT allow me to know more about the opinions of people who are in similar situations or who are active in support groups	3.90	.511	.596	.579	.560													
ICT allow me to better understand my personal health through online discussions or the opinions of people going through similar experiences	3.69	.551	.599	.577	.608	.673												
ICT allow me to play a more active role in my exchanges with my physician or the health professionals I consult	3.59	.633	.595	.590	.605	.499	.541											
ICT helps me feel better equipped to implement the advice of the physician or health professionals I consult	3.73	.644	.616	.603	.605	.515	.547	.605										
ICT helps me feel better equipped to make my own choices without being limited to the advice of a physician...	3.60	.569	.552	.586	.580	.465	.505	.553	.662									
ICT helps me feel better equipped to make positive changes to my situation	3.69	.567	.596	.591	.607	.584	.608	.578	.676	.647								

through discussions and exchanges with others																		
ICT helps me feel more confident in playing a more active role in my exchanges with my physician...	3.70	.612	.614	.603	.610	.530	.543	.667	.727	.657	.678							
ICT helps me feel more confident about the choices I plan on making between the various possible treatments and solutions	3.71	.592	.613	.631	.620	.533	.560	.581	.704	.704	.699	.718						
ICT helps me feel more confident in my discussions with the people in my life	3.69	.561	.578	.547	.591	.534	.553	.566	.655	.608	.704	.684	.684					
ICT facilitates making decisions on my health albeit without going against the advice of the physician....	3.61	.519	.519	.508	.529	.455	.479	.502	.593	.580	.571	.571	.599	.550				
ICT facilitates a more active role in my health by deciding which solutions I prefer...mainstream medicine or alternative approaches	3.67	.551	.568	.592	.563	.496	.518	.543	.632	.659	.627	.643	.673	.579	.664			
ICT facilitates making decisions about my health on the basis of my preferences and means rather than only on the advice of my physician	3.50	.510	.500	.520	.520	.439	.485	.501	.572	.660	.576	.579	.629	.540	.635	.716		
ICT facilitates a more active role in my health by continuing to talk with the people in my life who could help me clarify my ideas	3.63	.543	.558	.532	.574	.553	.576	.551	.621	.567	.659	.624	.632	.652	.624	.669	.636	
ICT facilitates making decisions about my health by relying on the experiences...with the people with whom I talk	3.47	.497	.489	.493	.512	.497	.553	.488	.559	.597	.608	.564	.604	.584	.607	.650	.664	.697
*p<0,001																		

Table 92: Barriers – Correlation matrix

	Mean	1	2	3	4	5	6	7	8	9
Lack of digital skills	2.73									
Lack of access to ICT for health applications	2.93	.603								
Lack of motivation and interest	2.94	.555	.563							
Lack of awareness	3.03	.570	.605	.644						
Lack of health literacy	3.06	.526	.550	.587	.631					
Lack of trust	3.25	.436	.516	.552	.592	.597				
Lack of liability	3.11	.487	.526	.550	.594	.587	.663			
Lack of privacy	3.32	.388	.473	.469	.522	.529	.688	.630		
Lack of security	3.31	.423	.495	.500	.559	.556	.717	.659	.772	
Lack of reliability	3.28	.422	.512	.527	.590	.579	.705	.630	.689	.733
*p<0,001										

Table 93: Health information sources – Correlation matrix

	Mean	1	2	3	4	5	6	7	8	9
How important...Internet	3.10									
How important...TV	2.50	.379								
How important...Radio	2.17	.226	.542							
How important...Books, medical encyclopaedias and leaflets	2.91	.331	.285	.284						
How important...Courses and lectures	2.52	.206	.289	.365	.470					
How important...Newspapers, magazines	2.46	.325	.505	.475	.399	.396				
How important...Family, friends and colleagues	2.92	.312	.321	.268	.233	.209	.334			
How important...Pharmacies	3.11	.127	.235	.230	.277	.284	.242	.260		
Direct face to face contact with doctors	3.69	.076	.049	.000	.184	.136	.054	.134	.358	
Direct face to face contact with nurses	3.17	.070	.143	.182	.238	.237	.121	.180	.414	.442
*p<0,001										

Table 94: Trust – Correlation matrix

	Mean	1	2	3	4	5	6	7
National public authorities	2.71							
European institutions	2.53	.626						
Banks and financial institutions	2.31	.508	.447					
Health and medical institutions	3.04	.480	.440	.333				
Shops and department stores	2.07	.302	.314	.418	.260			
Internet comapnies	2.16	.195	.247	.210	.252	.563		
Phone companies, mobile phone companies and ISP	1.94	.330	.318	.477	.237	.634	.549	
Pharmaceutical companies	2.35	.380	.382	.398	.432	.489	.417	.460
*p<0,001								

Table 95: ICT for Health readiness – Correlation matrix

	Mean	1	2	3	4	5	6	7	8	9	10	11	12	13	14	15	16	17	18	19
Looked for information about a physical illness or condition that you or someone you know has	2.41																			
Looked for information about wellness or lifestyle	2.37	.673																		
Bought medicine or vitamins online	1.62	.472	.462																	
Participated in an online support group for people who are concerned about the same health or medical issue	1.56	.554	.532	.668																
Participated in Social Networking Sites talking about health and wellness	1.63	.567	.577	.633	.791															
Used email or gone to a web site to communicate with a doctor's office	1.59	.524	.494	.634	.718	.703														
Clicked on a health or medical web site's privacy policy to read about how the site uses PI	1.71	.555	.530	.597	.683	.678	.665													
Described a medical condition or problem online in order to get advice from an online doctor	1.54	.534	.532	.668	.768	.757	.762	.700												
Described a medical condition or problem online in order to get advice from other online users	1.60	.568	.542	.653	.783	.782	.717	.693	.799											

Kept a health web site "bookmarked", or saved as a "favourite place", so you can go back to it regularly	1.93	.549	.535	.496	.597	.616	.560	.588	.592	.602										
Made, cancelled or changed an appointment with your family doctor, specialist or other health professionals online	1.53	.436	.415	.560	.610	.601	.644	.551	.631	.619	.458									
Sent or received an email from your doctor, nurse or healthcare organization	1.49	.450	.412	.579	.626	.626	.711	.592	.639	.631	.494	.750								
Made an online consultation through videoconference with your doctor or nurse	1.32	.415	.405	.619	.663	.631	.659	.584	.695	.660	.473	.763	.784							
Received online the results of your clinical or medical test	1.38	.427	.419	.589	.644	.629	.665	.592	.682	.657	.494	.749	.783	.865						
Accessed or uploaded your medical information or health record through an IP	1.34	.429	.417	.620	.665	.638	.645	.604	.697	.669	.491	.753	.778	.876	.850					
Accessed or uploaded your medical information or health record through an Internet application provided by your healthcare organization	1.36	.431	.420	.608	.660	.640	.653	.606	.692	.668	.493	.757	.778	.868	.850	.879				
Used a game console to play games related with your health or your wellness	1.46	.384	.404	.547	.581	.574	.552	.523	.590	.593	.438	.636	.651	.737	.713	.733	.738			
Used a health/wellness application on your mobile phone	1.40	.417	.440	.582	.627	.615	.612	.570	.648	.627	.482	.698	.731	.807	.784	.805	.798	.715		

Used devices to transmit clinical information, received alarms, follow-up about your health anytime, anywhere	1.44	.397	.377	.563	.580	.563	.565	.530	.599	.582	.436	.660	.686	.756	.724	.757	.755	.653	.702	
Received any message about health promotion and/or health prevention	1.65	.477	.466	.505	.572	.574	.542	.535	.577	.567	.509	.625	.659	.651	.649	.670	.664	.584	.638	.612

Table 96: ICT for Health willingness - Correlation matrix

	Mean	1	2	3	4	5	6	7	8	9	10	11	12	13	14	15	16	17	18	19	20	21	22	23
look for information about a physical illness or condition that you or someone you know has	1.93																							
look for information about wellness or lifestyle	1.87	.712																						
buy medicine or vitamins online	1.56	.447	.453																					
participate in an online support group for people who are concerned about the same health or medical issue	1.59	.588	.638	.611																				
participate in Social Networking Sites talking	1.59	.562	.638	.644	.898																			

about health and wellness																								
use email or go to a web site to communicate with a doctor's office	1.76	.598	.558	.517	.706	.694																		
click on a health or medical web site's privacy policy to read about how the site uses PI	1.65	.609	.589	.560	.752	.759	.777																	
describe a medical condition or problem online in order to get advice from an online doctor	1.64	.611	.581	.616	.800	.813	.768	.815																
describe a medical condition or problem online in order to get advice from other online users	1.58	.554	.607	.634	.833	.869	.708	.808	.882															
keep a health web site "bookmarked", or save as a "favourite place", so you can go back to it regularly	1.66	.596	.607	.591	.769	.791	.743	.769	.800	.811														
look to see what company or	1.65	.619	.622	.626	.818	.837	.735	.801	.860	.872	.834													

organization is providing the advice or information that appears on a health website																								
look for information about a mental health issue like depression or anxiety	1.66	.643	.643	.595	.782	.793	.642	.736	.763	.828	.741	.817												
disclose medical information on Social Networking Sites	1.55	.533	.606	.647	.817	.842	.667	.746	.809	.905	.795	.825	.806											
disclose medical information on websites to share pictures, videos, movies, etc.	1.54	.536	.612	.661	.839	.852	.646	.758	.810	.905	.791	.830	.809	.957										
Make, cancel or change an appointment with your family doctor, specialist or other health professionals online	1.97	.449	.347	.267	.368	.326	.483	.446	.422	.378	.390	.425	.364	.312	.309									
Send or receive an email from your doctor, nurse or healthcare organization	1.88	.476	.384	.314	.408	.382	.519	.461	.468	.435	.450	.458	.439	.397	.386	.807								

Make an online consultation through videoconference with your doctor or nurse	1.65	.414	.341	.323	.426	.409	.491	.483	.530	.482	.491	.481	.408	.430	.414	.669	.755							
Receive online the results of your clinical or medical test	1.83	.470	.356	.280	.409	.363	.493	.442	.468	.423	.425	.444	.407	.386	.367	.772	.845	.782						
Access or upload your medical information or health record through an IP	1.69	.401	.307	.282	.388	.366	.429	.461	.419	.434	.416	.408	.393	.410	.400	.661	.749	.799	.782					
Access or upload your medical information or health record through an Internet application provided by your healthcare organization	1.71	.459	.360	.275	.396	.372	.478	.472	.456	.444	.428	.433	.442	.441	.401	.692	.787	.800	.822	.895				
Use a game console to play games related with your health or your wellness	1.59	.361	.373	.293	.427	.441	.387	.409	.421	.483	.481	.448	.430	.483	.472	.525	.597	.785	.588	.708	.690			
Use a health/wellness application on your mobile phone	1.60	.355	.384	.315	.446	.449	.412	.440	.450	.501	.471	.470	.445	.504	.505	.593	.658	.822	.657	.781	.765	.859		

Use devices to transmit clinical information, receive alarms, follow-up about your health anytime, anywhere	1.66	.387	.374	.253	.419	.394	.409	.440	.443	.474	.444	.424	.449	.447	.423	.601	.726	.790	.720	.785	.808	.755	.808	
Receive any message about health promotion and/or health prevention	1.67	.437	.407	.302	.454	.426	.491	.486	.470	.492	.474	.466	.439	.462	.455	.660	.751	.826	.758	.810	.805	.779	.832	.842
*p<0,001																								

Table 97: ICT for Health assessment – Correlation matrix

	Mean	1	2	3	4	5	6
Secure handling of PI	3.58						
Information in my own language	3.49	.604					
Updated information	3.50	.673	.622				
Health professionals are involved	3.39	.620	.560	.673			
Clearly stated who is responsible for sponsoring the site	3.04	.422	.370	.464	.485		
Health organizations are involved	3.11	.480	.461	.537	.629	.512	
Governments are involved	2.57	.210	.230	.235	.312	.387	.493
*p<0,001							

Table 98: ICT for Health impact – Correlation matrix

	Mean	1	2	3	4	5	6	7	8	9		
ICT for health could increase my use of the ICT in other fields of my daily life	3.19											
ICT for health could lead to greater patients satisfaction	3.48	.595										
ICT for health could improve my health status	3.25	.618	.642									
ICT for health could improve the ability to take care and monitor my own health	3.50	.597	.668	.689								
ICT for health could change my behaviours towards a healthy lifestyle	3.40	.608	.615	.672	.698							
ICT for health could avoid travelling expenses and time	3.57	.522	.622	.554	.601	.560						
ICT for health could improve the quality of health care services received	3.47	.583	.692	.625	.663	.623	.639					
Internet health services substitute some of my face-to-face consultations with the physicians	2.76	.473	.471	.526	.451	.439	.454	.474				
Internet health services complement some of my face-to-face consultations with the	3.40	.494	.583	.545	.574	.534	.534	.585	.513			

physicians												
The quality of Internet health services is aligned with the quality of f2f sevices	2.99	.502	.516	.530	.492	.489	.477	.522	.570	.511		
I would feel more comfortable and safe at home with a remote monitoring system to track my heath	3.16	.490	.494	.502	.512	.480	.456	.495	.418	.441	.414	
I would be willing to pay to access Internet health services for myself or relatives	2.46	.447	.384	.440	.368	.392	.330	.374	.504	.359	.458	.447
*p<0,001												

European Commission
EUR 25427 - Joint Research Centre – Institute for Prospective Technological Studies

Title: Strategic Intelligence Monitor on Personal Health Systems, Phase 2. Citizens and ICT for Health in 14 European Countries: Results from an Online Panel

Authors: Francisco Lupiañez, Ioannis Maghiros, Fabienne Abadie

Luxembourg: Publications Office of the European Union

2013 – 211 pp. – 21.0 x 29.7 cm

EUR – Scientific and Technical Research series – ISSN 1831-9424 (online)

ISBN 978-92-79-25709-4 (pdf)

doi:10.2791/84062

Abstract
The Citizen Panel Survey carried out in SIMPHS2 to better assess users and patients' needs and expectations with regard to ICT for health, directly supports the objectives of the Digital Agenda in the area of eHealth which are to both cope with societal challenges and create opportunities for innovation and economic growth by reducing health inequalities, promoting active and healthy ageing and increasing empowerment. It also contributes to the goals of the European Innovation Partnership on Active and Healthy Aging which addresses the societal challenge of an ageing population focusing on the main areas of life events (Prevention, Care and cure and Independent living) with the following expected results:

- An improvement of the health status and quality of life of Europeans, especially older people;
- An improvement of the sustainability and efficiency of health and social care systems;
- Boosted EU competitiveness through an improved business environment for innovation.

In this policy context the analysis of users' demand undertaken through the SIMPHS2 Citizen panel survey aims to:

- develop typologies of digital healthcare users and measure the impact of ICT and the Internet on health status, health care demand and health management.
- identify factors that can enhance or inhibit the role and use of Personal Health Systems from a citizen' s perspective with special emphasis on mHealth, RMT, disease management, Telecare, Telemedicine and Wellness.

To reach these objectives, we started by defining a theoretical framework for policy-making, which was used to design and gather relevant information. A multivariate statistical analysis was subsequently carried out to identify the underlying conceptual dimensions emerging from the data collected. Key relationships between concepts (underlying dimensions) were identified to understand ICT for Health as a complex ecosystem. We concluded with some lessons learned.

LF-NA-25427-EN-N

As the Commission's in-house science service, the Joint Research Centre's mission is to provide EU policies with independent, evidence-based scientific and technical support throughout the whole policy cycle.

Working in close cooperation with policy Directorates-General, the JRC addresses key societal challenges while stimulating innovation through developing new standards, methods and tools, and sharing and transferring its know-how to the Member States and international community.

Key policy areas include: environment and climate change; energy and transport; agriculture and food security; health and consumer protection; information society and digital agenda; safety and security including nuclear; all supported through a cross-cutting and multi-disciplinary approach.

ISBN 978-92-79-25709-4

9 789279 257094

Publications Office
